# Synthesis, telomerase inhibitory and anticancer activity of new 2-phenyl-4H-chromone derivatives containing 1,3,4-oxadiazole moiety

**DOI:** 10.1080/14756366.2020.1864630

**Published:** 2020-12-27

**Authors:** Xu Han, Yun Long Yu, Duo Ma, Zhao Yan Zhang, Xin Hua Liu

**Affiliations:** School of Pharmacy, Anhui Province Key Laboratory of Major Autoimmune Diseases, Anhui Medical University, Hefei, P. R. China

**Keywords:** 2-phenyl-4H-chromone, synthesis, telomerase inhibitor, anticancer activity, dyskerin

## Abstract

Based on previous studies, 66 2-phenyl-4H-chromone derivatives containing amide and 1,3,4-oxadiazole moieties were prepared as potential telomerase inhibitors. The results showed most of the title compounds exhibited significantly inhibitory activity on telomerase. Among them, some compounds demonstrated the most potent telomerase inhibitory activity (IC_50_ < 1 µM), which was significantly superior to the staurosporine (IC_50_ = 6.41 µM). In addition, clear structure–activity relationships were summarised, indicating that the substitution of the methoxy group and the position, type and number of the substituents on the phenyl ring had significant effects on telomerase activity. Among them, compound **A33** showed considerable inhibition against telomerase. Flow cytometric analysis showed that compound **A33** could arrest MGC-803 cell cycle at G2/M phase and induce apoptosis in a concentration-dependent way. Meanwhile, Western blotting revealed that this compound could reduce the expression of dyskerin, which is a fragment of telomerase.

## 1. Introduction

Telomerase is a ribonucleoprotein that exists in mammalian cells, playing an important role in maintaining the length of stable telomere and the chromosomal integrity of frequently dividing cells^1^. It is almost undetectable in most somatic cells with the exception of some adult pluripotent stem cells and male germline cells^2^^,^^3^. However, in 85–90% of primary tumours, telomerase is reactivated, so that the ends of chromosomes are maintained during cells proliferation, which results in unlimited proliferation and immortalisation of tumour cells^4^. Therefore, telomerase is regarded as an effective drug target^5^. Regulating the stability of telomerase G-quadruplex as anticancer agents have been widely reported^6–13^.

A lot of studies confirmed that dyskerin, fragment protein of telomerase was essential for telomerase activity, which allowed the correct assembly and stabilisation of mature human telomerase RNA (hTR)^14^. Highly expressed dyskerin was closely related to the occurrence and development of various tumours^15–17^. Considering that most cancers rely on the holoenzyme telomerase to promote tumorigenesis and development, and that dyskerin was closely related to the maintenance of telomeres. So, this protein was a potential target for development of anticancer therapies^18^.

**Figure 1.**
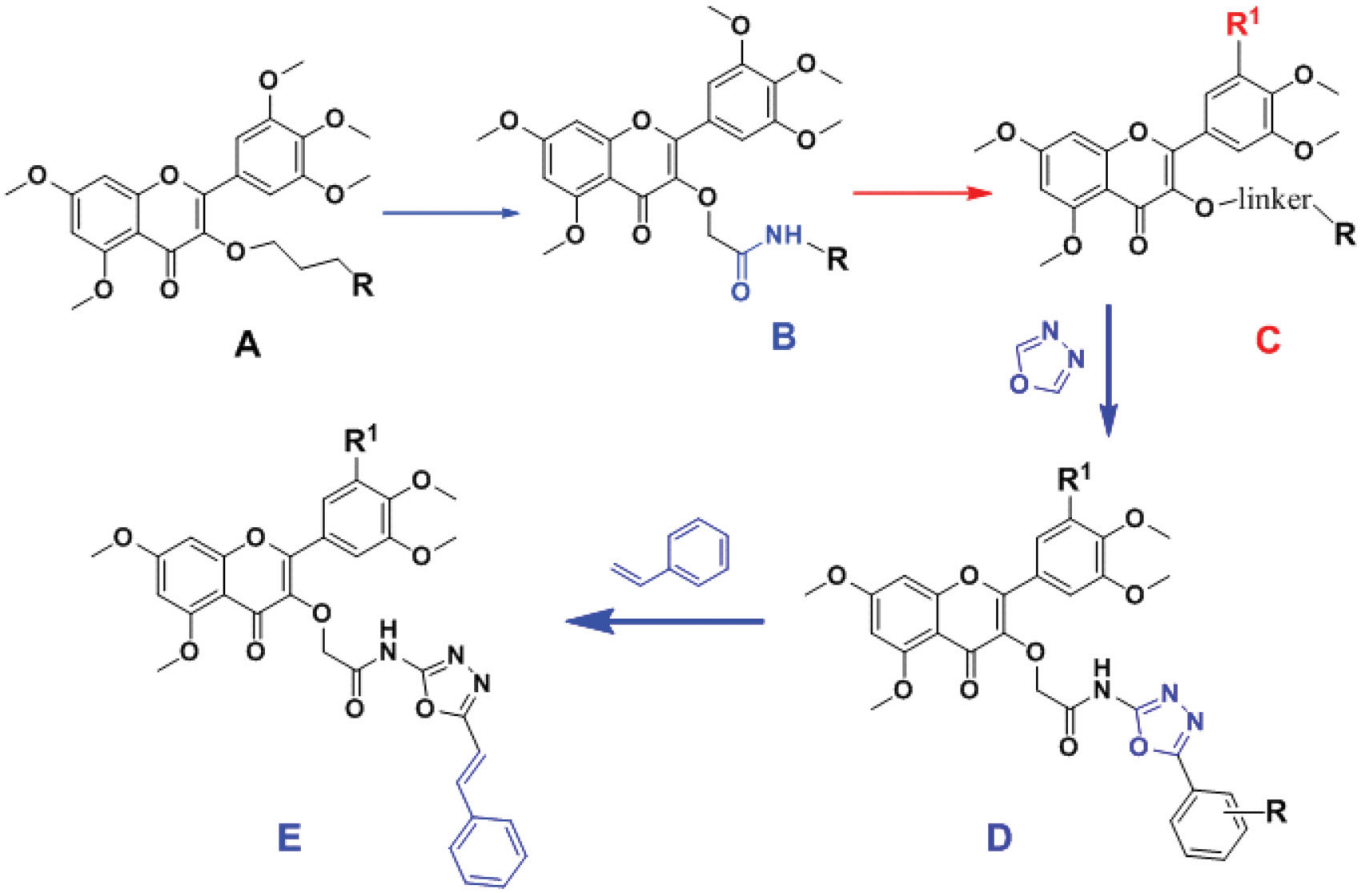
Design of the title compounds.

Several studies had shown that some flavonoid derivatives had strong telomerase inhibitory activity and extensive antitumor activity^19–24^. In our previous work^22^, myricetin derivatives exhibited moderate telomerase inhibitory activity (Figure 1(A)), and the preliminary structure–activity relationships (SARs) showed that the introduction of amide segment could significantly change the telomerase inhibitory activity and cytotoxicity. This indicated that the linker should be involved in the improvement of inhibitory activity (Figure 1(B)). In addition, the amount of methoxy groups on the benzene ring has an essential effect on antitumor activity, such as natural **A4**. Therefore, on basis of the above, the optimisation design of the structure was carried out in this study (Figure 1(C)).

As is known to us, 1,3,4-oxadiazole as a privileged scaffold was used extensively in drugs discovery^25–28^. It was often used as bioisosteres for compounds containing carbonyl such as esters and amides, participating in hydrogen bonding interactions with the receptors^29–33^. Furthermore, different substituted 1,3,4-oxadiazole derivatives with potent antitumor activity have been confirmed (Figure 1(D)). Therefore, 2-phenyl-4H-chromone used as a basic scaffold, following by adjusting the number and substitution positions of OCH_3_ and H on the phenyl ring, retaining the amide fragment as a linker, then introducing 1,3,4-oxadiazole heterocycle and continuing unsaturated substituent. At last, a series of new 2-phenyl-4H-chromone derivatives were designed and synthesised in this study (Figure 1(E)). Their telomerase inhibitory activity was evaluated, and the SAR was widely discussed. In addition, some compounds were selected to screen for their anticancer activity and explore the possible mechanism.

## 2. Experimental section

### 2.1. Chemistry

All reagents and solvents were purchased from standard commercial suppliers and used without further purification. The reactions were monitored by thin-layer chromatography (TLC) on pre-coated silica GF254 plates and visualised under UV light at 254 and 365 nm. Melting points (uncorrected) were determined on a XT4MP apparatus (Taike Corp., Beijing, China). ^1^H and ^13 ^C NMR spectral data were recorded on a Bruker 400 MHz or an Agilent 600 MHz spectrometer in CDCl_3_ or DMSO-*d_6_* using tetramethylsilane (TMS) as the internal standard at room temperature. High-resolution mass spectrometry (HRMS) was recorded on an Agilent Technologies LC-TOF instrument (Supporting Material). X-ray crystallographic data were collected on a Bruker SMART APEX-II CCD diffractometer.

### 2.2. General procedure for synthesis of title compounds A1-A33 and B1-B33

To a solution of the intermediate **1** (0.5 mmol, in acetone (20 ml), the intermediate **4** (0.48 mmol), K_2_CO_3_ (0.96 mmol) and KI (cat) were added. The reaction mixture was stirred at the reflux temperature for 12 h, monitored by TLC. After the reaction was completed, the reaction mixture was cooled to room temperature, diluted with water, extracted with CH_2_Cl_2_ (50 ml × 3), and washed with saturated sodium chloride. The combined organic layers were dried over anhydrous sodium sulphate, filtered, and concentrated under reduced pressure. The crude residue was purified by flash chromatography (DCM: MeOH = 25:1, v/v), and then recrystallized by ethanol to give title compounds **A1**–**A33**. The title compounds **B1**–**B33** could be obtained according to the same procedure.

*2–(5,7-dimethoxy-4-oxo-2–(3,4,5-trimethoxyphenyl)-4H-chromen-3-yloxy)-N-(5-phenyl-1,3,4-oxadiazol-2-yl)acetamide* **(A1)**. White solid, 46.23% yield, m.p.: 222–224 °C; ^1^H NMR (400 MHz, CDCl_3_) δ 12.62 (s, 1H), 8.12–8.06 (m, 2H), 7.54–7.45 (m, 3H), 7.25 (s, 2H), 6.57 (d, *J* = 2.2 Hz, 1H), 6.42 (d, *J* = 2.2 Hz, 1H), 4.40 (s, 2H), 4.00 (s, 3H), 3.95 (s, 3H), 3.94 (s, 6H), 3.93 (s, 3H). ^13 ^C NMR (151 MHz, CDCl_3_) δ 174.6, 166.6, 165.1, 161.5, 161.1, 159.1, 156.9, 154.4, 153.6 (2 C), 141.1, 141.1, 131.3, 128.9 (2 C), 126.7(2 C), 124.4, 123.9, 108.5, 105.9 (2 C), 96.5, 92.8, 73.4, 61.1, 56.6, 56.5(2 C), 55.9. HRMS (ESI): *m/z* [M + H]^+^ calcd for C_30_H_28_N_3_O_10_: 590.1769; found: 590.1767.

*2–(5,7-dimethoxy-4-oxo-2–(3,4,5-trimethoxyphenyl)-4H-chromen-3-yloxy)-N-(5–(4-fluorophenyl)-1,3,4-oxadiazol-2-yl)acetamide* **(A2)**. White solid, 47.60% yield, m.p.: 221–223 °C; ^1^H NMR (600 MHz, CDCl_3_) δ 12.56 (s, 1H), 8.11–8.07 (m, 2H), 7.25 (s, 2H), 7.18 (t, *J* = 8.6 Hz, 2H), 6.57 (d, *J* = 2.1 Hz, 1H), 6.43 (d, *J* = 2.1 Hz, 1H), 4.41 (s, 2H), 4.00 (s, 3H), 3.96 (s, 3H), 3.95 (s, 6H), 3.94 (s, 3H). ^13 ^C NMR (151 MHz, CDCl_3_) δ 174.7, 166.7, 165.1, 164.6 (d, *J* = 252.6 Hz), 161.1, 160.7, 159.1, 156.9, 154.4, 153.6 (2 C), 141.1 (2 C), 128.9 (d, *J* = 8.8 Hz) (2 C), 124.4, 120.2 (d, *J* = 3.2 Hz), 116.2 (d, *J* = 22.4 Hz) (2 C), 108.5, 105.9 (2 C), 96.5, 92.8, 73.4, 61.06, 56.6, 56.5 (2 C), 55.9. HRMS (ESI): *m/z* [M + H]^+^ calcd for C_30_H_27_FN_3_O_10_: 608.1675; found: 608.1674.

*2–(5,7-dimethoxy-4-oxo-2–(3,4,5-trimethoxyphenyl)-4H-chromen-3-yloxy)-N-(5–(3-fluorophenyl)-1,3,4-oxadiazol-2-yl)acetamide* **(A3)**. White solid, 35.83% yield, m.p.: 217–219 °C; ^1^H NMR (400 MHz, CDCl_3_) δ 12.77 (s, 1H), 7.91–7.87 (m, 1H), 7.79 (ddd, *J* = 9.2, 2.5, 1.5 Hz, 1H), 7.47 (td, *J* = 8.1, 5.6 Hz, 1H), 7.25 (s, 2H), 7.21 (tdd, *J* = 8.4, 2.6, 0.9 Hz, 1H), 6.57 (d, *J* = 2.2 Hz, 1H), 6.43 (d, *J* = 2.2 Hz, 1H), 4.40 (s, 2H), 4.01 (s, 3H), 3.96 (s, 3H), 3.95 (s, 6H), 3.94 (s, 3H). ^13 ^C NMR (151 MHz, CDCl_3_) δ 174.6, 166.6, 165.1, 162.8 (d, *J* = 247.6 Hz), 161.2, 160.5, 159.1, 157.1, 154.4, 153.6 (2 C), 141.3, 141.1, 130.7 (d, *J* = 7.2 Hz), 125.8 (d, *J* = 8.2 Hz), 124.4, 122.4, 118.3 (d, *J* = 20.9 Hz), 113.7 (d, *J* = 24.3 Hz), 108.5, 106.2 (2 C), 96.5, 92.8, 73.4, 60.99, 56.5 (3 C), 55.9. HRMS (ESI): *m/z* [M + H]^+^ calcd for C_30_H_27_FN_3_O_10_: 608.1675; found: 608.1672.

*2–(5,7-dimethoxy-4-oxo-2–(3,4,5-trimethoxyphenyl)-4H-chromen-3-yloxy)-N-(5–(2-fluorophenyl)-1,3,4-oxadiazol-2-yl)acetamide* **(A4)**. White solid, 44.80% yield, m.p.: 213–215 °C; ^1^H NMR (600 MHz, CDCl_3_) δ 12.73 (s, 1H), 8.04 (t, *J* = 7.0 Hz, 1H), 7.49 (dd, *J* = 12.0, 6.8 Hz, 1H), 7.27–7.20 (m, 4H), 6.54 (d, *J* = 1.4 Hz, 1H), 6.40 (s, 1H), 4.39 (s, 2H), 3.98 (s, 3H), 3.96–3.90 (m, 12H). ^13 ^C NMR (151 MHz, CDCl_3_) δ 174.6, 166.7, 165.1, 161.1, 159.9 (d, *J* = 258.4 Hz), 159.0, 158.1 (d, *J* = 4.9 Hz), 157.2, 154.4, 153.5 (2 C), 141.1, 141.0, 133.1 (d, *J* = 8.2 Hz), 129.6, 124.5 (d, *J* = 3.3 Hz), 124.4, 116.8 (d, *J* = 20.8 Hz),112.4 (d, *J* = 11.9 Hz), 108.4, 105.97 (2 C), 96.47, 92.72, 73.36, 61.01, 56.50 (3 C), 55.94. HRMS (ESI): *m/z* [M + H]^+^ calcd for C_30_H_27_FN_3_O_10_: 608.1675; found: 608.1671.

*N-(5–(4-chlorophenyl)-1,3,4-oxadiazol-2-yl)-2–(5,7-dimethoxy-4-oxo-2–(3,4,5-trimethoxyphenyl)-4H-chromen-3-yloxy)acetamide* **(A5)**. White solid, 49.06% yield, m.p.: 227–229 °C; ^1^H NMR (600 MHz, CDCl_3_) δ 12.69 (s, 1H), 8.02 (d, *J* = 8.5 Hz, 2H), 7.47 (d, *J* = 8.5 Hz, 2H), 7.25 (s, 2H), 6.56 (d, *J* = 1.9 Hz, 1H), 6.42 (d, *J* = 1.7 Hz, 1H), 4.40 (s, 2H), 4.00 (s, 3H), 3.99–3.90 (m, 12H). ^13 ^C NMR (151 MHz, CDCl_3_) δ 174.7, 166.6, 165.1, 161.1, 160.7, 159.1, 157.0, 154.5, 153.6 (2 C), 141.2, 141.1, 137.6, 129.3 (2 C), 127.9 (2 C), 124.4, 122.3, 108.5, 106.0 (2 C), 96.5, 92.8, 73.4, 61.1, 56.6, 56.5(2 C), 55.9. HRMS (ESI): *m/z* [M + H]^+^ calcd for C_30_H_27_ClN_3_O_10_: 624.1379; found: 624.1376.

*N-(5–(3-chlorophenyl)-1,3,4-oxadiazol-2-yl)-2–(5,7-dimethoxy-4-oxo-2–(3,4,5-trimethoxyphenyl)-4H-chromen-3-yloxy)acetamide* **(A6)**. White solid, 52.33% yield, m.p.: 206–208 °C; ^1^H NMR (400 MHz, CDCl_3_) δ 12.81 (s, 1H), 8.07 (t, *J* = 1.6 Hz, 1H), 7.99 (dt, *J* = 7.5, 1.2 Hz, 1H), 7.50–7.46 (m, 1H), 7.43 (t, *J* = 7.8 Hz, 1H), 7.25 (s, 2H), 6.57 (d, *J* = 2.0 Hz, 1H), 6.42 (d, *J* = 2.0 Hz, 1H), 4.40 (s, 2H), 4.01 (s, 3H), 3.95 (s, 3H), 3.94 (d, *J* = 2.7 Hz, 6H), 3.93 (s, 3H). ^13 ^C NMR (151 MHz, CDCl_3_) δ 174.7, 166.6, 165.1, 161.1, 160.2, 159.0, 157.2, 154.4, 153.6 (2 C), 141.2, 141.1, 135.0, 131.3, 130.2, 126.6, 125.5, 124.8, 124.4, 108.4, 106.0 (2 C), 96.5, 92.8, 73.4, 61.0, 56.5 (3 C), 55.9. HRMS (ESI): *m/z* [M + H]^+^ calcd for C_30_H_27_ClN_3_O_10_: 624.1379; found: 624.1375.

*N-(5–(2-chlorophenyl)-1,3,4-oxadiazol-2-yl)-2–(5,7-dimethoxy-4-oxo-2–(3,4,5-trimethoxyphenyl)-4H-chromen-3-yloxy)acetamide* **(A7)**. White solid, 43.61% yield, m.p.: 201–203 °C; ^1^H NMR (600 MHz, CDCl_3_) δ 12.78 (s, 1H), 7.99 (d, *J* = 7.7 Hz, 1H), 7.53 (d, *J* = 8.0 Hz, 1H), 7.47–7.36 (m, 2H), 7.25 (s, 2H), 6.56 (d, *J* = 1.9 Hz, 1H), 6.41 (d, *J* = 1.7 Hz, 1H), 4.40 (s, 2H), 3.99 (s, 3H), 3.97–3.91 (m, 12H). ^13 ^C NMR (151 MHz, CDCl_3_) δ 174.7, 166.6, 165.1, 161.2, 159.7, 159.0, 157.3, 154.4, 153.6 (2 C), 141.1, 141.1, 133.2, 132.0, 131.2, 131.0, 126.9, 124.4, 123.2, 108.5, 106.0 (2 C), 96.5, 92.7, 73.4, 61.1, 56.5 (3 C), 55.9. HRMS (ESI): *m/z* [M + H]^+^ calcd for C_30_H_27_ClN_3_O_10_: 624.1379; found: 624.1377.

*N-(5–(4-bromophenyl)-1,3,4-oxadiazol-2-yl)-2–(5,7-dimethoxy-4-oxo-2–(3,4,5-trimethoxyphenyl)-4H-chromen-3-yloxy)acetamide* **(A8)**. White solid, 50.89% yield, m.p.: 234–236 °C; ^1^H NMR (600 MHz, CDCl_3_) δ 12.67 (s, 1H), 7.96 (d, *J* = 8.4 Hz, 2H), 7.63 (d, *J* = 8.4 Hz, 2H), 7.25 (s, 2H), 6.57 (s, 1H), 6.43 (s, 1H), 4.40 (s, 2H), 4.00 (s, 3H), 3.96 (s, 3H), 3.95 (s, 6H), 3.94 (s, 3H). ^13 ^C NMR (151 MHz, CDCl_3_) δ 174.7, 166.6, 165.1, 161.1, 160.8, 159.1, 157.0, 154.5, 153.6 (2 C), 141.2, 141.1, 132.2 (2 C), 128.1 (2 C), 125.9, 124.4, 122.8, 108.5, 106.0 (2 C), 96.5, 92.8, 73.4, 61.1, 56.6, 56.5 (2 C), 55.9. HRMS (ESI): *m/z* [M + H]^+^ calcd for C_30_H_27_BrN_3_O_10_: 668.0874; found: 668.0873.

*N-(5–(3-bromophenyl)-1,3,4-oxadiazol-2-yl)-2–(5,7-dimethoxy-4-oxo-2–(3,4,5-trimethoxyphenyl)-4H-chromen-3-yloxy)acetamide* **(A9)**. White solid, 45.80% yield, m.p.: 202–204 °C; ^1^H NMR (400 MHz, CDCl_3_) δ 12.84 (s, 1H), 8.23 (s, 1H), 8.03 (d, *J* = 7.8 Hz, 1H), 7.66–7.61 (m, 1H), 7.36 (t, *J* = 7.9 Hz, 1H), 7.25 (s, 2H), 6.57 (d, *J* = 2.0 Hz, 1H), 6.42 (d, *J* = 2.1 Hz, 1H), 4.40 (s, 2H), 4.01 (s, 3H), 3.96 (s, 3H), 3.94 (s, 6H), 3.93 (s, 3H). ^13 ^C NMR (151 MHz, CDCl_3_) δ 174.7, 166.6, 165.1, 161.2, 160.1, 159.1, 157.2, 154.5, 153.6 (2 C), 141.2, 141.1, 134.3, 130.4, 129.5, 125.7, 125.2, 124.4, 122.9, 108.5, 106.0 (2 C), 96.5, 92.8, 73.4, 61.0, 56.6, 56.5 (2 C), 55.9. HRMS (ESI): *m/z* [M + H]^+^ calcd for C_30_H_27_BrN_3_O_10_: 668.0874; found: 668.0871.

*N-(5–(2-bromophenyl)-1,3,4-oxadiazol-2-yl)-2–(5,7-dimethoxy-4-oxo-2–(3,4,5-trimethoxyphenyl)-4H-chromen-3-yloxy)acetamide* **(A10)**. White solid, 53.94% yield, m.p.: 199–201 °C; ^1^H NMR (600 MHz, CDCl_3_) δ 12.77 (s, 1H), 7.93 (d, *J* = 7.7 Hz, 1H), 7.73 (d, *J* = 8.0 Hz, 1H), 7.43 (t, *J* = 7.6 Hz, 1H), 7.35 (dd, *J* = 10.9, 4.5 Hz, 1H), 7.24 (s, 2H), 6.55 (d, *J* = 1.9 Hz, 1H), 6.41 (d, *J* = 1.8 Hz, 1H), 4.40 (s, 2H), 3.98 (s, 3H), 3.96–3.90 (m, 12H). ^13 ^C NMR (151 MHz, CDCl_3_) δ 174.6, 166.6, 165.1, 161.2, 160.2, 159.0, 157.3, 154.4, 153.6 (2 C), 141.2, 141.1, 134.3, 132.1, 131.6, 127.4, 125.4, 124.4, 121.7, 108.5, 106.1 (2 C), 96.5, 92.8, 73.4, 61.0, 56.6 (2 C), 56.5, 55.9. HRMS (ESI): *m/z* [M + H]^+^ calcd for C_30_H_27_BrN_3_O_10_: 668.0874; found: 668.0873.

*2–(5,7-dimethoxy-4-oxo-2–(3,4,5-trimethoxyphenyl)-4H-chromen-3-yloxy)-N-(5-p-tolyl-1,3,4-oxadiazol-2-yl)acetamide* **(A11)**. White solid, 46.21% yield, m.p.: 225–227 °C; ^1^H NMR (600 MHz, CDCl_3_) δ 12.45 (s, 1H), 7.98 (d, *J* = 8.1 Hz, 2H), 7.29 (d, *J* = 8.0 Hz, 2H), 7.25 (s, 2H), 6.57 (d, *J* = 2.0 Hz, 1H), 6.43 (d, *J* = 1.9 Hz, 1H), 4.41 (s, 2H), 4.00 (s, 3H), 3.96 (s, 3H), 3.95 (s, 6H), 3.94 (s, 3H), 2.42 (s, 3H). ^13 ^C NMR (151 MHz, CDCl_3_) δ 174.6, 166.6, 165.0, 161.7, 161.1, 159.1, 156.6, 154.4, 153.6 (2 C), 141.8, 141.1 (2 C), 129.6 (2 C), 126.7 (2 C), 124.4, 121.1, 108.5, 105.9 (2 C), 96.5, 92.7, 73.4, 61.1, 56.6, 56.5 (2 C), 55.9, 21.6. HRMS (ESI): *m/z* [M + H]^+^ calcd for C_31_H_30_N_3_O_10_: 604.1926; found: 604.1922.

*2–(5,7-dimethoxy-4-oxo-2–(3,4,5-trimethoxyphenyl)-4H-chromen-3-yloxy)-N-(5-m-tolyl-1,3,4-oxadiazol-2-yl)acetamide* **(A12)**. White solid, 42.83% yield, m.p.: 197–199 °C; ^1^H NMR (400 MHz, CDCl_3_) δ 12.60 (s, 1H), 7.92 (s, 1H), 7.88 (d, *J* = 7.6 Hz, 1H), 7.37 (t, *J* = 7.6 Hz, 1H), 7.31 (d, *J* = 7.7 Hz, 1H), 7.25 (s, 2H), 6.57 (d, *J* = 2.2 Hz, 1H), 6.42 (d, *J* = 2.2 Hz, 1H), 4.40 (s, 2H), 4.00 (s, 3H), 3.96 (s, 3H), 3.95 (s, 6H), 3.93 (s, 3H), 2.42 (s, 3H). ^13^C NMR (151 MHz, CDCl_3_) δ 174.6, 166.6, 165.1, 161.7, 161.2, 159.1, 156.8, 154.4, 153.6 (2 C), 141.1, 141.1, 138.7, 132.1, 128.8, 127.2, 124.4, 123.9, 123.7, 108.5, 106.0 (2 C), 96.5, 92.8, 73.4, 61.1, 56.5, 56.5 (2 C), 55.9, 21.2. HRMS (ESI): *m/z* [M + H]^+^ calcd for C_31_H_30_N_3_O_10_: 604.1926; found: 604.1924.

*2–(5,7-dimethoxy-4-oxo-2–(3,4,5-trimethoxyphenyl)-4H-chromen-3-yloxy)-N-(5-o-tolyl-1,3,4-oxadiazol-2-yl)acetamide* **(A13)**. White solid, 45.08% yield, m.p.: 205–207 °C; ^1^H NMR (400 MHz, CDCl_3_) δ 12.64 (s, 1H), 8.00–7.95 (m, 1H), 7.42–7.36 (m, 1H), 7.34 − 7.27 (m, 2H), 7.25 (s, 2H), 6.56 (d, *J* = 2.2 Hz, 1H), 6.41 (d, *J* = 2.2 Hz, 1H), 4.40 (s, 2H), 3.99 (s, 3H), 3.95 (s, 3H), 3.94 (s, 6H), 3.93 (s, 3H), 2.72 (s, 3H). ^13 ^C NMR (151 MHz, CDCl_3_) δ 174.6, 166.6, 165.0, 161.8, 161.2, 159.0, 156.7, 154.4, 153.6 (2 C), 141.1, 141.1, 138.3, 131.5, 130.8, 128.9, 125.9, 124.4, 122.9, 108.5, 106.0 (2 C), 96.5, 92.7, 73.4, 61.0, 56.5, 56.5 (2 C), 55.9, 21.9. HRMS (ESI): *m/z* [M + H]^+^ calcd for C_31_H_30_N_3_O_10_: 604.1926; found: 604.1923.

*2–(5,7-dimethoxy-4-oxo-2–(3,4,5-trimethoxyphenyl)-4H-chromen-3-yloxy)-N-(5–(3-nitrophenyl)-1,3,4-oxadiazol-2-yl)acetamide* **(A14)**. Light yellow solid, 42.89% yield, m.p.: 233–235 °C; ^1^H NMR (600 MHz, CDCl_3_) δ 13.01 (s, 1H), 8.89 (s, 1H), 8.44 (d, *J* = 7.8 Hz, 1H), 8.36 (dd, *J* = 8.2, 1.1 Hz, 1H), 7.70 (t, *J* = 8.0 Hz, 1H), 7.25 (s, 2H), 6.57 (d, *J* = 2.0 Hz, 1H), 6.43 (d, *J* = 1.8 Hz, 1H), 4.41 (s, 2H), 4.02 (s, 3H), 3.97–3.92 (m, 12H). ^13 ^C NMR (151 MHz, CDCl_3_) δ 174.8, 166.7, 165.2, 161.2, 159.4, 159.1, 157.6, 154.6, 153.6 (2 C), 148.6, 141.2, 141.1, 132.2, 130.2, 125.7, 125.5, 124.3, 121.5, 108.4, 105.9 (2 C), 96.5, 92.8, 73.5, 61.0, 56.5(2 C), 55.9. HRMS (ESI): *m/z* [M + H]^+^ calcd for C_30_H_27_N_4_O_12_: 635.1620; found: 635.1620.

*2–(5,7-dimethoxy-4-oxo-2–(3,4,5-trimethoxyphenyl)-4H-chromen-3-yloxy)-N-(5–(2-nitrophenyl)-1,3,4-oxadiazol-2-yl)acetamide* **(A15)**. Light yellow solid, 45.03% yield, m.p.: 204–206 °C; ^1^H NMR (600 MHz, CDCl_3_) δ 12.99 (s, 1H), 8.08 (dd, *J* = 8.0, 1.2 Hz, 1H), 7.98 (dd, *J* = 7.5, 1.5 Hz, 1H), 7.77 (td, *J* = 7.6, 1.3 Hz, 1H), 7.73 (td, *J* = 7.8, 1.5 Hz, 1H), 7.24 (s, 2H), 6.56 (d, *J* = 2.2 Hz, 1H), 6.41 (d, *J* = 2.2 Hz, 1H), 4.38 (s, 2H), 3.99 (s, 3H), 3.95 (s, 3H), 3.95 (s, 6H), 3.93 (s, 3H). HRMS (ESI): *m/z* [M + H]^+^ calcd for C_30_H_27_N_4_O_12_: 635.1620; found: 635.1619.

*2–(5,7-dimethoxy-4-oxo-2–(3,4,5-trimethoxyphenyl)-4H-chromen-3-yloxy)-N-(5–(4-(trifluoromethyl)phenyl)-1,3,4-oxadiazol-2-yl)acetamide* **(A16)**. White solid, 51.73% yield, m.p.: 231–233 °C; ^1^H NMR (600 MHz, CDCl_3_) δ 12.78 (s, 1H), 8.22 (d, *J* = 8.2 Hz, 2H), 7.75 (d, *J* = 8.3 Hz, 2H), 7.25 (s, 2H), 6.57 (s, 1H), 6.43 (d, *J* = 1.7 Hz, 1H), 4.41 (s, 2H), 4.01 (s, 3H), 3.96 (s, 3H), 3.95 (s, 6H), 3.94 (s, 3H). ^13 ^C NMR (151 MHz, CDCl_3_) δ 174.7, 166.6, 165.1, 161.1, 160.3, 159.1, 157.4, 154.5, 153.6(2 C), 141.3, 141.1, 132.94 (m),127.1, 126.9 (2 C), 125.9 (d, *J* = 3.6 Hz) (2 C), 124.4, 123.6, 108.5, 106.1 (2 C), 96.5, 92.8, 73.4, 61.0, 56.5 (3 C), 55.9. HRMS (ESI): *m/z* [M + H]^+^ calcd for C_31_H_27_F_3_N_3_O_10_: 658.1643; found: 658.1641.

*2–(5,7-dimethoxy-4-oxo-2–(3,4,5-trimethoxyphenyl)-4H-chromen-3-yloxy)-N-(5–(3-(trifluoromethyl)phenyl)-1,3,4-oxadiazol-2-yl)acetamide* **(A17)**. White solid, 54.83% yield, m.p.: 220–222 °C; ^1^H NMR (600 MHz, CDCl_3_) δ 12.87 (s, 1H), 8.33 (s, 1H), 8.28 (d, *J* = 7.7 Hz, 1H), 7.76 (d, *J* = 7.6 Hz, 1H), 7.63 (t, *J* = 7.7 Hz, 1H), 7.25 (s, 2H), 6.56 (d, *J* = 1.6 Hz, 1H), 6.42 (d, *J* = 1.6 Hz, 1H), 4.40 (s, 2H), 3.99 (s, 3H), 3.98–3.89 (m, 12H). ^13 ^C NMR (151 MHz, CDCl_3_) δ 174.8, 166.7, 165.1, 161.1, 160.2, 159.1, 157.3, 154.5, 153.6 (2 C), 141.2, 141.1, 131.6, 129.8, 129.6, 127.8, 124.7, 124.3, 123.6, 123.5, 108.4, 106.0 (2 C), 96.5, 92.8, 73.4, 61.0, 56.5 (3 C), 55.9. HRMS (ESI): *m/z* [M + H]^+^ calcd for C_31_H_27_F_3_N_3_O_10_: 658.1643; found: 658.1642.

*2–(5,7-dimethoxy-4-oxo-2–(3,4,5-trimethoxyphenyl)-4H-chromen-3-yloxy)-N-(5–(2-(trifluoromethyl)phenyl)-1,3,4-oxadiazol-2-yl)acetamide* **(A18)**. White solid, 49.66% yield, m.p.: 191–193 °C; ^1^H NMR (600 MHz, CDCl_3_) δ 12.90 (s, 1H), 8.04 (d, *J* = 7.2 Hz, 1H), 7.85 (d, *J* = 7.3 Hz, 1H), 7.72–7.64 (m, 2H), 7.24 (s, 2H), 6.55 (d, *J* = 1.7 Hz, 1H), 6.41 (d, *J* = 1.4 Hz, 1H), 4.40 (s, 2H), 3.97 (s, 3H), 3.96–3.91 (m, 12H). ^13 ^C NMR (151 MHz, CDCl_3_) δ 174.6, 166.5, 165.1, 161.3, 159.4, 159.0, 157.9, 154.4, 153.6 (2 C), 141.2, 141.1, 131.9, 131.9, 131.2, 129.0, 126.8, 124.4, 123.2, 122.5, 108.5, 106.1 (2 C), 96.5, 92.7, 73.4, 60.9, 56.5 (2 C), 56.4, 55.9. HRMS (ESI): *m/z* [M + H]^+^ calcd for C_31_H_27_F_3_N_3_O_10_: 658.1643; found: 658.1641.

*2–(5,7-dimethoxy-4-oxo-2–(3,4,5-trimethoxyphenyl)-4H-chromen-3-yloxy)-N-(5–(4-(trifluoromethoxy)phenyl)-1,3,4-oxadiazol-2-yl)acetamide* **(A19)**. White solid, 51.51% yield, m.p.: 230–232 °C; ^1^H NMR (600 MHz, CDCl_3_) δ 12.62 (s, 1H), 8.13 (d, *J* = 8.6 Hz, 2H), 7.33 (d, *J* = 8.4 Hz, 2H), 7.25 (s, 2H), 6.56 (d, *J* = 1.4 Hz, 1H), 6.42 (d, *J* = 1.2 Hz, 1H), 4.41 (s, 2H), 3.99 (s, 3H), 3.96–3.92 (m, 12H). ^13 ^C NMR (151 MHz, CDCl_3_) δ 174.6, 166.6, 165.1, 161.2, 160.4, 159.1, 157.1, 154.4, 153.6 (2 C), 151.3 (d, *J* = 1.5 Hz), 141.3, 141.1, 128.4 (2 C), 124.4, 122.5, 121.1 (2 C), 120.3, 108.5, 106.1 (2 C), 96.5, 92.8, 73.3, 61.0, 56.6 (2 C), 56.5, 55.9. HRMS (ESI): *m/z* [M + H]^+^ calcd for C_31_H_27_F_3_N_3_O_11_: 674.1592; found: 674.1590.

*2–(5,7-dimethoxy-4-oxo-2–(3,4,5-trimethoxyphenyl)-4H-chromen-3-yloxy)-N-(5–(3-(trifluoromethoxy)phenyl)-1,3,4-oxadiazol-2-yl)acetamide* **(A20)**. White solid, 46.65% yield, m.p.: 221–223 °C; ^1^H NMR (600 MHz, CDCl_3_) δ 12.73 (s, 1H), 8.04 (d, *J* = 7.7 Hz, 1H), 7.94 (s, 1H), 7.53 (t, *J* = 8.0 Hz, 1H), 7.37 (d, *J* = 7.4 Hz, 1H), 7.25 (s, 2H), 6.57 (d, *J* = 1.9 Hz, 1H), 6.43 (d, *J* = 1.7 Hz, 1H), 4.41 (s, 2H), 4.00 (s, 3H), 3.98–3.92 (m, 12H). ^13 ^C NMR (151 MHz, CDCl_3_) δ 174.7, 166.6, 165.1, 161.2, 160.2, 159.1, 157.2, 154.4, 153.6 (2 C), 149.6, 141.3, 141.1, 130.5, 125.8, 124.9, 124.4, 123.6, 120.4, 119.2, 108.5, 106.1 (2 C), 96.5, 92.8, 73.4, 61.0, 56.6 (2 C), 56.5, 55.9. HRMS (ESI): *m/z* [M + H]^+^ calcd for C_31_H_27_F_3_N_3_O_11_: 674.1592; found: 674.1593.

*2–(5,7-dimethoxy-4-oxo-2–(3,4,5-trimethoxyphenyl)-4H-chromen-3-yloxy)-N-(5–(2-(trifluoromethoxy)phenyl)-1,3,4-oxadiazol-2-yl)acetamide* **(A21)**. White solid, 35.65% yield, m.p.: 202–204 °C; ^1^H NMR (600 MHz, CDCl_3_) δ 12.75 (s, 1H), 8.16 (dd, *J* = 7.7, 1.1 Hz, 1H), 7.59–7.54 (m, 1H), 7.44 (t, *J* = 7.7 Hz, 2H), 7.25 (s, 2H), 6.55 (d, *J* = 2.0 Hz, 1H), 6.41 (d, *J* = 1.9 Hz, 1H), 4.41 (s, 2H), 3.98 (s, 3H), 3.97–3.90 (m, 12H). ^13 ^C NMR (151 MHz, CDCl_3_) δ 174.6, 166.5, 165.0, 161.3, 159.0, 158.4, 157.5, 154.3, 153.6 (2 C), 146.4 (d, *J* = 1.7 Hz),141.2, 141.1, 132.5, 130.5, 127.2, 124.4, 122.1, 120.5, 118.2, 108.6, 106.1 (2 C), 96.5, 92.7, 73.4, 61.0, 56.5 (2 C), 56.4, 55.9. HRMS (ESI): *m/z* [M + H]^+^ calcd for C_31_H_27_F_3_N_3_O_11_: 674.1592; found: 674.1592.

*2–(5,7-dimethoxy-4-oxo-2–(3,4,5-trimethoxyphenyl)-4H-chromen-3-yloxy)-N-(5–(4-methoxyphenyl)-1,3,4-oxadiazol-2-yl)acetamide* **(A22)**. White solid, 38.43% yield, m.p.: 239–241 °C; ^1^H NMR (600 MHz, CDCl_3_) δ 12.41 (s, 1H), 8.01 (d, *J* = 8.5 Hz, 2H), 7.25 (s, 2H), 6.98 (d, *J* = 8.6 Hz, 2H), 6.56 (d, *J* = 1.5 Hz, 1H), 6.42 (d, *J* = 1.3 Hz, 1H), 4.40 (s, 2H), 4.00 (s, 3H), 3.98–3.92 (m, 12H), 3.87 (s, 3H). ^13 ^C NMR (151 MHz, CDCl_3_) δ 174.6, 166.6, 165.0, 162.1, 161.6, 161.1, 159.1, 156.4, 154.32, 153.6 (2 C), 141.1, 141.1, 128.5 (2 C), 124.5, 116.4, 114.3 (2 C), 108.5, 105.9 (2 C), 96.5, 92.8, 73.3, 61.1, 56.6, 56.5(2 C), 55.9, 55.4. HRMS (ESI): *m/z* [M + H]^+^ calcd for C_31_H_30_N_3_O_11_: 620.1875; found: 620.1871.

*2–(5,7-dimethoxy-4-oxo-2–(3,4,5-trimethoxyphenyl)-4H-chromen-3-yloxy)-N-(5–(3-methoxyphenyl)-1,3,4-oxadiazol-2-yl)acetamide* **(A23)**. White solid, 43.92% yield, m.p.: 208–210 °C; ^1^H NMR (400 MHz, CDCl_3_) δ 12.63 (s, 1H), 7.68–7.64 (m, 1H), 7.63–7.61 (m, 1H), 7.39 (t, *J* = 8.0 Hz, 1H), 7.25 (s, 2H), 7.07–7.03 (m, 1H), 6.57 (d, *J* = 2.2 Hz, 1H), 6.42 (d, *J* = 2.2 Hz, 1H), 4.40 (s, 2H), 4.00 (s, 3H), 3.96 (s, 3H), 3.95 (s, 6H), 3.93 (s, 3H), 3.88 (s, 3H). ^13 ^C NMR (151 MHz, CDCl_3_) δ 174.6, 166.6, 165.0, 161.5, 161.1, 159.9, 159.1, 156.9, 154.4, 153.6 (2 C), 141.1, 141.1, 129.9, 124.9, 124.4, 119.2, 118.1, 111.2, 108.5, 105.9 (2 C), 96.5, 92.8, 73.4, 61.1, 55.5, 56.6, 56.5 (2 C), 55.9. HRMS (ESI): *m/z* [M + H]^+^ calcd for C_31_H_30_N_3_O_11_: 620.1875; found: 620.1873.

*N-(5–(3-chloro-4-fluorophenyl)-1,3,4-oxadiazol-2-yl)-2–(5,7-dimethoxy-4-oxo-2–(3,4,5-trimethoxyphenyl)-4H-chromen-3-yloxy)acetamide* **(A24)**. White solid, 31.79% yield, m.p.: 228–230 °C; ^1^H NMR (600 MHz, CDCl_3_) δ 12.79 (s, 1H), 8.16 (d, *J* = 6.7 Hz, 1H), 8.03–7.98 (m, 1H), 7.28 (d, *J* = 9.2 Hz, 1H), 7.26 (s, 2H), 6.57 (s, 1H), 6.44 (s, 1H), 4.41 (s, 2H), 4.02 (s, 3H), 3.98–3.93 (m, 12H). ^13 ^C NMR (151 MHz, CDCl_3_) δ 174.7, 166.6, 165.1, 161.2, 160.2, 159.1, 159.1, 157.2, 154.5, 153.6 (2 C), 141.3, 141.1, 129.1, 126.8, 124.34, 122.2, 121.2, 117.3, 108.5, 106.1 (2 C), 96.5, 92.8, 73.4, 61.0, 56.6 (3 C), 55.9. HRMS (ESI): *m/z* [M + H]^+^ calcd for C_30_H_26_ClFN_3_O_10_: 642.1285; found: 642.1281.

*N-(5–(3,4-dichlorophenyl)-1,3,4-oxadiazol-2-yl)-2–(5,7-dimethoxy-4-oxo-2–(3,4,5-trimethoxyphenyl)-4H-chromen-3-yloxy)acetamide* **(A25)**. White solid, 36.16% yield, m.p.: 222–224 °C; ^1^H NMR (600 MHz, CDCl_3_) δ 12.87 (s, 1H), 8.18 (d, *J* = 1.8 Hz, 1H), 7.94 (dd, *J* = 8.4, 1.9 Hz, 1H), 7.58 (d, *J* = 8.4 Hz, 1H), 7.25 (s, 2H), 6.57 (d, *J* = 2.0 Hz, 1H), 6.43 (d, *J* = 1.9 Hz, 1H), 4.40 (s, 2H), 4.02 (s, 3H), 3.98–3.92 (m, 12H). ^13 ^C NMR (151 MHz, CDCl_3_) δ 174.8, 166.6, 165.1, 161.1, 159.6, 159.1, 157.3, 154.5, 153.6 (2 C), 141.2, 141.1, 135.8, 133.5, 131.1, 128.3, 125.7, 124.3, 123.7, 108.4, 106.0 (2 C), 96.6, 92.8, 73.4, 61.0, 56.6, 56.5 (2 C), 55.9. HRMS (ESI): *m/z* [M + H]^+^ calcd for C_30_H_26_Cl_2_N_3_O_10_: 658.0990; found: 658.0986.

*2–(5,7-dimethoxy-4-oxo-2–(3,4,5-trimethoxyphenyl)-4H-chromen-3-yloxy)-N-(5–(3,5-dimethoxyphenyl)-1,3,4-oxadiazol-2-yl)acetamide* **(A26)**. White solid, 47.13% yield, m.p.: 198–200 °C; ^1^H NMR (600 MHz, CDCl_3_) δ 12.47 (s, 1H), 7.25 (s, 2H), 7.23 (d, *J* = 2.2 Hz, 2H), 6.59 (t, *J* = 2.2 Hz, 1H), 6.56 (d, *J* = 2.1 Hz, 1H), 6.42 (d, *J* = 2.1 Hz, 1H), 4.41 (s, 2H), 4.00 (s, 3H), 3.96 (s, 3H), 3.95 (s, 6H), 3.94 (s, 3H), 3.86 (s, 6H). ^13 ^C NMR (151 MHz, CDCl_3_) δ 174.6, 166.6, 165.0, 161.5, 161.1, 161.0 (2 C), 159.1, 156.8, 154.4, 153.6 (2 C), 141.1, 141.0, 125.4, 124.4, 108.5, 105.9 (2 C), 104.5(2 C), 104.3, 96.5, 92.8, 73.3, 61.1, 56.6, 56.5 (2 C), 55.9, 55.7 (2 C). HRMS (ESI): *m/z* [M + H]^+^ calcd for C_32_H_32_N_3_O_12_: 650.1980; found: 650.1976.

*2–(5,7-dimethoxy-4-oxo-2–(3,4,5-trimethoxyphenyl)-4H-chromen-3-yloxy)-N-(5–(3,4-dimethoxyphenyl)-1,3,4-oxadiazol-2-yl)acetamide* **(A27)**. White solid, 41.89% yield, m.p.: 220–222 °C; ^1^H NMR (600 MHz, CDCl_3_) δ 12.36 (s, 1H), 7.64 (d, *J* = 8.3 Hz, 1H), 7.60 (s, 1H), 7.25 (s, 2H), 6.94 (d, *J* = 8.4 Hz, 1H), 6.56 (d, *J* = 1.5 Hz, 1H), 6.42 (d, *J* = 1.4 Hz, 1H), 4.41 (s, 2H), 3.99 (s, 3H), 3.98–3.91 (m, 18H). ^13 ^C NMR (151 MHz, CDCl_3_) δ 174.5, 166.6, 165.0, 161.7, 161.2, 159.0, 156.5, 154.3, 153.6 (2 C), 151.8, 149.3, 141.2, 141.0, 124.4, 120.3, 116.6, 111.1, 109.5, 108.6, 106.1 (2 C), 96.5, 92.8, 73.3, 61.0, 56.6 (2 C), 56.5, 56.2, 55.9, 55.9. HRMS (ESI): *m/z* [M + H]^+^ calcd for C_32_H_32_N_3_O_12_: 650.1980; found: 650.1978.

*2–(5,7-dimethoxy-4-oxo-2–(3,4,5-trimethoxyphenyl)-4H-chromen-3-yloxy)-N-(5-(naphthalen-1-yl)-1,3,4-oxadiazol-2-yl)acetamide* **(A28)**. White solid, 53.18% yield, m.p.: 238–240 °C; ^1^H NMR (600 MHz, CDCl_3_) δ 12.59 (s, 1H), 9.27 (d, *J* = 8.6 Hz, 1H), 8.24 (d, *J* = 7.1 Hz, 1H), 8.01 (d, *J* = 8.1 Hz, 1H), 7.91 (d, *J* = 8.1 Hz, 1H), 7.68 (t, *J* = 7.6 Hz, 1H), 7.57 (dd, *J* = 17.0, 8.2 Hz, 2H), 7.27 (s, 2H), 6.56 (s, 1H), 6.42 (s, 1H), 4.45 (s, 2H), 4.00 (s, 3H), 3.98–3.92 (m, 12H). ^13 ^C NMR (151 MHz, CDCl_3_) δ 174.5, 166.6, 165.0, 161.6, 161.2, 159.1, 156.7, 154.3, 153.6 (2 C), 141.3, 141.1, 133.8, 132.1, 130.0, 128.5, 128.3, 127.9, 126.5, 126.4, 124.8, 124.5, 120.4, 108.6, 106.2 (2 C), 96.5, 92.8, 73.4, 61.0, 56.6 (2 C), 56.5, 55.9. HRMS (ESI): *m/z* [M + H]^+^ calcd for C_34_H_30_N_3_O_10_: 640.1926; found: 640.1924.

*N-(5–(2,3-dihydrobenzo[b][1, 4]dioxin-6-yl)-1,3,4-oxadiazol-2-yl)-2–(5,7-dimethoxy-4-oxo-2–(3,4,5-trimethoxyphenyl)-4H-chromen-3-yloxy)acetamide* **(A29)**. White solid, 54.62% yield, m.p.: 231–233 °C; ^1^H NMR (400 MHz, CDCl_3_) δ 12.51 (s, 1H), 7.60 (d, *J* = 2.0 Hz, 1H), 7.57 (dd, *J* = 8.4, 2.1 Hz, 1H), 7.24 (s, 2H), 6.93 (d, *J* = 8.4 Hz, 1H), 6.56 (d, *J* = 2.2 Hz, 1H), 6.41 (d, *J* = 2.2 Hz, 1H), 4.39 (s, 2H), 4.30 (q, *J* = 5.1 Hz, 4H), 4.00 (s, 3H), 3.95 (s, 3H), 3.94 (s, 6H), 3.93 (s, 3H). ^13 ^C NMR (151 MHz, CDCl_3_) δ 174.6, 166.6, 165.0, 161.3, 161.1, 159.1, 156.5, 154.3, 153.5 (2 C), 146.4, 143.8, 141.1, 141.0, 124.5, 120.4, 117.8, 117.1, 115.9, 108.5, 105.9 (2 C), 96.5, 92.7, 73.4, 64.6, 64.2, 61.1, 56.6, 56.5 (2 C), 55.9. HRMS (ESI): *m/z* [M + H]^+^ calcd for C_32_H_30_N_3_O_12_: 648.1824; found: 648.1821.

*N-(5–(3-(benzyloxy)phenyl)-1,3,4-oxadiazol-2-yl)-2–(5,7-dimethoxy-4-oxo-2–(3,4,5-trimethoxyphenyl)-4H-chromen-3-yloxy)acetamide* **(A30)**. White solid, 44.01% yield, m.p.: 188–190 °C; ^1^H NMR (600 MHz, CDCl_3_) δ 12.54 (s, 1H), 7.73 (s, 1H), 7.68 (d, *J* = 7.6 Hz, 1H), 7.46 (d, *J* = 7.3 Hz, 2H), 7.39 (t, *J* = 7.7 Hz, 3H), 7.33 (t, *J* = 7.2 Hz, 1H), 7.26 (s, 2H), 7.15–7.10 (m, 1H), 6.56 (d, *J* = 1.7 Hz, 1H), 6.42 (d, *J* = 1.6 Hz, 1H), 5.14 (s, 2H), 4.41 (s, 2H), 3.99 (s, 3H), 3.98–3.91 (m, 12H). ^13 ^C NMR (151 MHz, CDCl_3_) δ 174.6, 166.6, 165.1, 161.4, 161.1, 159.1, 156.9, 154.4, 153.6, 141.1, 141.0, 136.5, 130.1, 128.6, 128.1, 127.6, 125.0, 124.4, 119.44, 118.6, 112.4, 108.5, 106.0, 96.5, 92.7, 73.4, 70.3, 61.0, 56.5, 55.9. HRMS (ESI): *m/z* [M + H]^+^ calcd for C_37_H_34_N_3_O_11_: 696.2188; found: 696.2183.

*2–(5,7-dimethoxy-4-oxo-2–(3,4,5-trimethoxyphenyl)-4H-chromen-3-yloxy)-N-(5-(furan-2-yl)-1,3,4-oxadiazol-2-yl)acetamide* **(A31)**. White solid, 49.30% yield, m.p.: 240–242 °C; ^1^H NMR (600 MHz, CDCl_3_) δ 12.64 (s, 1H), 7.61 (d, *J* = 0.9 Hz, 1H), 7.24 (s, 2H), 7.16 (d, *J* = 3.2 Hz, 1H), 6.57 (dd, *J* = 3.4, 1.7 Hz, 1H), 6.56 (d, *J* = 2.1 Hz, 1H), 6.42 (d, *J* = 1.9 Hz, 1H), 4.39 (s, 2H), 4.00 (s, 3H), 3.97–3.92 (m, 12H). ^13 ^C NMR (151 MHz, CDCl_3_) δ 174.6, 166.6, 165.0, 161.2, 159.1, 156.3, 154.4, 154.4, 153.6 (2 C), 145.3, 141.2, 141.1, 139.3, 124.4, 113.5, 111.9, 108.5, 106.1 (2 C), 96.5, 92.8, 73.3, 61.0, 56.5 (3 C), 55.9. HRMS (ESI): *m/z* [M + H]^+^ calcd for C_28_H_26_N_3_O_11_: 580.1562; found: 580.1562.

*2–(5,7-dimethoxy-4-oxo-2–(3,4,5-trimethoxyphenyl)-4H-chromen-3-yloxy)-N-(5-(thiophen-2-yl)-1,3,4-oxadiazol-2-yl)acetamide* **(A32)**. White solid, 45.68% yield, m.p.: 224–226 °C; ^1^H NMR (400 MHz, CDCl_3_) δ 12.60 (s, 1H), 7.78 (dd, *J* = 3.7, 1.1 Hz, 1H), 7.52 (dd, *J* = 5.0, 1.0 Hz, 1H), 7.25 (s, 2H), 7.15 (dd, *J* = 5.0, 3.8 Hz, 1H), 6.57 (d, *J* = 2.2 Hz, 1H), 6.42 (d, *J* = 2.2 Hz, 1H), 4.39 (s, 2H), 4.00 (s, 3H), 3.96 (s, 3H), 3.94 (s, 6H), 3.94 (s, 3H). ^13 ^C NMR (151 MHz, CDCl_3_) δ 174.6, 166.6, 165.1, 161.1, 159.0, 157.9, 156.3, 154.4, 153.6 (2 C), 141.2, 141.0, 129.6, 129.5, 127.9, 125.2, 124.4, 108.5, 106.1 (2 C), 96.5, 92.8, 73.3, 61.0, 56.5 (3 C), 55.9. HRMS (ESI): *m/z* [M + H]^+^ calcd for C_28_H_26_N_3_O_10_S: 596.1333; found: 596.1330.

*(E)-2–(5,7-dimethoxy-4-oxo-2–(3,4,5-trimethoxyphenyl)-4H-chromen-3-yloxy)-N-(5-styryl-1,3,4-oxadiazol-2-yl)acetamide* **(A33)**. White solid, 45.31% yield, m.p.: 223–225 °C; ^1^H NMR (600 MHz, CDCl_3_) δ 12.59 (s, 1H), 7.57 (d, *J* = 16.7 Hz, 1H), 7.54 (d, *J* = 7.4 Hz, 2H), 7.43–7.34 (m, 3H), 7.25 (s, 2H), 7.02 (d, *J* = 16.4 Hz, 1H), 6.57 (d, *J* = 2.0 Hz, 1H), 6.43 (d, *J* = 1.9 Hz, 1H), 4.40 (s, 2H), 4.01 (s, 3H), 3.98 − 3.92 (m, 12H). ^13 ^C NMR (151 MHz, CDCl_3_) δ 174.7, 166.5, 165.1, 161.2, 161.1, 159.1, 156.4, 154.4, 153.6 (2 C), 141.1, 141.2, 138.2, 134.8, 129.7, 128.9 (2 C), 127.4 (2 C), 124.4, 109.9, 108.5, 105.9 (2 C), 96.5, 92.8, 73.4, 61.0, 56.6, 56.6 (2 C), 55.9. HRMS (ESI): *m/z* [M + H]^+^ calcd for C_32_H_30_N_3_O_10_: 616.1926; found: 616.1924.

*2–(2-(3,4-dimethoxyphenyl)-5,7-dimethoxy-4-oxo-4H-chromen-3-yloxy)-N-(5-phenyl-1,3,4-oxadiazol-2-yl)acetamide* **(B1)**. White solid, 46.92% yield, m.p.: 237–239 °C; ^1^H NMR (600 MHz, CDCl_3_) δ 12.57 (s, 1H), 8.09 (d, *J* = 6.9 Hz, 2H), 7.67 (d, *J* = 8.5 Hz, 1H), 7.55 (s, 1H), 7.53–7.46 (m, 3H), 7.01 (d, *J* = 8.5 Hz, 1H), 6.56 (s, 1H), 6.41 (s, 1H), 4.40 (s, 2H), 3.99 (s, 3H), 3.98 (s, 3H), 3.97 (s, 3H), 3.93 (s, 3H). ^13 ^C NMR (151 MHz, CDCl_3_) δ 174.7, 166.8, 164.9, 161.6, 161.2, 159.1, 156.9, 154.6, 151.9, 149.4, 140.8, 131.3, 128.9 (2 C), 126.8 (2 C), 123.9, 122.3, 121.9, 111.4, 111.1, 108.6, 96.5, 92.8, 73.3, 56.6, 56.3, 56.1, 55.9. HRMS (ESI): *m/z* [M + H]^+^ calcd for C_29_H_26_N_3_O_9_: 560.1664; found: 560.1665.

*2–(2-(3,4-dimethoxyphenyl)-5,7-dimethoxy-4-oxo-4H-chromen-3-yloxy)-N-(5–(4-fluorophenyl)-1,3,4-oxadiazol-2-yl)acetamide* **(B2)**. White solid, 54.11% yield, m.p.: 236–238 °C; ^1^H NMR (600 MHz, CDCl_3_) δ 12.61 (s, 1H), 8.12–8.06 (m, 2H), 7.67 (dd, *J* = 8.5, 2.0 Hz, 1H), 7.54 (d, *J* = 1.9 Hz, 1H), 7.17 (dd, *J* = 12.0, 5.3 Hz, 2H), 7.02 (d, *J* = 8.6 Hz, 1H), 6.57 (d, *J* = 2.2 Hz, 1H), 6.41 (d, *J* = 2.1 Hz, 1H), 4.40 (s, 2H), 4.01–3.96 (m, 9H), 3.93 (s, 3H). ^13 ^C NMR (151 MHz, CDCl_3_) δ 174.6, 166.7, 164.9, 164.6, 161.1, 160.67, 159.0, 156.9, 154.5, 151.9, 149.3, 140.8, 128.9 (2 C), 122.2, 121.8, 120.3, 116.2 (2 C), 111.3, 110.9, 108.5, 96.4, 92.8, 73.2, 56.5, 56.3, 56.1, 55.9. HRMS (ESI): *m/z* [M + H]^+^ calcd for C_29_H_25_FN_3_O_9_: 578.1569; found: 578.1569.

*2–(2-(3,4-dimethoxyphenyl)-5,7-dimethoxy-4-oxo-4H-chromen-3-yloxy)-N-(5–(3-fluorophenyl)-1,3,4-oxadiazol-2-yl)acetamide* **(B3)**. White solid, 34.63% yield, m.p.: 229–231 °C; ^1^H NMR (600 MHz, CDCl_3_) δ 12.77 (s, 1H), 7.89 (d, *J* = 6.8 Hz, 1H), 7.79 (d, *J* = 8.2 Hz, 1H), 7.68 (d, *J* = 8.0 Hz, 1H), 7.55 (s, 1H), 7.47 (d, *J* = 6.2 Hz, 1H), 7.21 (d, *J* = 7.2 Hz, 1H), 7.02 (d, *J* = 7.8 Hz, 1H), 6.57 (s, 1H), 6.42 (s, 1H), 4.40 (s, 2H), 4.04–3.90 (m, 12H). HRMS (ESI): *m/z* [M + H]^+^ calcd for C_29_H_25_FN_3_O_9_: 578.1569; found: 578.1568.

*2–(2-(3,4-dimethoxyphenyl)-5,7-dimethoxy-4-oxo-4H-chromen-3-yloxy)-N-(5–(2-fluorophenyl)-1,3,4-oxadiazol-2-yl)acetamide* **(B4)**. White solid, 38.97% yield, m.p.: 235–237 °C; ^1^H NMR (600 MHz, CDCl_3_) δ 12.70 (s, 1H), 8.07 (t, *J* = 7.0 Hz, 1H), 7.67 (d, *J* = 8.2 Hz, 1H), 7.54 (s, 1H), 7.50 (q, *J* = 11.4, 6.7 Hz, 1H), 7.28 (d, *J* = 7.6 Hz, 1H), 7.22 (d, *J* = 9.3 Hz, 1H), 7.01 (d, *J* = 8.5 Hz, 1H), 6.56 (s, 1H), 6.41 (d, *J* = 0.8 Hz, 1H), 4.40 (s, 2H), 3.99 (s, 3H), 3.98 (s, 3H), 3.97 (s, 3H), 3.92 (s, 3H). ^13 ^C NMR (151 MHz, CDCl_3_) δ 174.6, 166.7, 164.9, 161.2, 159.9 (d, *J* = 258.7 Hz), 159.0, 158.2, 157.2, 154.5, 151.9, 149.3, 140.7, 132.9, 129.7, 124.4, 122.2, 121.9, 116.8, 112.6, 111.4, 111.1, 108.6, 96.4, 92.7, 73.2, 56.5, 56.3, 56.1, 55.9. HRMS (ESI): *m/z* [M + H]^+^ calcd for C_29_H_25_FN_3_O_9_: 578.1569; found: 578.1567.

*N-(5–(4-chlorophenyl)-1,3,4-oxadiazol-2-yl)-2–(2-(3,4-dimethoxyphenyl)-5,7-dimethoxy-4-oxo-4H-chromen-3-yloxy)acetamide* **(B5)**. White solid, 42.10% yield, m.p.: 213–215 °C; ^1^H NMR (600 MHz, CDCl_3_) δ 12.75 (s, 1H), 8.01 (d, *J* = 8.1 Hz, 2H), 7.67 (d, *J* = 8.4 Hz, 1H), 7.54 (s, 1H), 7.46 (d, *J* = 8.2 Hz, 2H), 7.01 (d, *J* = 8.5 Hz, 1H), 6.56 (s, 1H), 6.40 (s, 1H), 4.39 (s, 2H), 4.03–3.95 (m, 9H), 3.92 (s, 3H). ^13^C NMR (151 MHz, CDCl_3_) δ 174.7, 166.7, 164.9, 161.1, 160.6, 159.0, 157.1, 154.6, 151.9, 149.3, 140.7, 137.5, 129.2 (2 C), 127.9 (2 C), 122.4, 122.2, 121.8, 111.3, 110.9, 108.4, 96.4, 92.7, 73.2, 56.5, 56.2, 56.1, 55.9. HRMS (ESI): *m/z* [M + H]^+^ calcd for C_29_H_25_ClN_3_O_9_: 594.1274; found: 594.1272.

*N-(5–(3-chlorophenyl)-1,3,4-oxadiazol-2-yl)-2–(2-(3,4-dimethoxyphenyl)-5,7-dimethoxy-4-oxo-4H-chromen-3-yloxy)acetamide* **(B6)**. White solid, 45.24% yield, m.p.: 220–222 °C; ^1^H NMR (600 MHz, CDCl_3_) δ 12.81 (s, 1H), 8.08 (s, 1H), 7.99 (d, *J* = 7.7 Hz, 1H), 7.68 (dd, *J* = 8.5, 1.8 Hz, 1H), 7.54 (d, *J* = 1.7 Hz, 1H), 7.48 (d, *J* = 8.1 Hz, 1H), 7.43 (t, *J* = 7.9 Hz, 1H), 7.02 (d, *J* = 8.6 Hz, 1H), 6.57 (d, *J* = 2.0 Hz, 1H), 6.41 (d, *J* = 1.9 Hz, 1H), 4.40 (s, 2H), 4.00 (s, 3H), 3.98 (s, 3H), 3.97 (s, 3H), 3.93 (s, 3H). ^13 ^C NMR (151 MHz, CDCl_3_) δ 174.7, 166.7, 164.9, 161.1, 160.2, 159.0, 157.2, 154.6, 151.9, 149.3, 140.7, 135.0, 131.3, 130.2, 126.6, 125.5, 124.7, 122.2, 121.8, 111.4, 111.1, 108.4, 96.4, 92.8, 73.2, 56.5, 56.3, 56.1, 55.9. HRMS (ESI): *m/z* [M + H]^+^ calcd for C_29_H_25_ClN_3_O_9_: 594.1274; found: 594.1271.

*N-(5–(2-chlorophenyl)-1,3,4-oxadiazol-2-yl)-2–(2-(3,4-dimethoxyphenyl)-5,7-dimethoxy-4-oxo-4H-chromen-3-yloxy)acetamide* **(B7)**. White solid, 31.57% yield, m.p.: 221–223 °C; ^1^H NMR (600 MHz, CDCl_3_) δ 12.73 (s, 1H), 8.00 (dd, *J* = 7.7, 1.4 Hz, 1H), 7.67 (dd, *J* = 8.5, 1.9 Hz, 1H), 7.56 − 7.52 (m, 2H), 7.44 (td, *J* = 7.8, 1.5 Hz, 1H), 7.38 (td, *J* = 7.6, 0.8 Hz, 1H), 7.02 (d, *J* = 8.6 Hz, 1H), 6.56 (d, *J* = 2.0 Hz, 1H), 6.41 (d, *J* = 2.0 Hz, 1H), 4.40 (s, 2H), 3.99–3.96 (m, 9H), 3.93 (s, 3H). ^13 ^C NMR (151 MHz, CDCl_3_) δ 174.7, 166.7, 164.9, 161.1, 159.6, 159.0, 157.3, 154.5, 151.8, 149.3, 140.8, 133.2, 132.0, 131.2, 131.0, 126.9, 123.3, 122.2, 121.8, 111.3,110.9, 108.5, 96.4, 92.7, 73.3, 56.5, 56.3, 56.1, 55.9. HRMS (ESI): *m/z* [M + H]^+^ calcd for C_29_H_25_ClN_3_O_9_: 594.1274; found: 594.1270.

*N-(5–(4-bromophenyl)-1,3,4-oxadiazol-2-yl)-2–(2-(3,4-dimethoxyphenyl)-5,7-dimethoxy-4-oxo-4H-chromen-3-yloxy)acetamide* **(B8)**. White solid, 44.05% yield, m.p.: 212–214 °C; ^1^H NMR (600 MHz, CDCl_3_) δ 12.73 (s, 1H), 7.98–7.94 (m, 2H), 7.68 (dd, *J* = 8.5, 2.0 Hz, 1H), 7.65–7.61 (m, 2H), 7.54 (d, *J* = 2.0 Hz, 1H), 7.02 (d, *J* = 8.6 Hz, 1H), 6.57 (d, *J* = 2.1 Hz, 1H), 6.42 (d, *J* = 2.1 Hz, 1H), 4.39 (s, 2H), 4.00 (s, 3H), 3.98 (s, 3H), 3.97 (s, 3H), 3.93 (s, 3H). ^13 ^C NMR (151 MHz, CDCl_3_) δ 174.7, 166.7, 164.9, 161.1, 160.7, 159.0, 157.1, 154.6, 151.9, 149.3, 140.8, 132.2 (2 C), 128.1 (2 C), 125.9, 122.8, 122.2, 121.8, 111.3, 110.9, 108.5, 96.4, 92.8, 73.3, 56.5, 56.3, 56.1, 55.9. HRMS (ESI): *m/z* [M + H]^+^ calcd for C_29_H_25_BrN_3_O_9_: 638.0769; found: 638.0764.

*N-(5–(3-bromophenyl)-1,3,4-oxadiazol-2-yl)-2–(2-(3,4-dimethoxyphenyl)-5,7-dimethoxy-4-oxo-4H-chromen-3-yloxy)acetamide* **(B9)**. White solid, 39.16% yield, m.p.: 204–206 °C; ^1^H NMR (400 MHz, CDCl_3_) δ 12.87 (s, 1H), 8.24 (t, *J* = 1.7 Hz, 1H), 8.07–8.02 (m, 1H), 7.68 (dd, *J* = 8.5, 2.1 Hz, 1H), 7.65–7.62 (m, 1H), 7.55 (d, *J* = 2.0 Hz, 1H), 7.37 (t, *J* = 7.9 Hz, 1H), 7.02 (d, *J* = 8.6 Hz, 1H), 6.58 (d, *J* = 2.2 Hz, 1H), 6.42 (d, *J* = 2.2 Hz, 1H), 4.40 (s, 2H), 4.01 (s, 3H), 3.99 (s, 3H), 3.97 (s, 3H), 3.94 (s, 3H). ^13 ^C NMR (151 MHz, CDCl_3_) δ 174.7, 166.7, 164.9, 161.1, 160.0, 159.0, 157.2, 154.6, 151.9, 149.3, 140.8, 134.2, 130.4, 129.4, 125.7, 125.2, 122.9, 122.2, 121.8, 111.3, 110.9, 108.4, 96.4, 92.7, 73.3, 56.6, 56.3, 56.1, 55.9. HRMS (ESI): *m/z* [M + H]^+^ calcd for C_29_H_25_BrN_3_O_9_: 638.0769; found: 638.0765.

*N-(5–(2-bromophenyl)-1,3,4-oxadiazol-2-yl)-2–(2-(3,4-dimethoxyphenyl)-5,7-dimethoxy-4-oxo-4H-chromen-3-yloxy)acetamide* **(B10)**. White solid, 48.95% yield, m.p.: 226–228 °C; ^1^H NMR (600 MHz, CDCl_3_) δ 12.80 (s, 1H), 7.94 (d, *J* = 7.6 Hz, 1H), 7.73 (d, *J* = 8.0 Hz, 1H), 7.67 (d, *J* = 8.4 Hz, 1H), 7.54 (s, 1H), 7.43 (t, *J* = 7.5 Hz, 1H), 7.35 (t, *J* = 7.7 Hz, 1H), 7.01 (d, *J* = 8.5 Hz, 1H), 6.55 (s, 1H), 6.40 (s, 1H), 4.40 (s, 2H), 4.00–3.95 (m, 9H), 3.92 (s, 3H). ^13 ^C NMR (151 MHz, CDCl_3_) δ 174.6, 166.6, 164.9, 161.2, 160.2, 159.0, 157.4, 154.5, 151.9, 149.4, 140.7, 134.3, 132.1, 131.6, 127.3, 125.5, 122.2, 121.9, 121.7, 111.4, 111.1, 108.5, 96.4, 92.8, 73.24, 56.5, 56.3, 56.1, 55.9. HRMS (ESI): *m/z* [M + H]^+^ calcd for C_29_H_25_BrN_3_O_9_: 638.0769; found: 638.0766.

*2–(2-(3,4-dimethoxyphenyl)-5,7-dimethoxy-4-oxo-4H-chromen-3-yloxy)-N-(5-p-tolyl-1,3,4-oxadiazol-2-yl)acetamide* **(B11)**. White solid, 41.50% yield, m.p.: 239–241 °C; ^1^H NMR (600 MHz, CDCl_3_) δ 12.53 (s, 1H), 7.97 (d, *J* = 8.1 Hz, 2H), 7.67 (dd, *J* = 8.5, 1.9 Hz, 1H), 7.54 (d, *J* = 1.6 Hz, 1H), 7.28 (d, *J* = 7.9 Hz, 2H), 7.01 (d, *J* = 8.6 Hz, 1H), 6.56 (d, *J* = 2.0 Hz, 1H), 6.40 (d, *J* = 1.9 Hz, 1H), 4.39 (s, 2H), 3.99 (s, 3H), 3.98 (s, 3H), 3.96 (s, 3H), 3.92 (s, 3H), 2.41 (s, 3H). ^13 ^C NMR (151 MHz, CDCl_3_) δ 174.7, 166.7, 164.9, 161.6, 161.1, 159.0, 156.7, 154.5, 151.8, 149.3, 141.8, 140.7, 129.5 (2 C), 126.7 (2 C), 122.2, 121.8, 121.1, 111.3, 110.9, 108.5, 96.4, 92.7, 73.2, 56.5, 56.2, 56.1, 55.9, 21.6. HRMS (ESI): *m/z* [M + H]^+^ calcd for C_30_H_28_N_3_O_9_: 574.1820; found: 574.1817.

*2–(2-(3,4-dimethoxyphenyl)-5,7-dimethoxy-4-oxo-4H-chromen-3-yloxy)-N-(5-m-tolyl-1,3,4-oxadiazol-2-yl)acetamide* **(B12)**. White solid, 35.96% yield, m.p.: 221–223 °C; ^1^H NMR (600 MHz, CDCl_3_) δ 12.61 (s, 1H), 7.92 (s, 1H), 7.88 (d, *J* = 7.6 Hz, 1H), 7.68 (d, *J* = 7.9 Hz, 1H), 7.55 (s, 1H), 7.37 (t, *J* = 7.6 Hz, 1H), 7.31 (d, *J* = 7.4 Hz, 1H), 7.02 (d, *J* = 8.5 Hz, 1H), 6.57 (s, 1H), 6.41 (s, 1H), 4.40 (s, 2H), 4.02 − 3.96 (m, 9H), 3.93 (s, 3H), 2.42 (s, 3H). ^13 ^C NMR (151 MHz, CDCl_3_) δ 174.7, 166.7, 164.9, 161.6, 161.1, 159.0, 156.8, 154.5, 151.8, 149.3, 140.7, 138.7, 132.1, 128.7, 127.2, 123.9, 123.7, 122.2, 121.8, 111.3, 110.9, 108.5, 96.4, 92.7, 73.2, 56.6, 56.3, 56.1, 55.9, 21.3. HRMS (ESI): *m/z* [M + H]^+^ calcd for C_30_H_28_N_3_O_9_: 574.1820; found: 574.1816.

*2–(2-(3,4-dimethoxyphenyl)-5,7-dimethoxy-4-oxo-4H-chromen-3-yloxy)-N-(5-o-tolyl-1,3,4-oxadiazol-2-yl)acetamide* **(B13)**. White solid, 49.04% yield, m.p.: 234–236 °C; ^1^H NMR (600 MHz, CDCl_3_) δ 12.62 (s, 1H), 7.98 (d, *J* = 7.7 Hz, 1H), 7.68 (dd, *J* = 8.4, 1.3 Hz, 1H), 7.55 (s, 1H), 7.39 (t, *J* = 7.4 Hz, 1H), 7.31 (dd, *J* = 16.6, 8.0 Hz, 2H), 7.02 (d, *J* = 8.5 Hz, 1H), 6.56 (d, *J* = 1.6 Hz, 1H), 6.41 (d, *J* = 1.4 Hz, 1H), 4.40 (s, 2H), 4.01–3.96 (m, 9H), 3.93 (s, 3H), 2.73 (s, 3H). ^13 ^C NMR (151 MHz, CDCl_3_) δ 174.6, 166.7, 164.9, 161.7, 161.1, 159.0, 156.7, 154.5, 151.8, 149.3, 140.7, 138.3, 131.5, 130.8, 128.9, 125.9, 123.0, 122.2, 121.9, 111.3, 110.9, 108.5, 96.4, 92.7, 73.2, 56.5, 56.3, 56.1, 55.9, 21.9. HRMS (ESI): *m/z* [M + H]^+^ calcd for C_30_H_28_N_3_O_9_: 574.1820; found: 574.1818.

*2–(2-(3,4-dimethoxyphenyl)-5,7-dimethoxy-4-oxo-4H-chromen-3-yloxy)-N-(5–(3-nitrophenyl)-1,3,4-oxadiazol-2-yl)acetamide* **(B14)**. Light yellow solid, 36.20% yield, m.p.: 235–237 °C; ^1^H NMR (600 MHz, CDCl_3_) δ 13.10 (s, 1H), 8.91 (s, 1H), 8.46 (d, *J* = 7.6 Hz, 1H), 8.37 (d, *J* = 8.1 Hz, 1H), 7.70 (dd, *J* = 18.7, 8.6 Hz, 2H), 7.55 (s, 1H), 7.03 (d, *J* = 8.5 Hz, 1H), 6.58 (s, 1H), 6.43 (s, 1H), 4.41 (s, 2H), 4.03 (s, 3H), 3.99 (s, 3H), 3.98 (s, 3H), 3.94 (s, 3H). ^13 ^C NMR (151 MHz, CDCl_3_) δ 174.9, 166.8, 165.0, 161.1, 159.3, 159.1, 157.6, 154.7, 151.9, 149.3, 148.6, 140.8, 132.2, 130.2, 125.7, 125.6, 122.3, 121.7, 121.5, 111.3, 110.9, 108.4, 96.5, 92.7, 73.4, 56.6, 56.2, 56.1, 55.9. HRMS (ESI): *m/z* [M + H]^+^ calcd for C_29_H_25_N_4_O_11_: 605.1514; found: 605.1511.

*2–(2-(3,4-dimethoxyphenyl)-5,7-dimethoxy-4-oxo-4H-chromen-3-yloxy)-N-(5–(2-nitrophenyl)-1,3,4-oxadiazol-2-yl)acetamide* **(B15)**. Light yellow solid, 46.53% yield, m.p.: 254–256 °C; ^1^H NMR (600 MHz, DMSO-*d_6_*) δ 12.31 (s, 1H), 8.18 (d, *J* = 8.0 Hz, 1H), 8.00 (d, *J* = 7.5 Hz, 1H), 7.95 (t, *J* = 7.5 Hz, 1H), 7.91 (t, *J* = 7.6 Hz, 1H), 7.75 (d, *J* = 8.1 Hz, 2H), 7.12 (d, *J* = 8.4 Hz, 1H), 6.87 (s, 1H), 6.52 (s, 1H), 4.80 (s, 2H), 3.91 (s, 3H), 3.87–3.82 (m, 9H). ^13 ^C NMR (151 MHz, DMSO-*d_6_*) δ 172.8, 167.2, 164.4, 160.8, 158.6, 158.3, 157.5, 152.3, 151.4, 148.9, 148.20, 139.4, 134.2, 133.7, 131.6, 125.3, 122.6, 122.2, 117.5, 112.0, 111.9, 108.5, 96.6, 93.6, 70.7, 56.6, 56.5, 56.1, 56.1. HRMS (ESI): *m/z* [M + H]^+^ calcd for C_29_H_25_N_4_O_11_: 605.1514; found: 605.1510.

*2–(2-(3,4-dimethoxyphenyl)-5,7-dimethoxy-4-oxo-4H-chromen-3-yloxy)-N-(5–(4-(trifluoromethyl)phenyl)-1,3,4-oxadiazol-2-yl)acetamide* **(B16)**. White solid, 41.83% yield, m.p.: 210–212 °C; ^1^H NMR (600 MHz, CDCl_3_) δ 12.94 (s, 1H), 8.22 (d, *J* = 8.2 Hz, 2H), 7.75 (d, *J* = 8.3 Hz, 2H), 7.68 (dd, *J* = 8.5, 2.0 Hz, 1H), 7.55 (d, *J* = 2.0 Hz, 1H), 7.02 (d, *J* = 8.6 Hz, 1H), 6.57 (d, *J* = 2.2 Hz, 1H), 6.42 (d, *J* = 2.1 Hz, 1H), 4.40 (s, 2H), 4.01 (s, 3H), 3.99 (s, 3H), 3.97 (s, 3H), 3.93 (s, 3H). ^13 ^C NMR (151 MHz, CDCl_3_) δ 174.8, 166.8, 164.9, 161.0, 160.2, 159.0, 157.5, 154.7, 151.9, 149.3, 140.8, 132.9, 127.1, 126.9 (2 C), 125.9 (2 C), 123.6, 122.3, 121.7, 111.3, 110.9, 108.4, 96.5, 92.7, 73.3, 56.6, 56.2, 56.1, 55.9. HRMS (ESI): *m/z* [M + H]^+^ calcd for C_30_H_25_F_3_N_3_O_9_: 628.1537; found: 628.1535.

*2–(2-(3,4-dimethoxyphenyl)-5,7-dimethoxy-4-oxo-4H-chromen-3-yloxy)-N-(5–(3-(trifluoromethyl)phenyl)-1,3,4-oxadiazol-2-yl)acetamide* **(B17)**. White solid, 50.80% yield, m.p.: 195–197 °C; ^1^H NMR (600 MHz, CDCl_3_) δ 12.90 (s, 1H), 8.34 (s, 1H), 8.30 (d, *J* = 7.7 Hz, 1H), 7.76 (d, *J* = 7.5 Hz, 1H), 7.68 (d, *J* = 8.5 Hz, 1H), 7.64 (t, *J* = 7.8 Hz, 1H), 7.54 (s, 1H), 7.02 (d, *J* = 8.5 Hz, 1H), 6.57 (d, *J* = 1.8 Hz, 1H), 6.42 (s, 1H), 4.40 (s, 2H), 4.00 (s, 3H), 3.98 (s, 3H), 3.97 (s, 3H), 3.93 (s, 3H). ^13 ^C NMR (151 MHz, CDCl_3_) δ 174.8, 166.8, 165.0, 161.1, 160.1, 159.1, 157.4, 154.7, 151.9, 149.3, 140.8, 131.6, 129.8, 129.5, 127.8, 124.8, 123.6, 123.5, 122.3, 121.7, 111.3, 110.9, 108.4, 96.5, 92.7, 73.3, 56.5, 56.2, 56.1, 55.9. HRMS (ESI): *m/z* [M + H]^+^ calcd for C_30_H_25_F_3_N_3_O_9_: 628.1537; found: 628.1533.

*2–(2-(3,4-dimethoxyphenyl)-5,7-dimethoxy-4-oxo-4H-chromen-3-yloxy)-N-(5–(2-(trifluoromethyl)phenyl)-1,3,4-oxadiazol-2-yl)acetamide* **(B18)**. White solid, 58.25% yield, m.p.: 225–227 °C; ^1^H NMR (600 MHz, CDCl_3_) δ 12.99 (s, 1H), 8.05 (d, *J* = 7.3 Hz, 1H), 7.85 (d, *J* = 7.4 Hz, 1H), 7.72–7.63 (m, 3H), 7.54 (s, 1H), 7.01 (d, *J* = 8.6 Hz, 1H), 6.55 (d, *J* = 1.7 Hz, 1H), 6.40 (d, *J* = 1.6 Hz, 1H), 4.39 (s, 2H), 4.00–3.95 (m, 9H), 3.92 (s, 3H). ^13 ^C NMR (151 MHz, CDCl_3_) δ 174.8, 166.7, 165.0, 161.3, 159.4, 159.1, 158.0, 154.6, 151.9, 149.4, 140.9, 131.9, 131.3, 129.2, 129.0, 126.9, 123.2, 122.6, 122.3, 121.9, 111.4, 111.1, 108.6, 96.5, 92.8, 73.4, 56.5, 56.3, 56.1, 55.9. HRMS (ESI): *m/z* [M + H]^+^ calcd for C_30_H_25_F_3_N_3_O_9_: 628.1537; found: 628.1537.

*2–(2-(3,4-dimethoxyphenyl)-5,7-dimethoxy-4-oxo-4H-chromen-3-yloxy)-N-(5–(4-(trifluoromethoxy)phenyl)-1,3,4-oxadiazol-2-yl)acetamide* **(B19)**. White solid, 54.40% yield, m.p.: 217–219 °C; ^1^H NMR (600 MHz, CDCl_3_) δ 12.78 (s, 1H), 8.13 (d, *J* = 8.7 Hz, 2H), 7.68 (dd, *J* = 8.5, 1.8 Hz, 1H), 7.54 (d, *J* = 1.6 Hz, 1H), 7.33 (d, *J* = 8.3 Hz, 2H), 7.02 (d, *J* = 8.6 Hz, 1H), 6.57 (d, *J* = 1.9 Hz, 1H), 6.42 (d, *J* = 1.9 Hz, 1H), 4.39 (s, 2H), 4.00 (s, 3H), 3.98 (s, 3H), 3.97 (s, 3H), 3.93 (s, 3H). ^13 ^C NMR (151 MHz, CDCl_3_) δ 174.7, 166.8, 164.9, 161.0, 160.3, 159.0, 157.2, 154.6, 151.8, 151.3, 149.2, 140.7, 128.4 (2 C), 122.4, 122.2, 121.7, 121.1 (2 C), 120.3, 111.2, 110.9, 108.4, 96.4, 92.7, 73.2, 56.5, 56.2, 56.1, 55.9. HRMS (ESI): *m/z* [M + H]^+^ calcd for C_30_H_25_F_3_N_3_O_10_: 644.1487; found: 644.1484.

*2–(2-(3,4-dimethoxyphenyl)-5,7-dimethoxy-4-oxo-4H-chromen-3-yloxy)-N-(5–(3-(trifluoromethoxy)phenyl)-1,3,4-oxadiazol-2-yl)acetamide* **(B20)**. White solid, 38.85% yield, m.p.: 207–209 °C; ^1^H NMR (600 MHz, CDCl_3_) δ 12.78 (s, 1H), 8.04 (d, *J* = 7.7 Hz, 1H), 7.94 (s, 1H), 7.70–7.65 (m, 1H), 7.57–7.51 (m, 2H), 7.36 (d, *J* = 7.7 Hz, 1H), 7.02 (d, *J* = 8.5 Hz, 1H), 6.57 (d, *J* = 1.7 Hz, 1H), 6.42 (d, *J* = 1.6 Hz, 1H), 4.40 (s, 2H), 4.00 (s, 3H), 3.98 (s, 3H), 3.97 (s, 3H), 3.93 (s, 3H). ^13 ^C NMR (151 MHz, CDCl_3_) δ 174.7, 166.7, 164.9, 161.1, 160.1, 159.0, 157.3, 154.6, 151.9, 149.6, 149.4, 140.8, 130.5, 125.8, 124.9, 123.5, 122.2, 121.8, 120.4, 119.2, 111.4, 111.1, 108.5, 96.4, 92.8, 73.3, 56.5, 56.3, 56.1, 55.9. HRMS (ESI): *m/z* [M + H]^+^ calcd for C_30_H_25_F_3_N_3_O_10_: 644.1487; found: 644.1486.

*2–(2-(3,4-dimethoxyphenyl)-5,7-dimethoxy-4-oxo-4H-chromen-3-yloxy)-N-(5–(2-(trifluoromethoxy)phenyl)-1,3,4-oxadiazol-2-yl)acetamide* **(B21)**. White solid, 43.71% yield, m.p.: 226–228 °C; ^1^H NMR (600 MHz, CDCl_3_) δ 12.82 (s, 1H), 8.16 (d, *J* = 7.5 Hz, 1H), 7.67 (d, *J* = 8.1 Hz, 1H), 7.59–7.52 (m, 2H), 7.48–7.40 (m, 2H), 7.01 (d, *J* = 8.4 Hz, 1H), 6.56 (s, 1H), 6.40 (s, 1H), 4.40 (s, 2H), 4.01–3.90 (m, 12H). ^13 ^C NMR (151 MHz, CDCl_3_) δ 174.6, 166.6, 164.9, 161.2, 159.0, 158.3, 157.6, 154.5, 151.9, 149.3, 146.4, 140.8, 132.4, 130.5, 127.2, 122.2, 122.1, 121.9, 120.5, 118.2, 111.3, 111.1, 108.5, 96.4, 92.7, 73.3, 56.4, 56.3, 56.1, 55.9. HRMS (ESI): *m/z* [M + H]^+^ calcd for C_30_H_25_F_3_N_3_O_10_: 644.1487; found: 644.1482.

*2–(2-(3,4-dimethoxyphenyl)-5,7-dimethoxy-4-oxo-4H-chromen-3-yloxy)-N-(5–(4-methoxyphenyl)-1,3,4-oxadiazol-2-yl)acetamide* **(B22)**. White solid, 44.52% yield, m.p.: 206–208 °C; ^1^H NMR (600 MHz, CDCl_3_) δ 12.51 (s, 1H), 8.02 (d, *J* = 8.7 Hz, 2H), 7.67 (d, *J* = 8.5 Hz, 1H), 7.54 (s, 1H), 7.02 (d, *J* = 8.5 Hz, 1H), 6.98 (d, *J* = 8.7 Hz, 2H), 6.56 (s, 1H), 6.41 (s, 1H), 4.39 (s, 2H), 4.01–3.96 (m, 9H), 3.93 (s, 3H), 3.87 (s, 3H). ^13 ^C NMR (151 MHz, CDCl_3_) δ 174.6, 166.7, 164.9, 162.1, 161.5, 161.1, 159.0, 156.5, 154.5, 151.8, 149.3, 140.7, 128.5 (2 C), 122.2, 121.9, 116.5, 114.3 (2 C), 111.3, 110.9, 108.5, 96.4, 92.7, 73.2, 56.5, 56.3, 56.1, 55.9, 55.4. HRMS (ESI): *m/z* [M + H]^+^ calcd for C_30_H_28_N_3_O_10_: 590.1769; found: 590.1769.

*2–(2-(3,4-dimethoxyphenyl)-5,7-dimethoxy-4-oxo-4H-chromen-3-yloxy)-N-(5–(3-methoxyphenyl)-1,3,4-oxadiazol-2-yl)acetamide* **(B23)**. White solid, 47.70% yield, m.p.: 205–207 °C; ^1^H NMR (400 MHz, CDCl_3_) δ 12.67 (s, 1H), 7.70–7.64 (m, 2H), 7.63–7.60 (m, 1H), 7.54 (d, *J* = 2.0 Hz, 1H), 7.38 (t, *J* = 8.0 Hz, 1H), 7.07–7.03 (m, 1H), 7.01 (d, *J* = 8.6 Hz, 1H), 6.56 (d, *J* = 2.2 Hz, 1H), 6.41 (d, *J* = 2.2 Hz, 1H), 4.39 (s, 2H), 3.99 (s, 3H), 3.98 (s, 3H), 3.97 (s, 3H), 3.93 (s, 3H), 3.88 (s, 3H). ^13 ^C NMR (151 MHz, CDCl_3_) δ 174.7, 166.8, 164.9, 161.4, 161.1, 159.9, 159.1, 156.9, 154.5, 151.8, 149.2, 140.7, 129.9, 124.9, 122.2, 121.8, 119.2, 118.1, 111.2, 111.1, 110.9, 108.4, 96.4, 92.7, 73.2, 56.6, 56.2, 56.1, 55.9, 55.5. HRMS (ESI): *m/z* [M + H]^+^ calcd for C_30_H_28_N_3_O_10_: 590.1769; found: 590.1764.

*N-(5–(3-chloro-4-fluorophenyl)-1,3,4-oxadiazol-2-yl)-2–(2-(3,4-dimethoxyphenyl)-5,7-dimethoxy-4-oxo-4H-chromen-3-yloxy)acetamide* **(B24)**. White solid, 40.86% yield, m.p.: 232–234 °C; ^1^H NMR (600 MHz, CDCl_3_) δ 12.85 (s, 1H), 8.16 (dd, *J* = 6.9, 2.0 Hz, 1H), 8.02–7.98 (m, 1H), 7.68 (dd, *J* = 8.5, 1.9 Hz, 1H), 7.54 (d, *J* = 1.7 Hz, 1H), 7.27 (t, *J* = 8.6 Hz, 2H), 7.02 (d, *J* = 8.6 Hz, 1H), 6.57 (d, *J* = 2.1 Hz, 1H), 6.42 (d, *J* = 2.0 Hz, 1H), 4.39 (s, 2H), 4.01 (s, 3H), 3.99 (s, 3H), 3.97 (s, 3H), 3.93 (s, 3H). ^13 ^C NMR (151 MHz, CDCl_3_) δ 174.8, 166.8, 164.9, 161.1, 160.1, 159.1, 159.0, 157.2, 154.7, 151.9, 149.3, 140.8, 129.1, 126.8, 122.3, 122.3, 121.8, 121.2, 117.3, 111.4, 110.9, 108.4, 96.5, 92.8, 73.3, 56.6, 56.3, 56.1, 55.9. HRMS (ESI): *m/z* [M + H]^+^ calcd for C_29_H_24_ClFN_3_O_9_: 612.1180; found: 612.1180.

*N-(5–(3,4-dichlorophenyl)-1,3,4-oxadiazol-2-yl)-2–(2-(3,4-dimethoxyphenyl)-5,7-dimethoxy-4-oxo-4H-chromen-3-yloxy)acetamide* **(B25)**. White solid, 35.80% yield, m.p.: 230–232 °C; ^1^H NMR (600 MHz, DMSO-*d_6_*) δ 12.25 (s, 1H), 8.04 (s, 1H), 7.87 (s, 2H), 7.77–7.72 (m, 2H), 7.11 (d, *J* = 8.4 Hz, 1H), 6.85 (s, 1H), 6.51 (s, 1H), 4.80 (s, 2H), 3.90 (s, 3H), 3.88–3.84 (m, 6H), 3.83 (s, 3H). ^13 ^C NMR (151 MHz, DMSO-*d_6_*) δ 172.8, 167.3, 164.5, 160.8, 159.3, 158.6, 157.9, 152.4, 151.5, 148.9, 139.4, 134.8, 132.7, 132.3, 127.9, 126.5, 124.3, 122.7, 122.2, 112.2, 111.9, 108.6, 96.6, 93.6, 70.9, 56.6, 56.5, 56.2, 56.1. HRMS (ESI): *m/z* [M + H]^+^ calcd for C_29_H_24_Cl_2_N_3_O_9_: 628.0884; found: 628.0884.

*N-(5–(3,5-dimethoxyphenyl)-1,3,4-oxadiazol-2-yl)-2–(2-(3,4-dimethoxyphenyl)-5,7-dimethoxy-4-oxo-4H-chromen-3-yloxy)acetamide* **(B26)**. White solid, 45.40% yield, m.p.: 200–202 °C; ^1^H NMR (600 MHz, CDCl_3_) δ 12.52 (s, 1H), 7.67 (dd, *J* = 8.5, 1.9 Hz, 1H), 7.55 (d, *J* = 1.8 Hz, 1H), 7.23 (d, *J* = 2.2 Hz, 2H), 7.01 (d, *J* = 8.6 Hz, 1H), 6.59 (t, *J* = 2.2 Hz, 1H), 6.56 (d, *J* = 2.1 Hz, 1H), 6.41 (d, *J* = 2.0 Hz, 1H), 4.40 (s, 2H), 3.99 (s, 3H), 3.98 (s, 3H), 3.97 (s, 3H), 3.93 (s, 3H), 3.85 (s, 6H). ^13 ^C NMR (151 MHz, CDCl_3_) δ 174.6, 166.7, 164.9, 161.5, 161.1, 161.1 (2 C), 159.0, 156.9, 154.5, 151.8, 149.3, 140.7, 125.4, 122.2, 121.8, 111.3, 110.9, 108.5, 104.5 (2 C), 104.3, 96.4, 92.7, 73.2, 56.5, 56.3, 56.1, 55.9, 55.6 (2 C). HRMS (ESI): *m/z* [M + H]^+^ calcd for C_31_H_30_N_3_O_11_: 620.1875; found: 620.1873.

*N-(5–(3,4-dimethoxyphenyl)-1,3,4-oxadiazol-2-yl)-2–(2-(3,4-dimethoxyphenyl)-5,7-dimethoxy-4-oxo-4H-chromen-3-yloxy)acetamide* **(B27)**. White solid, 42.37% yield, m.p.: 151–153 °C; ^1^H NMR (600 MHz, CDCl_3_) δ 12.62 (s, 1H), 7.66 (d, *J* = 8.5 Hz, 1H), 7.64 (d, *J* = 8.3 Hz, 1H), 7.59 (s, 1H), 7.54 (s, 1H), 7.01 (d, *J* = 8.5 Hz, 1H), 6.93 (d, *J* = 8.3 Hz, 1H), 6.55 (s, 1H), 6.40 (s, 1H), 4.38 (s, 2H), 4.01–3.90 (m, 18H). ^13 ^C NMR (151 MHz, CDCl_3_) δ 174.7, 166.8, 164.9, 161.6, 161.1, 159.0, 156.6, 154.6, 151.9, 151.8, 149.3(2 C), 140.7, 122.2, 121.8, 120.3, 116.5, 111.3, 111.1, 111.0, 109.4, 108.5, 96.4, 92.8, 73.2, 56.5, 56.3, 56.2, 56.1, 55.9, 55.9. HRMS (ESI): *m/z* [M + H]^+^ calcd for C_31_H_30_N_3_O_11_: 620.1875; found: 620.1872.

*2–(2-(3,4-dimethoxyphenyl)-5,7-dimethoxy-4-oxo-4H-chromen-3-yloxy)-N-(5-(naphthalen-1-yl)-1,3,4-oxadiazol-2-yl)acetamide* **(B28)**. White solid, 51.26% yield, m.p.: 230–232 °C; ^1^H NMR (600 MHz, CDCl_3_) δ 12.64 (s, 1H), 9.28 (d, *J* = 8.4 Hz, 1H), 8.24 (d, *J* = 7.1 Hz, 1H), 8.00 (d, *J* = 7.9 Hz, 1H), 7.91 (d, *J* = 8.0 Hz, 1H), 7.68 (t, *J* = 7.9 Hz, 2H), 7.61–7.53 (m, 3H), 7.02 (d, *J* = 8.5 Hz, 1H), 6.56 (s, 1H), 6.41 (s, 1H), 4.44 (s, 2H), 4.03–3.96 (m, 9H), 3.93 (s, 3H). ^13 ^C NMR (151 MHz, CDCl_3_) δ 174.7, 166.8, 164.9, 161.6, 161.2, 159.1, 156.8, 154.5, 151.9, 149.4, 140.8, 133.9, 132.2, 130.1, 128.5, 128.3, 128.1, 126.6, 126.5, 124.9, 122.3, 121.9, 120.5, 111.4, 111.2, 108.6, 96.5, 92.8, 73.3, 56.6, 56.4, 56.1, 55.9. HRMS (ESI): *m/z* [M + H]^+^ calcd for C_33_H_28_N_3_O_9_: 610.1820; found: 610.1817.

*N-(5–(2,3-dihydrobenzo[b][1, 4]dioxin-6-yl)-1,3,4-oxadiazol-2-yl)-2–(2-(3,4-dimethoxyphenyl)-5,7-dimethoxy-4-oxo-4H-chromen-3-yloxy)acetamide* **(B29)**. White solid, 50.60% yield, m.p.: 228–230 °C; ^1^H NMR (600 MHz, CDCl_3_) δ 12.45 (s, 1H), 7.67 (dd, *J* = 8.5, 1.6 Hz, 1H), 7.60 (d, *J* = 1.6 Hz, 1H), 7.57 (dd, *J* = 8.5, 1.6 Hz, 1H), 7.55 (s, 1H), 7.01 (d, *J* = 8.6 Hz, 1H), 6.94 (d, *J* = 8.4 Hz, 1H), 6.56 (d, *J* = 1.8 Hz, 1H), 6.41 (d, *J* = 1.7 Hz, 1H), 4.39 (s, 2H), 4.30 (dd, *J* = 12.1, 5.0 Hz, 4H), 3.99 (s, 3H), 3.98 (s, 3H), 3.97 (s, 3H), 3.93 (s, 3H). ^13 ^C NMR (151 MHz, CDCl_3_) δ 174.6, 166.7, 164.9, 161.3, 161.1, 159.0, 156.5, 154.5, 151.9, 149.3, 146.4, 143.8, 140.7, 122.2, 121.9, 120.4, 117.8, 117.2, 115.9, 111.3, 111.0, 108.5, 96.4, 92.7, 73.2, 64.6, 64.2, 56.5, 56.3, 56.1, 55.9. HRMS (ESI): *m/z* [M + H]^+^ calcd for C_31_H_28_N_3_O_11_: 618.1718; found: 618.1715.

*N-(5–(3-(benzyloxy)phenyl)-1,3,4-oxadiazol-2-yl)-2–(2-(3,4-dimethoxyphenyl)-5,7-dimethoxy-4-oxo-4H-chromen-3-yloxy)acetamide* **(B30)**. White solid, 47.88% yield, m.p.: 156–158 °C; ^1^H NMR (600 MHz, CDCl_3_) δ 12.58 (s, 1H), 7.72 (s, 1H), 7.70–7.65 (m, 2H), 7.55 (d, *J* = 1.5 Hz, 1H), 7.46 (d, *J* = 7.3 Hz, 2H), 7.39 (t, *J* = 7.4 Hz, 3H), 7.33 (t, *J* = 7.2 Hz, 1H), 7.11 (dd, *J* = 8.1, 1.7 Hz, 1H), 7.01 (d, *J* = 8.6 Hz, 1H), 6.56 (d, *J* = 1.9 Hz, 1H), 6.40 (d, *J* = 1.8 Hz, 1H), 5.14 (s, 2H), 4.40 (s, 2H), 4.00–3.90 (m, 12H). ^13 ^C NMR (151 MHz, CDCl_3_) δ 174.7, 166.7, 164.9, 161.4, 161.1, 159.1, 159.0, 156.9, 154.5, 151.9, 149.3, 140.7, 136.5, 130.1, 128.6 (2 C), 128.1, 127.6 (2 C), 125.0, 122.2, 121.8, 119.4, 118.6, 112.3, 111.3, 110.9, 108.5, 96.4, 92.7, 73.2, 70.3, 56.5, 56.2, 56.1, 55.9. HRMS (ESI): *m/z* [M + H]^+^ calcd for C_36_H_32_N_3_O_10_: 666.2082; found: 666.2079.

*2–(2-(3,4-dimethoxyphenyl)-5,7-dimethoxy-4-oxo-4H-chromen-3-yloxy)-N-(5-(furan-2-yl)-1,3,4-oxadiazol-2-yl)acetamide* **(B31)**. White solid, 47.78% yield, m.p.: 233–235 °C; ^1^H NMR (600 MHz, CDCl_3_) δ 12.73 (s, 1H), 7.67 (dd, *J* = 8.5, 1.9 Hz, 1H), 7.61 (d, *J* = 0.9 Hz, 1H), 7.54 (d, *J* = 1.7 Hz, 1H), 7.16 (d, *J* = 2.9 Hz, 1H), 7.01 (d, *J* = 8.6 Hz, 1H), 6.58–6.57 (m, 1H), 6.57 (d, *J* = 2.2 Hz, 1H), 6.41 (d, *J* = 1.9 Hz, 1H), 4.39 (s, 2H), 4.00 (s, 3H), 3.98 (s, 3H), 3.97 (s, 3H), 3.93 (s, 3H). ^13 ^C NMR (151 MHz, CDCl_3_) δ 174.7, 166.7, 164.9, 161.1, 159.0, 156.4, 154.6, 154.3, 151.8, 149.3, 145.3, 140.8, 139.3, 122.2, 121.8, 113.5, 111.9, 111.3, 110.9, 108.4, 96.4, 92.7, 73.2, 56.6, 56.2, 56.1, 55.9. HRMS (ESI): *m/z* [M + H]^+^ calcd for C_27_H_24_N_3_O_10_: 550.1456; found: 550.1457.

*2–(2-(3,4-dimethoxyphenyl)-5,7-dimethoxy-4-oxo-4H-chromen-3-yloxy)-N-(5-(thiophen-2-yl)-1,3,4-oxadiazol-2-yl)acetamide* **(B32)**. White solid, 49.73% yield, m.p.: 238–240 °C; ^1^H NMR (600 MHz, CDCl_3_) δ 12.54 (s, 1H), 7.78 (d, *J* = 2.7 Hz, 1H), 7.67 (d, *J* = 8.5 Hz, 1H), 7.55 (s, 1H), 7.51 (d, *J* = 4.5 Hz, 1H), 7.15 (t, *J* = 4.2 Hz, 1H), 7.02 (d, *J* = 8.5 Hz, 1H), 6.57 (s, 1H), 6.42 (s, 1H), 4.40 (s, 2H), 4.00 (s, 3H), 3.98 (s, 3H), 3.97 (s, 3H), 3.93 (s, 3H). ^13 ^C NMR (151 MHz, CDCl_3_) δ 174.6, 166.7, 164.9, 161.2, 159.0, 157.9, 156.3, 154.5, 151.9, 149.4, 140.7, 129.5, 129.4, 127.8, 125.3, 122.2, 121.9, 111.4, 111.1, 108.6, 96.4, 92.8, 73.2, 56.5, 56.3, 56.1, 55.9. HRMS (ESI): *m/z* [M + H]^+^ calcd for C_27_H_24_N_3_O_9_S: 566.1228; found: 566.1227.

*(E)-2–(2-(3,4-dimethoxyphenyl)-5,7-dimethoxy-4-oxo-4H-chromen-3-yloxy)-N-(5-styryl-1,3,4-oxadiazol-2-yl)acetamide* **(B33)**. White solid, 42.70% yield, m.p.: 226–228 °C; ^1^H NMR (600 MHz, CDCl_3_) δ 12.64 (s, 1H), 7.70–7.65 (m, 1H), 7.60–7.52 (m, 4H), 7.43–7.34 (m, 3H), 7.03 (d, *J* = 2.4 Hz, 1H), 7.01 (d, *J* = 5.1 Hz, 1H), 6.57 (d, *J* = 1.9 Hz, 1H), 6.41 (d, *J* = 1.8 Hz, 1H), 4.39 (s, 2H), 4.00 (s, 3H), 3.98 (s, 3H), 3.97 (s, 3H), 3.93 (s, 3H). ^13 ^C NMR (151 MHz, CDCl_3_) δ 174.7, 166.6, 164.9, 161.1, 161.1, 159.1, 156.4, 154.6, 151.9, 149.3, 140.8, 138.1, 135.0, 129.6, 128.9, 127.4, 122.2, 121.8, 111.3, 110.9, 109.9, 108.5, 96.4, 92.8, 73.3, 56.6, 56.2, 56.1, 55.9. HRMS (ESI): *m/z* [M + H]^+^ calcd for C_31_H_28_N_3_O_9_: 586.1820; found: 586.1816.

### 2.3. Telomerase activity assay^23^

#### 2.4. Cell culture

A375, MDA-MB-231, MGC-803, SMMC-7721, SGC-7901 and L-02 cell lines were cultured in DMEM medium supplemented with 10% (V/V) heat-inactivated fetal bovine serum (FBS) (Biological Industries, Israel) along with 100 U/mL penicillin and 100 mg/mL streptomycin (Beyotime, China). Cells were grown in a humidified 5% CO_2_ atmosphere at 37 °C and maintained in a logarithmic growth phase for all experiments.

### 2.5. Anticancer assay^23^

#### 2.6. Cell cycle assay

For cell cycle analysis, cell cycle kit (Beyotime, China) was performed. MGC-803 cells were treated with compound **A33** at different concentrations for 48 h. Untreated and treated cells were harvested, and then MGC-803 cells were washed three times using cold PBS. And then cells were fixed in 70% ethanol at −20 °C for 1 h. After fixation, cells were washed with cold PBS and stained with 0.5 ml of propidium iodide (PI) staining buffer, which contain 200 mg/mL RNase A and 50 µg/mL PI, at 37 °C for 30 min in the dark. Analyses were conducted on FACSVerse Flow Cytometer (Becton Dickinson). The experiments were repeated three times.

### 2.7. Apoptosis assay

For cell apoptosis analysis, we employed annexin V-FITC/PI apoptosis detection kit (BestBio, China). MGC-803 cells in logarithmic growth phase were treated with compound **A33** at different concentrations for 48 h. Cells were collected in cold PBS by centrifugation for 5 min at 1000 g. And then cells were re-suspended at a buffer (1 × 10^6^ cells/mL), stained with FITC-labeled annexin V and PI for 20 min in the dark and immediately analysed on FACSVerse Flow Cytometer (Becton Dickinson).

### 2.8. Western blotting

Human MGC-803 cells were lysed with RIPA lysis buffer (Beyotime, China). Whole extracts were prepared, and protein concentration was detected using a BCA protein assay kit (Beyotime, China). The protein samples were separated by SDS-PAGE and blotted onto a PVDF membrane (Millipore Corp, Billerica, MA). After blockade of non-specific protein binding, nitrocellulose blots were incubated at 4 °C for 8 h with primary antibodies. After extensive washing in TBS/Tween-20, the membranes were incubated at room temperature for 1 h with secondary antibodies. After washed in TBS/Tween-20, the blots were processed with distilled water for detection of antigen using the enhanced chemiluminescence system. Proteins were visualised with ECL-chemiluminescent kit (ECL-plus, Thermo Scientific).

### 2.9. Statistical analysis

All data are expressed as means ± SD. Student's *t*-test was used to determine statistical significance at *p* < 0.05. SPSS 17.0 and Graphpad Prism 5 software were used for the statistical analyses.

## 3. Results and discussion

### 3.1. Chemistry

**Scheme 1.**
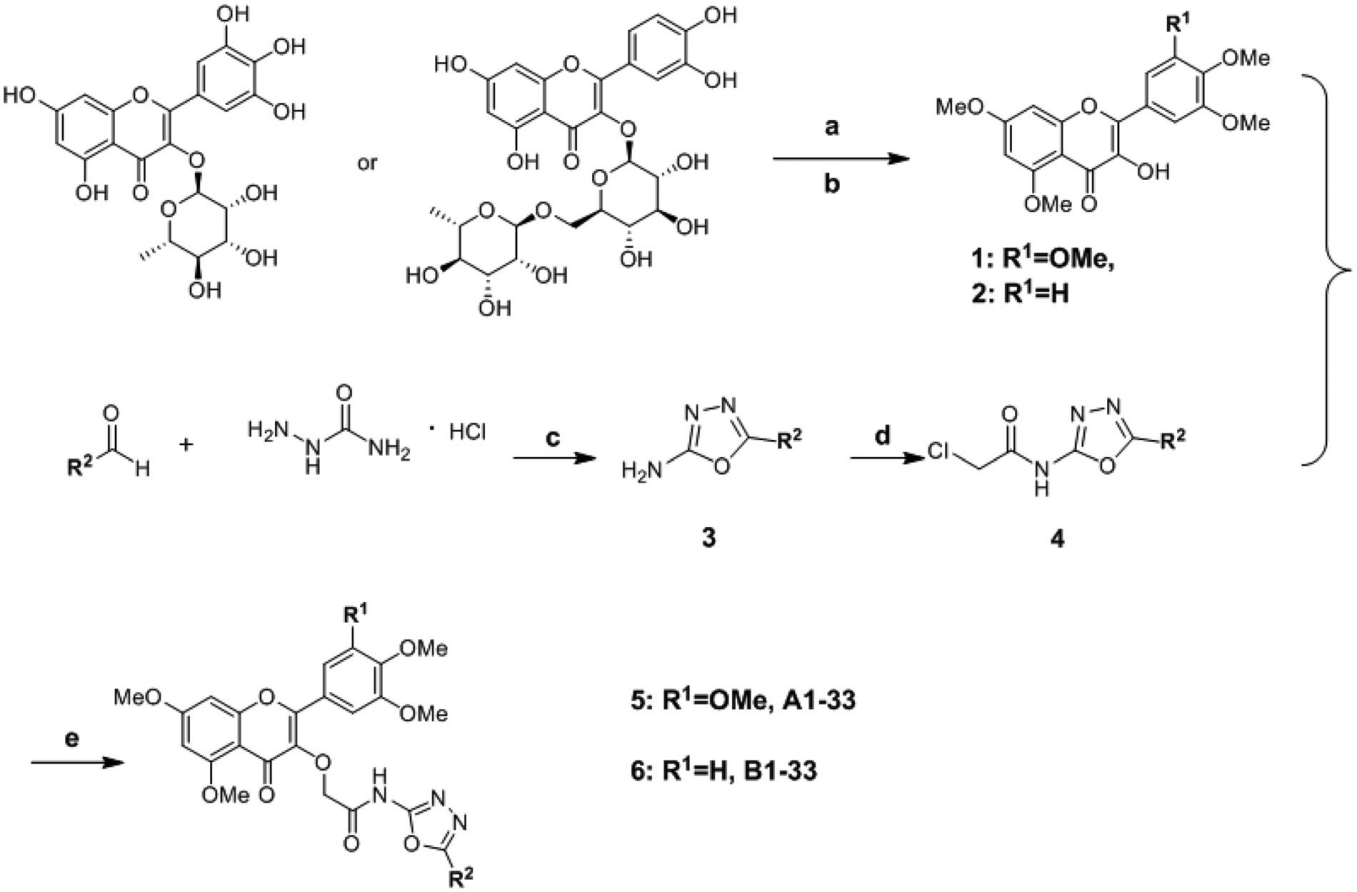
Synthesis of title compounds **A1–A33** and **B1–B33**. Reagent and conditions: (**a**) K_2_CO_3_, (CH_3_)_2_SO_4_, acetone, reflux, 48 h; (**b**) Conc.HCl, EtOH, reflux, 2 h; (**c**) AcONa, MeOH/H_2_O, rt, K_2_CO_3_, I_2_, 1,4-dioxane, 85 °C, 5 h; (**d**) Chloroacetyl chloride, DMF, rt, 12 h; (**e**) K_2_CO_3_, KI, acetone, reflux, overnight.

Myricitrin and Rutin are used as raw materials. The hydroxyl groups on the benzene ring were protected by methylation with dimethyl sulphate, and the glycosides were removed under strong acidic and reflux conditions to obtain 3-hydroxy-5,7-dimethoxy-2–(3,4,5-trimethoxyphenyl)-4H-chromen-4-one (**1**)^22^ and 2–(3,4-dimethoxyphenyl)-3-hydroxy-5,7-dimethoxy-4H-chromen-4-one (**2**)^34^, respectively. Secondly, a series of 5-substituted-1,3,4-oxadiazol-2-amines (**3**) were synthesised by the condensation of semicarbazide hydrochloride and the corresponding aldehydes and following by I_2_**4**)^35^ were prepared from reacting of the intermediate **3** with chloroethyl acid chloride in the presence of anhydrous DMF. Finally, title compounds, 2-phenyl-4H-chromone derivatives containing 1,3,4-oxadiazole and amide moieties, were synthesised by refluxing the key intermediate **1** with **4** in the presence of K_2_CO_3_ and KI in acetone. The synthetic route of title compounds **A1-A33** and **B1-B33** was showed in Scheme 1. All title compounds were characterised by means of ^1^H NMR, ^13 ^C NMR and HR-MS spectral analysis.

### 3.2. Crystal structure analysis

**Figure 2.**
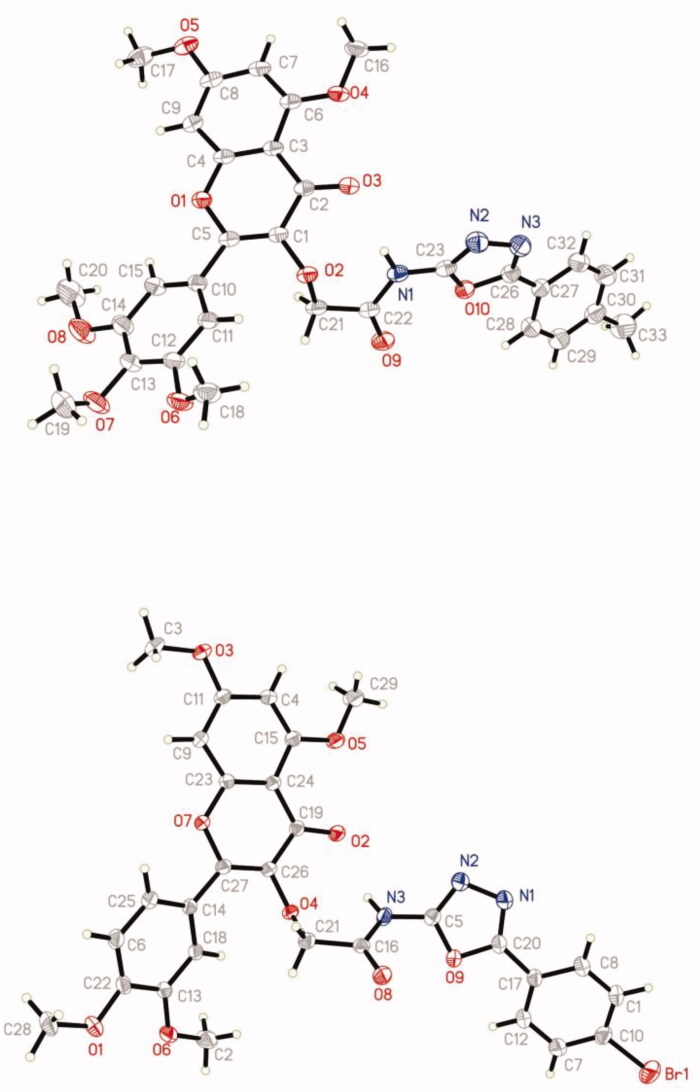
ORTEP drawing of compounds **A11** and **B8**.

The structure of compounds **A11** and **B8** was further determined by X-ray crystallography. The crystal data were presented in Table 1. The molecular structure of compounds **A11** and **B8** was showed in Figure 2, respectively. Crystallographic data (excluding structure factors) for the structure had been deposited with the Cambridge Crystallographic Data Centre as supplementary publication No. CCDC 2010105 and 2005763.

**Table 1.** Crystallographical and experimental data of compounds **A11** and **B8**.

Properties	A11	B8
Chemical formula	C_31_H_29_N_3_O_10_	C_29_H_24_BrN_3_O_9_
Formula weight	603.57	638.42
Temperature/K	292.56(16)	293(2)
Crystal system	Monoclinic	Monoclinic
Space group	*P2_1_/n*	*P2_1_/c*
a/Å	20.5711(10)	11.1453(2)
b/Å	7.5520(3)	29.8454(7)
c/Å	20.9843(11)	8.3870(3)
α/°	90	90
β/°	116.413(6)	101.156(3)
γ/°	90	90
Volume/Å^3^	2919.7(3)	2737.11(12)
Z	4	4
ρ_calc_g/cm^3^	1.369	1.549
μ/mm^1^	0.849	1.564
F(000)	1264.0	1304.0
Crystal size/mm^3^	0.17 × 0.04 × 0.02	0.25 × 0.22 × 0.19
2Θ range for data collection/°	8.078 to 133.186	3.72–52
Index ranges		
Reflections collected	11523	22575
Data/restraints/parameters	5029/0/403	5385/0/379
Goodness-of-fit on F^2^	1.036	1.012
Final R indexes [*I* ≥ 2σ (I)]	R_1_= 0.0529, wR_2_= 0.1381	R_1_= 0.0472, wR_2_= 0.1039
Final R indexes [all data]	R_1_= 0.0739, wR_2_= 0.1577	R_1_= 0.0705, wR_2_= 0.1137
Largest diff. peak/hole/e Å^−3^	0.25/−0.23	0.62/−0.69

### 3.3. Telomerase inhibitory activity and SAR

All title compounds were assayed for telomerase activity using MGC-803 cells extract, Staurosporine and BIBR1532 used as the references^23^. The results were presented as mean ± SD, summarised in Table 2. Most of the title compounds demonstrated potent inhibition against telomerase. Among these, compounds **A2**, **A5**, **A16**, **A20**, **A27**, **A33**, **B27** and **B33** displayed significant inhibitory activity (IC_50_ < 1 µM), with IC_50_ values of 0.77, 0.81, 0.62, 0.92, 0.32, 0.44, 0.51 and 0.97 µM, respectively, which were found to be obviously superior to staurosporine (IC_50_ = 6.41 µM), and were comparable to BIBR1532 (IC_50_ = 0.29 µM). Moreover, these compounds have stronger telomerase inhibitory effect than the myricetin^22^ and 1,3,4-oxadiazole^33^ derivatives we reported previously.

**Table 2.** Chemical structures of compounds **A1–A33** and **B1–B33** and inhibitory activity on telomerase.

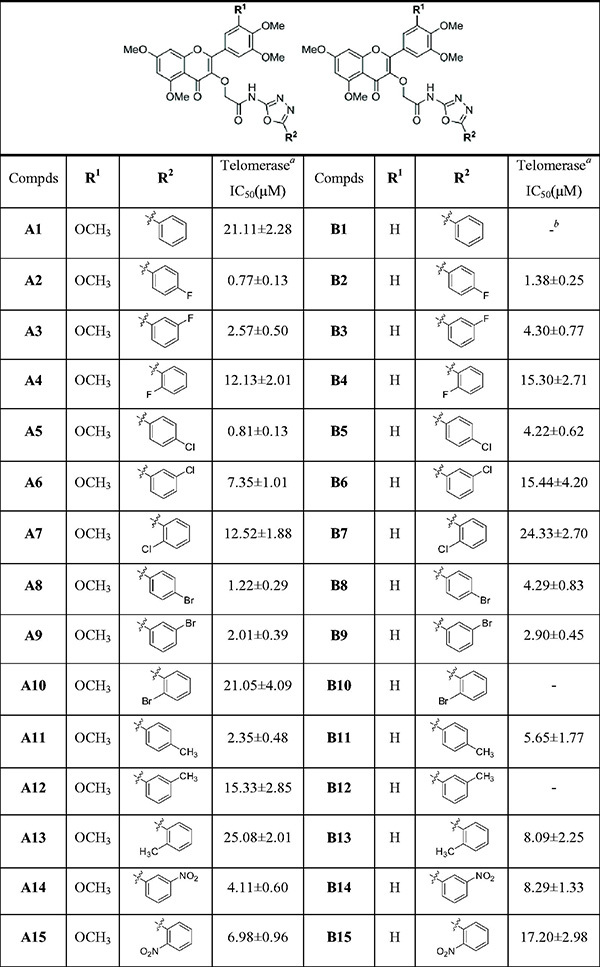	
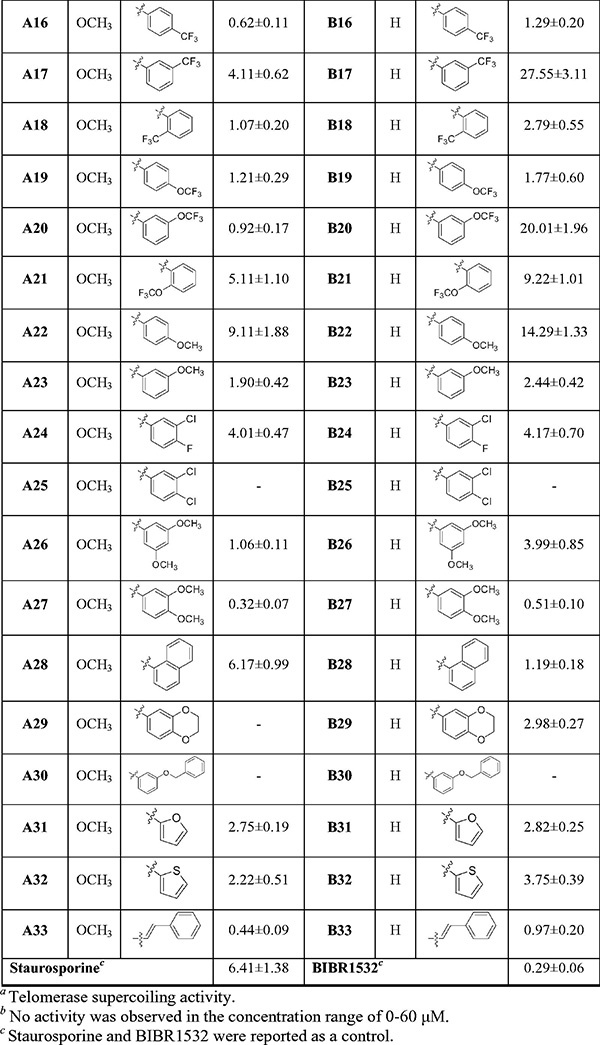	

Based on the data of Table 2, the preliminary SARs analysis revealed that except compounds **A13**, **A25**, **A28**, **A29** and **A30**, other compounds of **A** series (**R^1^** = OCH_3_) possessed higher telomerase inhibitory activity than **B** series (**R^1^** = H). Therefore, it could be seen that the methoxy group (OCH_3_) as a substituent at the **R^1^** position played a vital role in telomerase inhibitory activity.

The position, type and number of the substituents on the phenyl ring at **R^2^** and electronic effect had significant effects on inhibition of telomerase. Firstly, by comparing compounds **A2**–**A10**, when the phenyl ring at **R^2^** was substituted with halogen (F, Cl, Br), the inhibitory activity was *para* > *meta* > *ortho*. In addition, the substitution of halogen in the *para* and *ortho* position at the phenyl ring increased the activity with the increase of electronegativity (F > Cl > Br). A similar trend was also observed by comparing compounds **B2**-**B7**, **B8**, **B10**. Furthermore, compounds **A24**, **A25**, **B24** and **B25** disubstituted in the *para* and *meta* position at the phenyl ring demonstrated significant reduction or even complete loss of inhibitory activity as compared to the *para*-substituted compounds **A2**, **A5**, **B2** and **B5**. Secondly, by comparing compounds **A2-A21**, it was found that compounds with electron-withdrawing groups (F, Cl, Br, NO_2_, CF_3_, OCF_3_) on the phenyl ring at **R^2^** displayed higher inhibitory activity than those with electron-donating groups (CH_3_).

Interestingly, as compared to compounds **A22** and **A23**, compounds **A26** and **A27** bearing two the methoxy groups on the phenyl ring at **R^2^** exhibited stronger activity. A similar trend was also observed at compounds **B22**, **B23**, **B26** and **B27**. However, compounds **A30** and **B30** with a benzyl group at the *meta* position of the phenyl ring at **R^2^** completely lost inhibitory activity, which might be affected by steric hindrance. Finally, we found that replacement of the phenyl group at **R^2^** with aromatic fused rings and different aromatic heterocycles was also greatly important for activity.

As compared to compound **A1**, compounds **A28**, **A31**, **A32** substituted by naphthalene ring, furan ring and thiophene ring at **R^2^,** respectively, displayed more potent inhibitory activity. A similar trend was also observed by comparing compounds **B1**, **B28**, **B31** and **B32**. In particular, replacement of the phenyl group at **R^2^** with styryl yielded compound **A33** and **B33**, which significantly increased inhibitory activity as compared to compounds **A1** and **B1**. It can be seen that styryl is crucial for activity and should be further optimised in the future study.

### 3.4. In vitro anticancer activity

The most active compounds **A2**, **A5**, **A16**, **A20**, **A27**, **A33**, **B27** and **B33** (IC_50_ < 1 µM) were selected to screen their *in vitro* anticancer activity against A375 (human melanoma cell), MDA-MB-231 (human breast cancer cell), MGC-803 (human gastric cancer cell), SMMC-7721 (human hepatoma cell) and SGC-7901 (human gastric cancer cell) cell lines using MTT assay. Adriamycin (ADM) and BIBR1532 were used as the references^22^. The IC_50_ values were summarised in Table 3. In general, similar to the telomerase inhibitor BIBR1532, most of compounds possessed excellent telomerase inhibitory activity but no obvious antiproliferative activity against solid cancer cells (**A5**, **A16**, **A27**, **B27**, **B33**). However, many title compounds exhibited moderate antiproliferative activity on human melanoma A375 cells (**A2**, **A5**, **A16**, **A20**, **A33**), which may be due to the high expression of telomerase in human melanoma A375 cells^36^. Besides, what should be of most concern was that compound **A33** with styryl, which demonstrated moderately effective antiproliferative activity against all tested five cancer cell lines as compared to other compounds. The results suggest that compound **A33** may have different mechanisms from BIBR1532 in inhibiting telomerase activity, which supports that this compound deserves further study.

**Table 3.** Antiproliferative activity in *vitro* of compounds with strong telomerase inhibitory activity (IC_50_<1 μM) against A375, MDA-MB-231, MGC-803, SMMC-7721 and SGC-7901 cell lines^a^.

	IC_50_ (μM)^b^				
Compounds	A375	MDA-MB-231	MGC-803	SMMC-7721	SGC-7901
**A2**	11.03 ± 1.54	25.06 ± 2.10	17.26 ± 2.21	8.07 ± 1.30	56.91 ± 1.24
**A5**	20.09 ± 0.62	–^c^	15.22 ± 0.41	–	25.36 ± 0.59
**A16**	10.09 ± 0.52	–	–	–	–
**A20**	8.92 ± 0.69	19.50 ± 1.00	6.29 ± 0.36	–	10.22 ± 0.65
**A27**	–	–	–	–	–
**A33**	11.21 ± 0.69	9.89 ± 0.44	8.76 ± 0.25	9.67 ± 0.82	10.01 ± 0.51
**B27**	–	–	–	–	–
**B33**	–	–	–	–	–
**BIBR1532** ^d^	57.58 ± 0.21	–	–	–	–
**ADM** ^d^	0.58 ± 0.20	0.51 ± 0.12	0.42 ± 0.08	0.79 ± 0.13	0.82 ± 0.43

Negative control 0.1% DMSO, no activity.

^a^The data represented the mean of three experiments in triplicate and were expressed as means ± SD.

^b^The IC_50_ value was defined as the concentration at which 50% survival of cells was observed. The results are listed in the table.

^c^Not observed in the tested concentration range (>100 μM).

^d^Used as a positive control.

### 3.5. Assay of human normal cell

In order to determine the selective cytotoxicity of selected compounds, we subsequently conducted a proliferative inhibition assay with human normal liver cell (L-02). The results were summarised in Table 4. It was observed that the selected eight title compounds all showed lower cytotoxicity. In particular, compound **A33** manifested an obvious non-toxic effect on L-02, with IC_50_ of 2.21 mM. The data indicated that compound **A33** displayed excellent selectivity against tumour cells over the normal somatic cells. Moreover, this compound exhibited lower cytotoxicity than 1,3,4-oxadiazole derivatives reported previously^33^. Therefore, in combination with the above points, it is quite meaningful to further explore the mechanisms of this compound.

**Table 4.** Toxicity of compounds with strong telomerase inhibitory activity (IC_50_ < 1 μM) against human normal liver cells L-02^a^.

Compounds	L-02 (IC_50_, mM)
**A2**	1.37 ± 0.43
**A5**	1.99 ± 0.13
**A16**	0.90 ± 0.11
**A20**	0.62 ± 0.24
**A27**	1.38 ± 0.21
**A33**	2.21 ± 0.17
**B27**	1.67 ± 0.25
**B33**	1.01 ± 0.14

^a^MTT assays were used for evaluation, and values were expressed as mean IC_50_ of the triplicate experiment.

### 3.6. Cell cycle analysis

**Figure 3.**
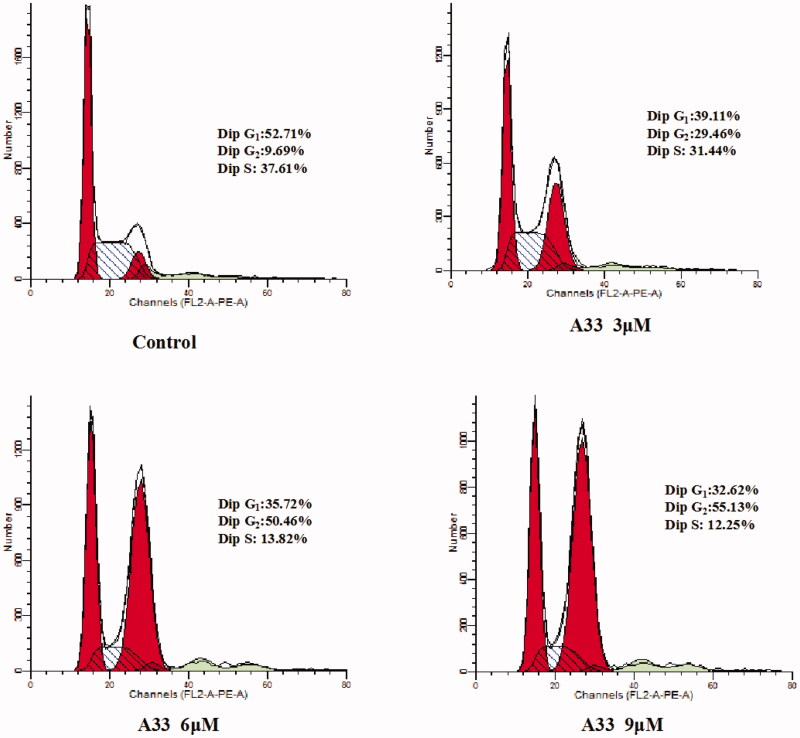
Cell cycle distribution induced by compound **A33** was measured in MGC-803 cells. Cells were treated with compound **A33** of 3, 6 and 9 μM for 48 h. Samples were analysed by flow cytometry and received results were analysed by modifit software.

The results of anticancer activity showed that compound **A33** could inhibit proliferation of MGC-803 cells. To verify whether cell cycle arrest leads to decrease cells proliferation, we used flow cytometric analysis to measure the effect of this compound on induction of cell cycle. As shown in Figure 3, treatment of MGC-803 cells with increasing concentrations (3, 6, 9 µM) of compound **A33** for 48 h, increased the G2/M phase distribution by 45.37% (from 9.76 to 55.13%), whereas the G0/G1 and S phase distribution decreased from 52.71 to 32.62% and from 37.61 to 12.25% in MGC-803 cells, respectively. In a word, this compound can induce cell cycle arrest at G2/M phase in a concentration-dependent manner, delaying cell cycle progression, thereby resulting in cell proliferation inhibition.

### 3.7. Cell apoptosis analysis

**Figure 4.**
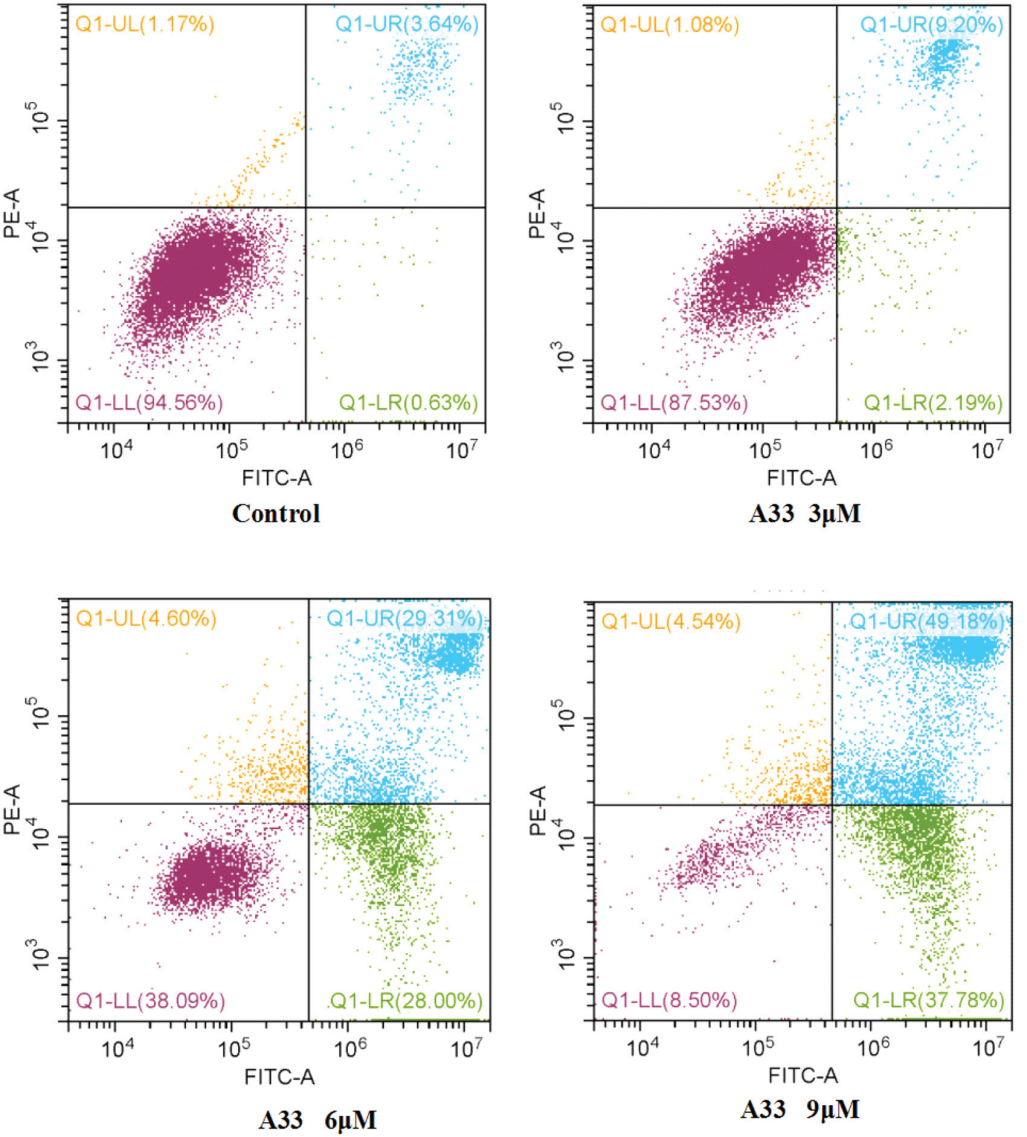
Percentage of apoptotic cells was determined in MGC-803 cells by Annexin-V FITC/PI staining. MGC-803 cells were treated with increasing concentrations of compound **A33** for 48 h and stained with Annexin-V FITC/PI. Apoptotic ratio increased, accompanied with the increase of concentration.

To determine whether compound **A33** meditated inhibition of proliferation was related with apoptosis, MGC-803 cells was selected for examination. The Annexin V-FITC/PI apoptosis detection kit was used in cell apoptosis analysis. As shown in Figure 4, the first quadrant usually represents damaged cells which was induced by mechanical forces, environmental stimulus and so on; the second quadrant generally denotes later period apoptotic cells and necrotic cells; the third quadrant often represents early apoptotic cells; and the fourth quadrant customarily denotes normal cells. The percentage of AnnexinV-FITC binding MGC-803 cells significantly increased from 4.27% to 11.39, 57.31 and 86.96%, respectively, after 48 h of treatment with increasing concentrations of compound **A33**. The results show that compound **A33** can induce apoptosis of MGC-803 cells in a concentration-dependent manner. This is consistent with the fact that telomerase inhibitors can induce apoptosis and thus inhibit the unlimited proliferation of tumour cells^37^.

### 3.8. Down-regulated expression of Dyskerin-NOP10-NHP2

**Figure 5.**
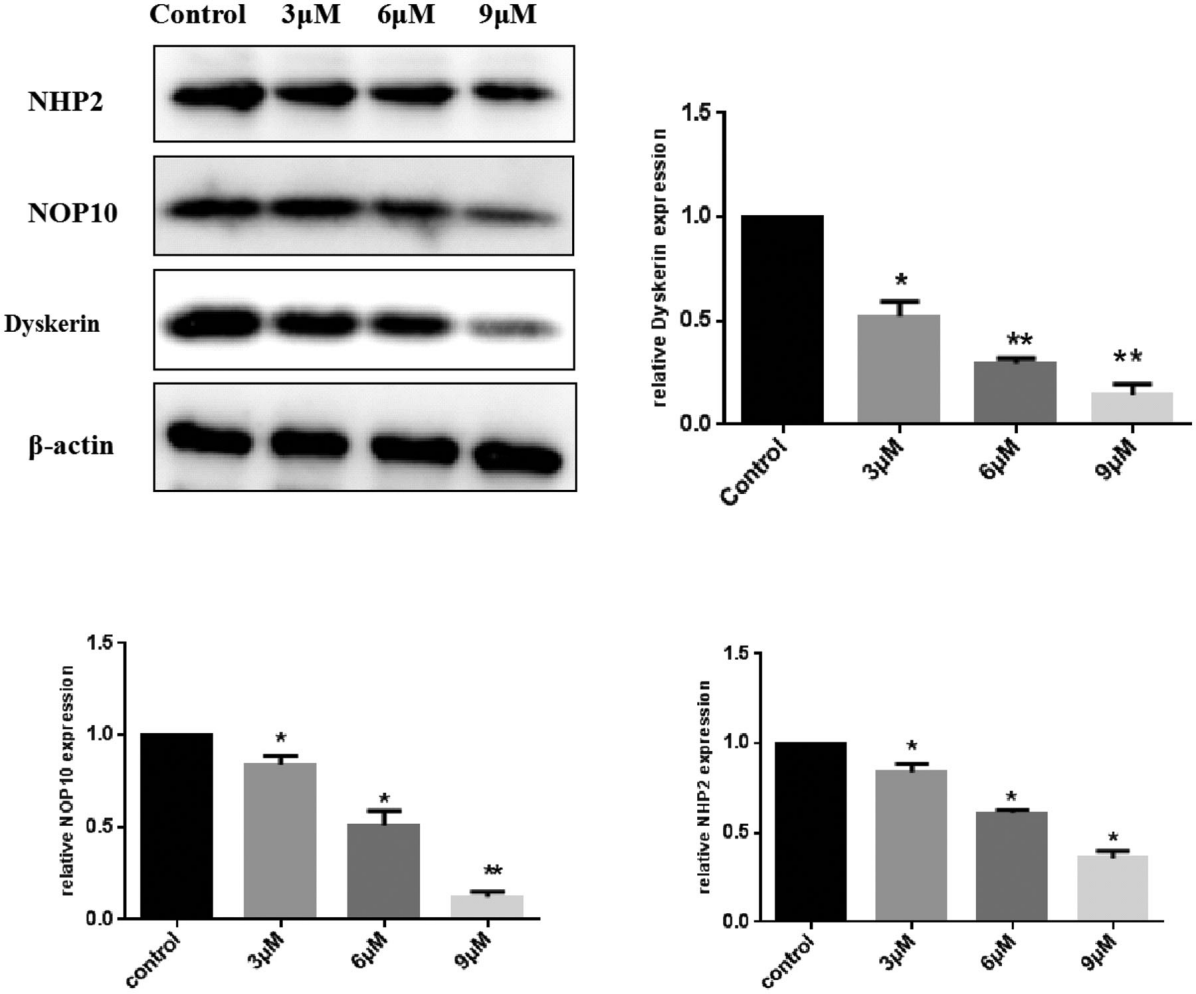
Compound **A33** inhibited Dyskerin expression in MGC-803 cells. MGC-803 cells were treated with compound **A33** of 3, 6 and 9 μM for 48 h. The proteins expression of Dyskerin, NOP10 and NHP2 were analysed by Western blotting. The results are expressed as relative expression against control expression. *n* = 3. Results are shown as mean ± SD from three independent experiments. *p < 0.05, **p < 0.01.

Dyskerin-NOP10-NHP2, trimer proteins are the core components of telomerase, playing a key role in the stabilisation, activation and assembly of telomerase, and the loss of dyskerin function can influence telomerase activity. Dyskerin over-expression associated with a variety of tumour types has been reported^38^. To test whether compound **A33** can modulate the expression of the trimer proteins, we used Western blotting. As shown in Figure 5, treatment with different concentrations (3, 6, 9 µM) of compound **A33** for 48 h (MGC-803 cells were selected), expression level of dyskerin protein was reduced in a concentration-dependent manner. Meanwhile, NHP2^39^ and NOP10^40^, as the important components of dyskerin-NHP2-NOP10 trimer, had also been assessed together. The results indicated that expressions of NOP10 and NHP2 were also lower level than control group. Therefore, compound **A33** may be an efficient dyskerin regulator.

## 4. Conclusions

With the aim to discover highly efficient telomerase inhibitors, upon extensive optimisation, a total of 66 2-phenyl-4H-chromone derivatives containing amide and 1,3,4-oxadiazole moieties were designed and synthesised. Most of the title compounds demonstrated potent telomerase inhibitory activity. SARs studies showed that the substitution of the methoxy group at **R^1^** was very advantageous for telomerase activity, and the substitution of halogen for the *para* position of the phenyl ring at **R^2^** significantly improved the telomerase inhibitory activity. However, replacing phenyl ring at **R^2^** with aromatic fused rings, aromatic heterocycles and other substituents had also the significant effect on telomerase activity. In particular, compound **A33** substituted by styryl at **R^2^** not only possessed strong activity against telomerase, but also exhibited moderately effective antiproliferative activity against all tested five human cancer cell lines, which was superior to telomerase inhibitor BIBR1532. Furthermore, it had no obvious toxicity towards human normal L-02 cell with IC_50_ of 2.21 mM. Flow cytometric analysis indicated that MGC-803 cell cycle was arrested in the G2/M phase by this compound, inducing MGC-803 cells apoptosis. Western blotting revealed that compound **A33** could significantly decrease the expression of dyskerin. In conclusion, it is believed that these results will help to regulate the expression of dyskerin protein through the rational design of small molecules in the future.

## Supplementary Material

Supplemental Material

## Supporting information

The following files are available free. ^1^H NMR and ^13 ^C NMR spectra and HRMS of all compounds. Fitting plot of compounds **A2**, **A5**, **A16**, **A20**, **A27**, **A33**, **B27** and **B33** for telomerase activity.

## References

[1] Saraswati AP, Relitti N, Brindisi M, et al. Raising the bar in anticancer therapy: recent advances in, and perspectives on, telomerase inhibitors. Drug Discov Today 2019;24:1370–88. 31136800 10.1016/j.drudis.2019.05.015

[2] Wright WE, Piatyszek MA, Rainey WE, et al. Telomerase activity in human germline and embryonic tissues and cells. Dev Genet 1996;18:173–9. 8934879 10.1002/(SICI)1520-6408(1996)18:2<173::AID-DVG10>3.0.CO;2-3

[3] Collins K, Mitchell JR. Telomerase in the human organism. Oncogene 2002;21:564–79. 11850781 10.1038/sj.onc.1205083

[4] Rocchi L, Barbosa AJM, Onofrillo C, et al. Inhibition of human dyskerin as a new approach to target ribosome biogenesis. PLoS One 2014;9:e101971. 25010840 10.1371/journal.pone.0101971 PMC4092089

[5] Sugarman ET, Zhang G, Shay JW. In perspective: An update on telomere targeting in cancer. Mol Carcinogen 2019;58:1581–8. 10.1002/mc.23035 PMC6692182 31062416

[6] Bhattacharya S, Chaudhuri P, Jain AK, et al. Symmetrical bisbenzimidazoles with benzenediyl spacer: the role of the shape of the ligand on the stabilization and structural alterations in telomeric G-quadruplex DNA and telomerase inhibition. Bioconjug Chem 2010;21:1148–59. 20536245 10.1021/bc9003298

[7] Zhou K, Liu JC, Xiong XQ, et al. Design, synthesis of 4,5-diazafluorene derivatives and their anticancer activity via targeting telomeric DNA G-quadruplex. Eur J Med Chem 2019;178:484–99. 31202994 10.1016/j.ejmech.2019.06.012

[8] Recagni M, Greco ML, Milelli A, et al. Distinct biological responses of metastatic castration resistant prostate cancer cells upon exposure to G-quadruplex interacting naphthalenediimide derivatives. Eur J Med Chem 2019;177:401–13. 31158753 10.1016/j.ejmech.2019.05.066

[9] Roy S, Ali A, Kamra M, et al. Specific stabilization of promoter G-quadruplex DNA by 2,6-disubstituted amidoanthracene-9,10-dione based dimeric distamycin analogues and their selective cancer cell cytotoxicity. Eur J Med Chem 2020;195:112202. 32302880 10.1016/j.ejmech.2020.112202

[10] Qin QP, Chen ZF, Shen WY, et al. Synthesis of a platinum(II) complex with 2-(4-methoxy-phenyl) imidazo [4,5-f]-[1,10] phenanthrolin and study of its antitumor activity. Eur J Med Chem 2015;89:77–87. 25462228 10.1016/j.ejmech.2014.10.019

[11] Chen ZF, Qin QP, Qin JL, et al. Stabilization of G-quadruplex DNA, inhibition of telomerase activity, and tumor cell apoptosis by organoplatinum(II) complexes with oxoisoaporphine. J Med Chem 2015;58:2159–79. 25650792 10.1021/jm5012484

[12] Ou TM, Lin J, Lu YJ, et al. Inhibition of cell proliferation by quindoline derivative (SYUIQ-05) through its preferential interaction with c-myc promoter G-quadruplex. J Med Chem 2011;54:5671–9. 21774525 10.1021/jm200062u

[13] Jiang Y, Chen AC, Kuang GT, et al. Design, synthesis and biological evaluation of 4-anilinoquinazoline derivatives as new c-myc G-quadruplex ligands. Eur J Med Chem 2016;122:264–79. 27372288 10.1016/j.ejmech.2016.06.040

[14] Ashbridge B, Orte A, Yeoman JA, et al. Single-Molecule Analysis of the Human Telomerase RNA.dyskerin interaction and the effect of dyskeratosis congenita mutations. Biochemistry-Us 2009;48:10858–65. 10.1021/bi901373e PMC2778356 19835419

[15] Turano M, Angrisani A, De Rosa M, et al. Real-time PCR quantification of human DKC1 expression in colorectal cancer. Acta Oncol 2008;47:1598–9. 18607840 10.1080/02841860801898616

[16] Sieron P, Hader C, Hatina J, et al. DKC1 overexpression associated with prostate cancer progression. Br J Cancer 2009;101:1410–6. 19755982 10.1038/sj.bjc.6605299 PMC2768451

[17] Liu B, Zhang J, Huang C, et al. Dyskerin overexpression in human hepatocellular carcinoma is associated with advanced clinical stage and poor patient prognosis. Plos One 2012;7:e43147. 22912812 10.1371/journal.pone.0043147 PMC3418259

[18] Arndt GM, MacKenzie KL. New prospects for targeting telomerase beyond the telomere. Nat Rev Cancer 2016;16:508–24. 27339602 10.1038/nrc.2016.55

[19] Naasani I, Oh-hashi F, Oh-hara T, et al. Blocking telomerase by dietary polyphenols is a major mechanism for limiting the growth of human cancer cells in vitro and in *vivo*. Cancer Res 2003;63:824–30. 12591733

[20] Menichincheri M, Ballinari D, Bargiotti A, et al. Catecholic flavonoids acting as telomerase inhibitors. J Med Chem 2004;47:6466–75. 15588081 10.1021/jm040810b

[21] Rao YK, Kao TY, Wu MF, et al. Identification of small molecule inhibitors of telomerase activity through transcriptional regulation of hTERT and calcium induction pathway in human lung adenocarcinoma A549 cells. Bioorg Med Chem 2010;18:6987–94. 20813535 10.1016/j.bmc.2010.08.021

[22] Xue W, Song BA, Zhao HJ, et al. Novel myricetin derivatives: design, synthesis and anticancer activity. Eur J Med Chem 2015;97:155–63. 25965778 10.1016/j.ejmech.2015.04.063

[23] Wang JQ, Yang MD, Chen X, et al. Discovery of new chromen-4-one derivatives as telomerase inhibitors through regulating expression of dyskerin. J Enzyme Inhib Med Chem 2018;33:1199–211. 30132373 10.1080/14756366.2018.1466881 PMC6104605

[24] Fan ZF, Ho ST, Wen R, et al. Design, synthesis and molecular docking analysis of flavonoid derivatives as potential telomerase inhibitors. Molecules 2019;24:3180. 10.3390/molecules24173180 PMC6749477 31480619

[25] Li Z, Zhan P, Liu X. 1,3,4-Oxadiazole: a privileged structure in antiviral agents. Mini Rev Med Chem 2011;11:1130–42. 22353222 10.2174/138955711797655407

[26] Chawla G, Naaz B, Siddiqui AA. Exploring 1,3,4-oxadiazole scaffold for anti-inflammatory and analgesic activities: a review of literature from 2005-2016. Mini Rev Med Chem 2018;18:216–33. 28137242 10.2174/1389557517666170127121215

[27] Glomb T, Szymankiewicz K, Świątek P. Anti-cancer activity of derivatives of 1,3,4-oxadiazole. Molecules 2018;23:3361. 10.3390/molecules23123361 PMC6320996 30567416

[28] Verma G, Khan MF, Akhtar W, et al. Shaquiquzzaman, a review exploring therapeutic worth of 1,3,4-oxadiazole tailored compounds. Mini Rev Med Chem 2019;19:477–509. 30324877 10.2174/1389557518666181015152433

[29] Guimaraes CRW, Boger DL, Jorgensen WL. Elucidation of fatty acid amide hydrolase inhibition by potent alpha-ketoheterocycle derivatives from Monte Carlo simulations. J Am Chem Soc 2005;127:17377–84. 16332087 10.1021/ja055438j

[30] Zheng QZ, Zhang XM, Xu Y, et al. Synthesis, biological evaluation, and molecular docking studies of 2-chloropyridine derivatives possessing 1,3,4-oxadiazole moiety as potential antitumor agents. Bioorg Med Chem 2010;18:7836–41. 20947362 10.1016/j.bmc.2010.09.051

[31] Zhang XM, Qiu M, Sun J, et al. Synthesis, biological evaluation, and molecular docking studies of 1,3,4-oxadiazole derivatives possessing 1,4-benzodioxan moiety as potential anticancer agents. Bioorg Med Chem 2011;19:6518–24. 21962523 10.1016/j.bmc.2011.08.013

[32] Sun J, Zhu H, Yang ZM, et al. Synthesis, molecular modeling and biological evaluation of 2-aminomethyl-5-(quinolin-2-yl)-1,3,4-oxadiazole-2(3H)-thione quinolone derivatives as novel anticancer agent. Eur J Med Chem 2013;60:23–8. 23279864 10.1016/j.ejmech.2012.11.039

[33] Zhang F, Wang XL, Shi J, et al. Synthesis, molecular modeling and biological evaluation of *N*-benzylidene-2-((5-(pyridin-4-yl)-1,3,4-oxadiazol-2-yl)thio)acetohydrazide derivatives as potential anticancer agents. Bioorg Med Chem 2014;22:468–77. 24286761 10.1016/j.bmc.2013.11.004

[34] Han Y, Ding Y, Xie DD, et al. Design, synthesis, and antiviral activity of novel rutin derivatives containing 1, 4-pentadien-3-one moiety. Eur J Med Chem 2015;92:732–7. 25618020 10.1016/j.ejmech.2015.01.017

[35] Zhang C, Jiang SC, Chen Y, et al. Synthesis and biological of novel myricetin derivatives containing 1,3,4-oxadiazoles. Chinese J Org Chem 2019;39:1160–8.

[36] El-Daly H, Kull M, Zimmermann S, et al. Selective cytotoxicity and telomere damage in leukemia cells using the telomerase inhibitor BIBR1532. Blood 2005;105:1742–9. 15507522 10.1182/blood-2003-12-4322

[37] Ameri Z, Ghiasi S, Farsinejad A, et al. Telomerase inhibitor MST-312 induces apoptosis of multiple myeloma cells and down-regulation of anti-apoptotic, proliferative and inflammatory genes. Life Sci 2019;228:66–71. 31029779 10.1016/j.lfs.2019.04.060

[38] Vasuri F, Rocchi L, Degiovanni A, et al. Dyskerin expression in human fetal, adult and neoplastic intrahepatic bile ducts: correlations with cholangiocarcinoma aggressiveness. Histopathology 2015;66:244–51. 25367684 10.1111/his.12480

[39] Vulliamy T, Beswick R, Kirwan M, et al. Mutations in the telomerase component NHP2 cause the premature ageing syndrome dyskeratosis congenita. Proc Natl Acad Sci USA 2008;105:8073–8. 18523010 10.1073/pnas.0800042105 PMC2430361

[40] Walne AJ, Vulliamy T, Marrone A, et al. Genetic heterogeneity in autosomal recessive dyskeratosis congenita with one subtype due to mutations in the telomerase-associated protein NOP10. Hum Mol Genet 2007;16:1619–29. 17507419 10.1093/hmg/ddm111 PMC2882227

