# Supplementary material for: Synthesis, telomerase inhibitory and anticancer activity of new 2-phenyl-4H-chromone derivatives containing 1,3,4-oxadiazole moiety
Source: J Enzyme Inhib Med Chem. 2020 Dec 27;36(1):345–61. doi: 10.1080/14756366.2020.1864630 (PMC7782168; doi:10.1080/14756366.2020.1864630)
Supplement: Supplemental Material [file IENZ_A_1864630_SM6611.pdf]

Synthesis, telomerase inhibitory and anticancer activity of new 2-phenyl-4H-chromone derivatives containing 1,3,4-oxadiazole moiety

Content

1. The spectrum of ^{1}H NMR, ^{13}C NMR and HRMS of compounds A1-A33 and B1-B33.. S1-S96

2. Fitting plot of compounds A2, A5, A16, A20, A27, A33, B27 and B33 for telomerase activity...S97-S100

1. The spectrum of ^{1}H NMR, ^{13}C NMR and HRMS of compounds A1-A33 and B1-B33

A1

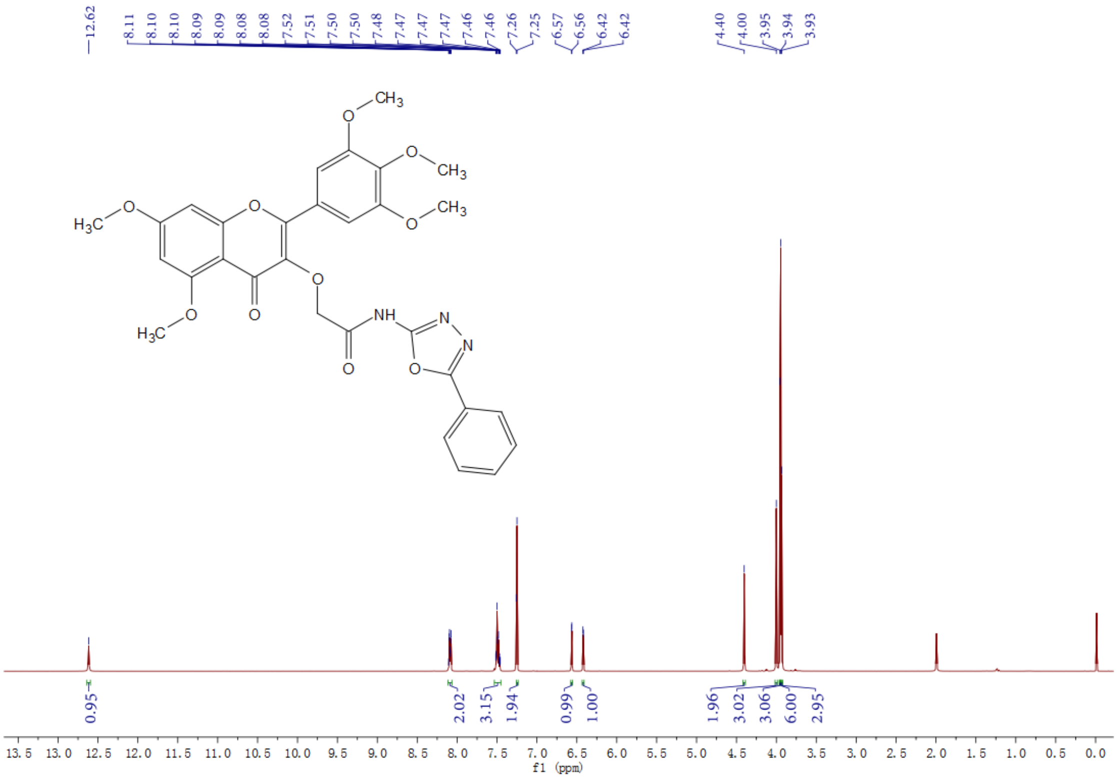

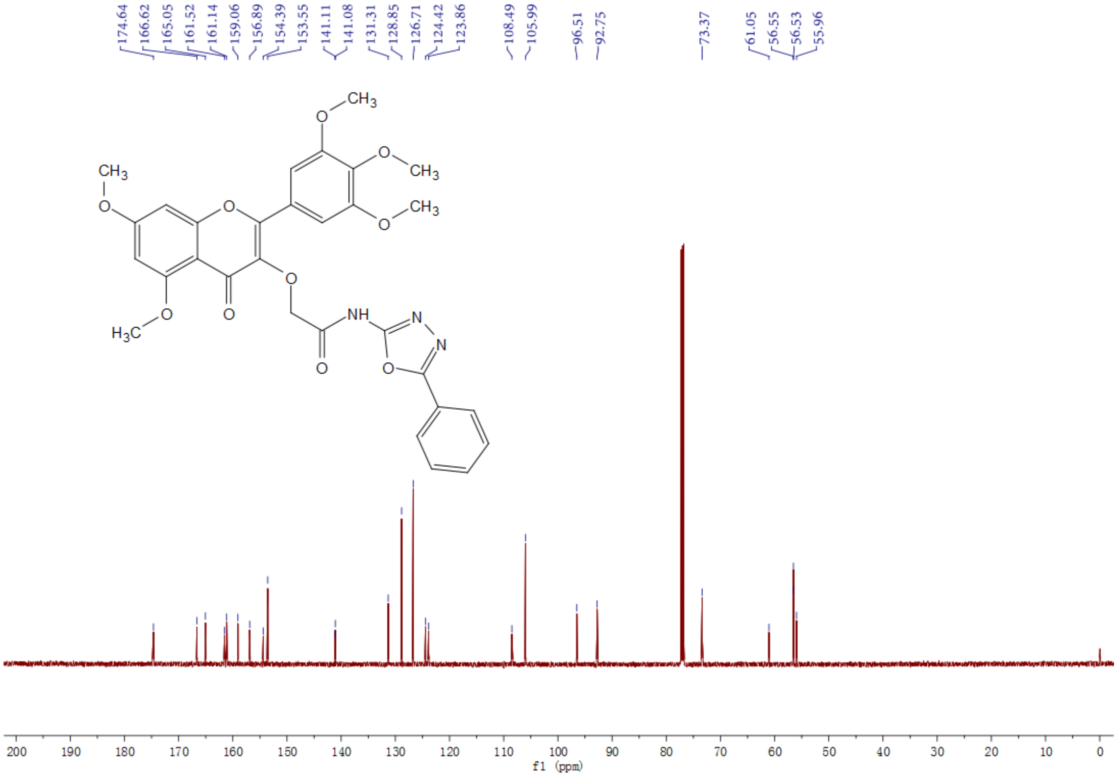

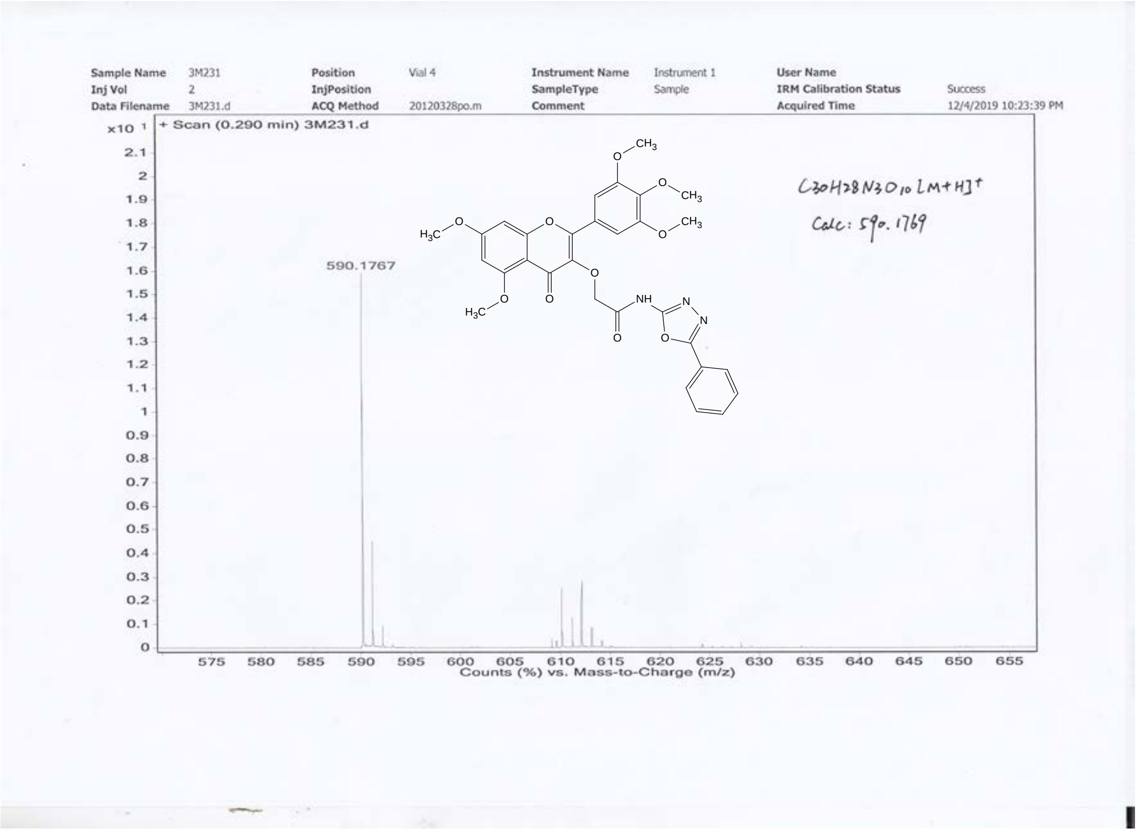

The spectrum of ^{1}H NMR, ^{13}C NMR and HRMS of compound A1

A2

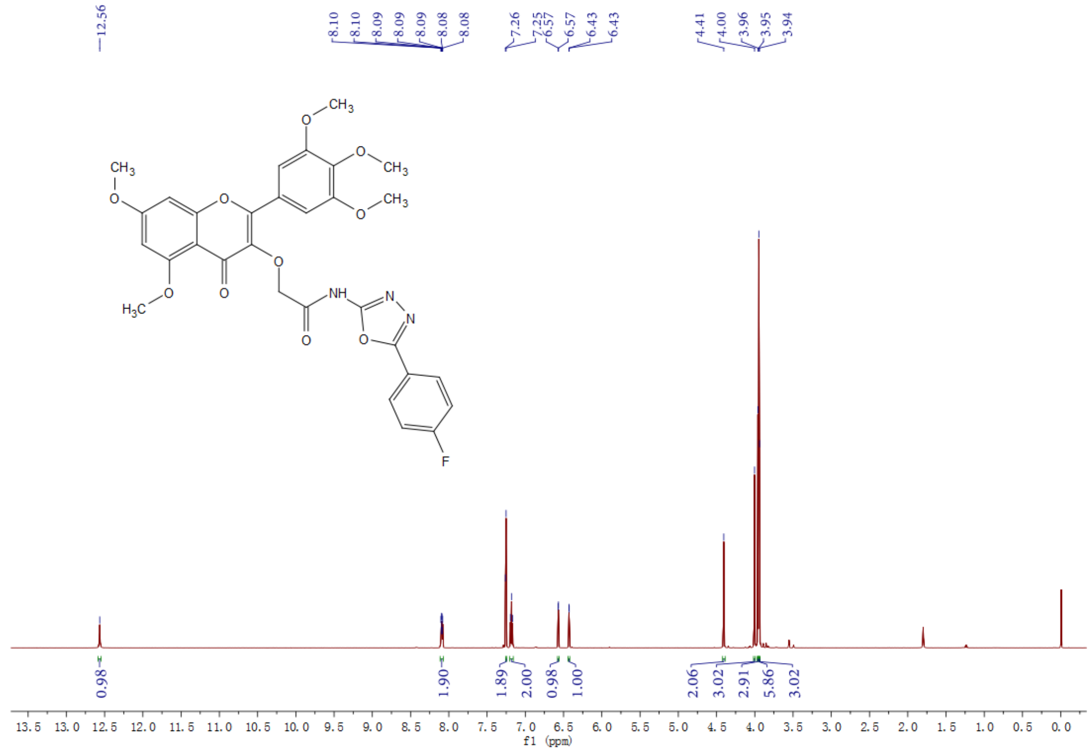

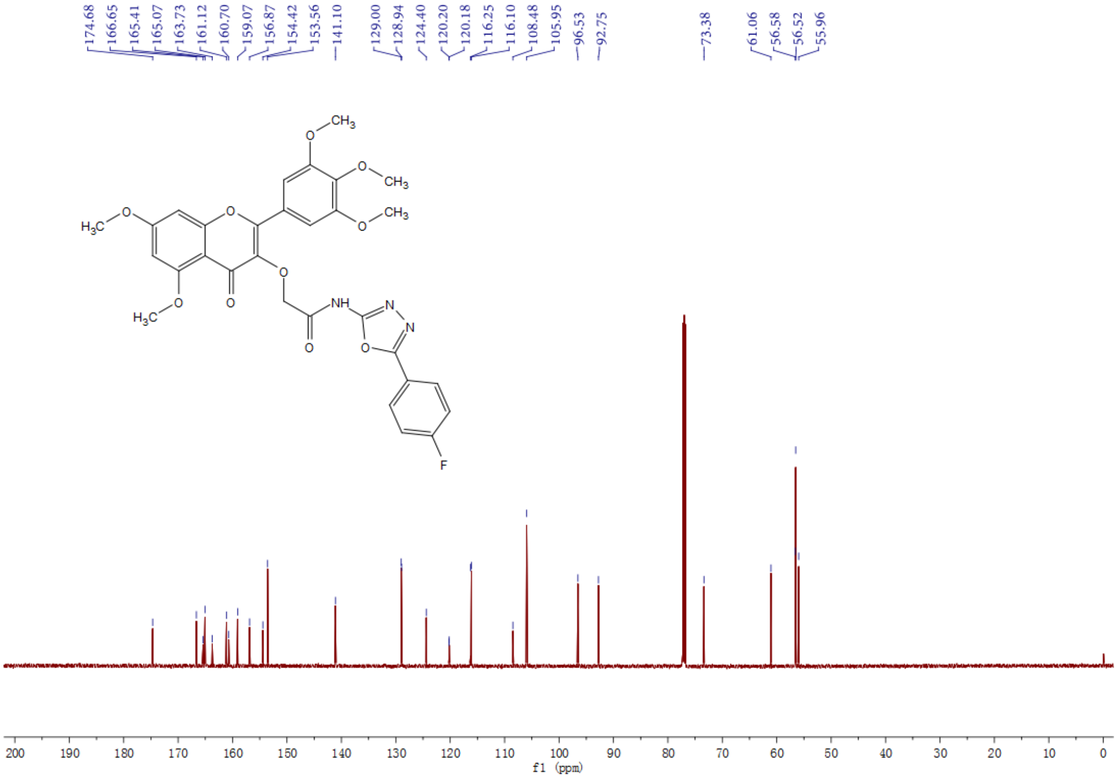

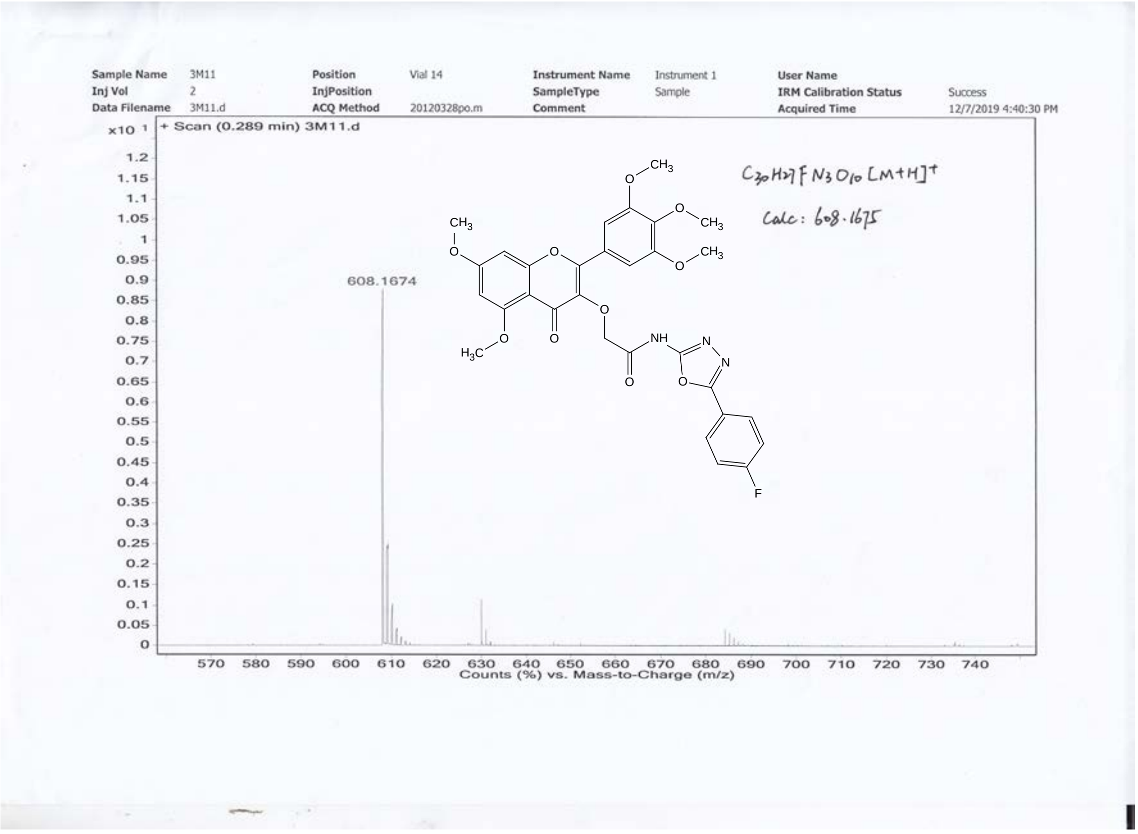

The spectrum of ^{1}H NMR, ^{13}C NMR and HRMS of compound A2

A3

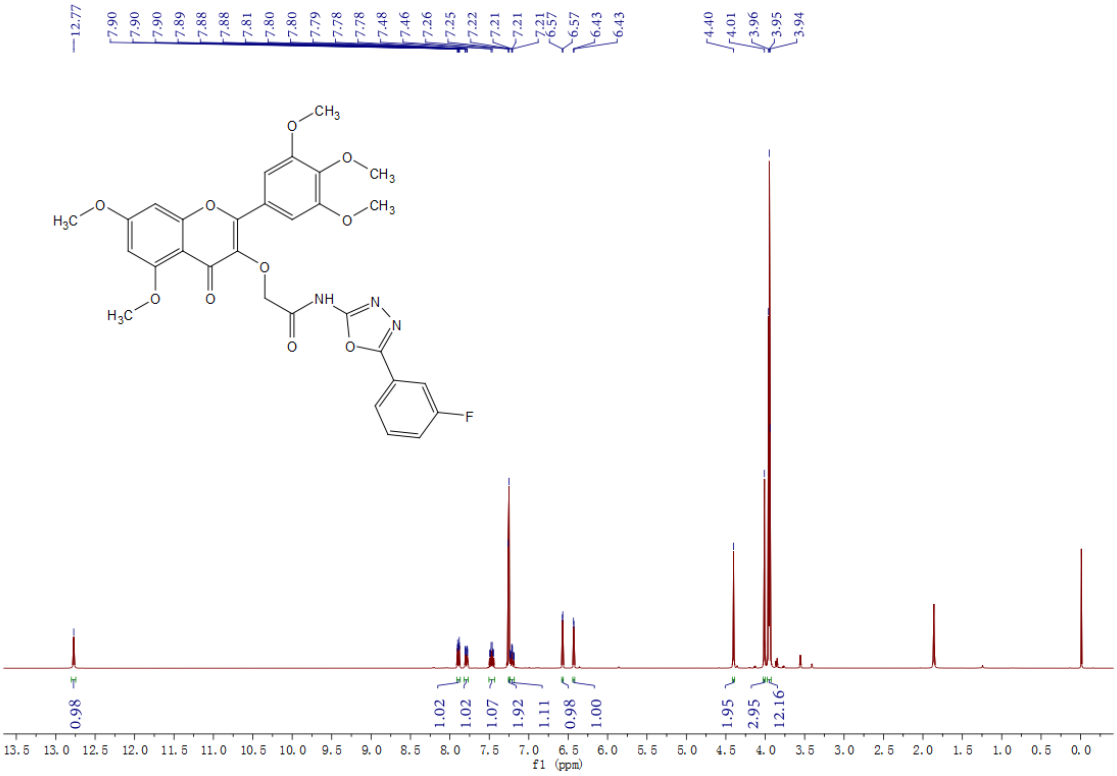
12.77
7.90 7.90 7.90 7.89 7.88 7.88 7.81 7.80 7.80 7.79 7.78 7.78 7.48 7.46 7.26 7.25 7.22 7.21 7.21 7.21 6.57 6.57 6.43 6.43
4.40 4.01 3.96 3.95 3.94
CH3
O
CH3
CH3
H3C
NH
N
N
F
0.98 1.02 1.02 1.07 1.92 1.11 0.98 1.00 1.95 2.95 12.16
13.5 13.0 12.5 12.0 11.5 11.0 10.5 10.0 9.5 9.0 8.5 8.0 7.5 7.0 6.5 6.0 5.5 5.0 4.5 4.0 3.5 3.0 2.5 2.0 1.5 1.0 0.5 0.0
f1 (ppm)

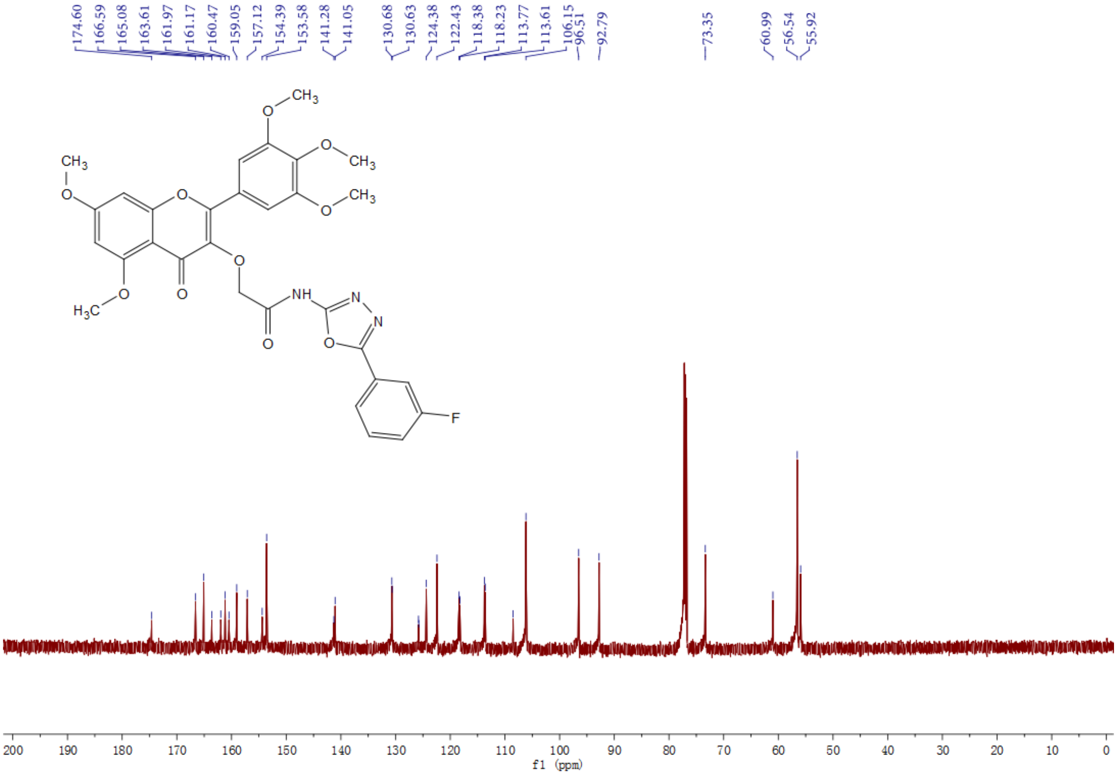
174.60 166.59 165.08 163.61 161.97 161.17 160.47 159.05 157.12 154.39 153.58 141.28 141.05
130.68 130.63 124.38 122.43 118.38 118.23 113.77 113.61 106.15 96.51 92.79
73.35
60.99 56.54 55.92
CH3
O
H3C
NH
N
N
F
200 190 180 170 160 150 140 130 120 110 100 90 80 70 60 50 40 30 20 10 0
f1 (ppm)

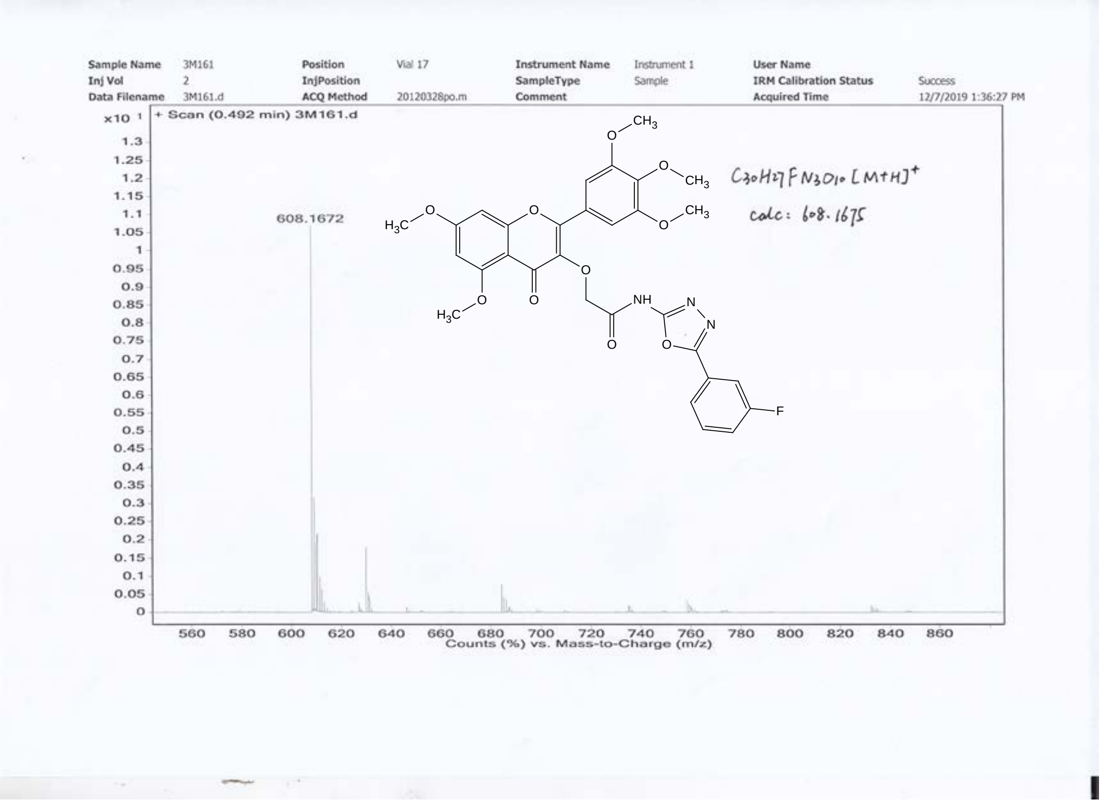

The spectrum of ^{1}H NMR, ^{13}C NMR and HRMS of compound A3

A4

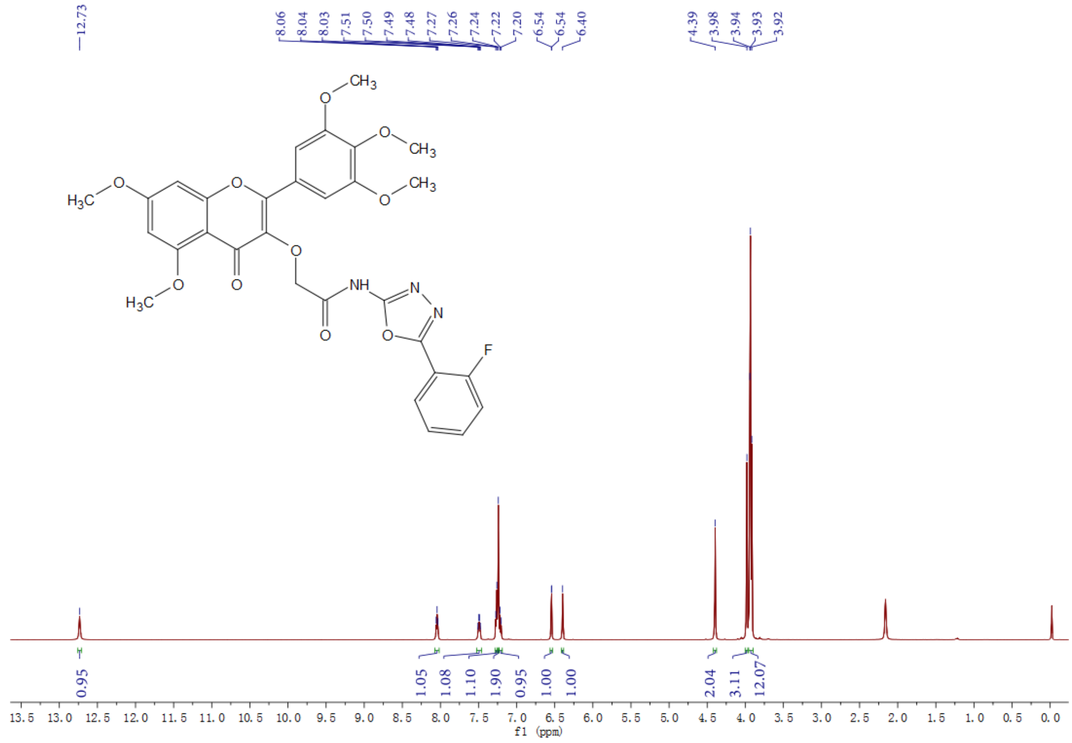

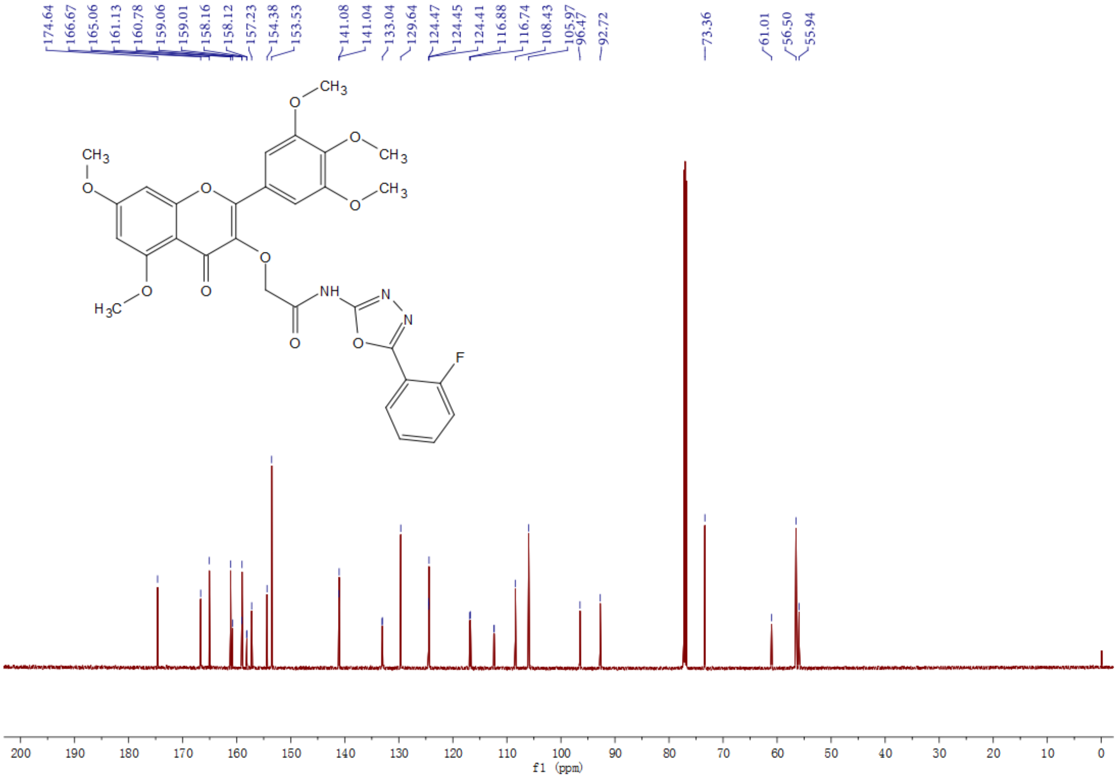

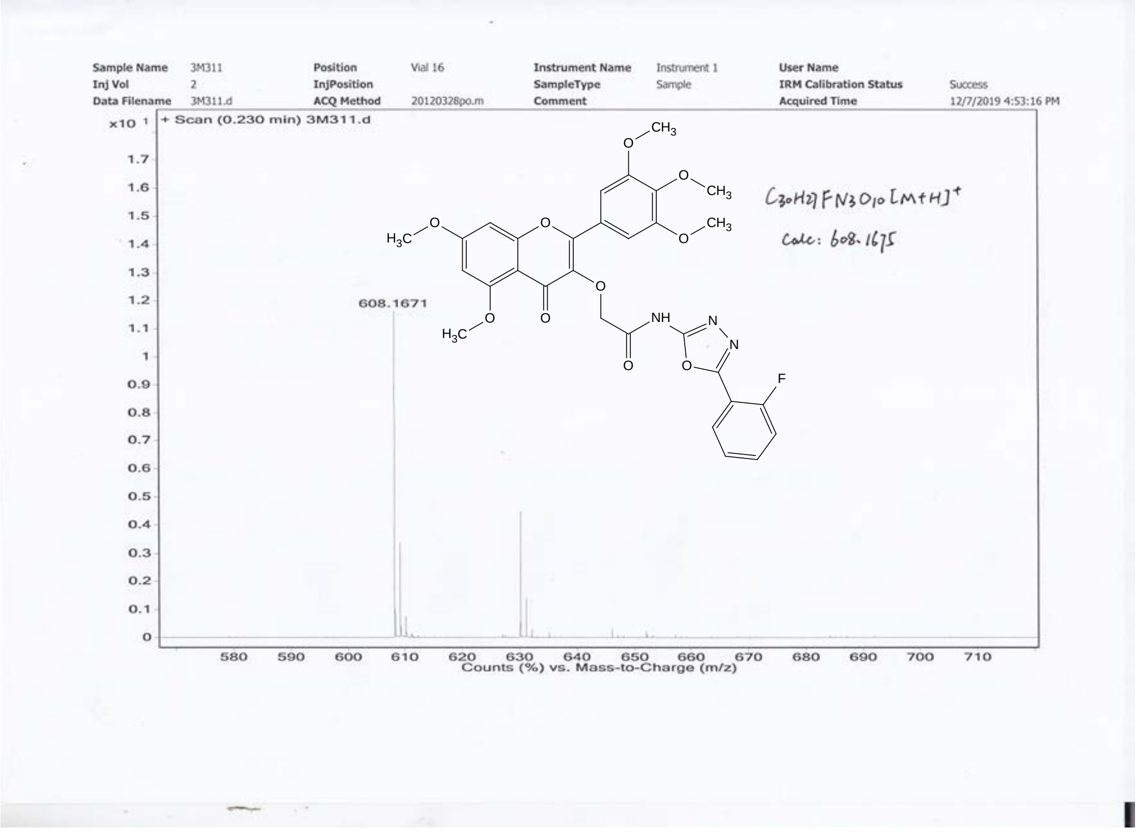

The spectrum of ^{1}H NMR, ^{13}C NMR and HRMS of compound A4

A5

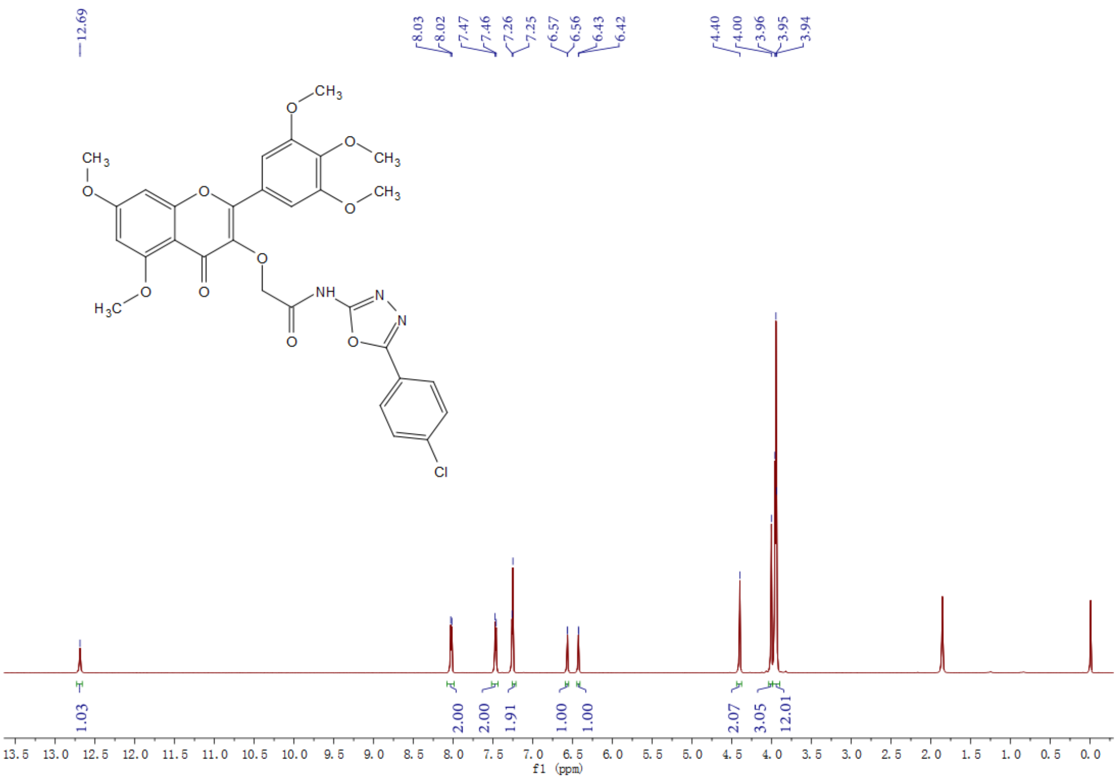

12.69
8.03
8.02
7.47
7.46
7.26
7.25
6.57
6.56
6.43
6.42
4.40
4.00
3.96
3.95
3.94
CH3
O
NH
N
Cl
1.03
2.00
2.00
1.91
1.00
1.00
2.07
3.05
12.01
f1 (ppm)

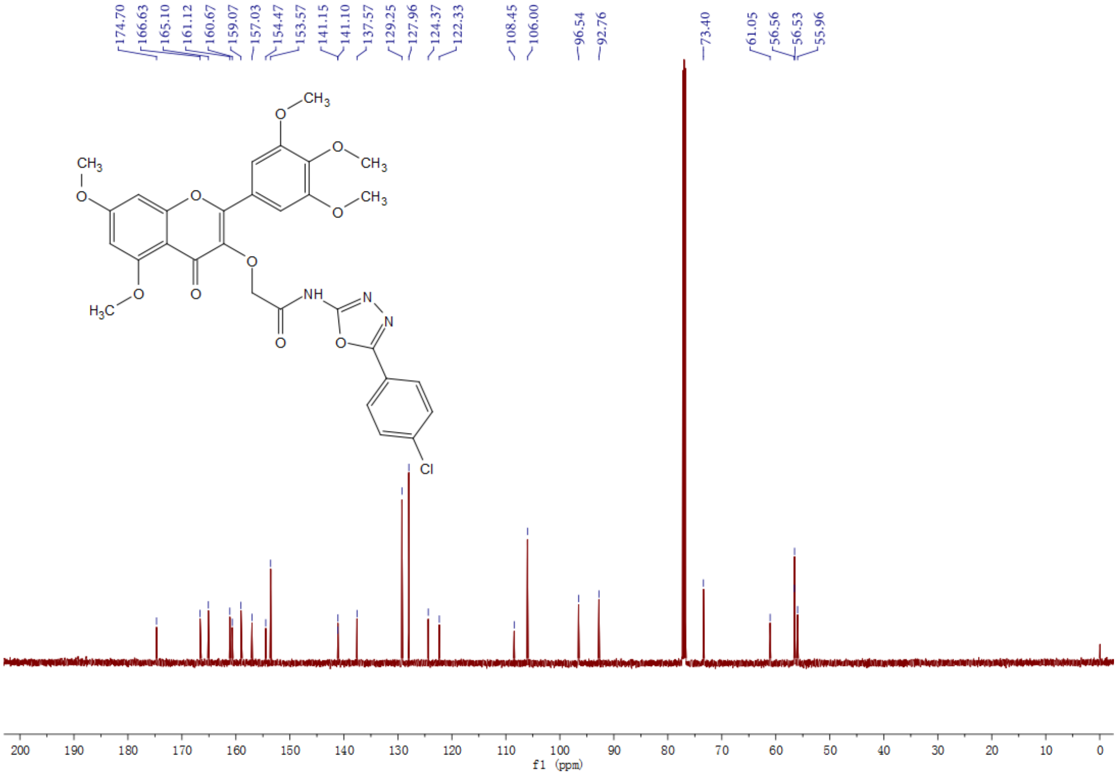

174.70
166.63
165.10
161.12
160.67
159.07
157.03
154.47
153.57
141.15
141.10
137.57
129.25
127.96
124.37
122.33
108.45
106.00
96.54
92.76
73.40
61.05
56.56
56.53
55.96
f1 (ppm)

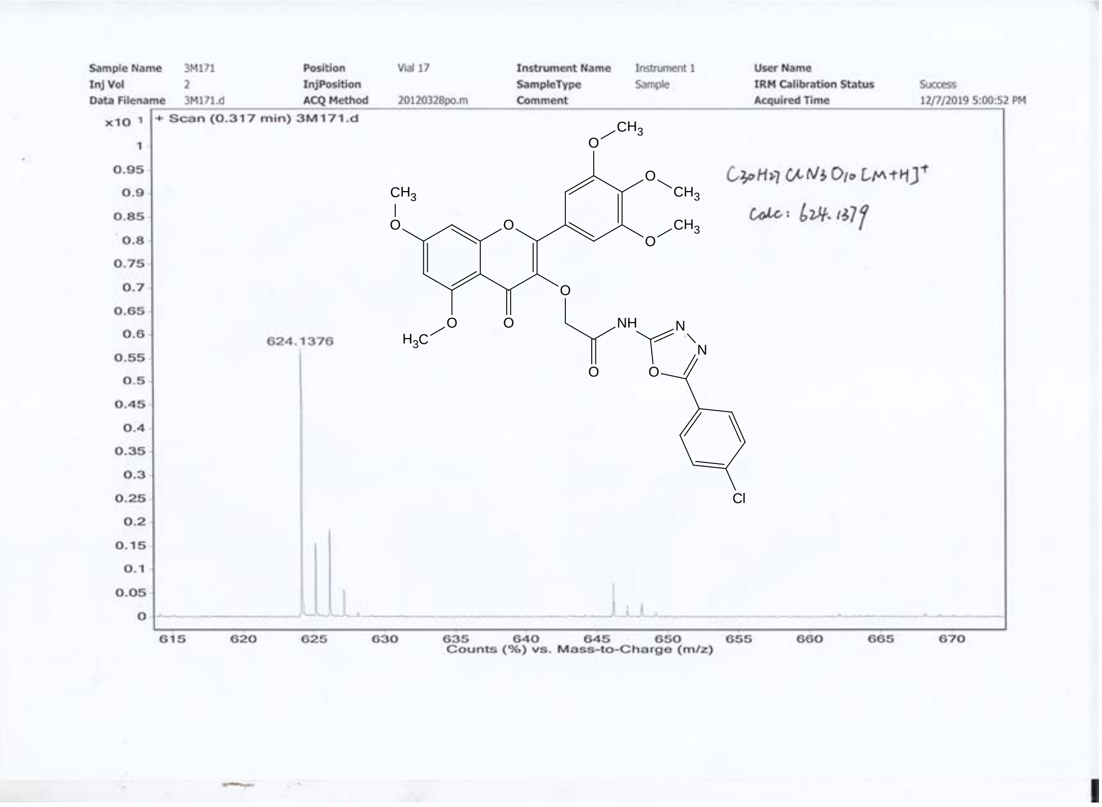

The spectrum of ^{1}H NMR, ^{13}C NMR and HRMS of compound A5

A6

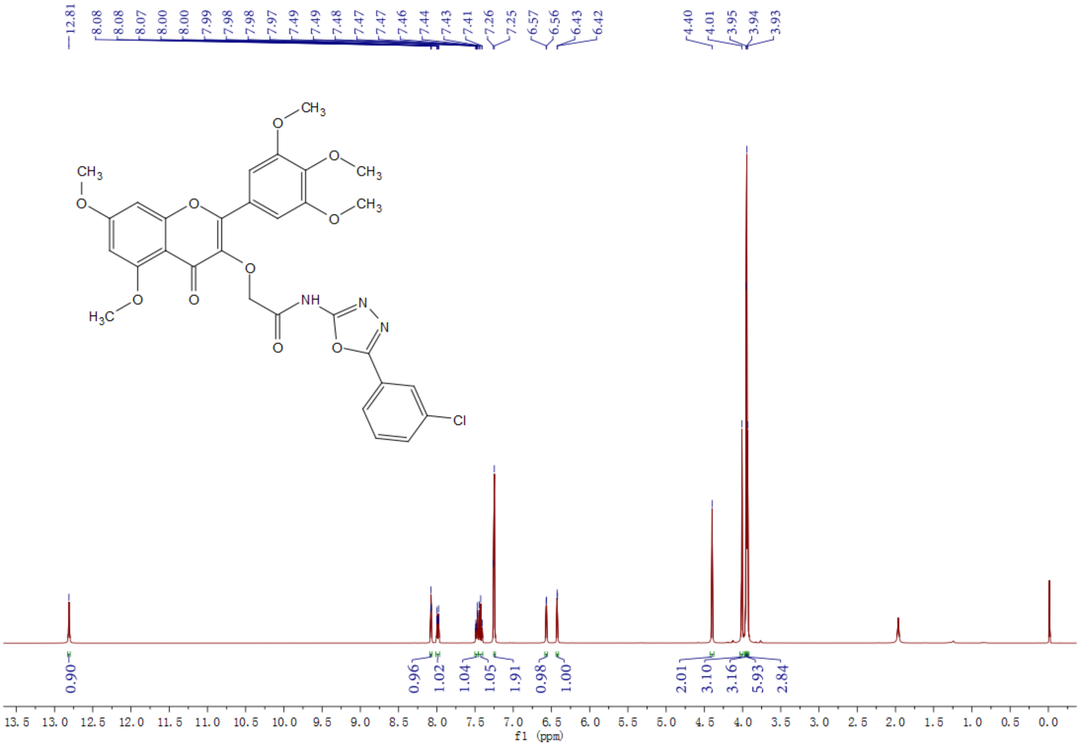

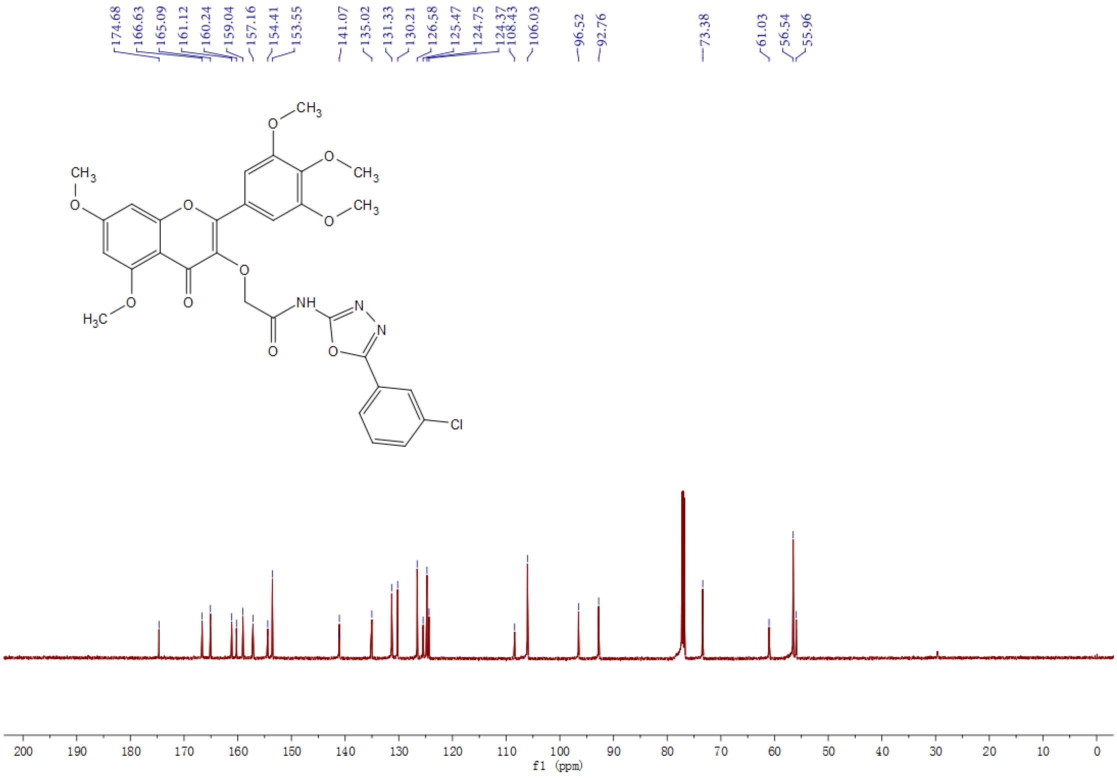

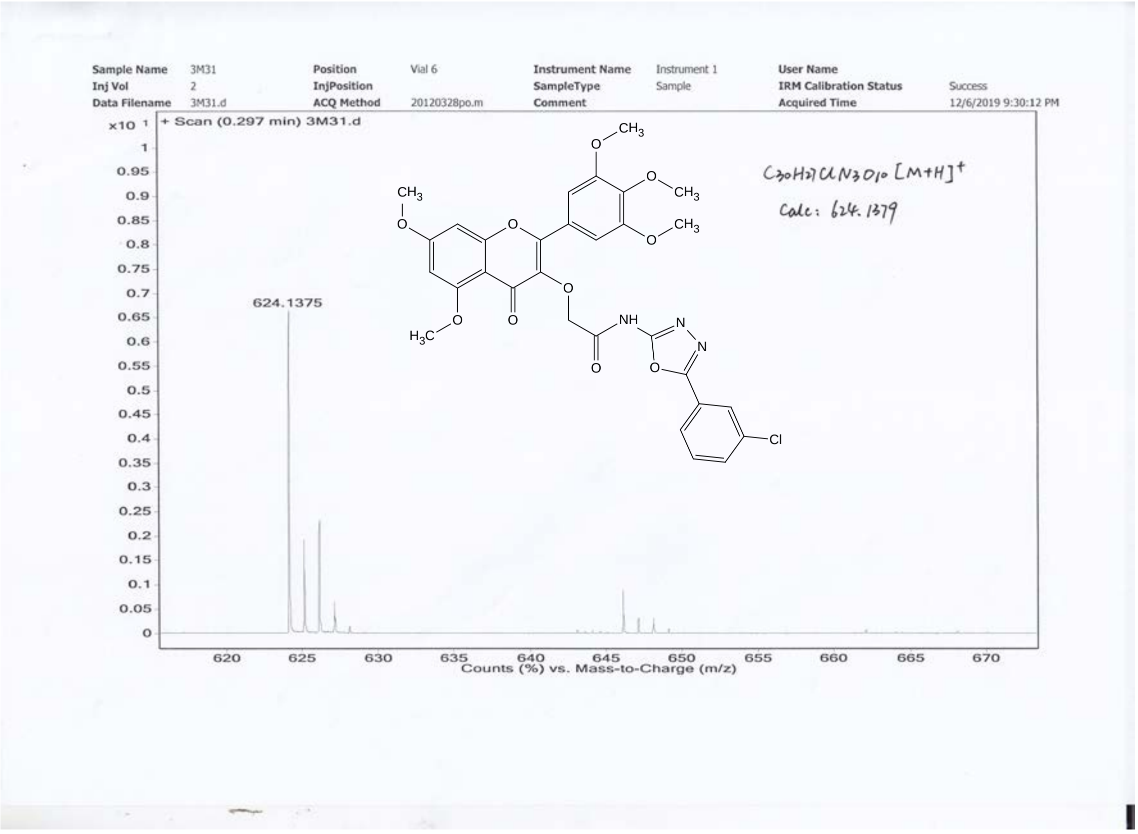

The spectrum of ^{1}H NMR, ^{13}C NMR and HRMS of compound A6

A7

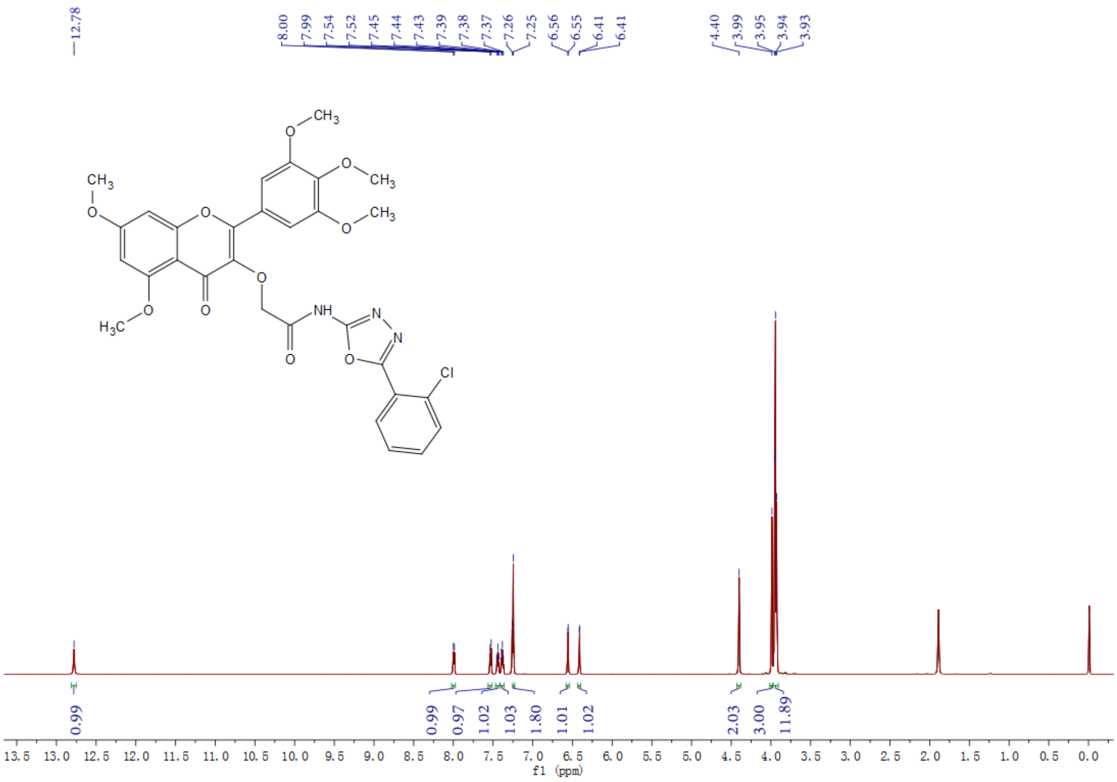

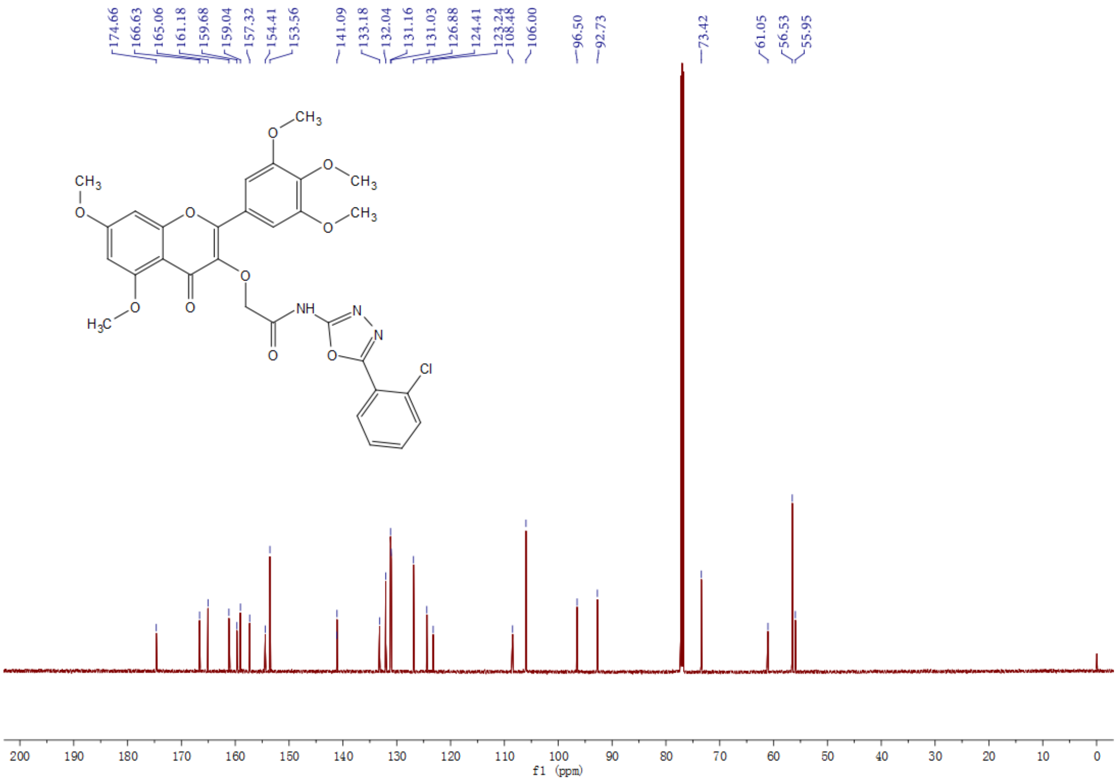

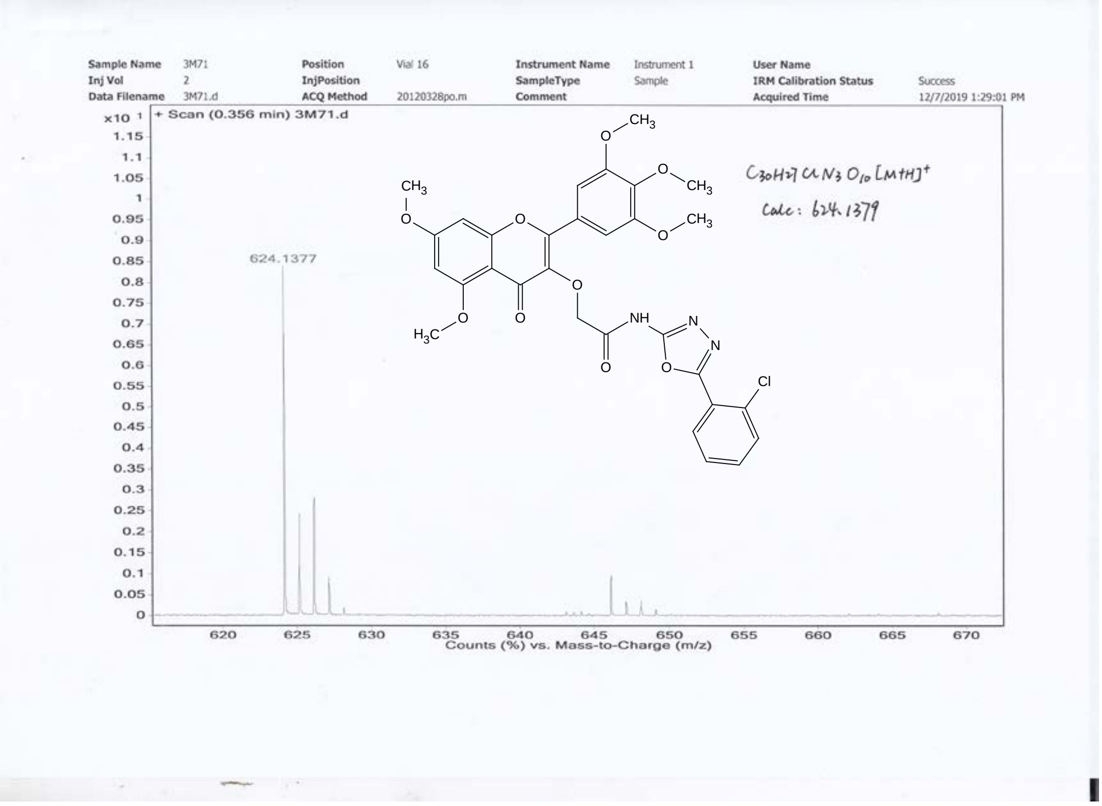

The spectrum of ^{1}H NMR, ^{13}C NMR and HRMS of compound A7

A8

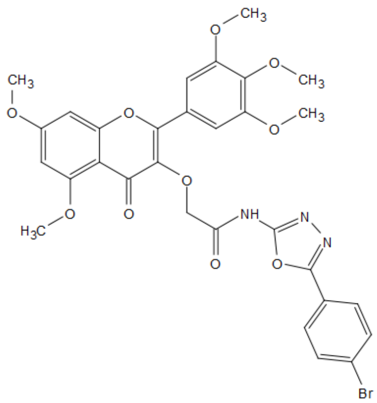

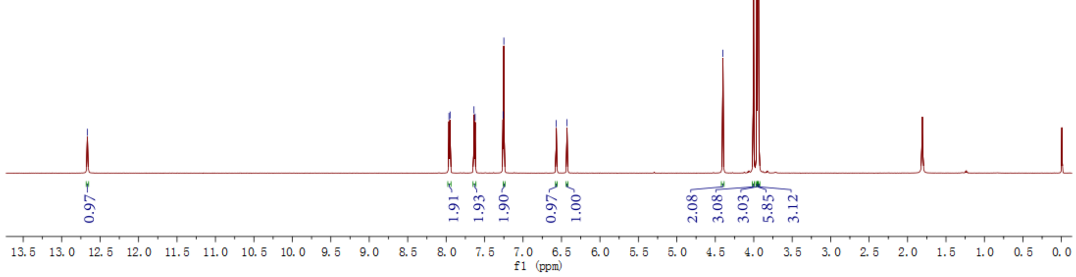

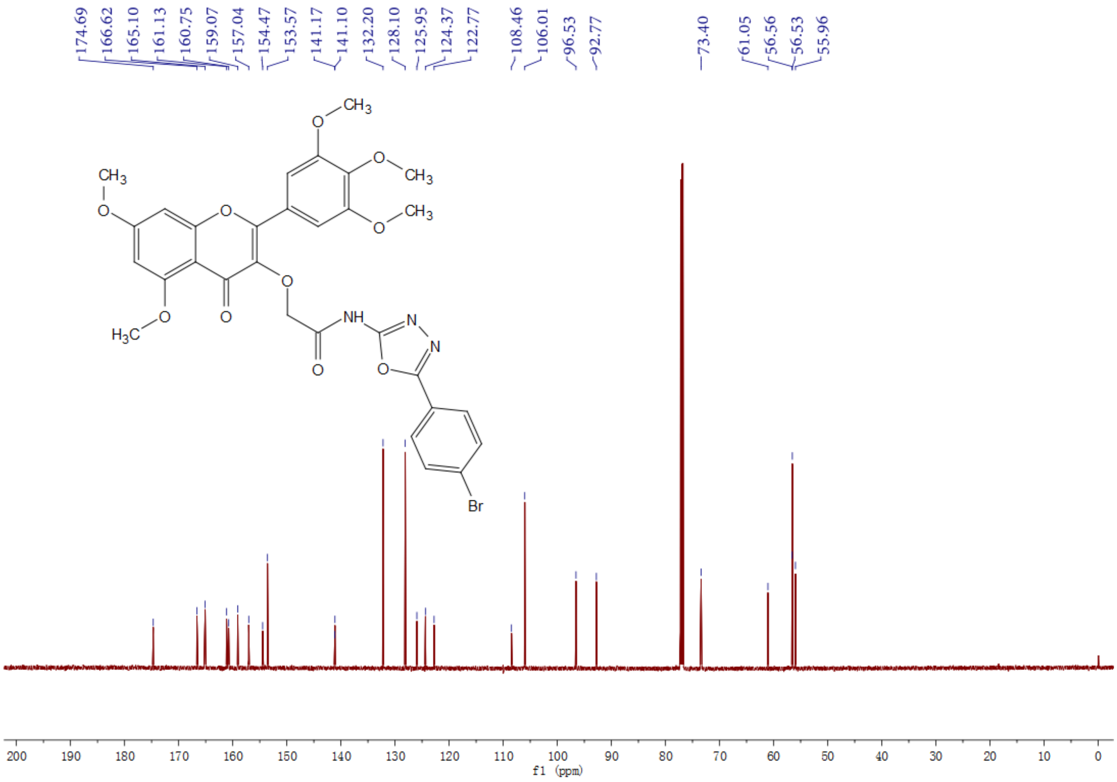

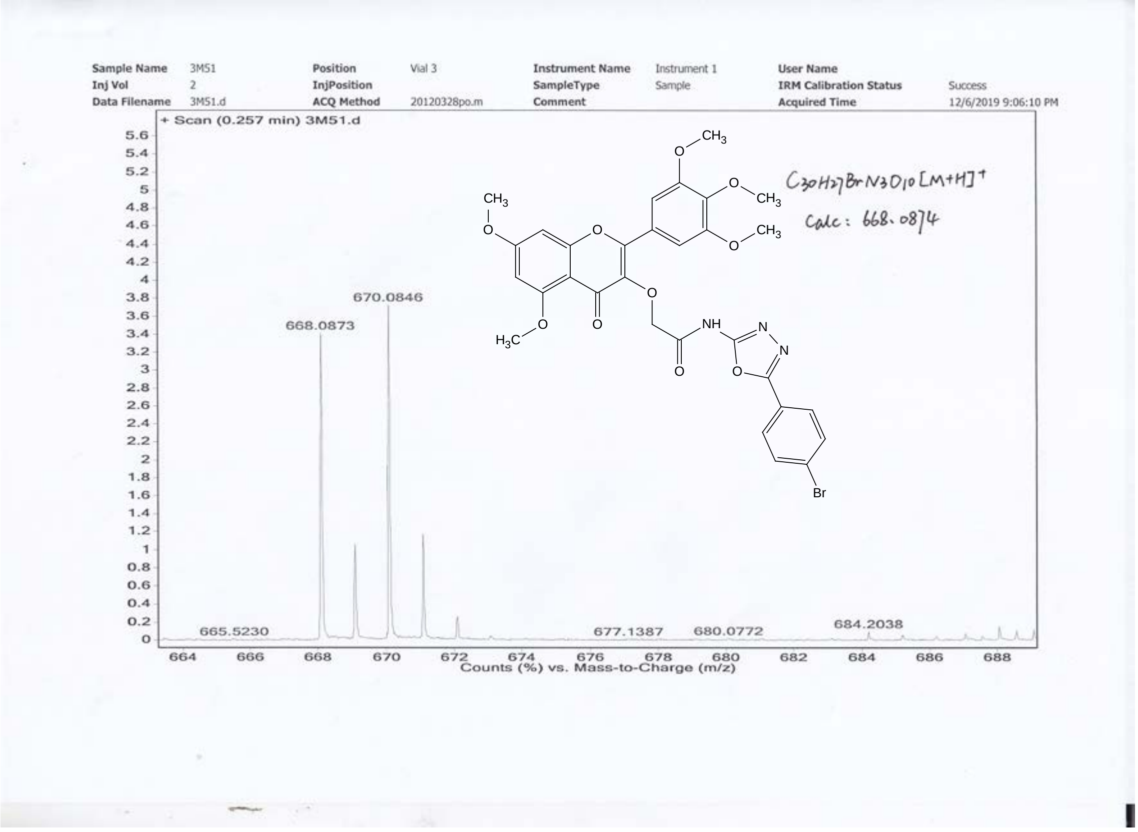

The spectrum of ^{1}H NMR, ^{13}C NMR and HRMS of compound A8

A9

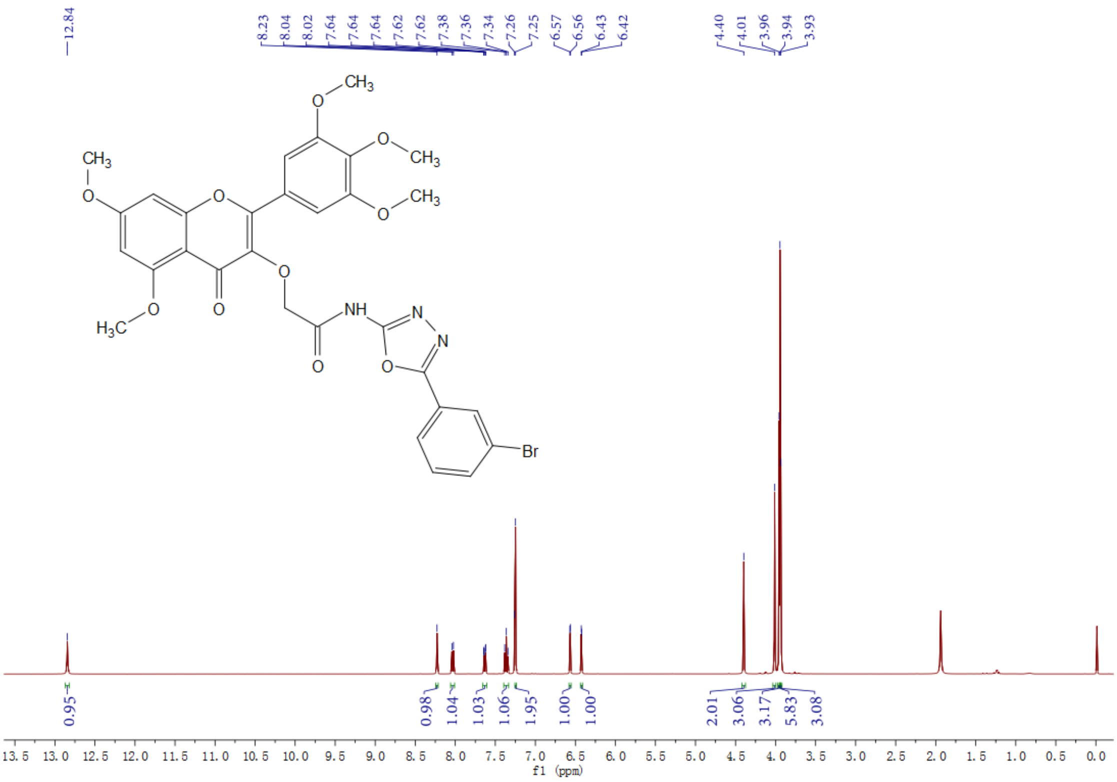

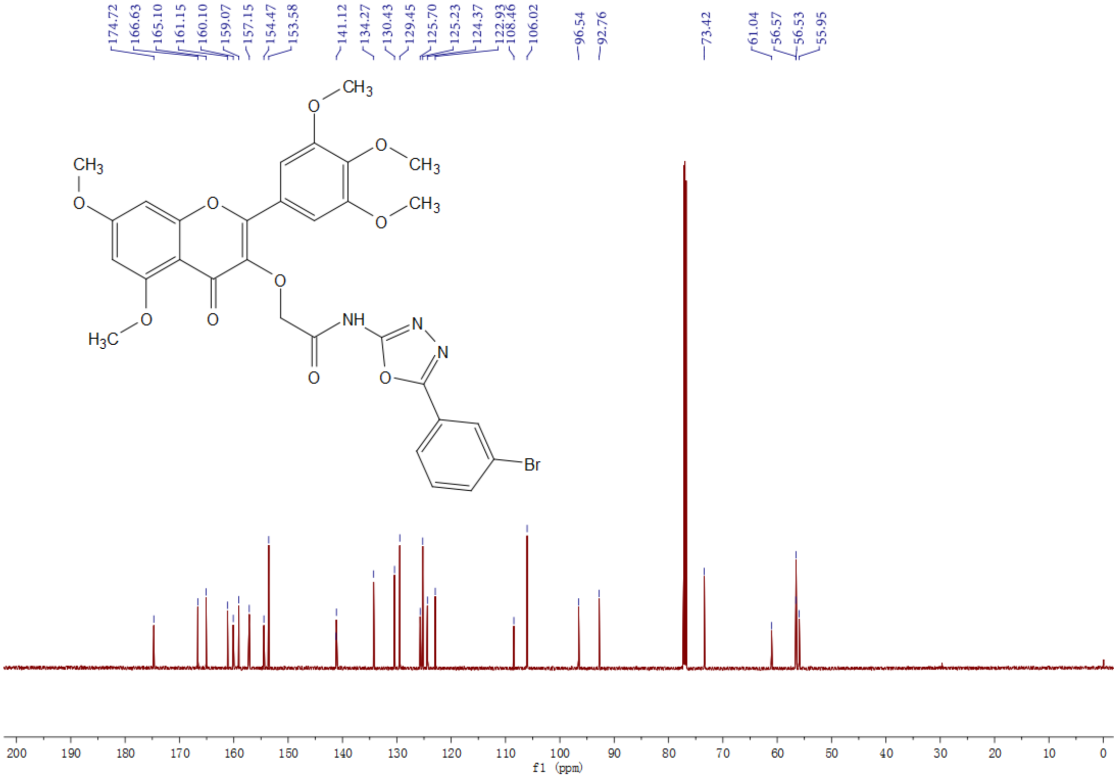

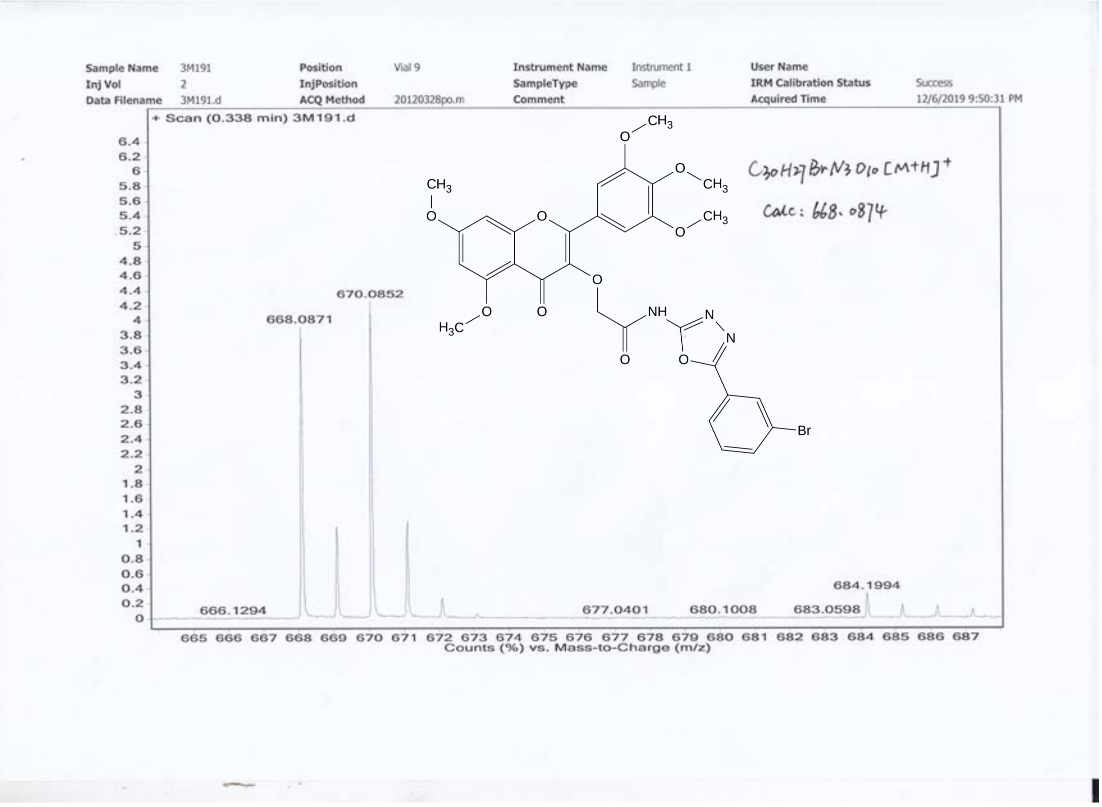

The spectrum of ^{1}H NMR, ^{13}C NMR and HRMS of compound A9

A10

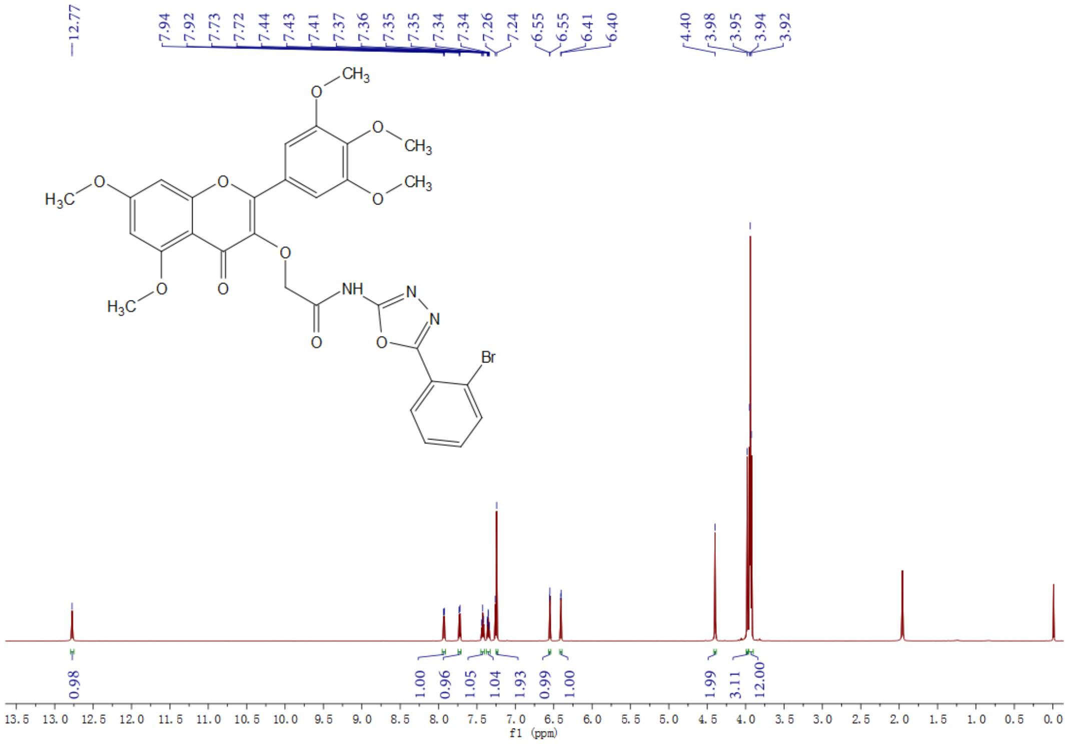

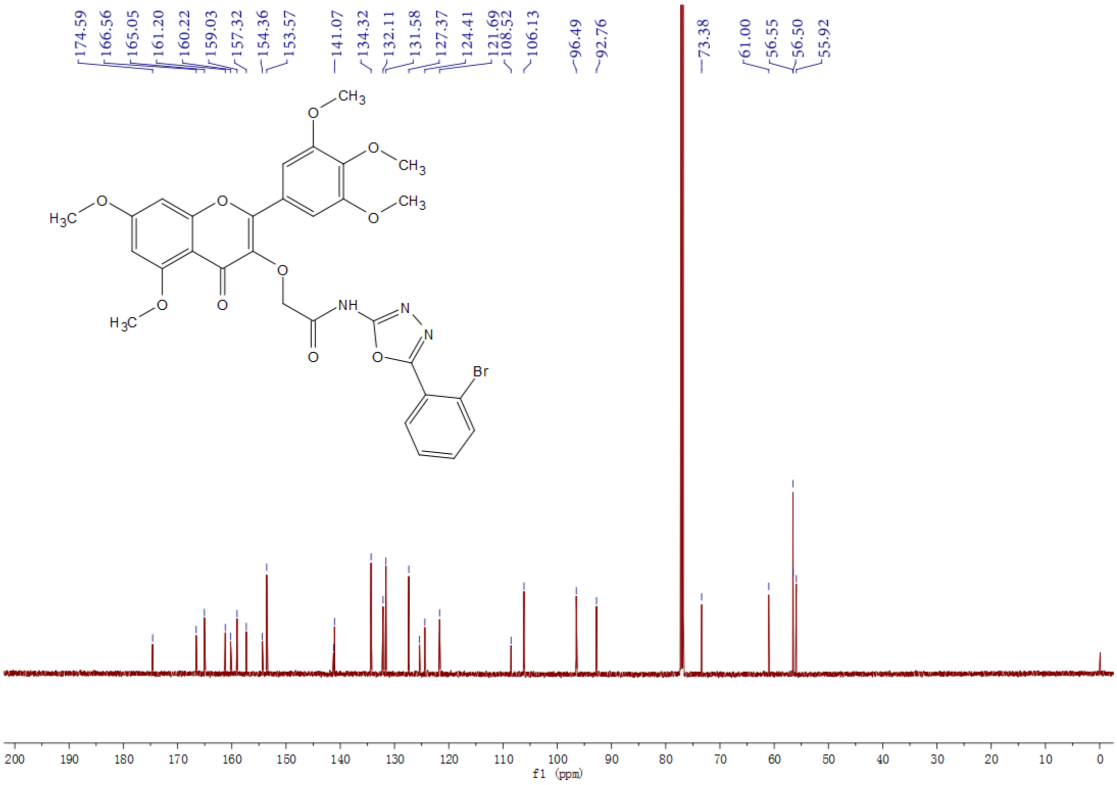

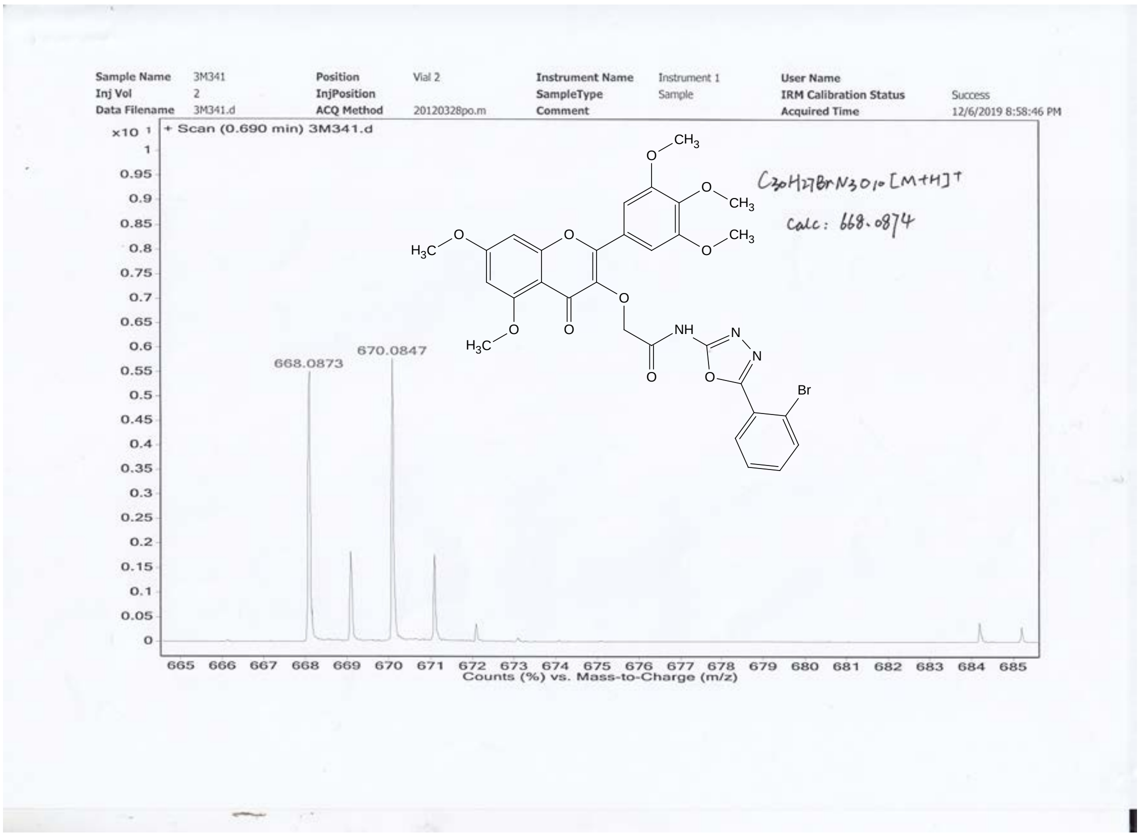

The spectrum of ^{1}H NMR, ^{13}C NMR and HRMS of compound A10

A11

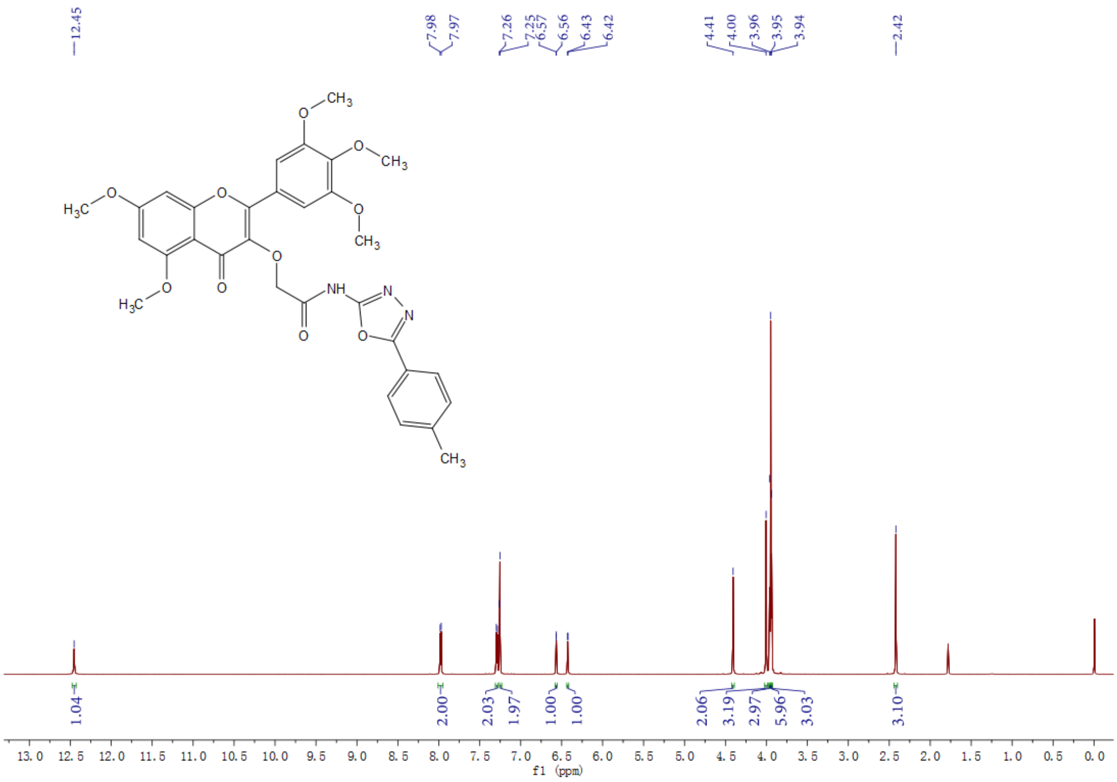

12.45
7.98
7.97
7.26
7.25
6.57
6.56
6.43
6.42
4.41
4.00
3.96
3.95
3.94
2.42
1.04
2.00
2.03
1.97
1.00
1.00
2.06
3.19
2.97
5.96
3.03
3.10
f1 (ppm)

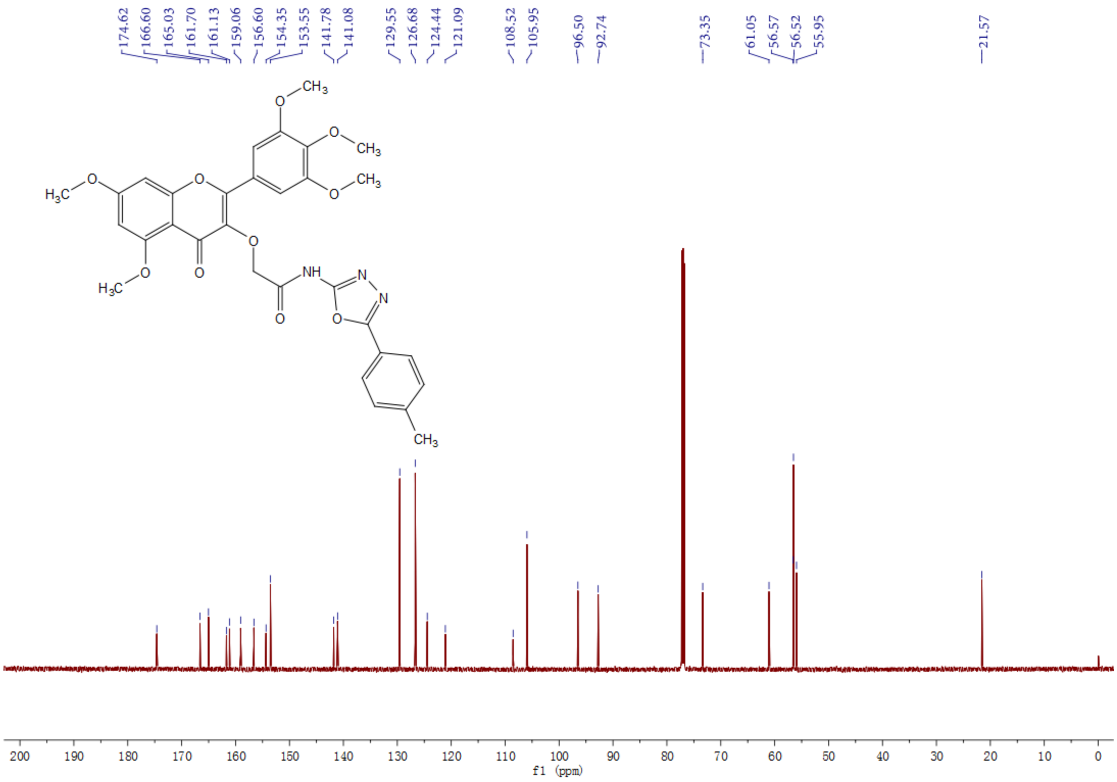

174.62
166.60
165.03
161.70
161.13
159.06
156.60
154.35
153.55
141.78
141.08
129.55
126.68
124.44
121.09
108.52
105.95
96.50
92.74
73.35
61.05
56.57
56.52
55.95
21.57
f1 (ppm)

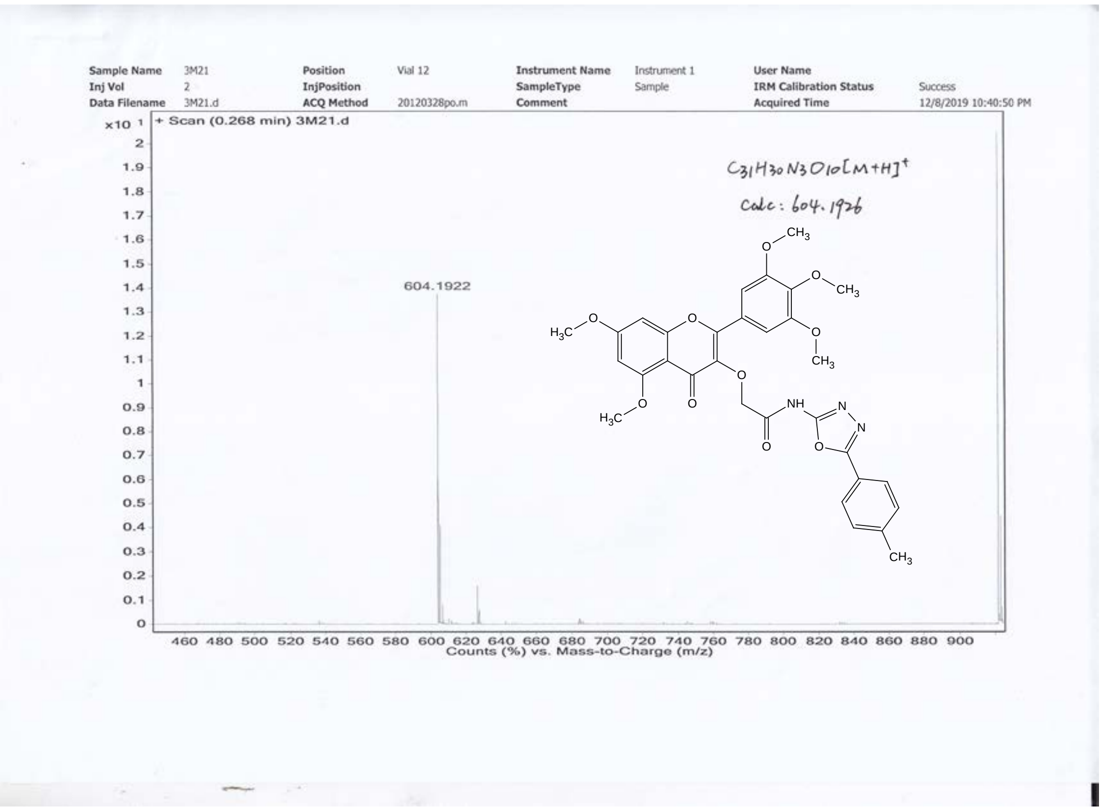

The spectrum of ^{1}H NMR, ^{13}C NMR and HRMS of compound A11

A12

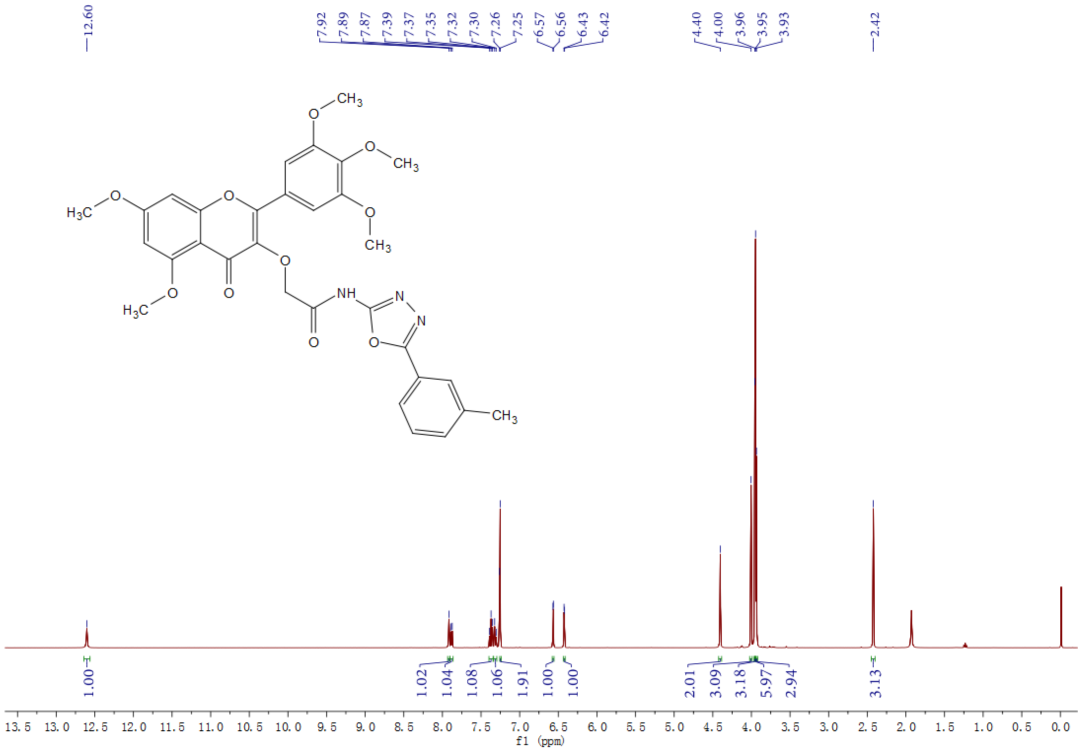

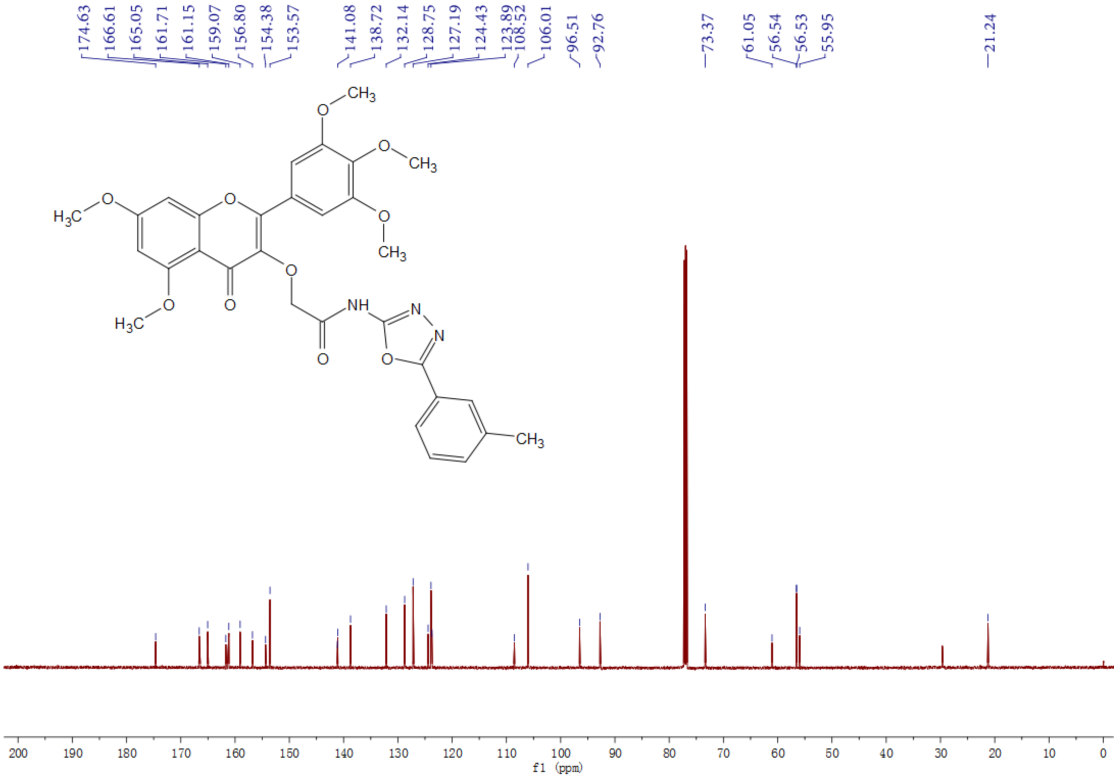

The spectrum of ^{1}H NMR and ^{13}C NMR of compound A12

A13

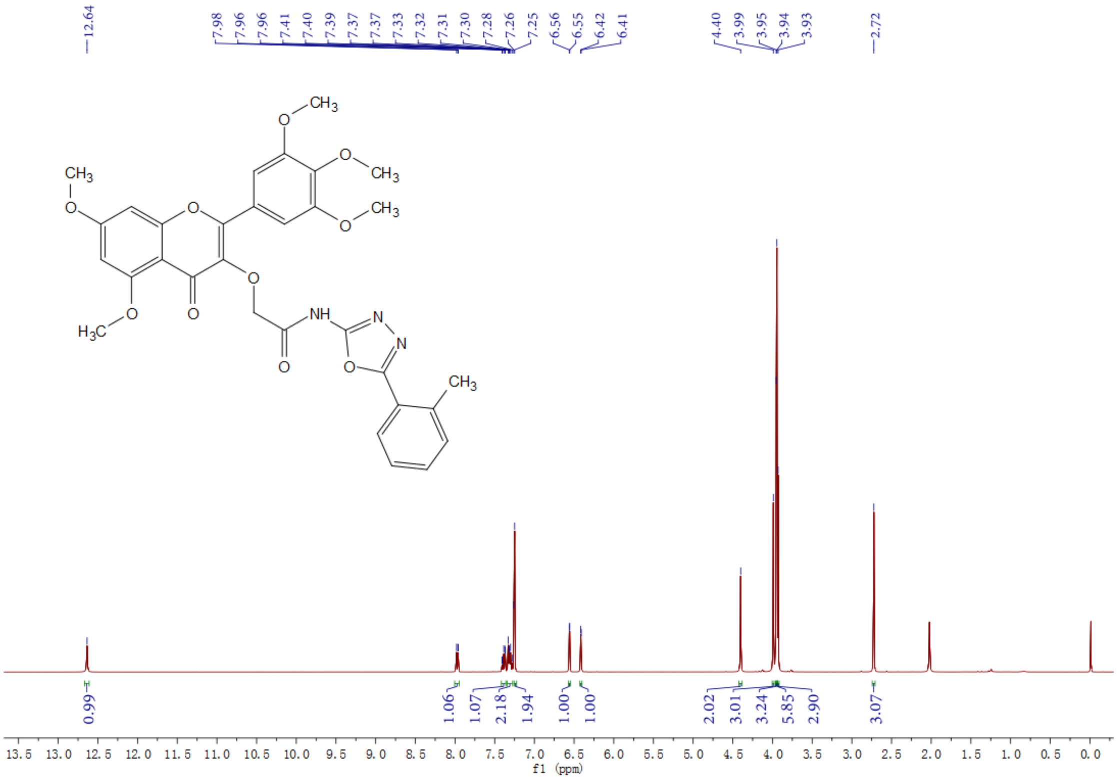

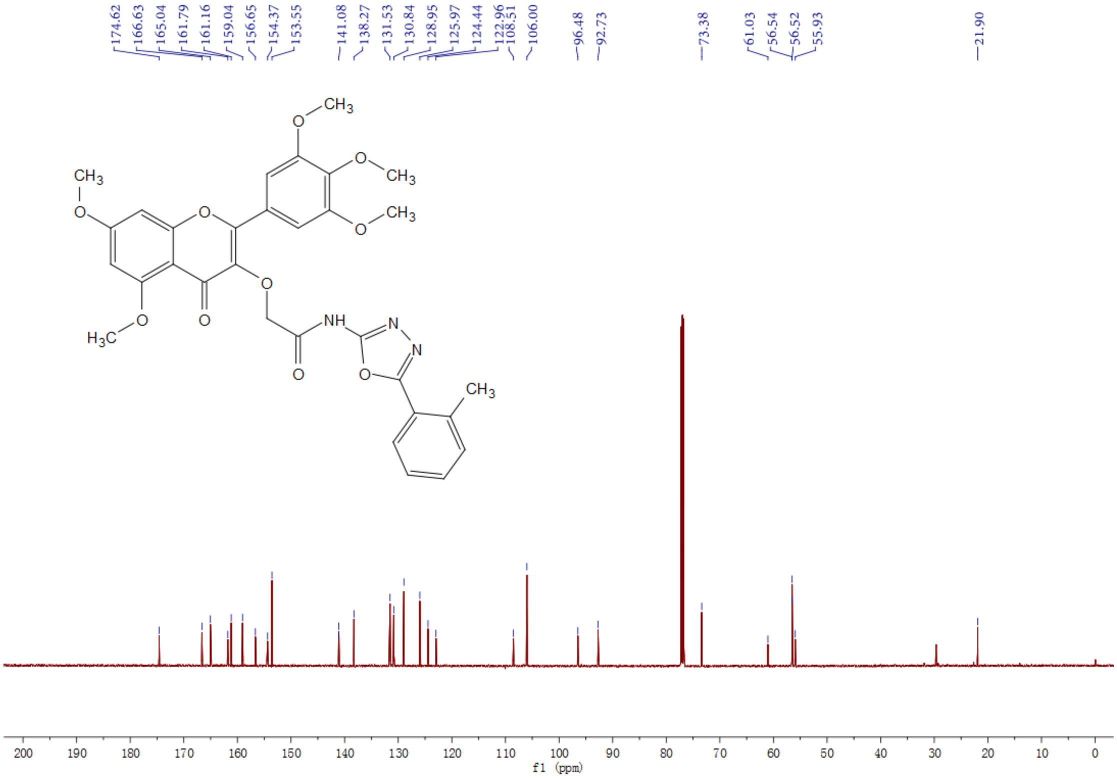

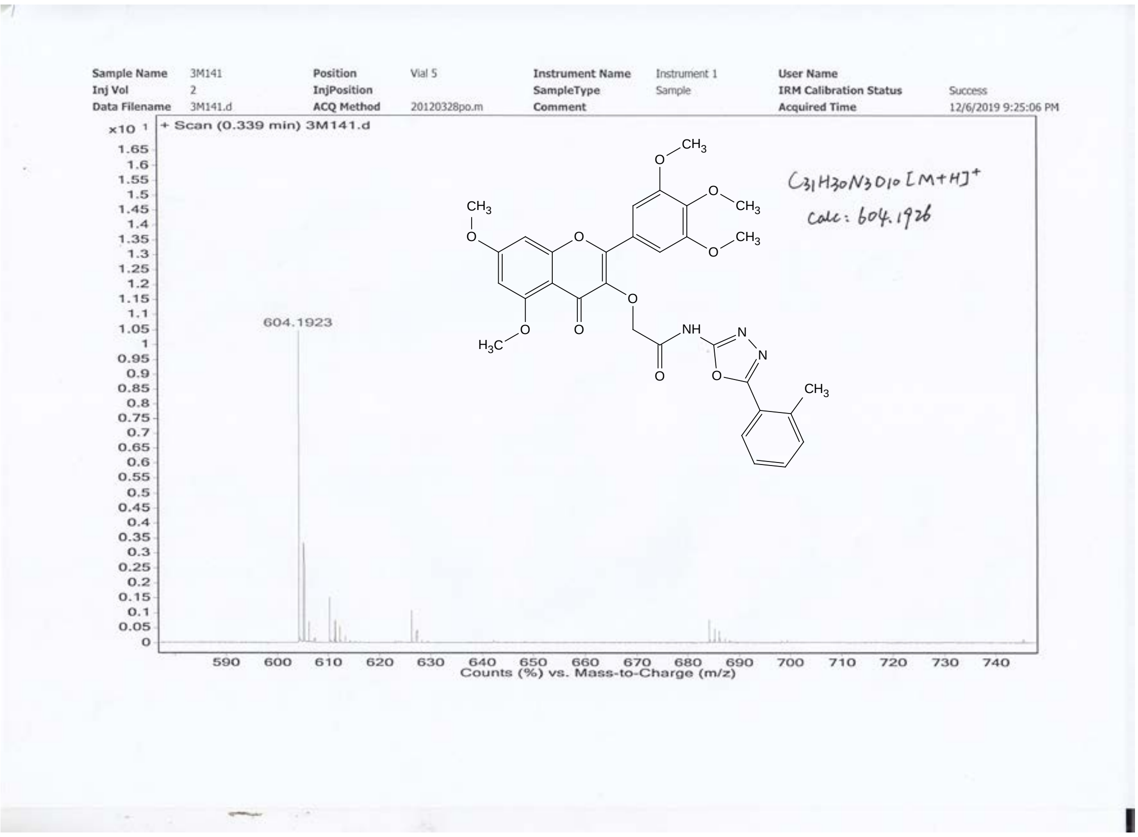

The spectrum of ^{1}H NMR, ^{13}C NMR and HRMS of compound A13

A14

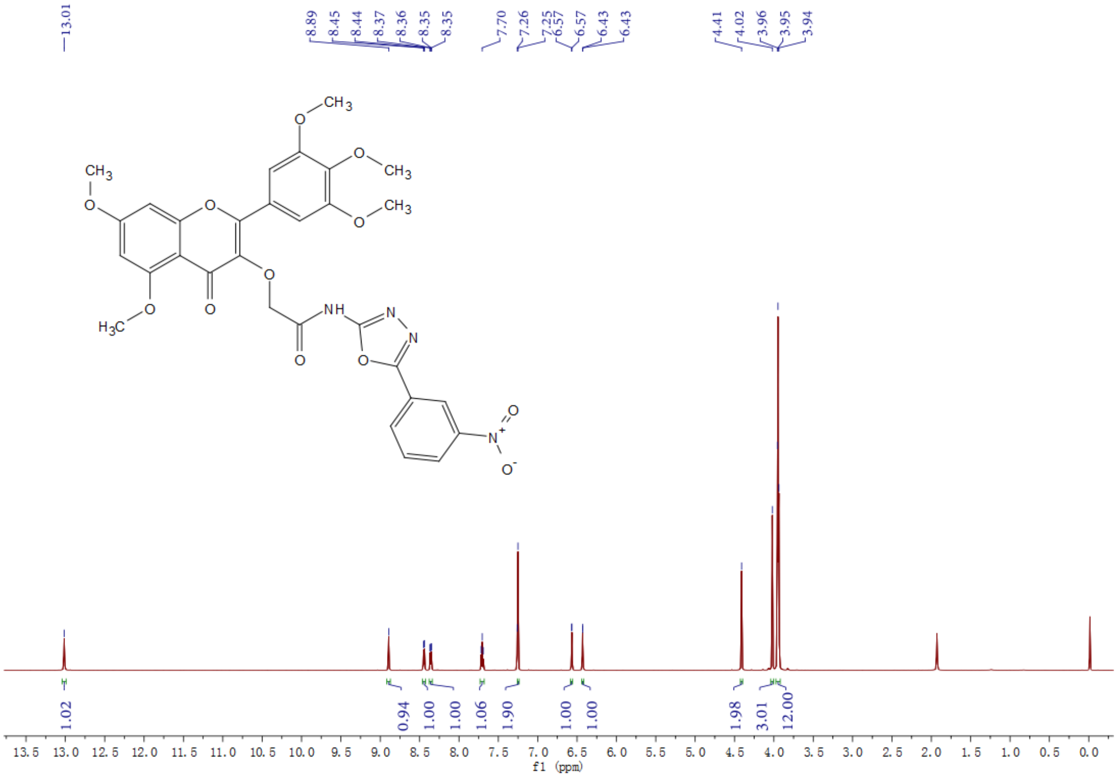

13.01
8.89
8.45
8.44
8.37
8.36
8.35
8.35
7.70
7.26
7.25
6.57
6.57
6.43
6.43
4.41
4.02
3.96
3.95
3.94
1.02
0.94
1.00
1.00
1.06
1.90
1.00
1.00
1.98
3.01
12.00
f1 (ppm)

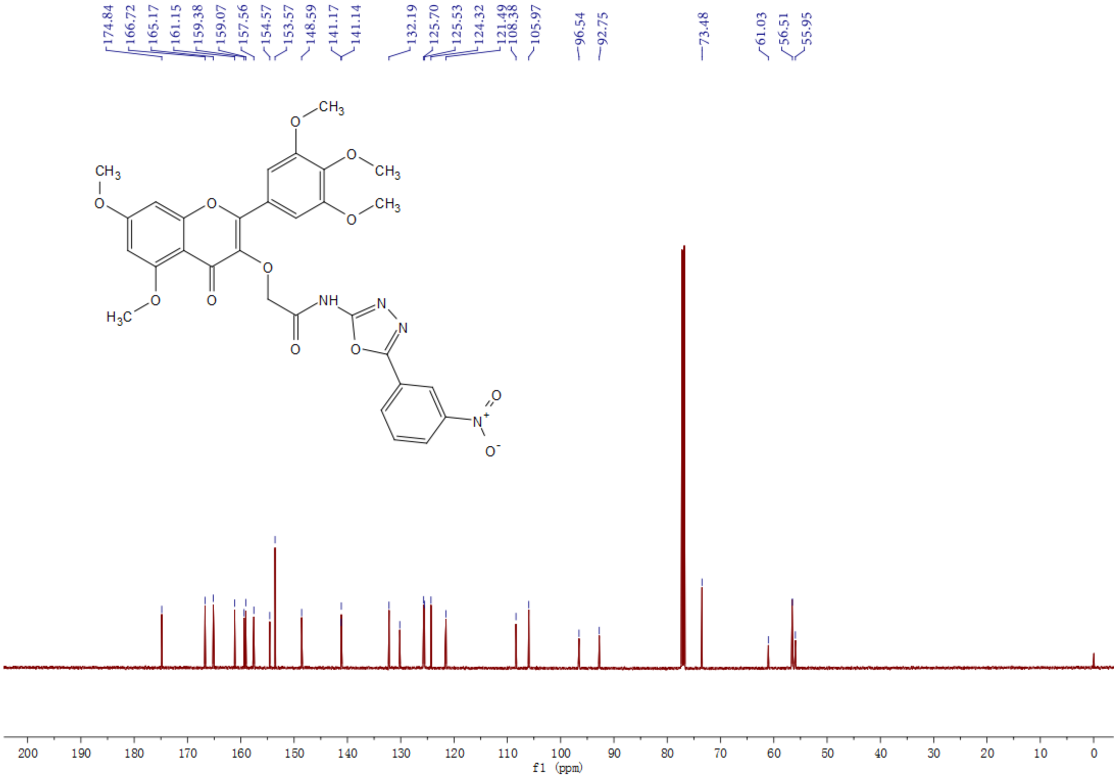

174.84
166.72
165.17
161.15
159.38
159.07
157.56
154.57
153.57
148.59
141.17
141.14
132.19
125.70
125.53
124.32
121.49
108.38
105.97
96.54
92.75
73.48
61.03
56.51
55.95
f1 (ppm)

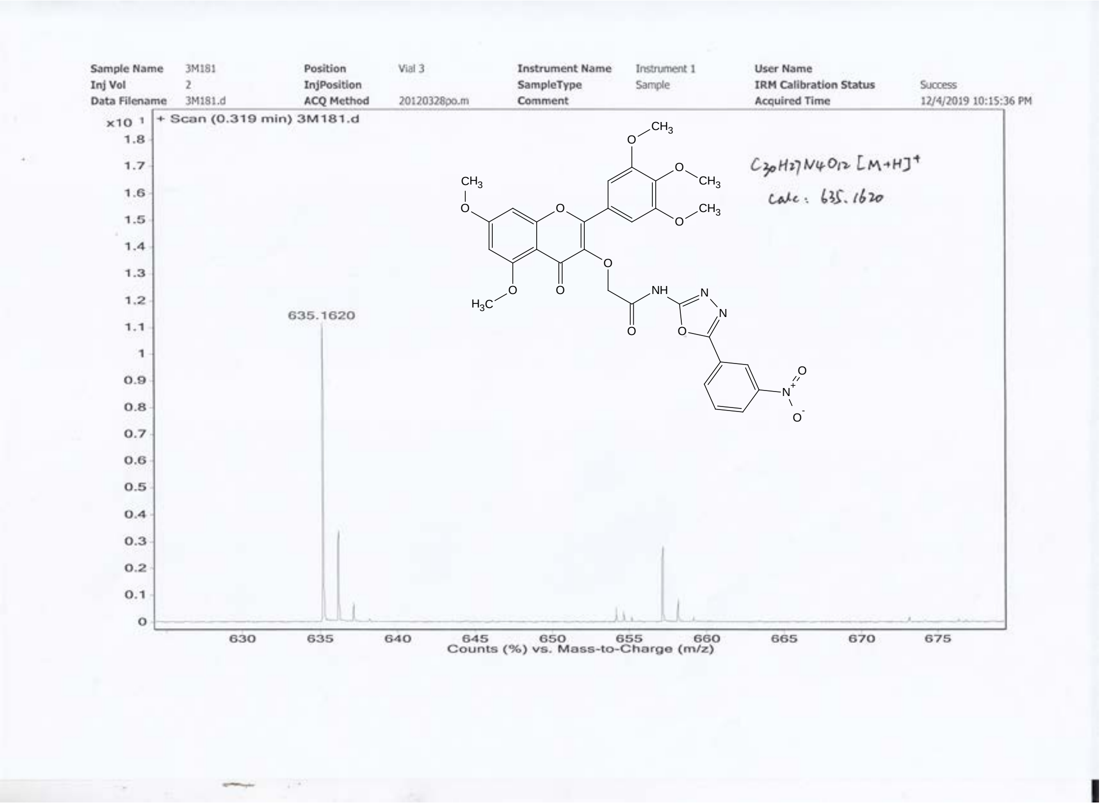

The spectrum of ^{1}H NMR, ^{13}C NMR and HRMS of compound A14

A15

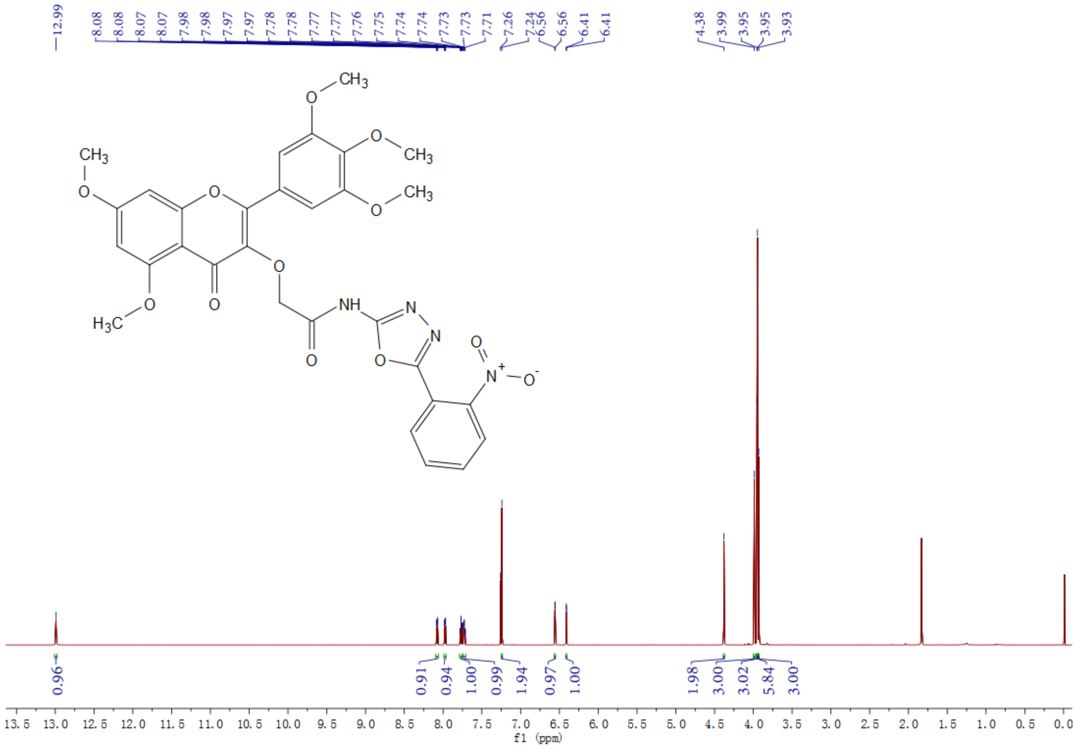

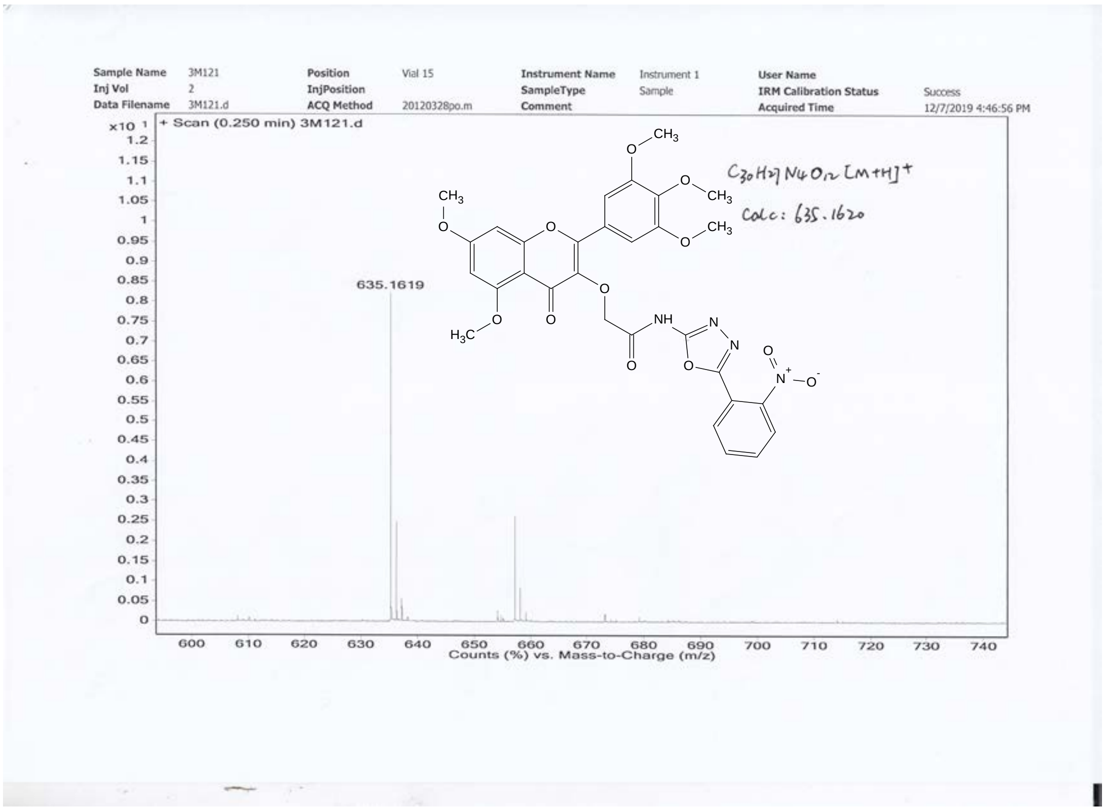

The spectrum of 1H NMR and HRMS of compound A15

A16

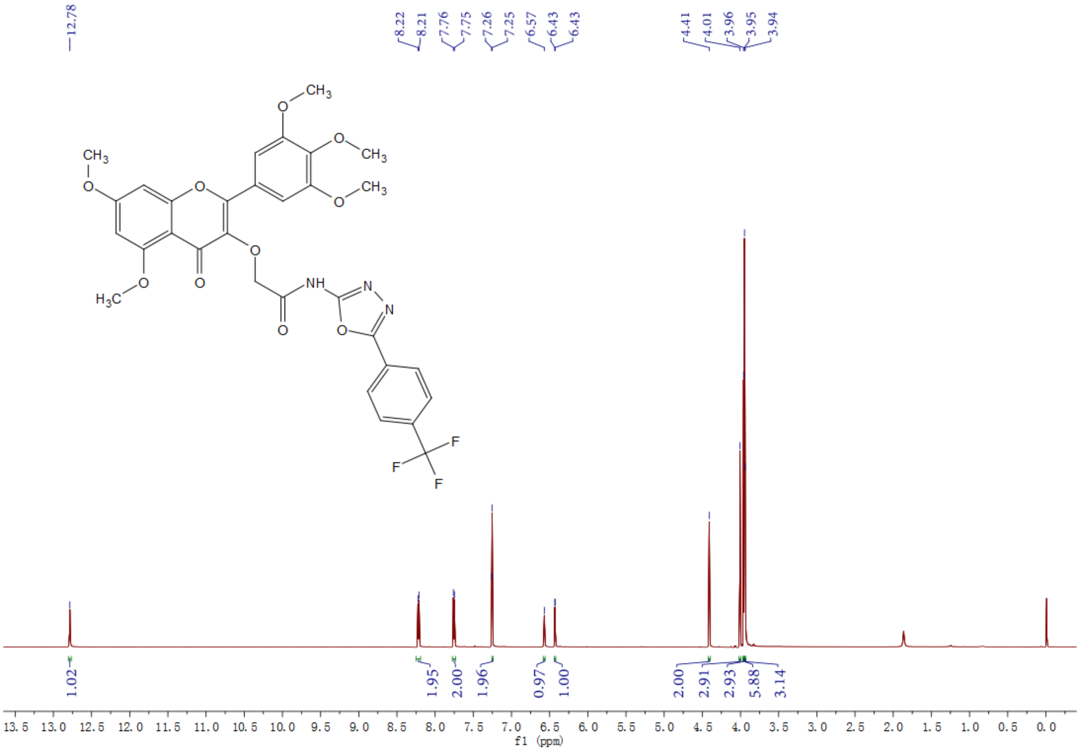

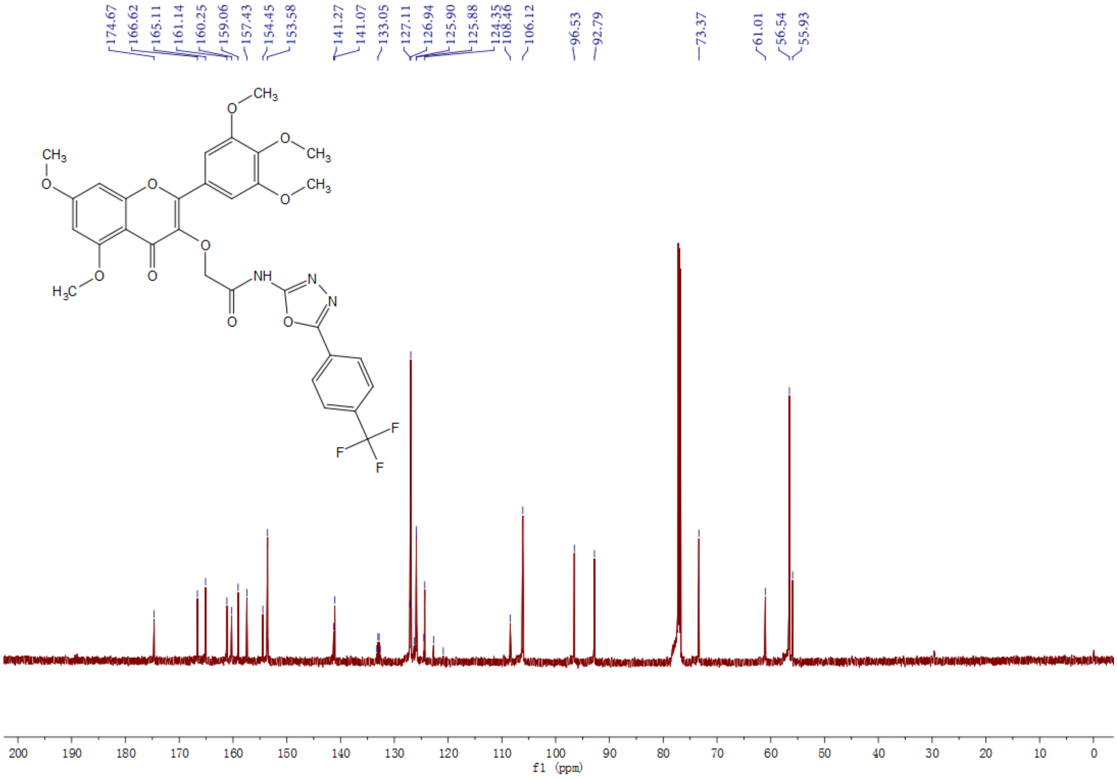

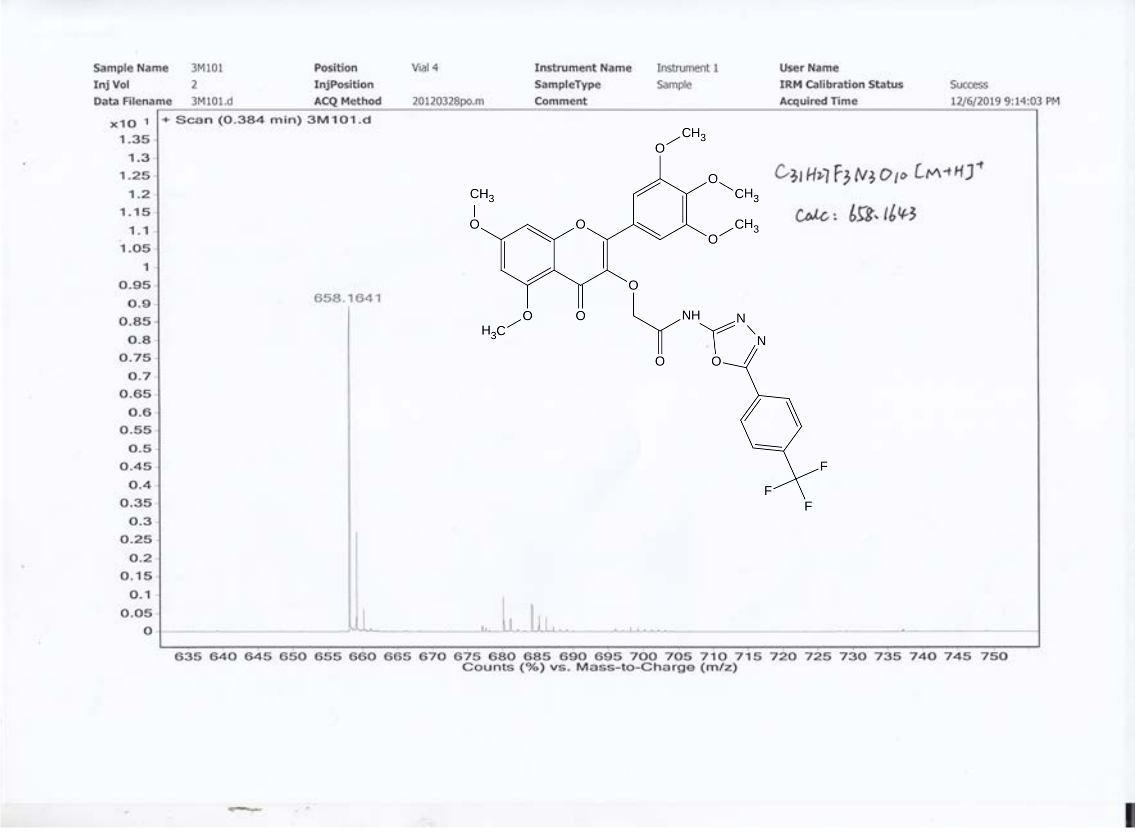

The spectrum of ^{1}H NMR, ^{13}C NMR and HRMS of compound A16

A17

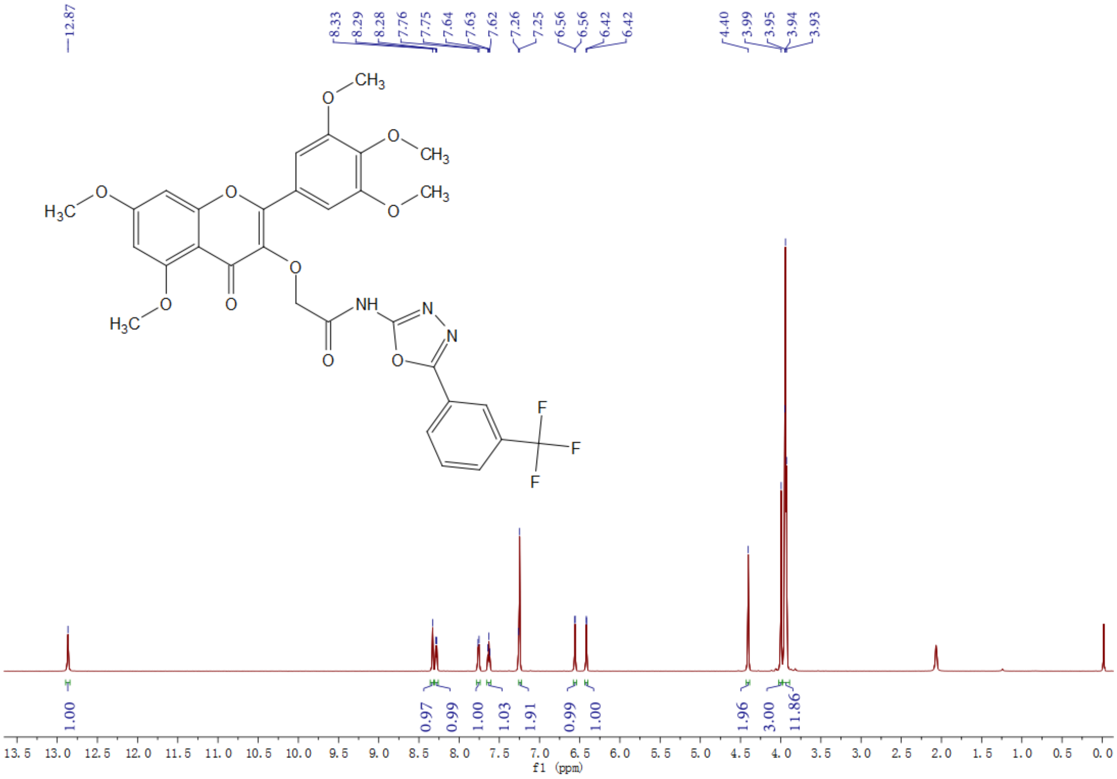

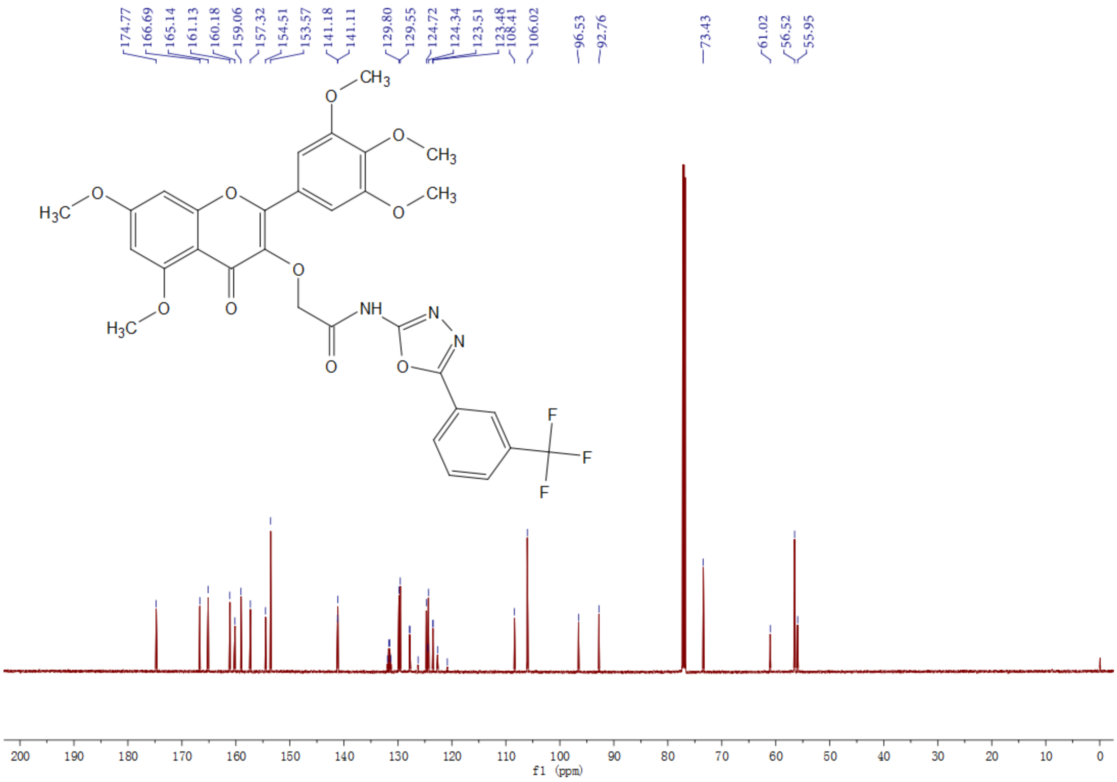

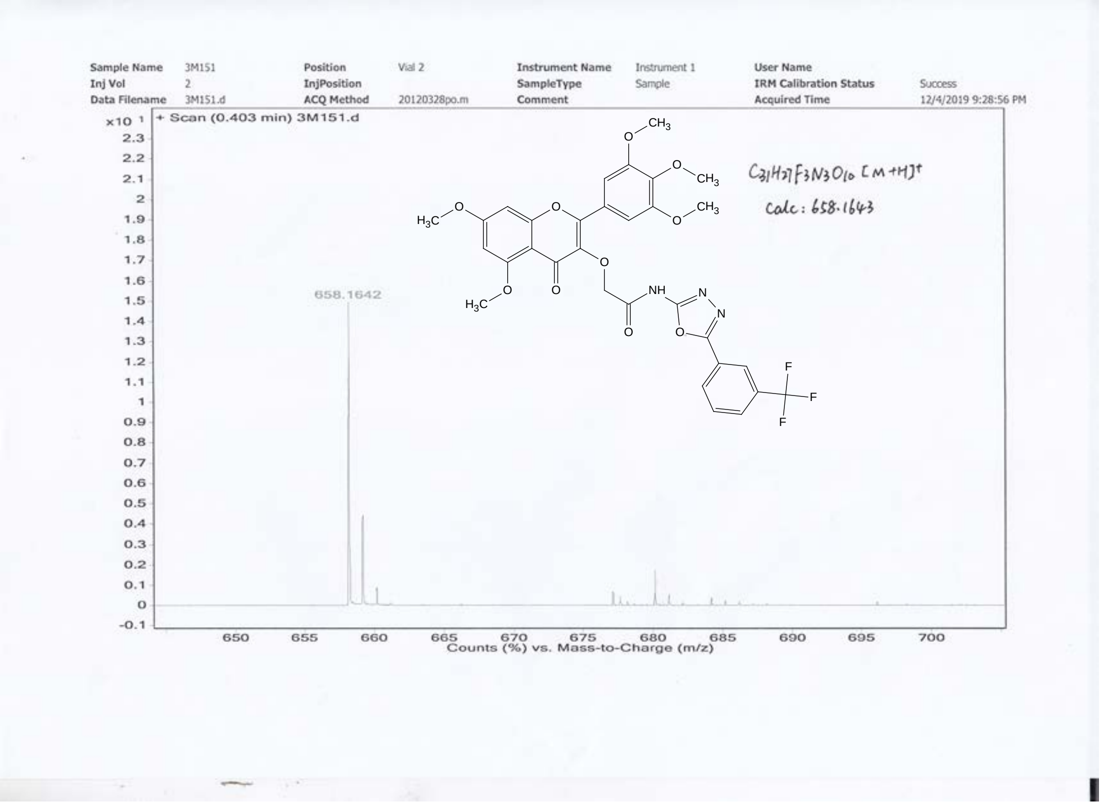

The spectrum of ^{1}H NMR, ^{13}C NMR and HRMS of compound A17

A18

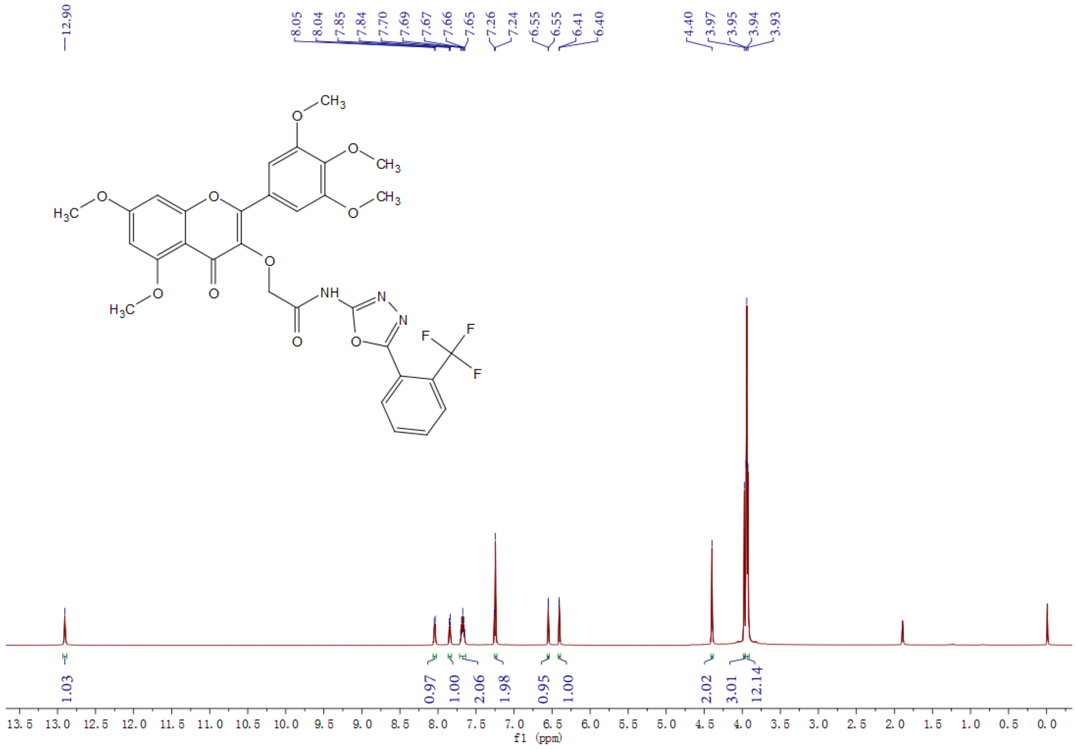

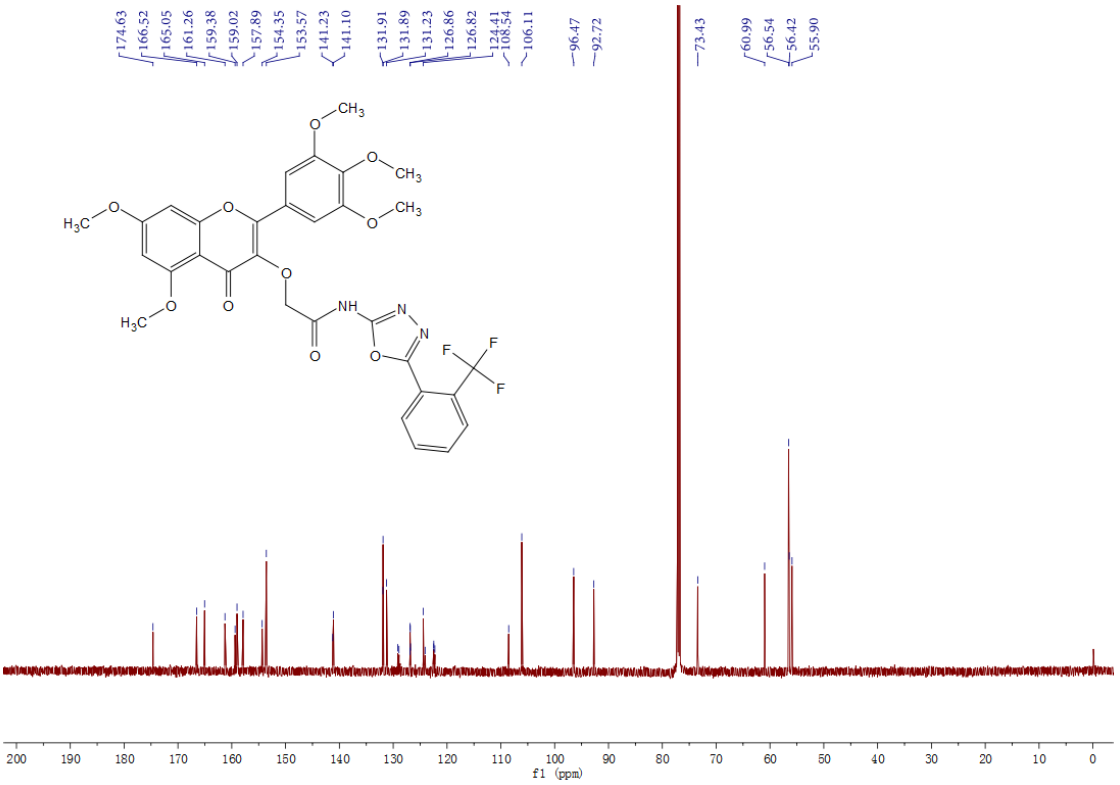

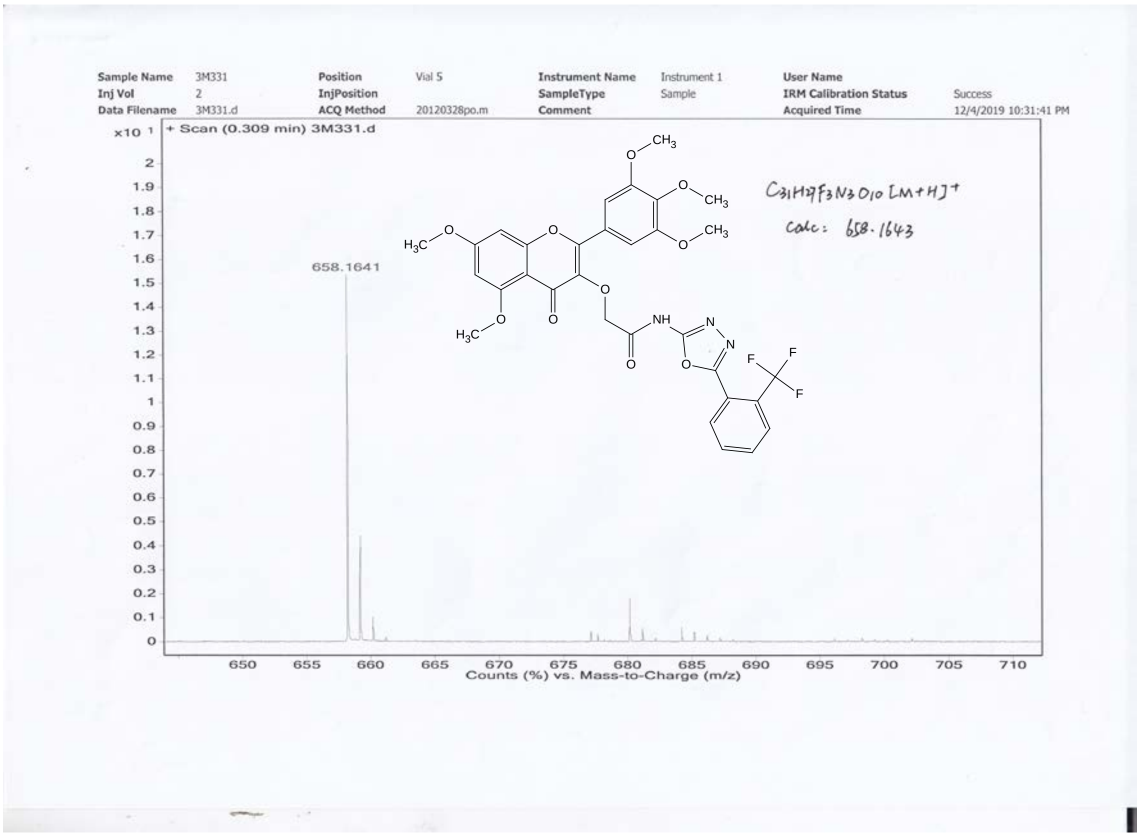

The spectrum of 1H NMR, 13C NMR and HRMS of compound A18

A19

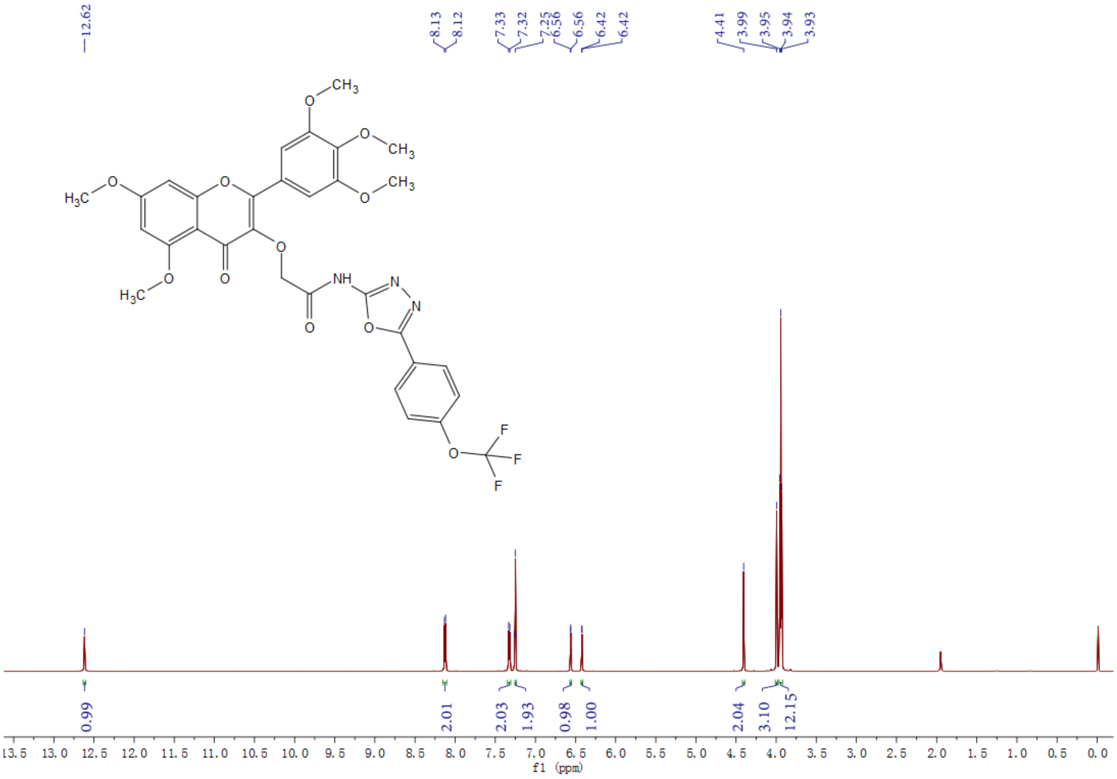

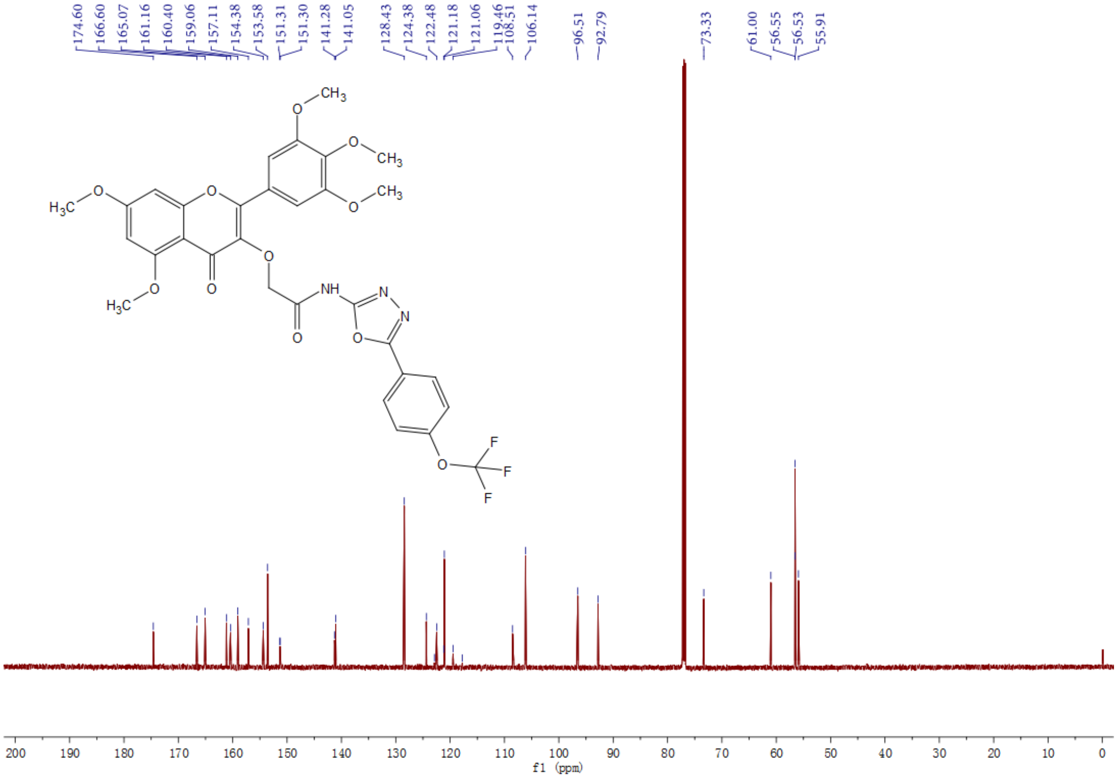

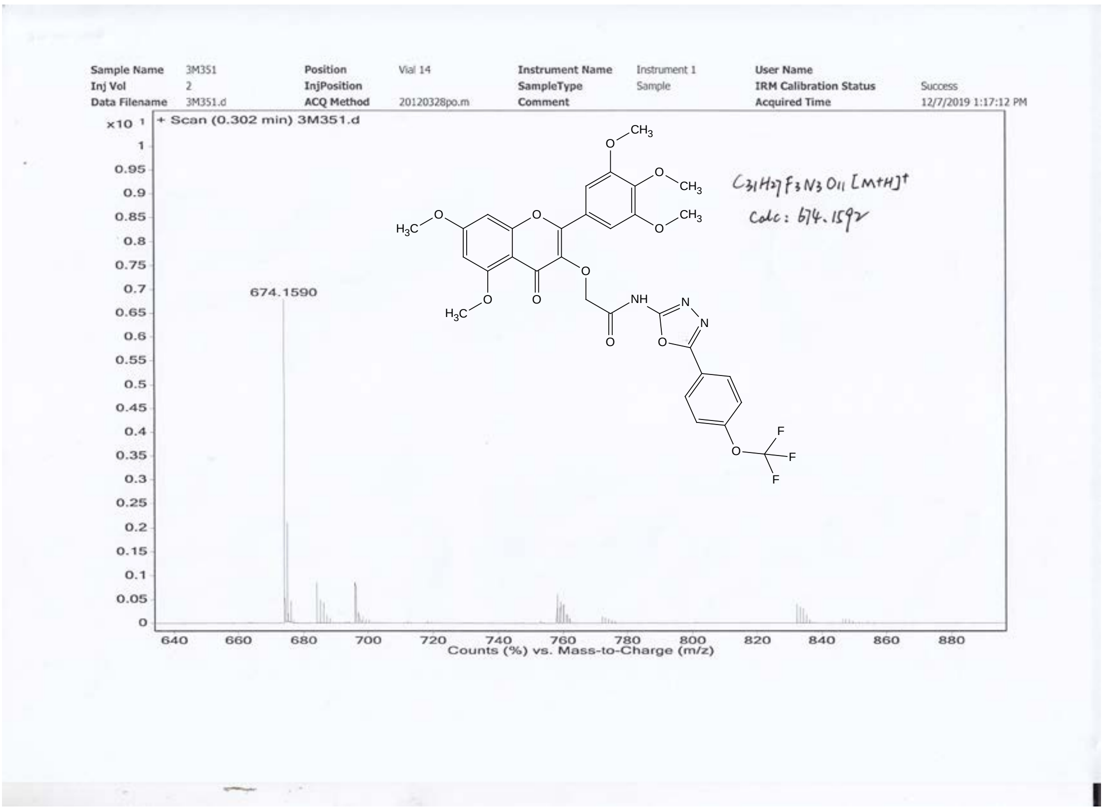

The spectrum of ^{1}H NMR, ^{13}C NMR and HRMS of compound A19

A20

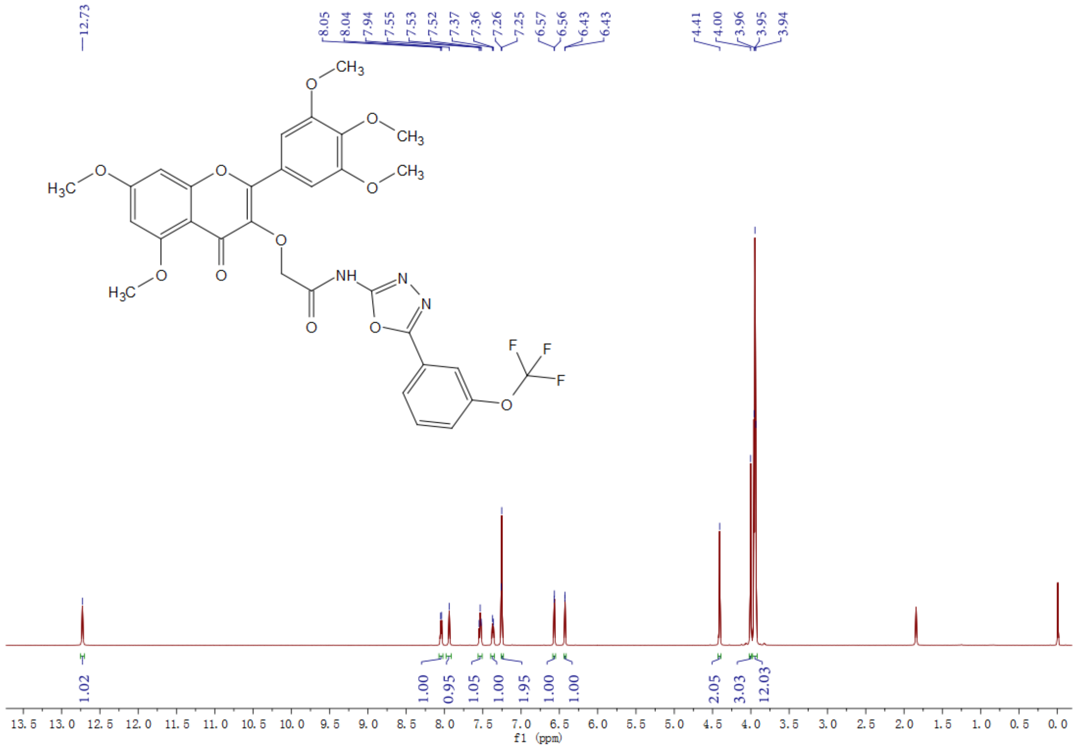

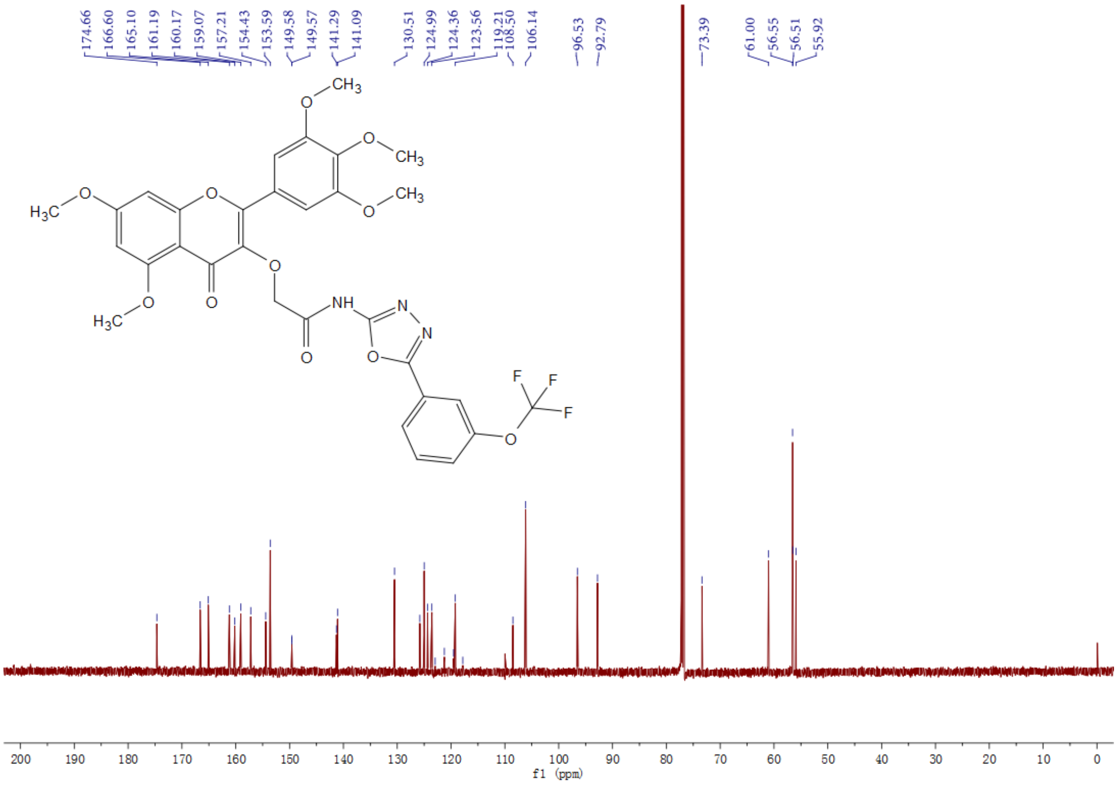

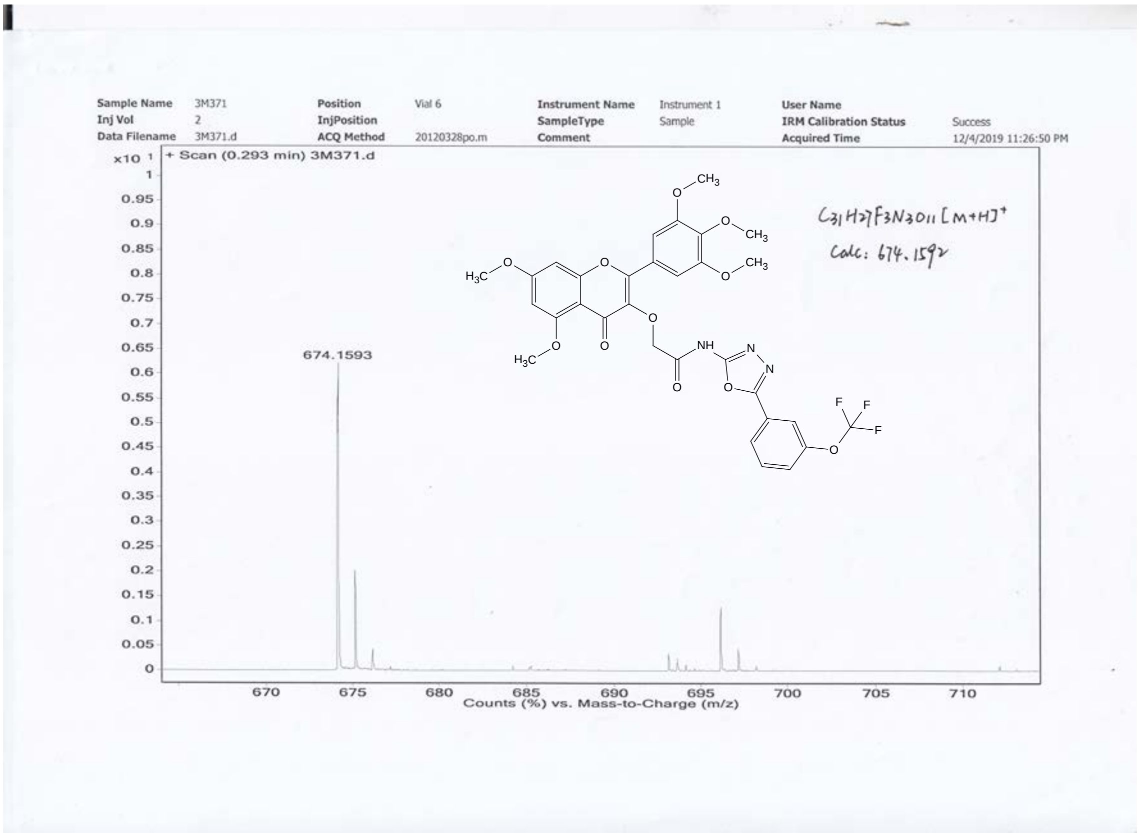

The spectrum of ^{1}H NMR, ^{13}C NMR and HRMS of compound A20

A21

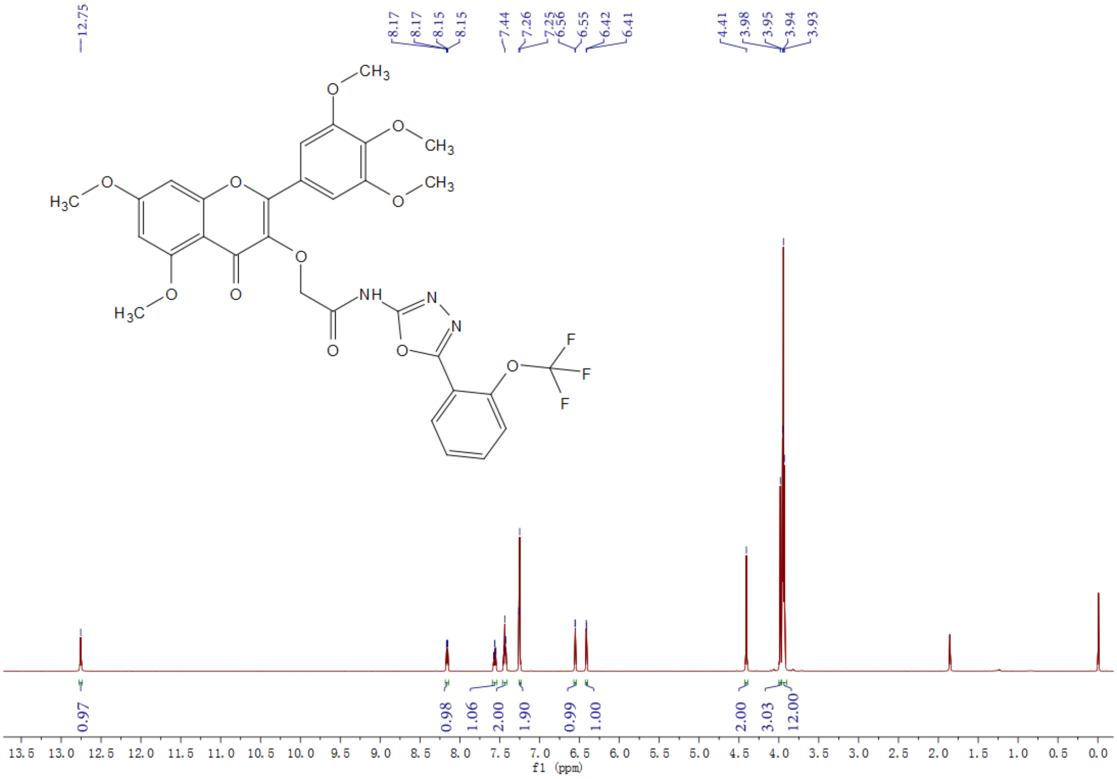
12.75
8.17
8.17
8.15
8.15
7.44
7.26
7.25
6.56
6.55
6.42
6.41
4.41
3.98
3.95
3.94
3.93
0.97
0.98
1.06
2.00
1.90
0.99
1.00
2.00
3.03
12.00
f1 (ppm)

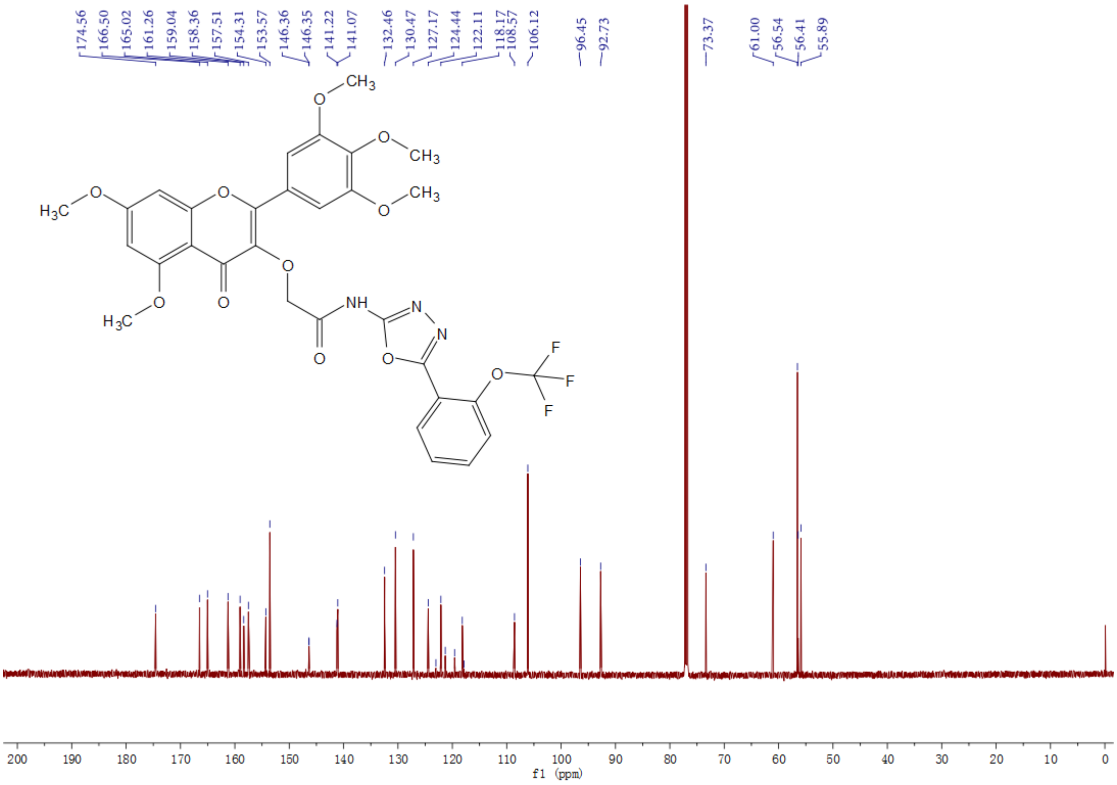
174.56
166.50
165.02
161.26
159.04
158.36
157.51
154.31
153.57
146.36
146.35
141.22
141.07
132.46
130.47
127.17
124.44
122.11
118.17
108.57
106.12
96.45
92.73
73.37
61.00
56.54
56.41
55.89
f1 (ppm)

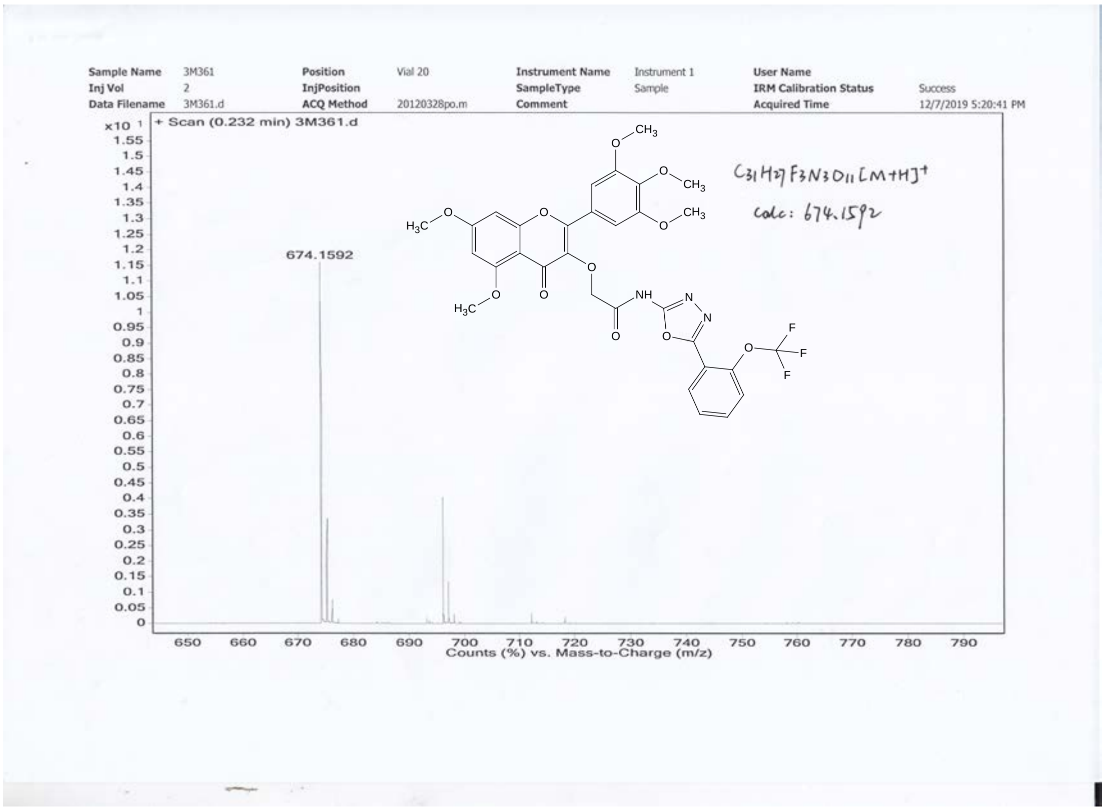

The spectrum of ^{1}H NMR, ^{13}C NMR and HRMS of compound A21

A22

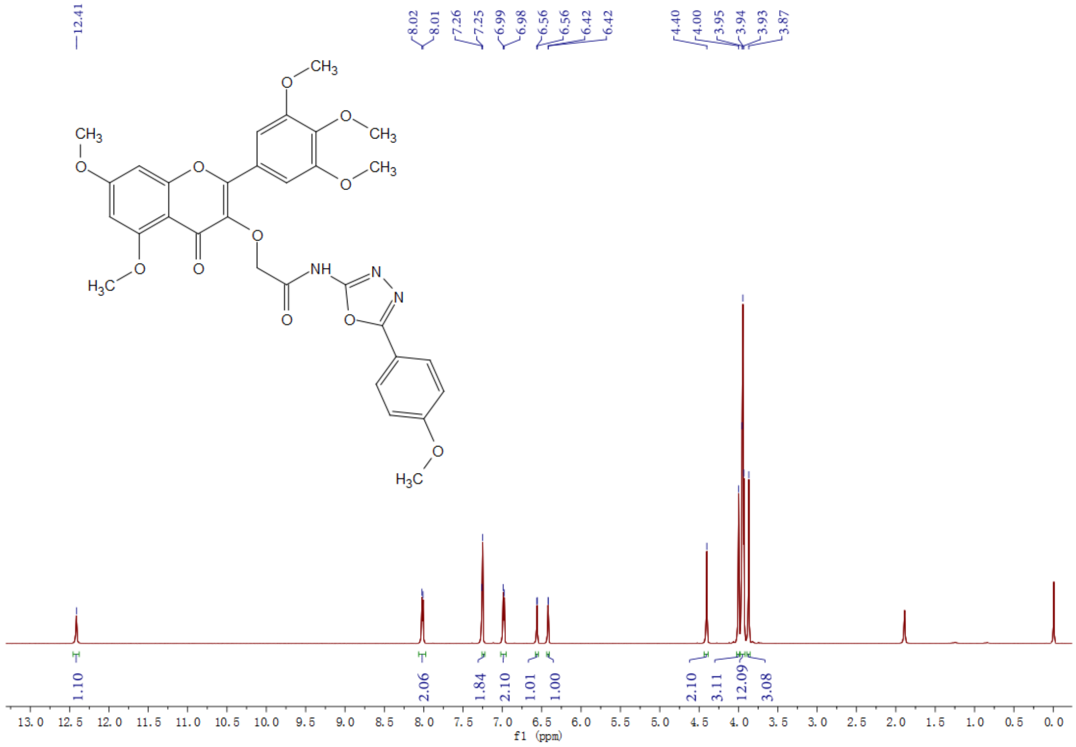

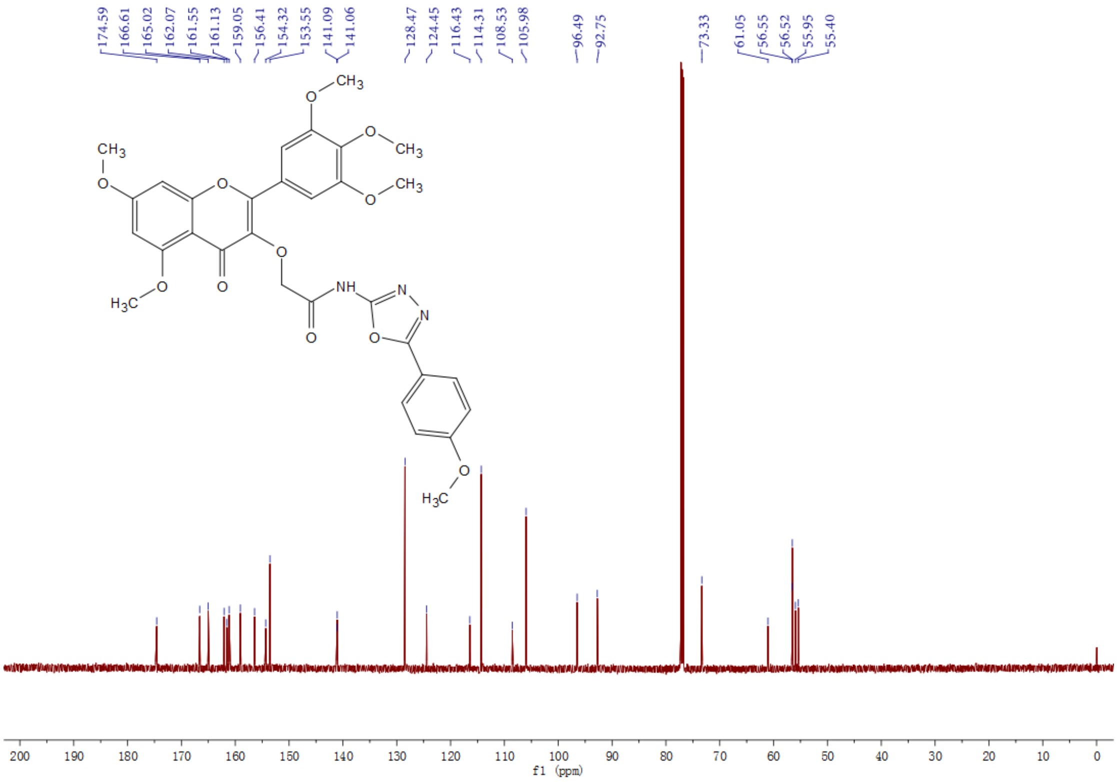

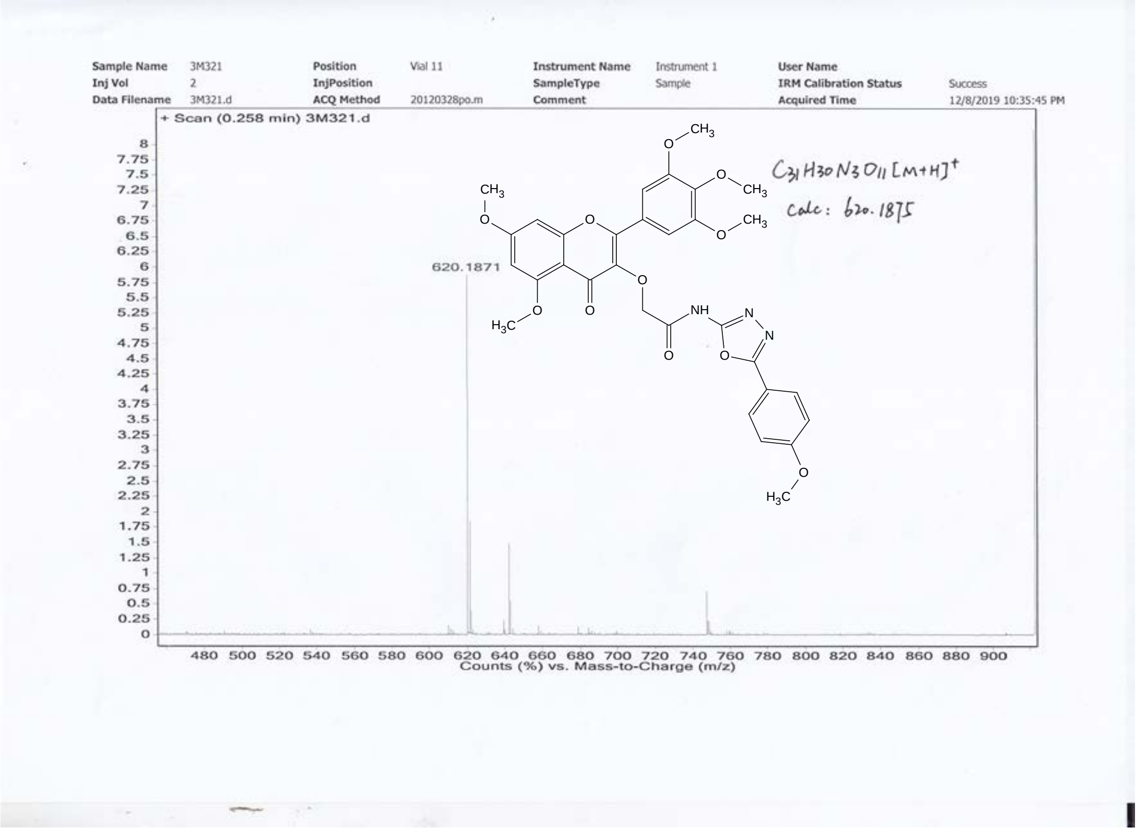

The spectrum of ^{1}H NMR, ^{13}C NMR and HRMS of compound A22

A23

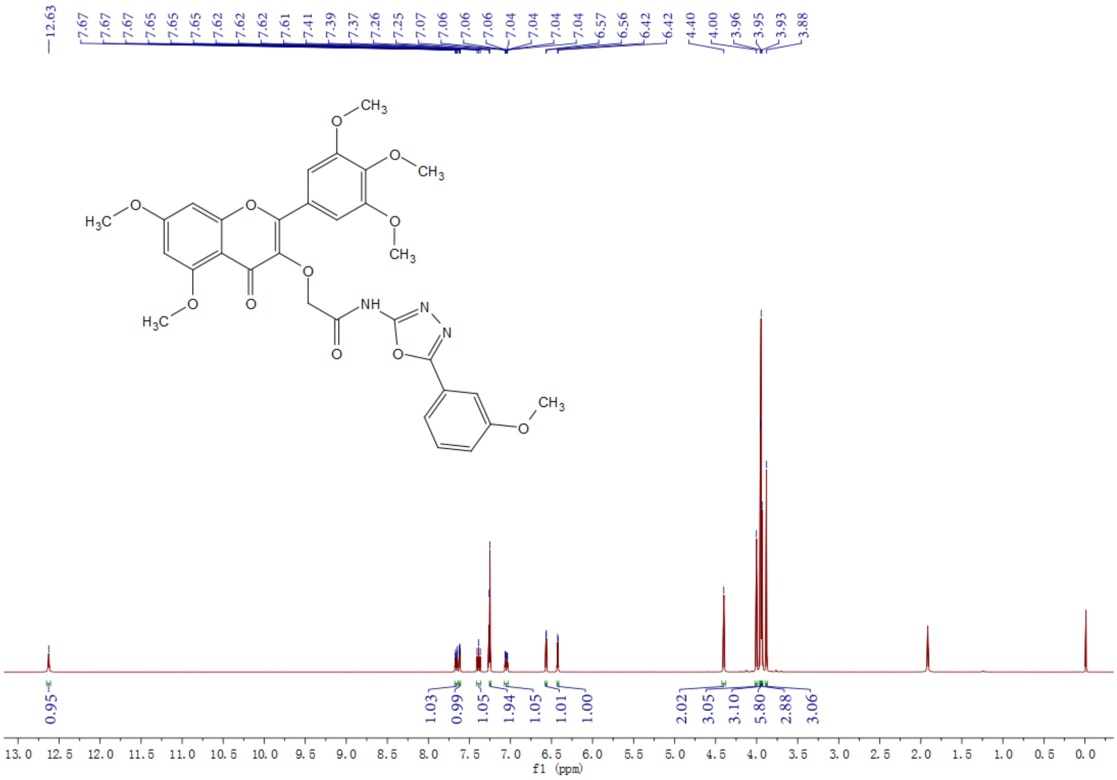

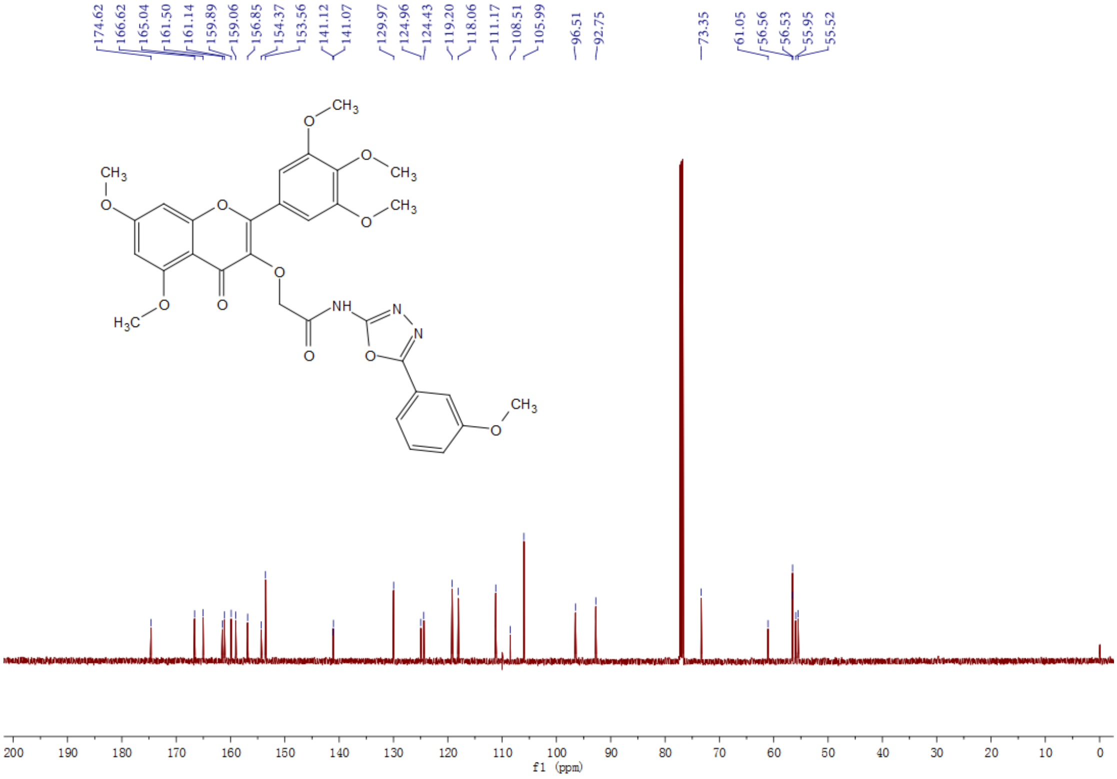

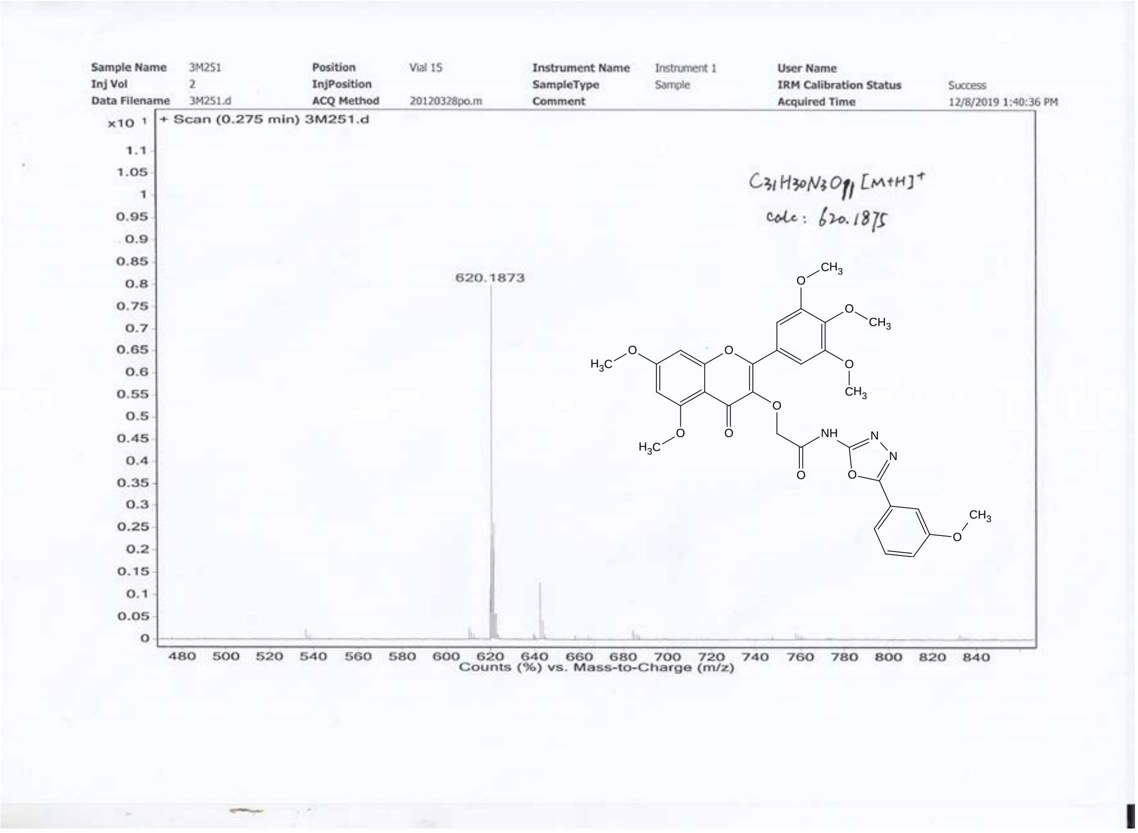

The spectrum of ^{1}H NMR, ^{13}C NMR and HRMS of compound A23

A24

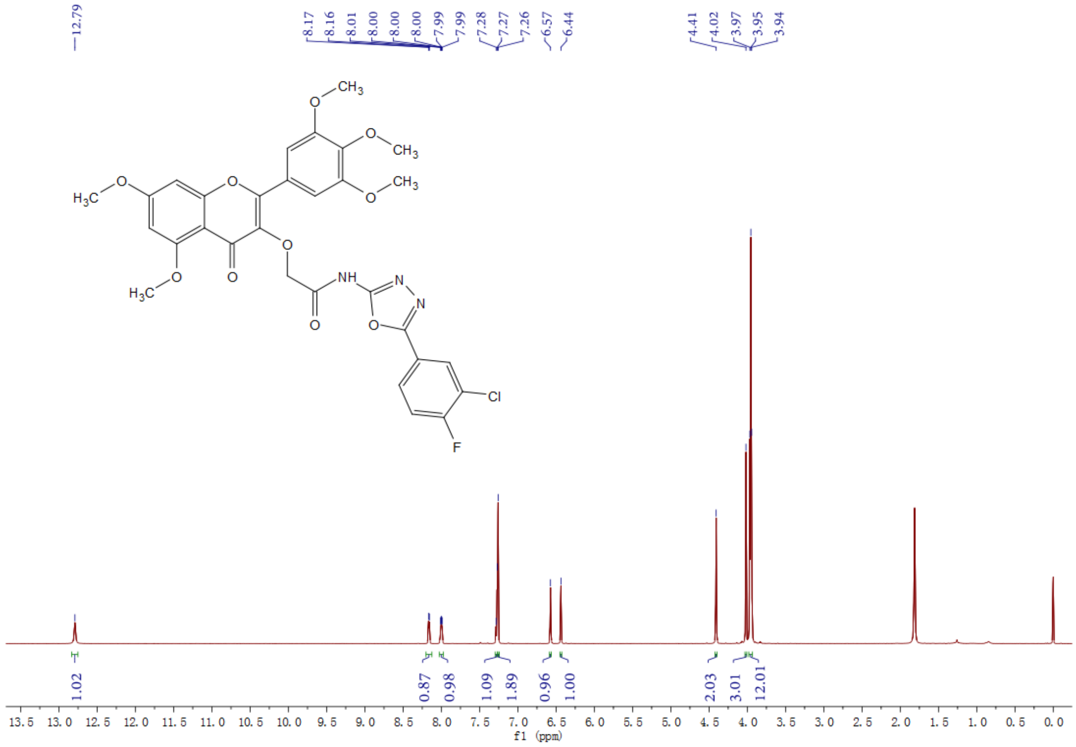

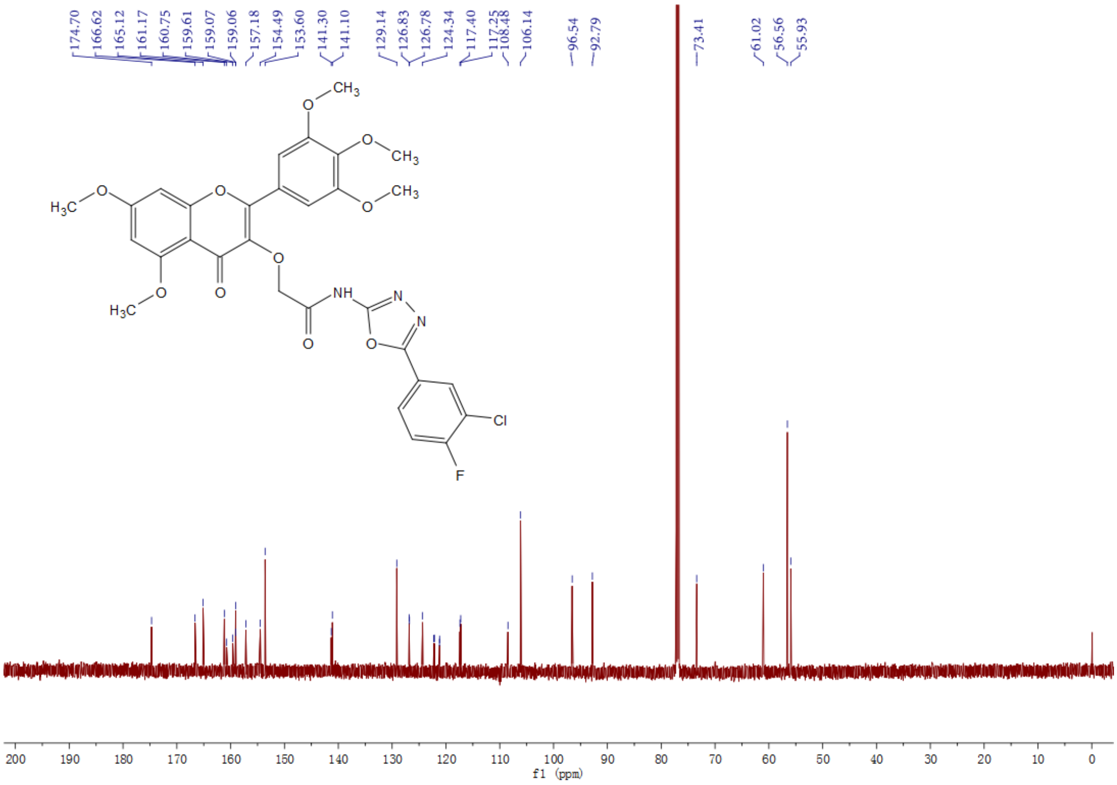

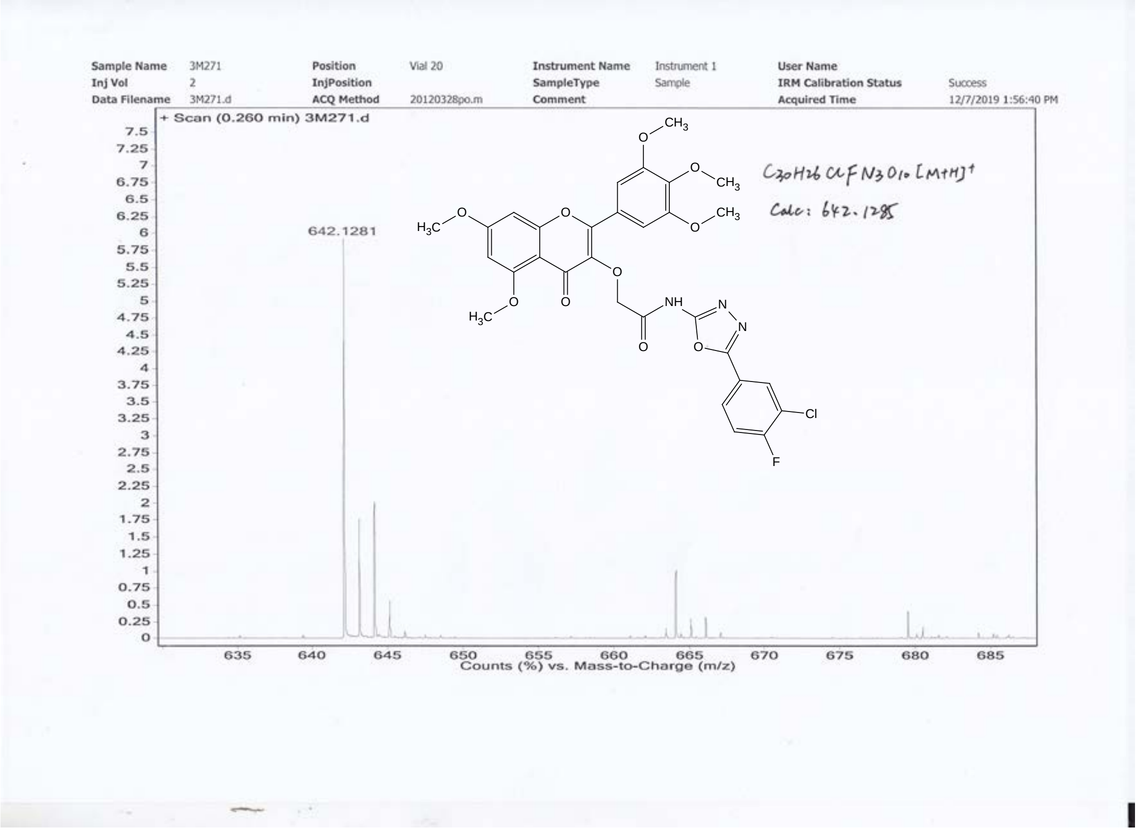

The spectrum of ^{1}H NMR, ^{13}C NMR and HRMS of compound A24

A25

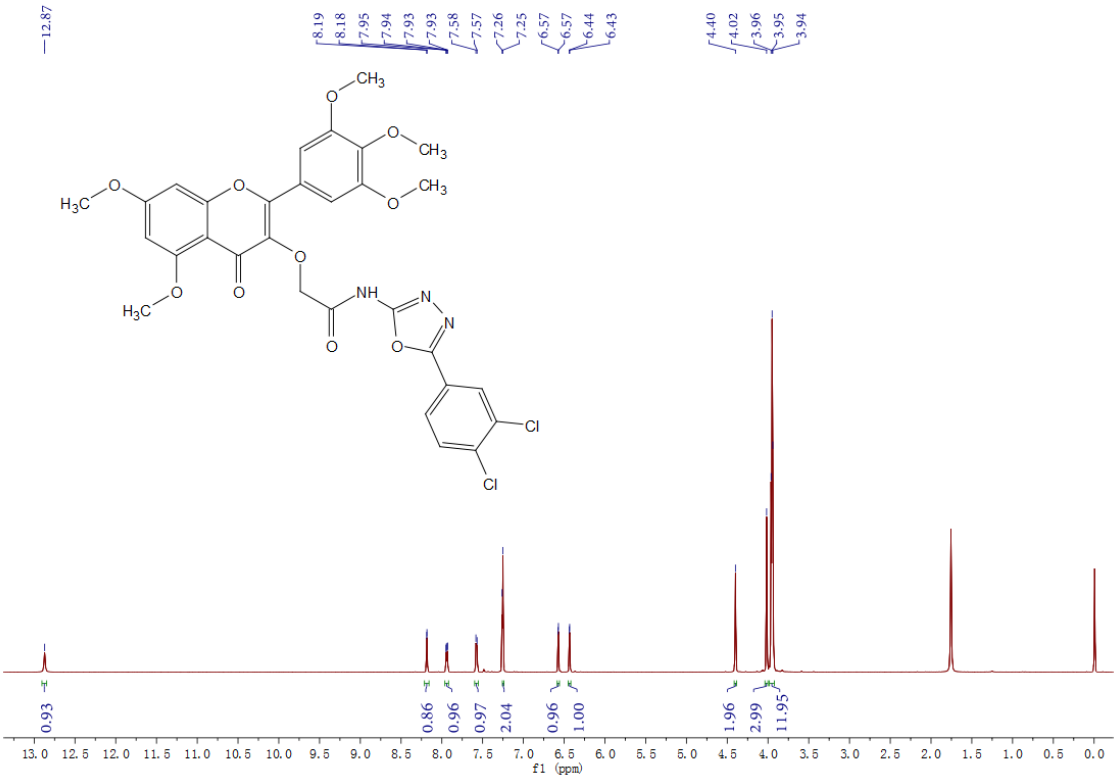

12.87
8.19
8.18
7.95
7.94
7.93
7.93
7.58
7.57
7.26
7.25
6.57
6.57
6.44
6.43
4.40
4.02
3.96
3.95
3.94
CH3
CH3
CH3
H3C
H3C
O
O
O
O
O
O
O
NH
N
N
O
O
Cl
Cl
0.93
0.86
0.96
0.97
2.04
0.96
1.00
1.96
2.99
11.95
13.0
12.5
12.0
11.5
11.0
10.5
10.0
9.5
9.0
8.5
8.0
7.5
7.0
6.5
6.0
5.5
5.0
4.5
4.0
3.5
3.0
2.5
2.0
1.5
1.0
0.5
0.0
f1 (ppm)

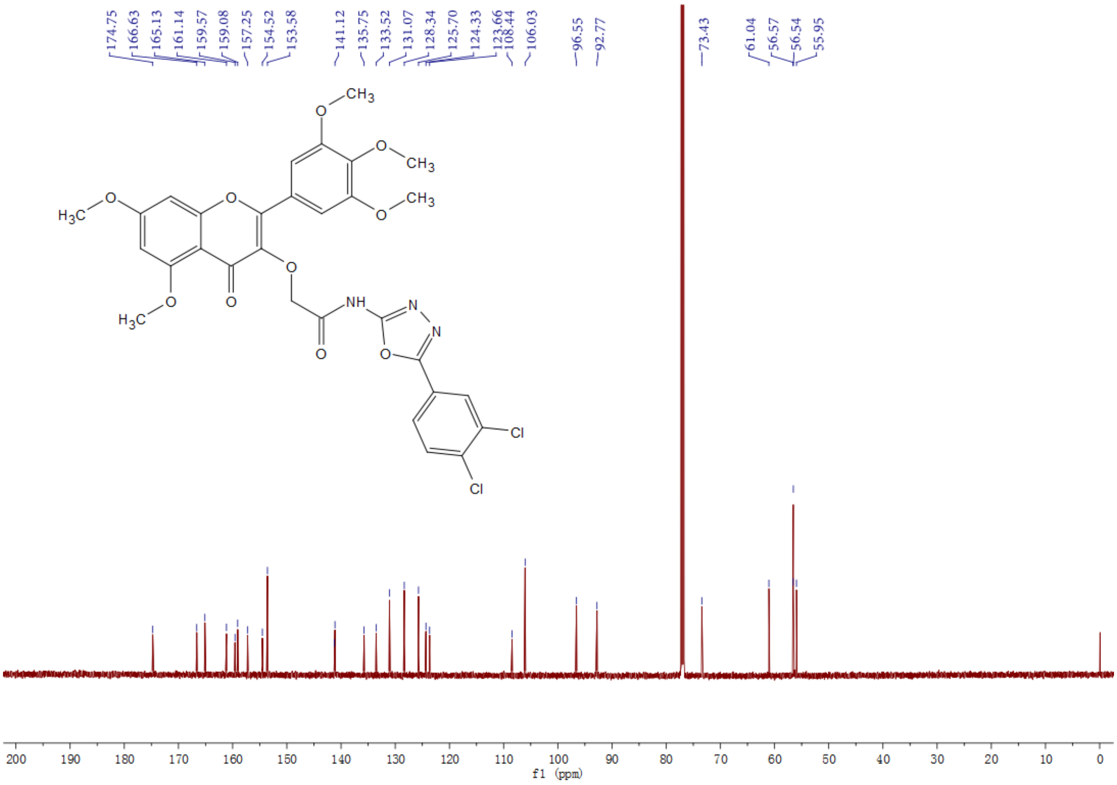

174.75
166.63
165.13
161.14
159.57
159.08
157.25
154.52
153.58
141.12
135.75
133.52
131.07
128.34
125.70
124.33
123.66
108.44
106.03
96.55
92.77
73.43
61.04
56.57
56.54
55.95
CH3
CH3
CH3
H3C
H3C
O
O
O
O
O
O
O
NH
N
N
O
O
Cl
Cl
200
190
180
170
160
150
140
130
120
110
100
90
80
70
60
50
40
30
20
10
0
f1 (ppm)

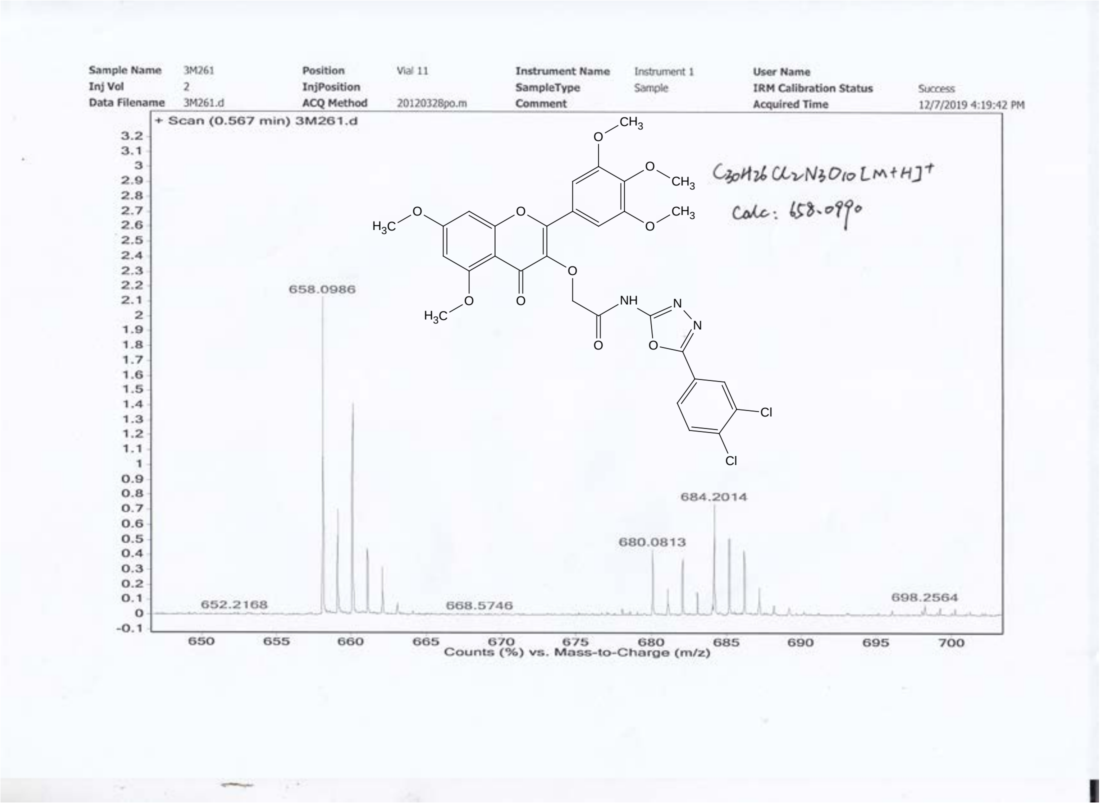

The spectrum of ^{1}H NMR, ^{13}C NMR and HRMS of compound A25

A26

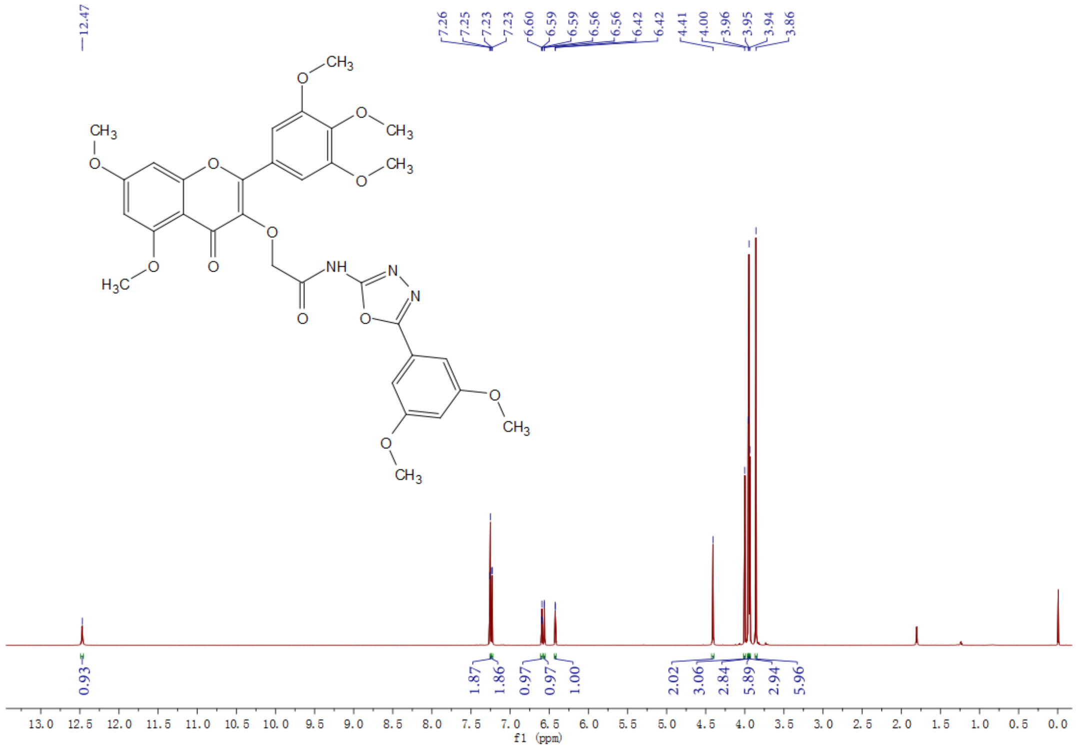

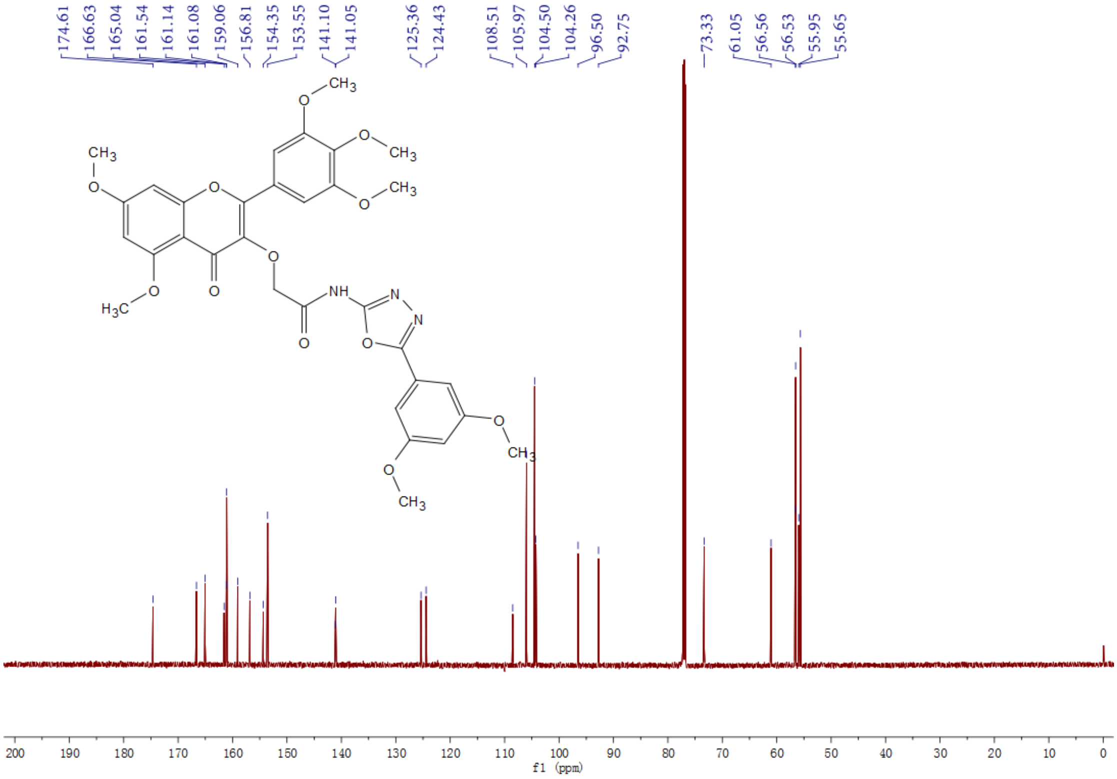

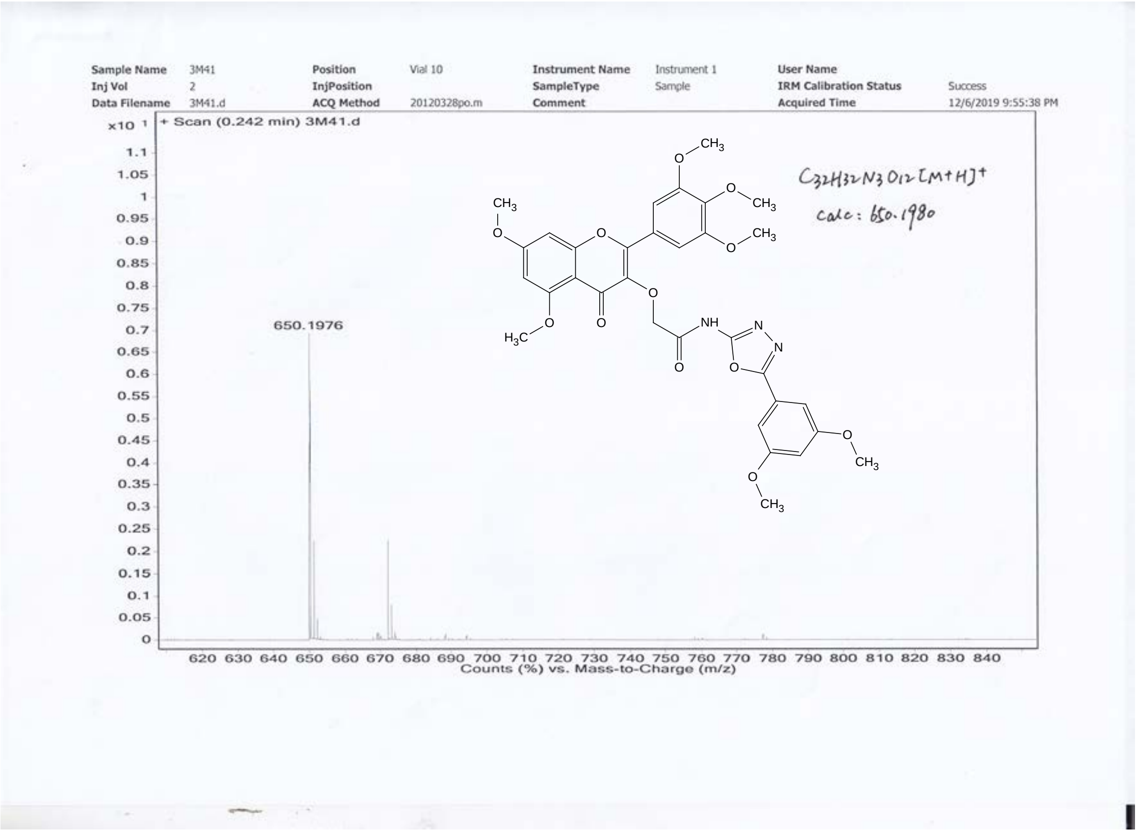

The spectrum of ^{1}H NMR, ^{13}C NMR and HRMS of compound A26

A27

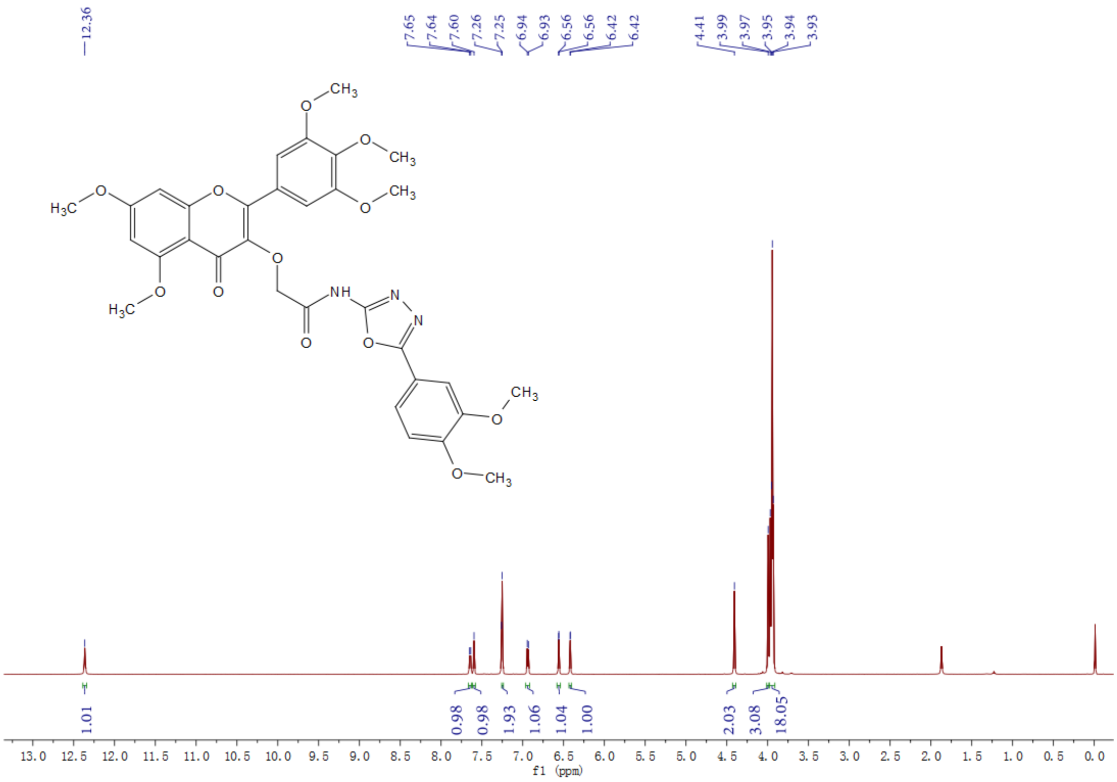

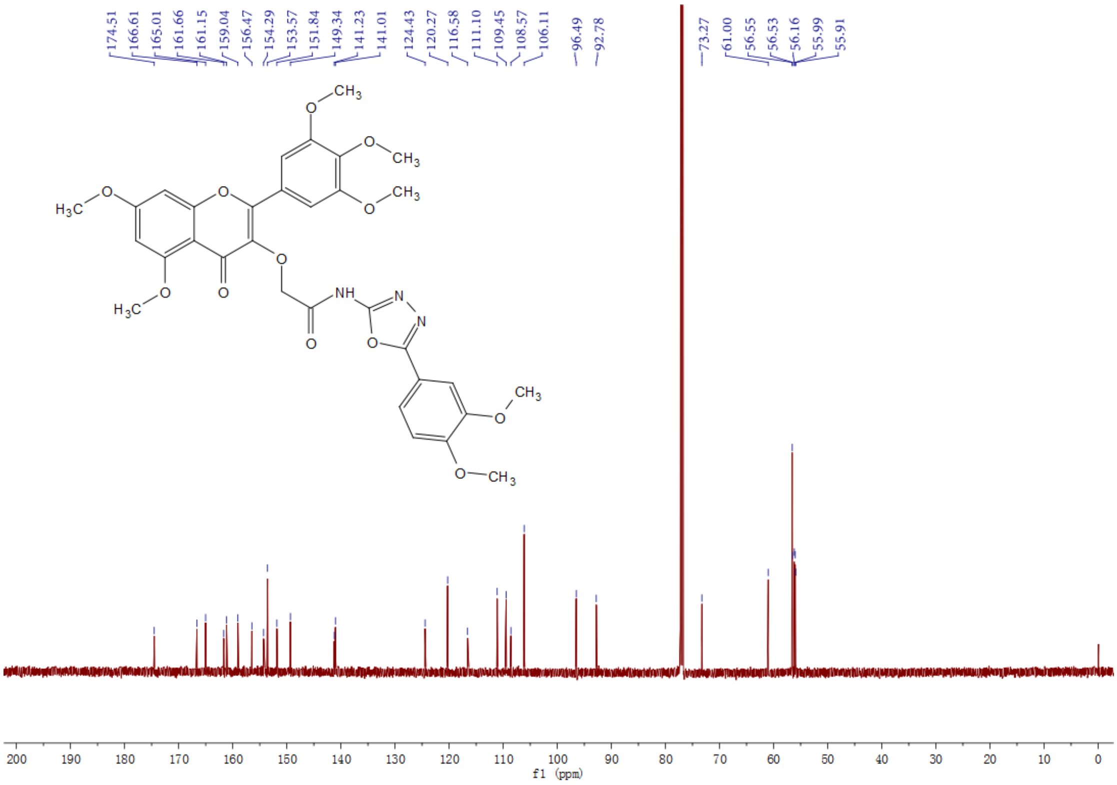

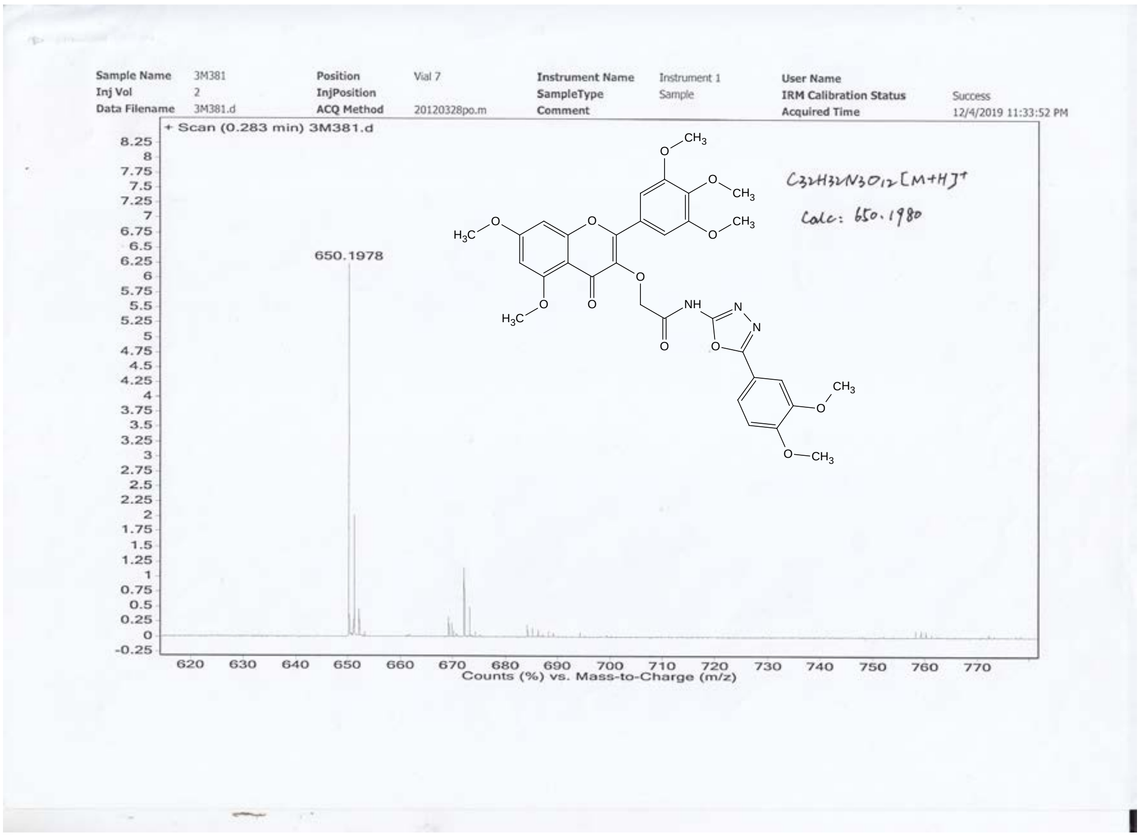

The spectrum of ^{1}H NMR, ^{13}C NMR and HRMS of compound A27

A28

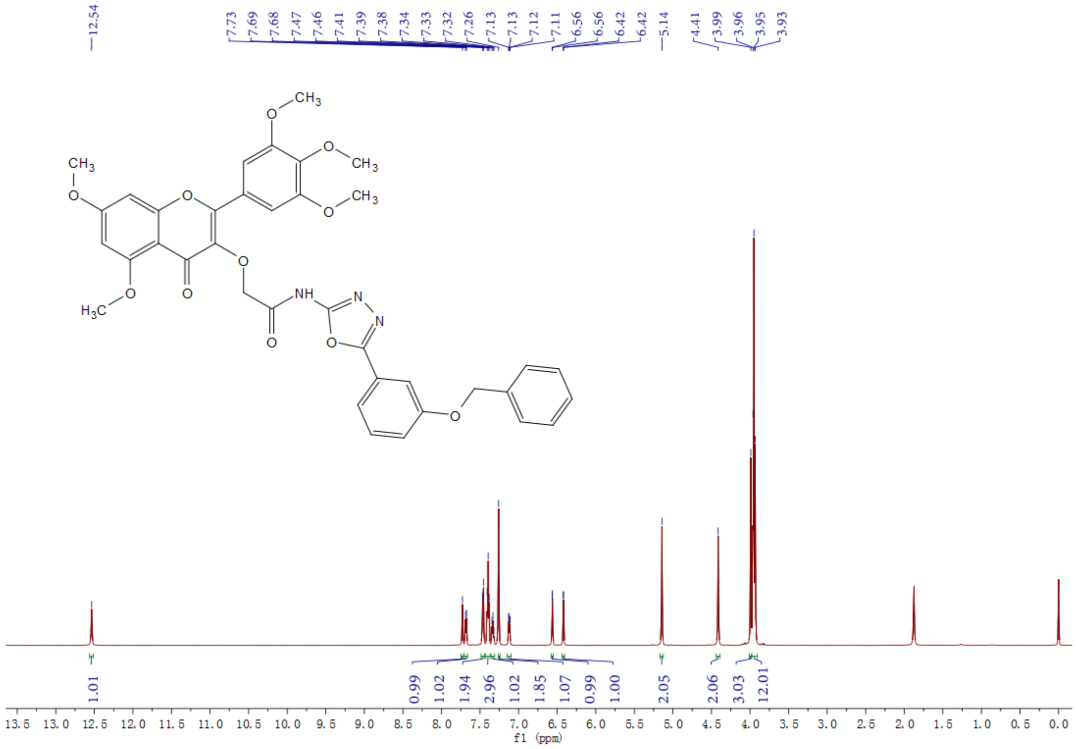

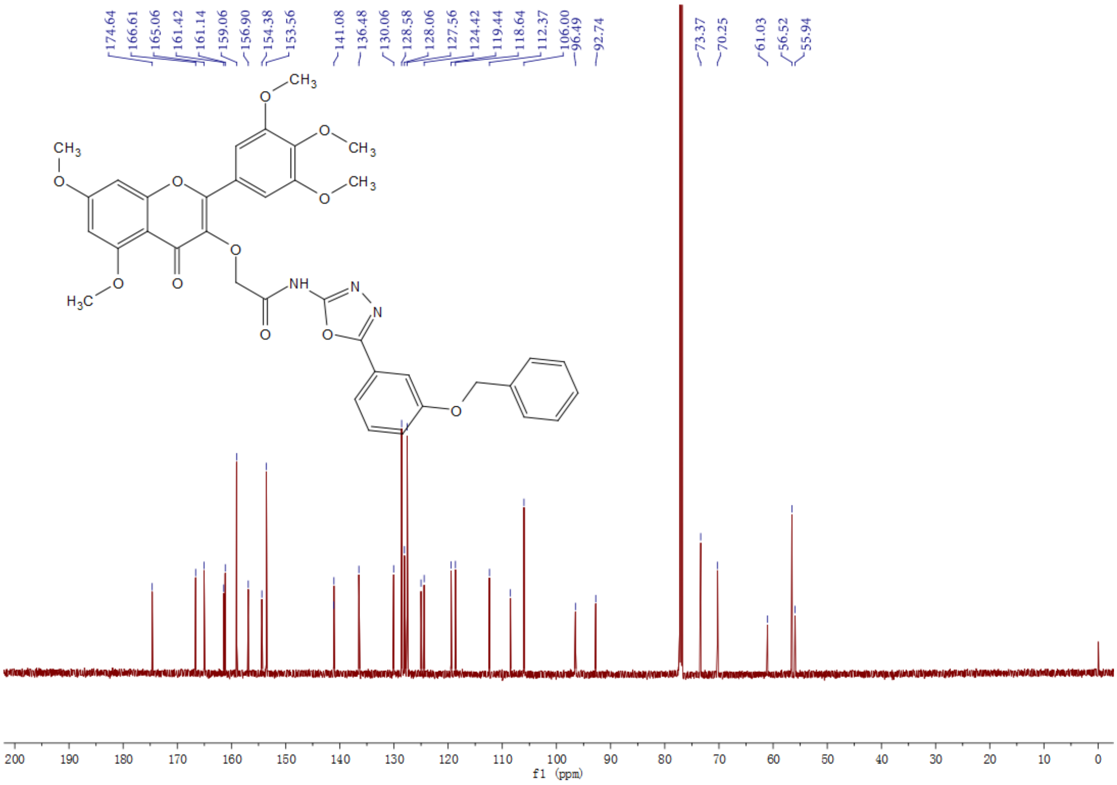

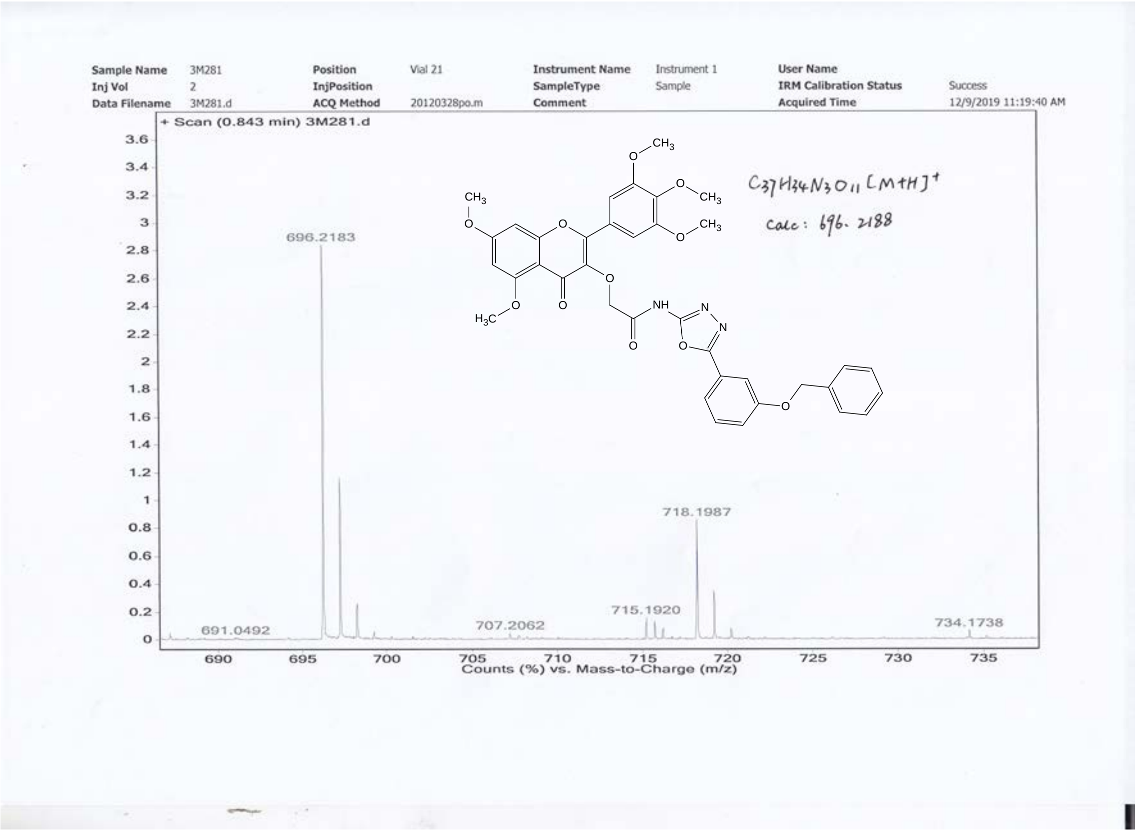

The spectrum of 1H NMR, 13C NMR and HRMS of compound A28

A29

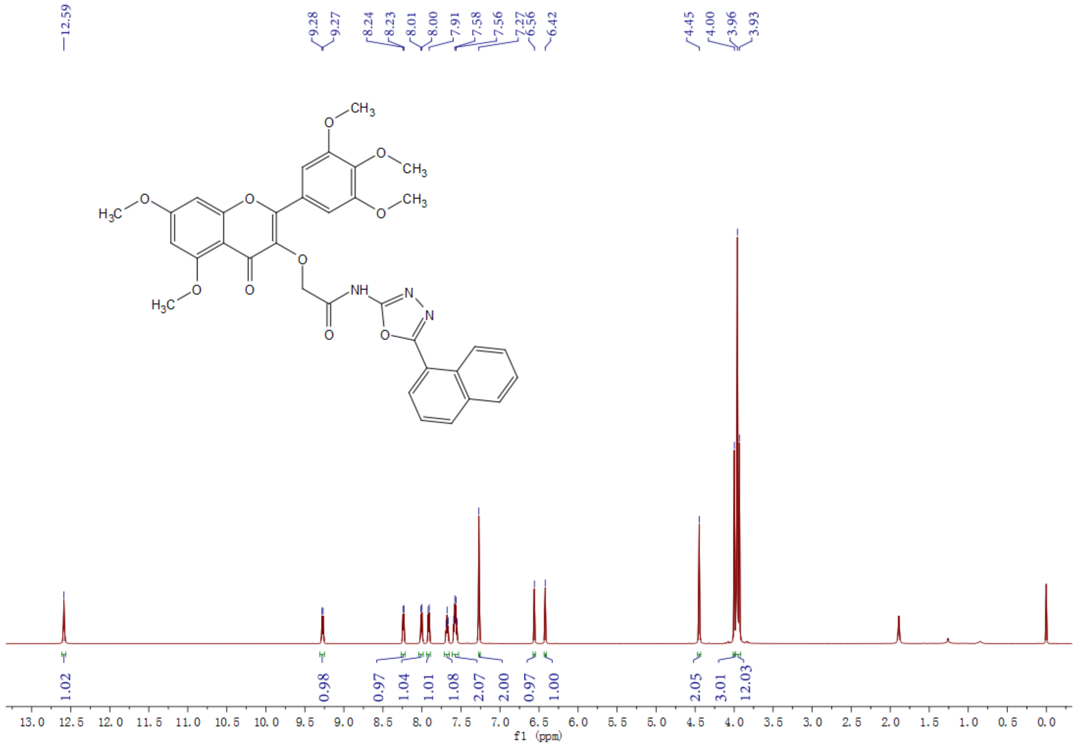
12.59
9.28
9.27
8.24
8.23
8.01
8.00
7.91
7.58
7.56
7.27
6.56
6.42
4.45
4.00
3.96
3.93
1.02
0.98
0.97
1.04
1.01
1.08
2.07
2.00
0.97
1.00
2.05
3.01
12.03
f1 (ppm)

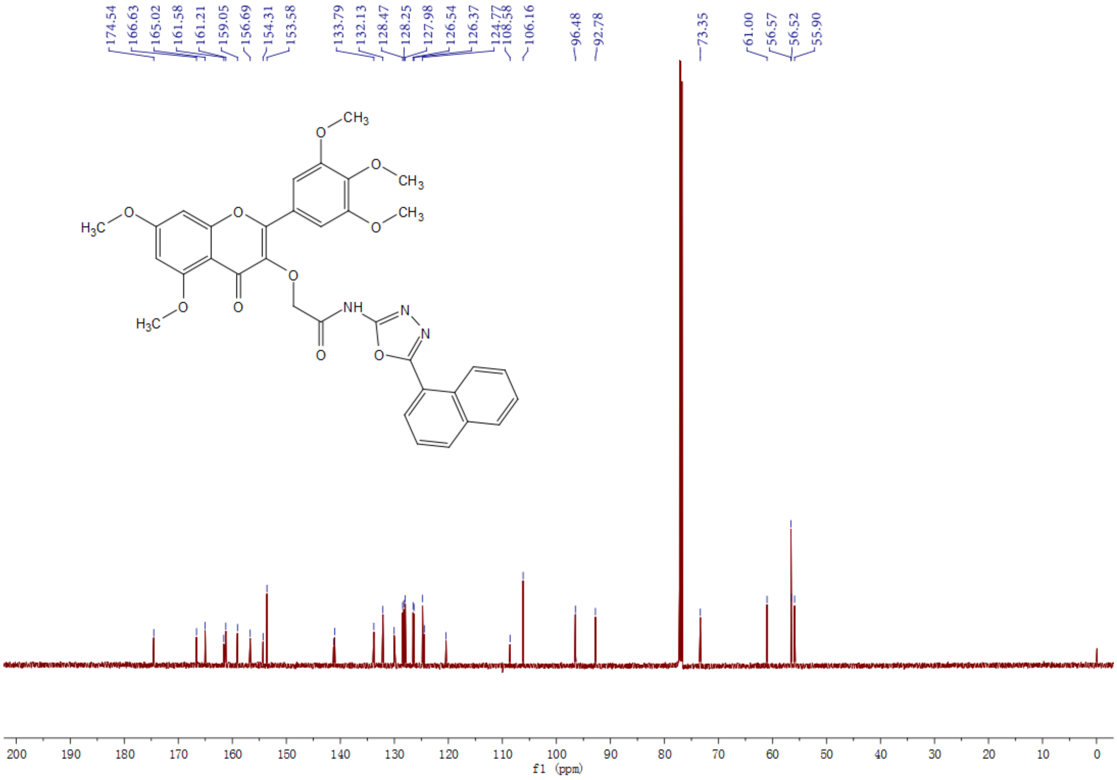
174.54
166.63
165.02
161.58
161.21
159.05
156.69
154.31
153.58
133.79
132.13
128.47
128.25
127.98
126.54
126.37
124.77
108.58
106.16
96.48
92.78
73.35
61.00
56.57
56.52
55.90
f1 (ppm)

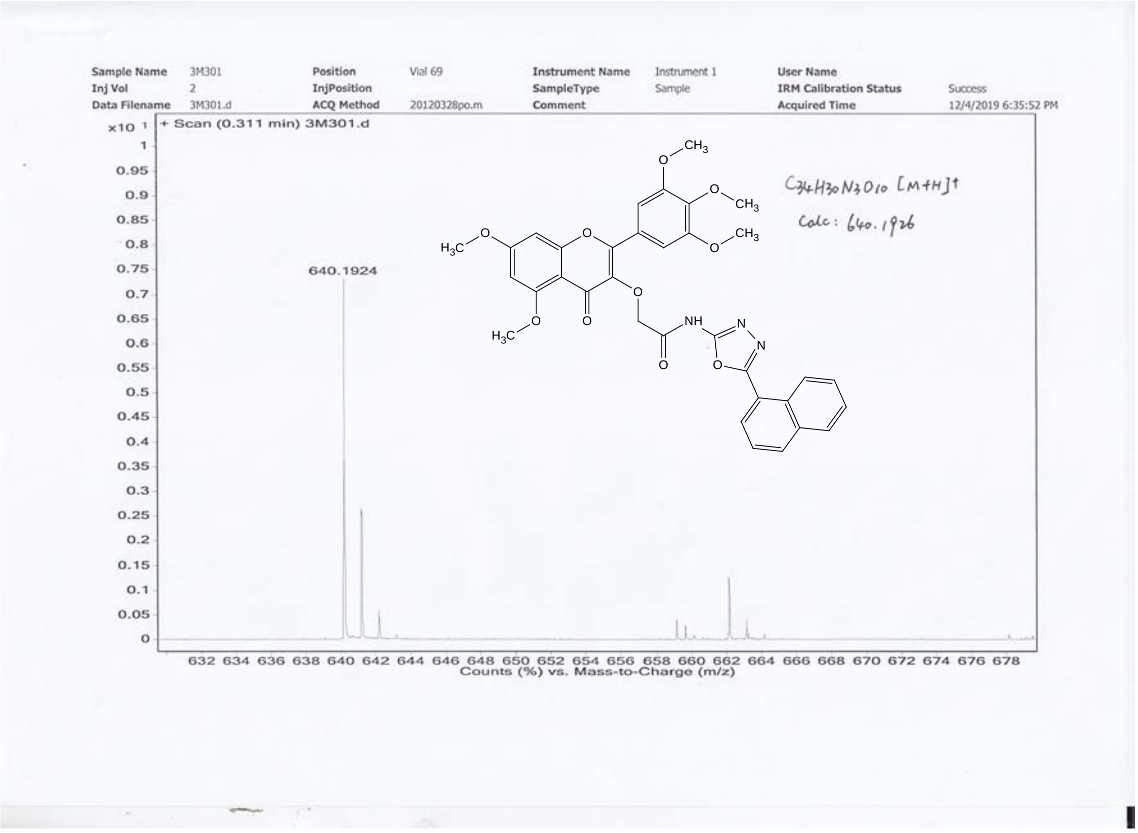

The spectrum of ^{1}H NMR, ^{13}C NMR and HRMS of compound A29

A30

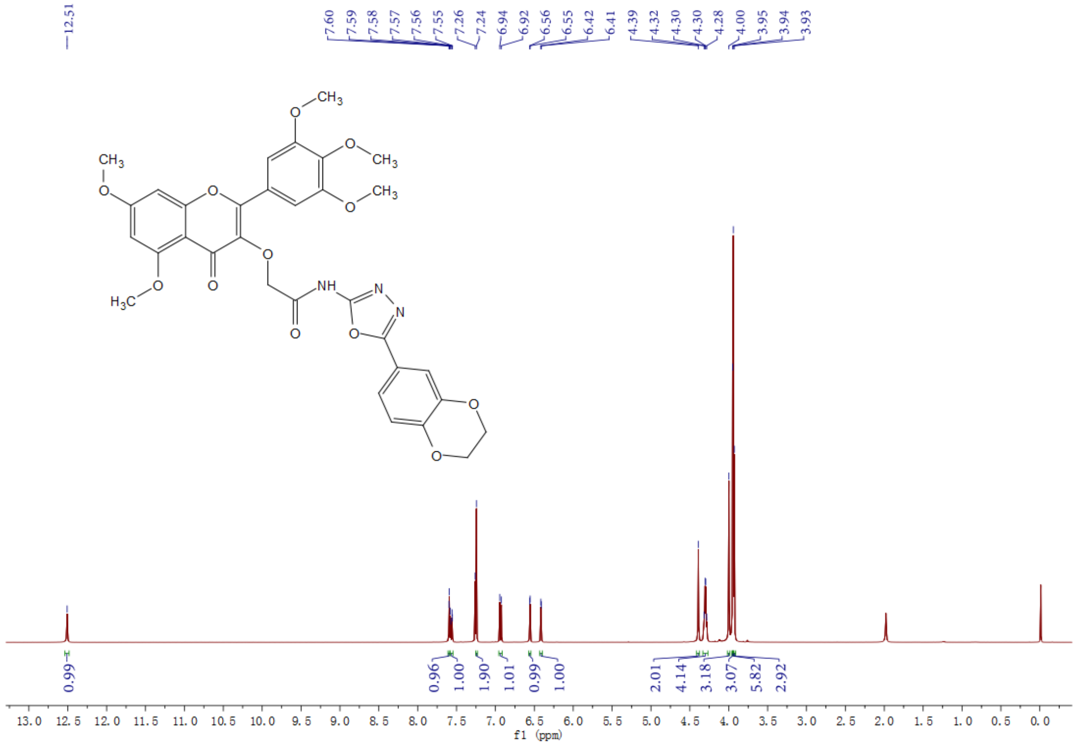

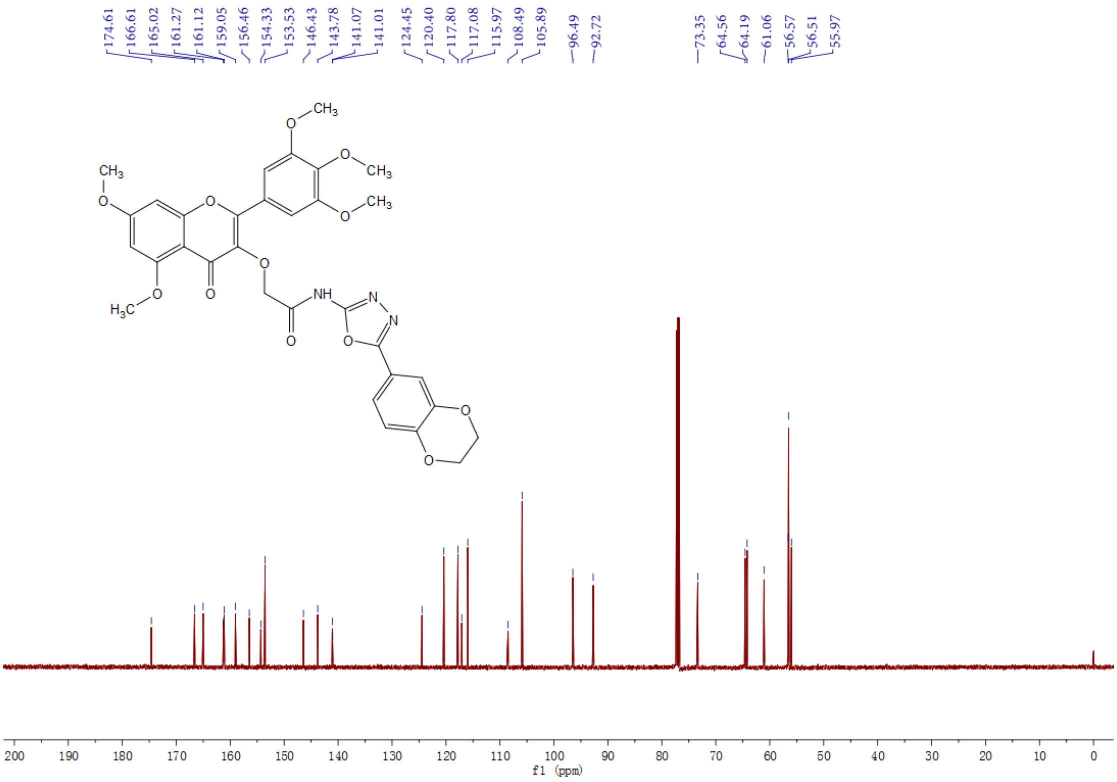

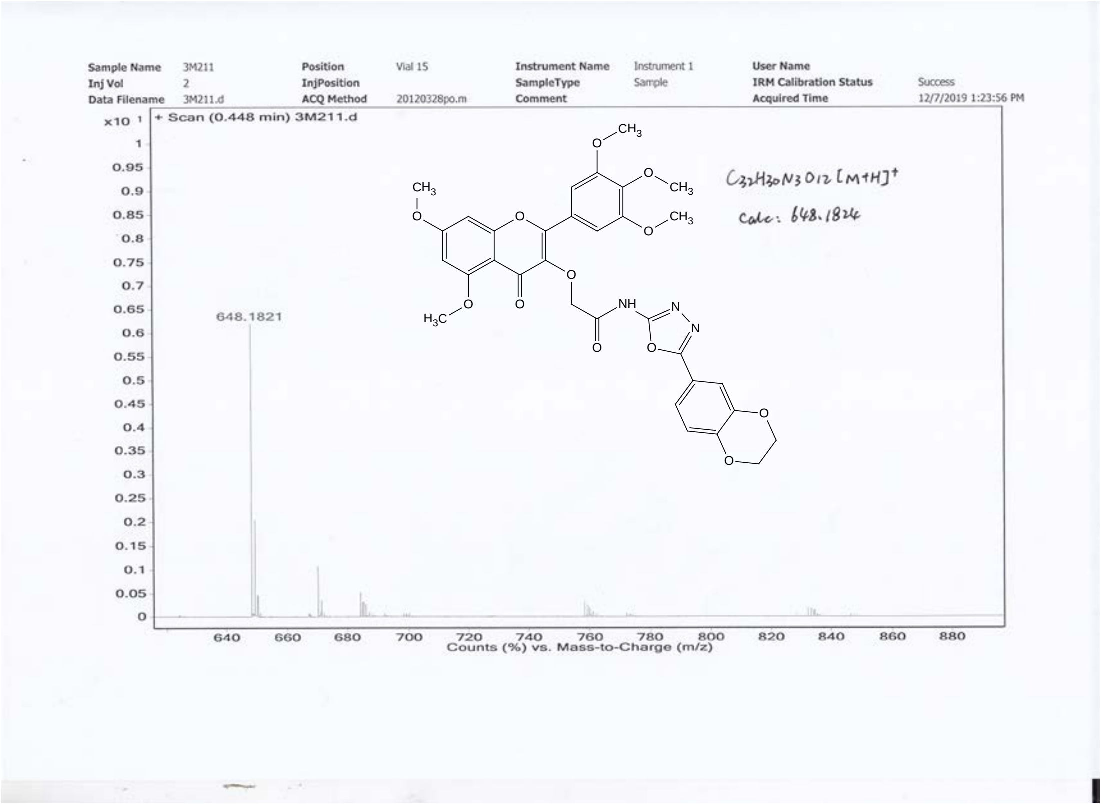

The spectrum of ^{1}H NMR, ^{13}C NMR and HRMS of compound A30

A31

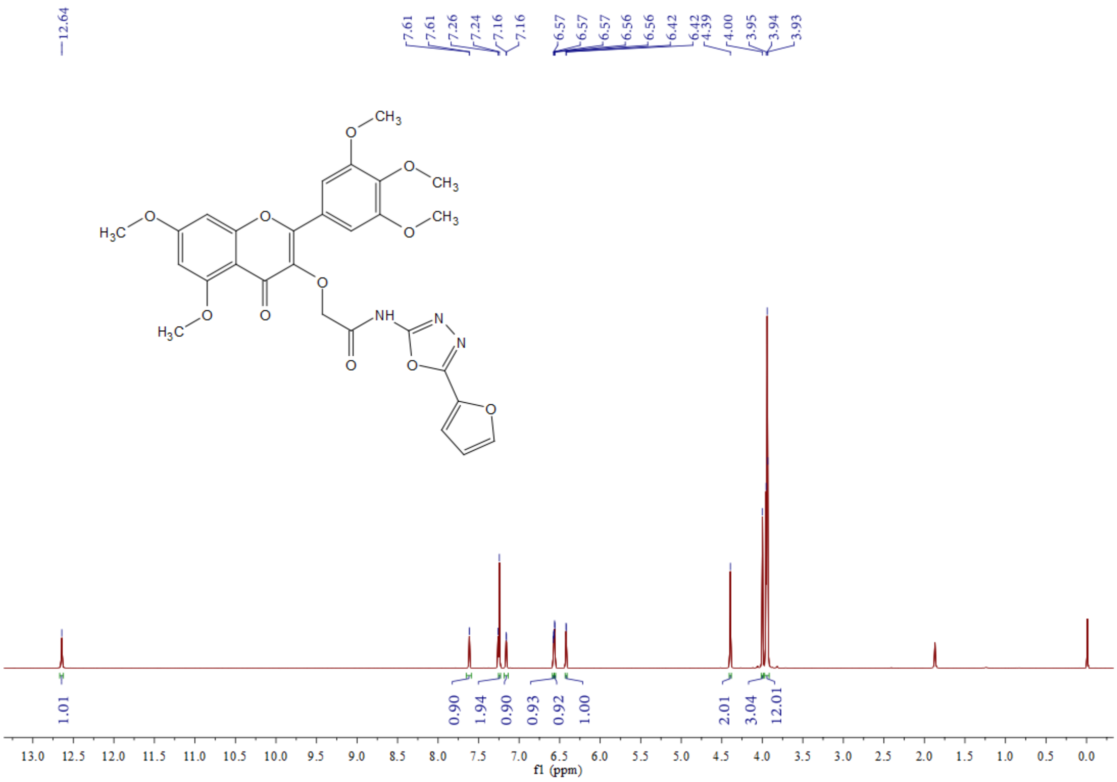

12.64
7.61
7.61
7.26
7.24
7.16
7.16
6.57
6.57
6.57
6.56
6.56
6.42
6.42
4.39
4.00
3.95
3.94
3.93
1.01
0.90
1.94
0.90
0.93
0.92
1.00
2.01
3.04
12.01
f1 (ppm)

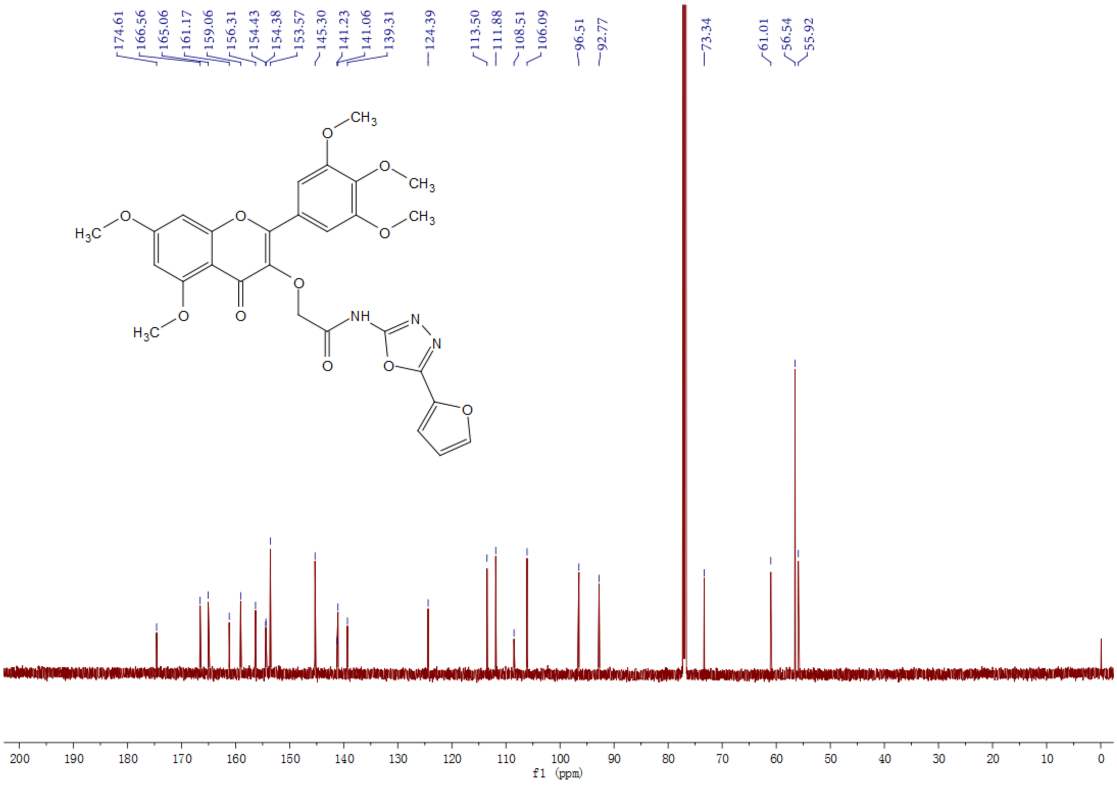

174.61
166.56
165.06
161.17
159.06
156.31
154.43
154.38
153.57
145.30
141.23
141.06
139.31
124.39
113.50
111.88
108.51
106.09
96.51
92.77
73.34
61.01
56.54
55.92
f1 (ppm)

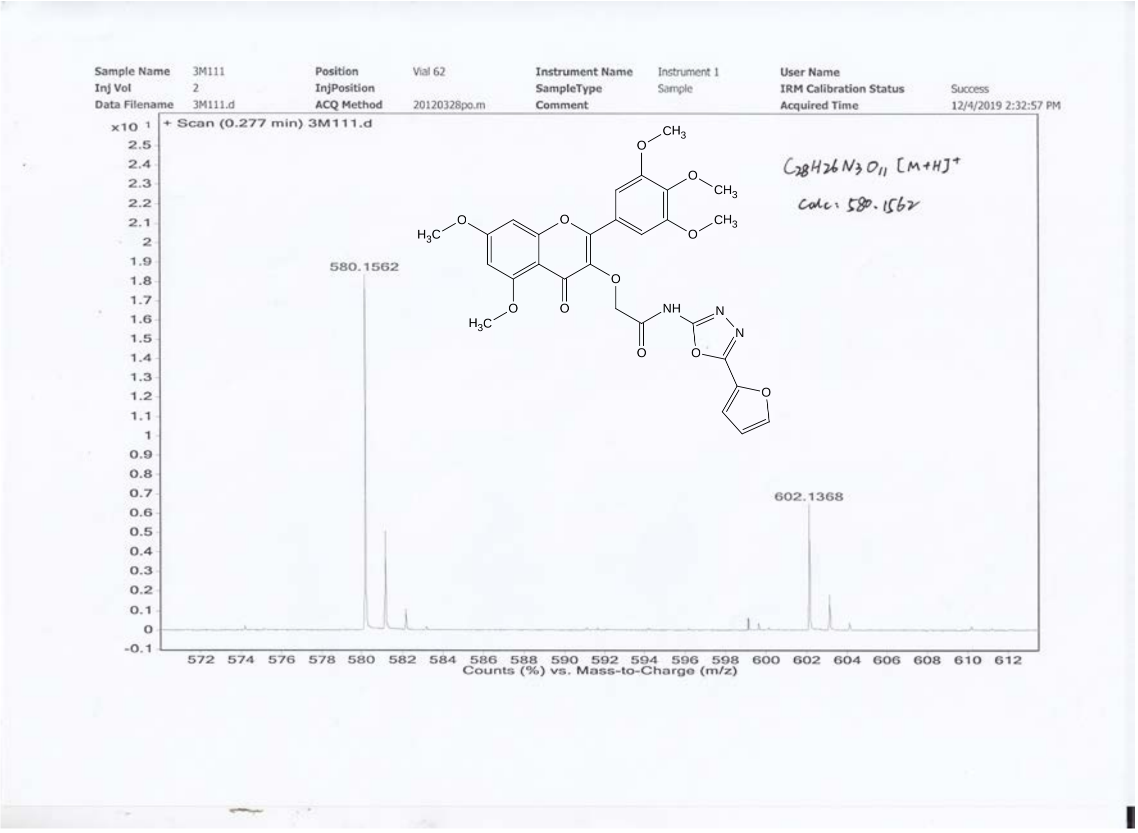

The spectrum of ^{1}H NMR, ^{13}C NMR and HRMS of compound A31

A32

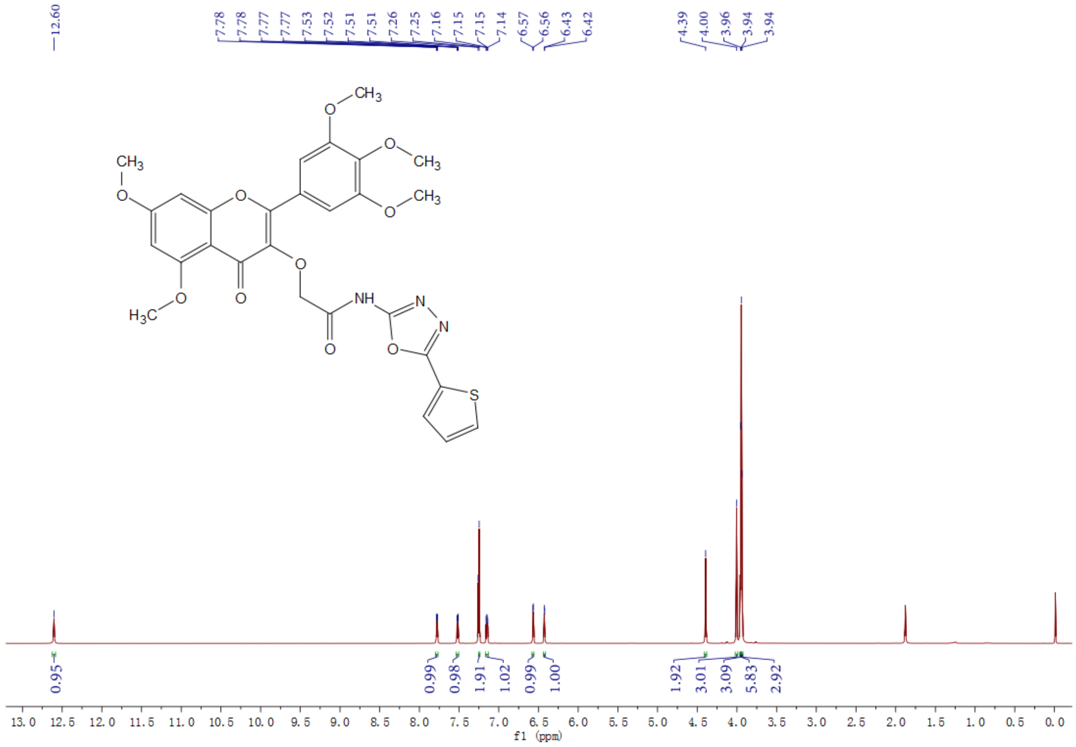

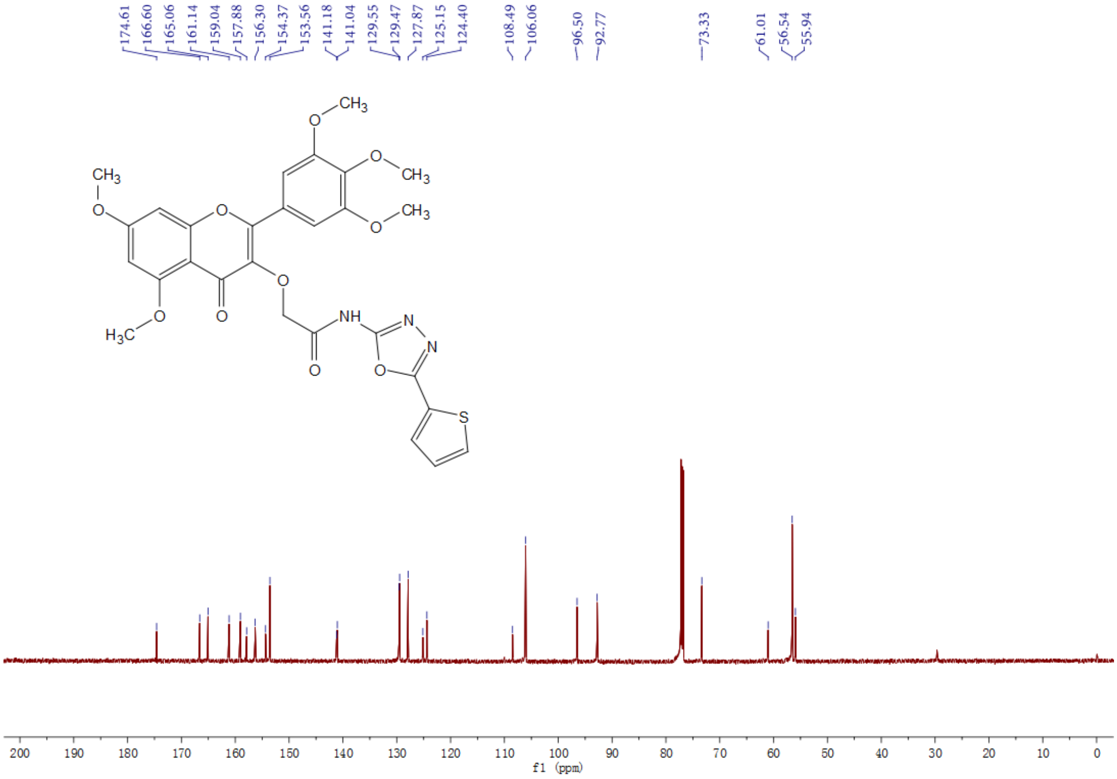

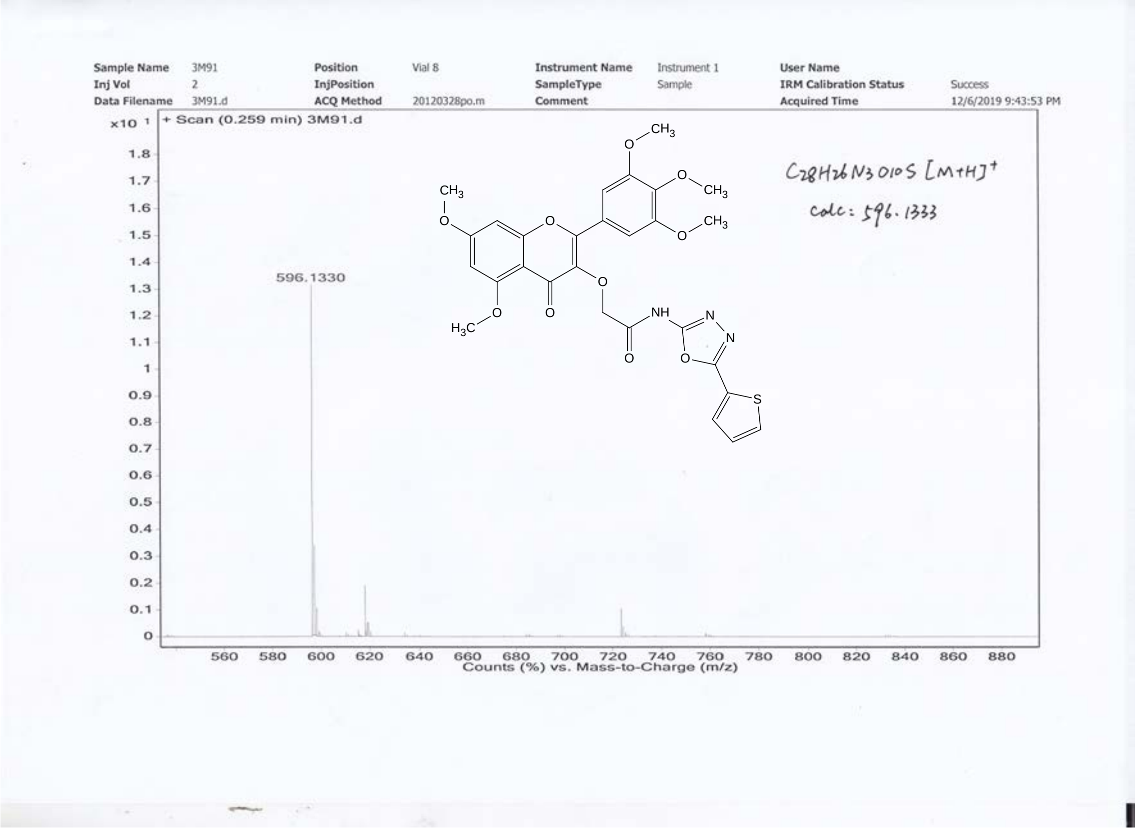

The spectrum of ¹H NMR, ¹³C NMR and HRMS of compound A32

A33

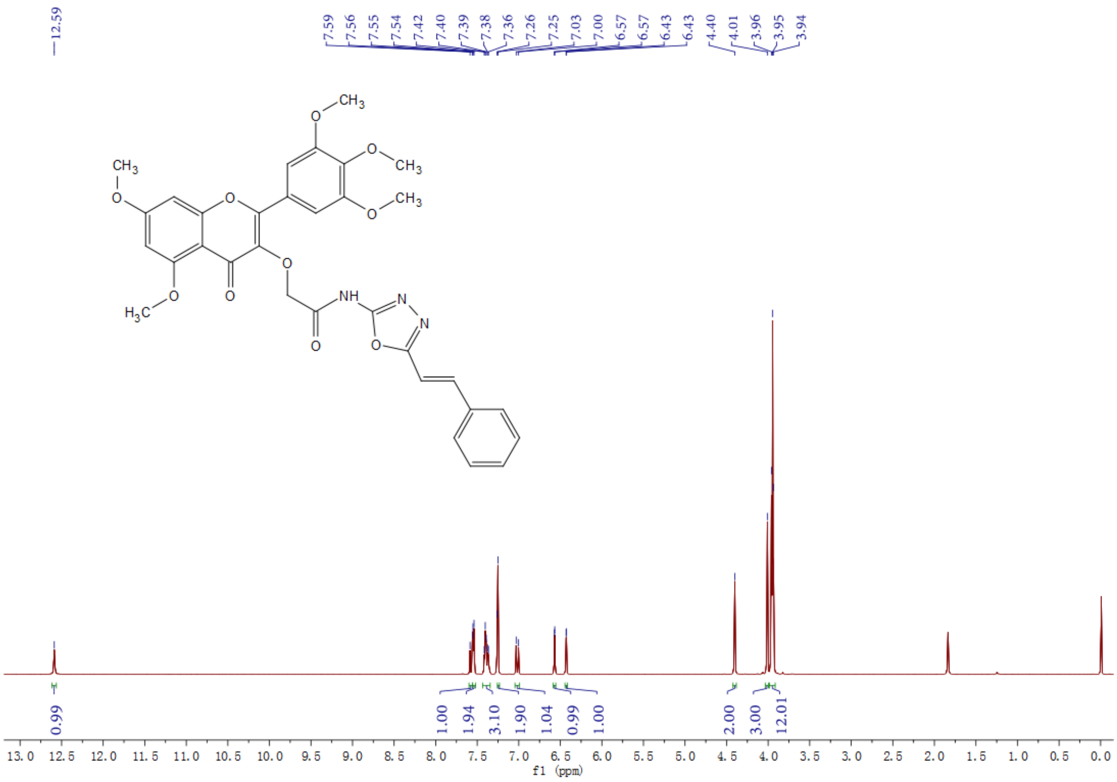

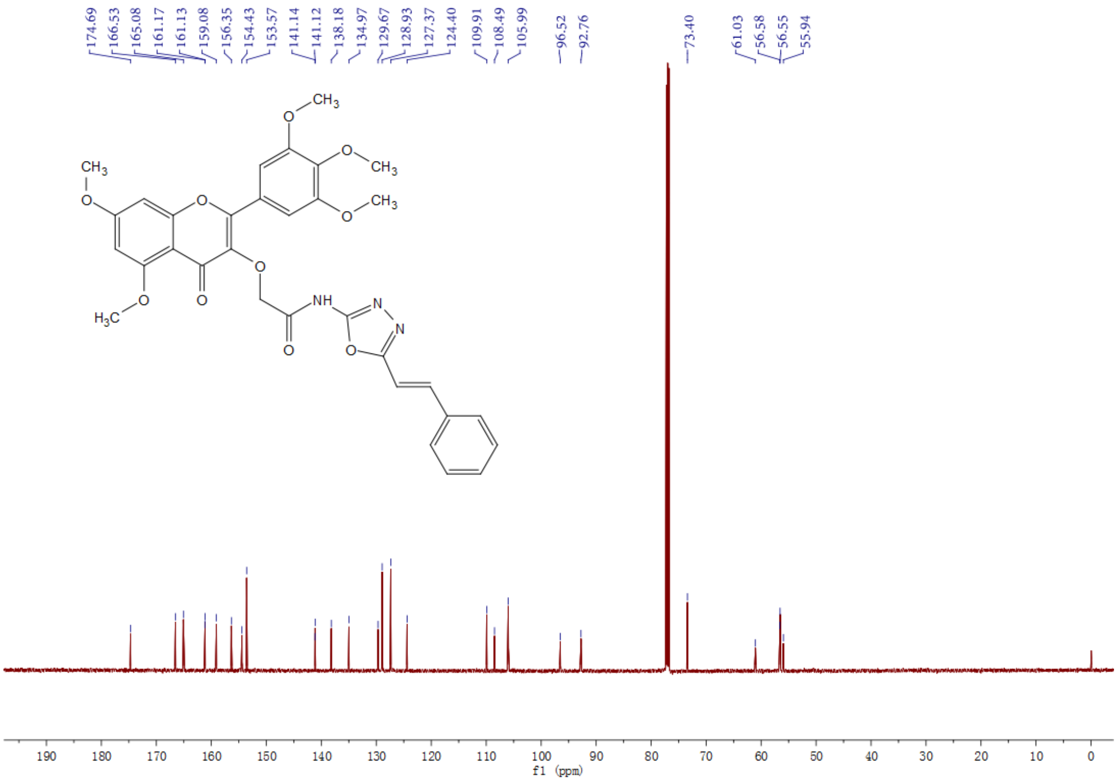

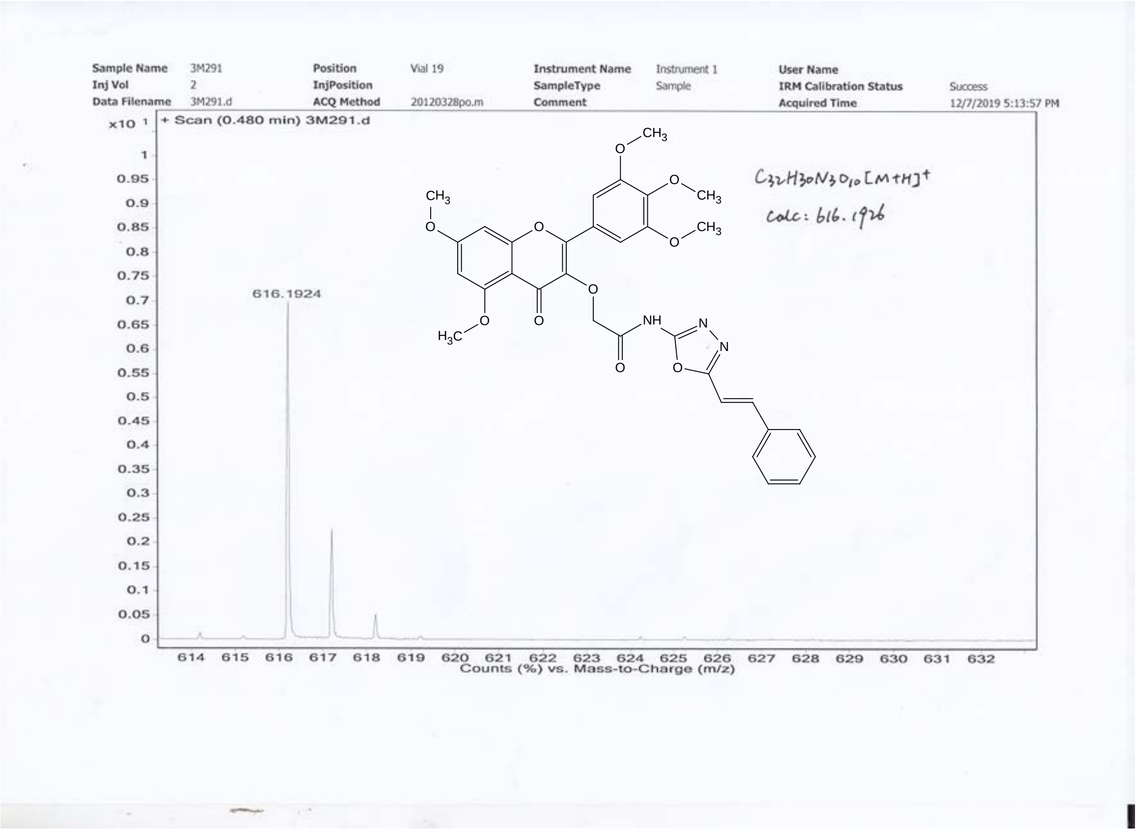

The spectrum of ¹H NMR, ¹³C NMR and HRMS of compound A33

B1

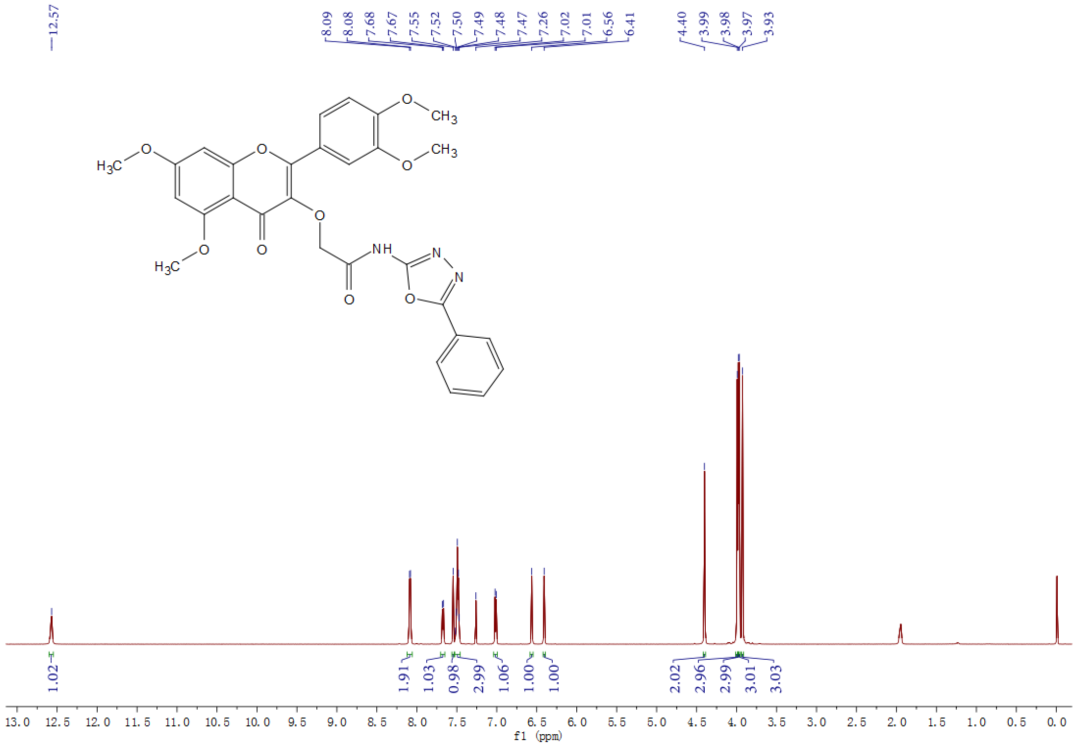

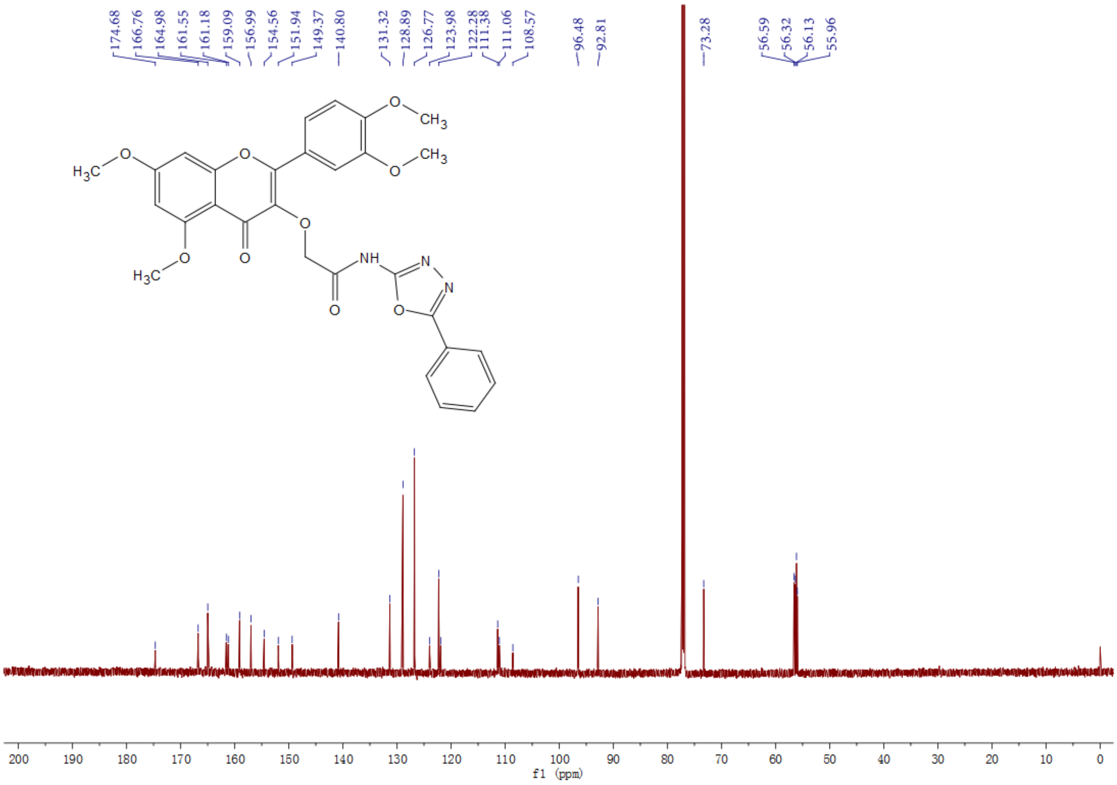

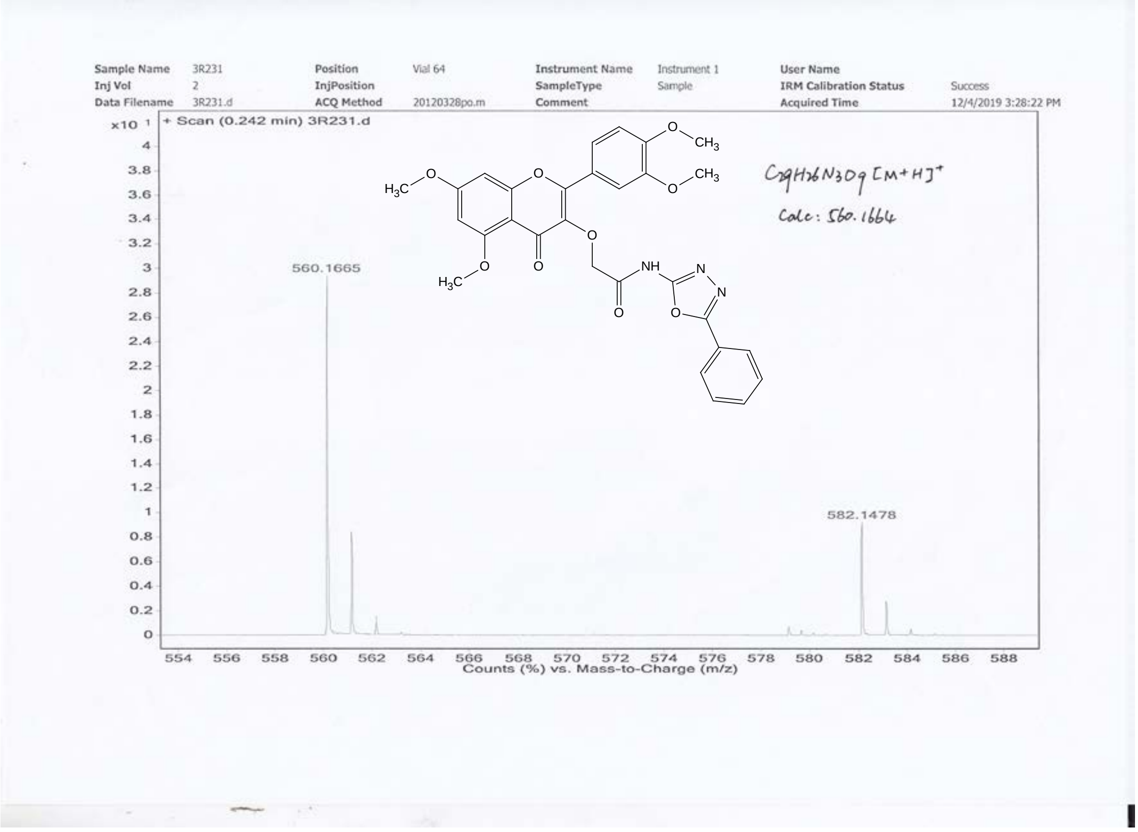

The spectrum of ^{1}H NMR, ^{13}C NMR and HRMS of compound B1

B2

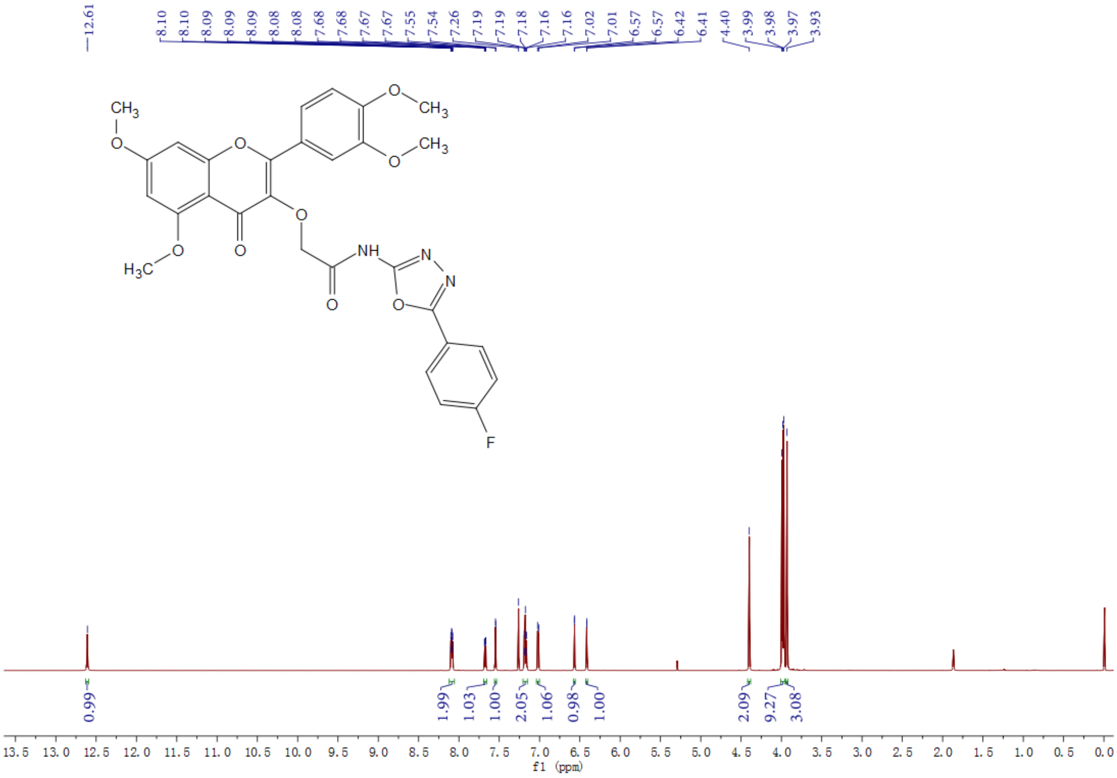

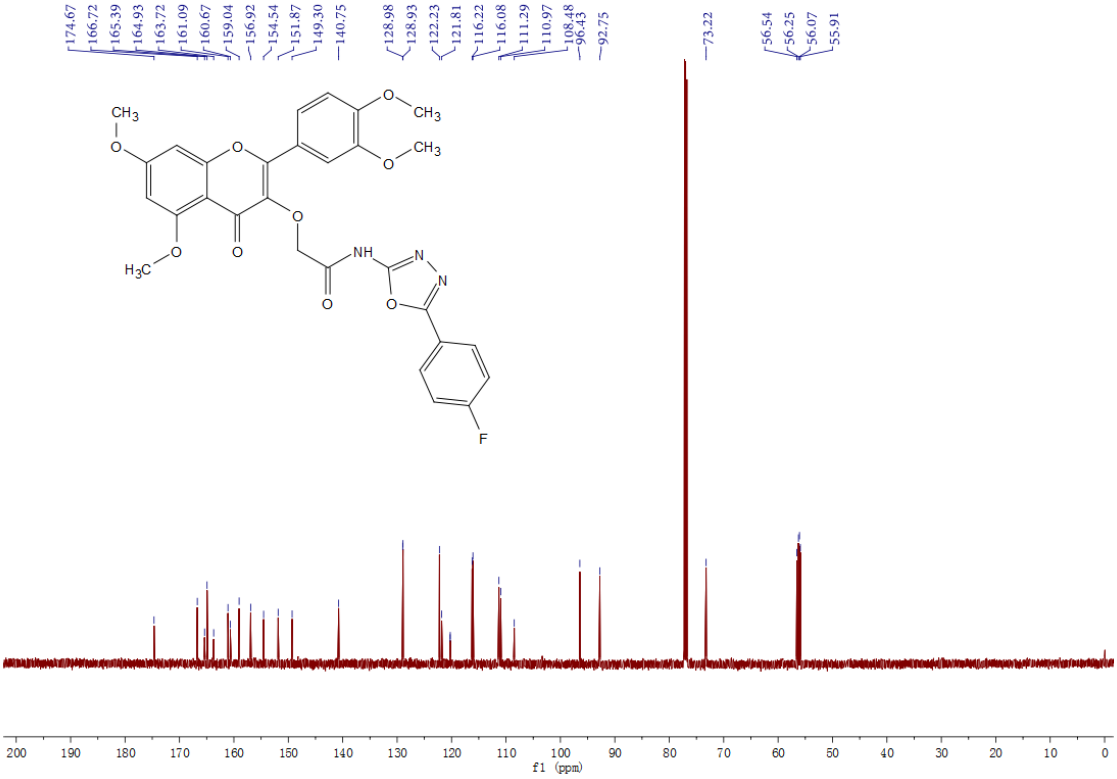

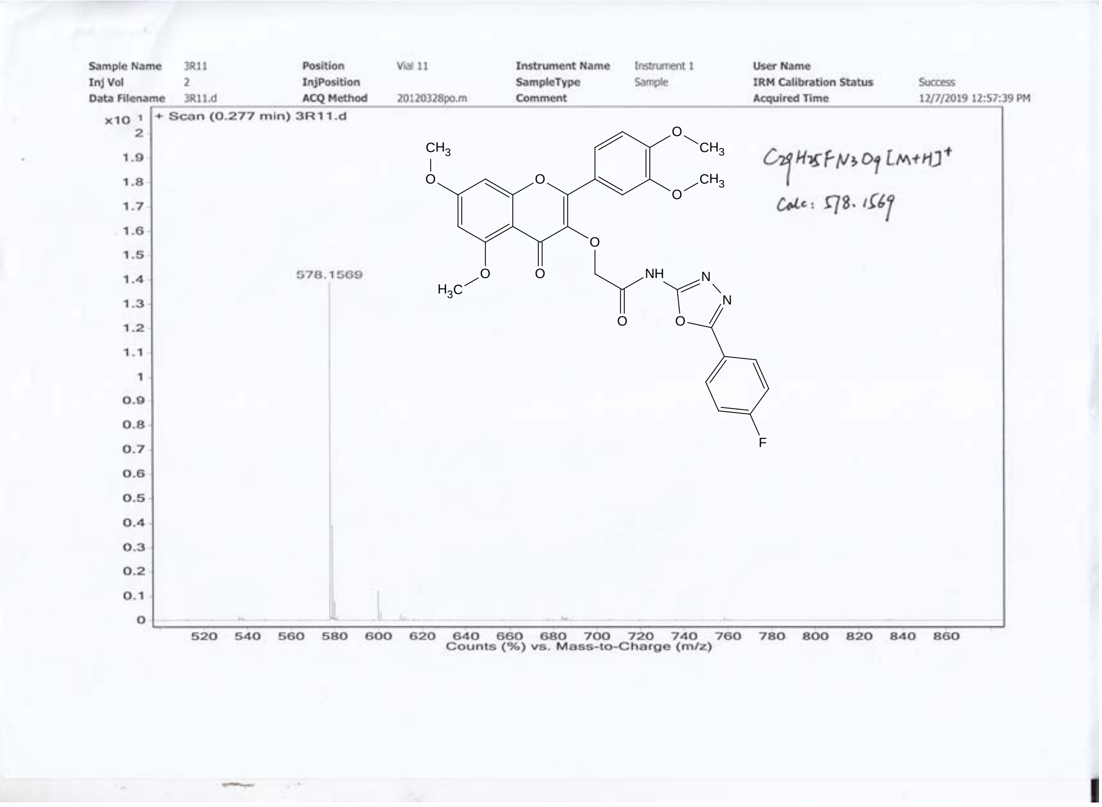

The spectrum of ¹H NMR, ¹³C NMR and HRMS of compound B2

B3

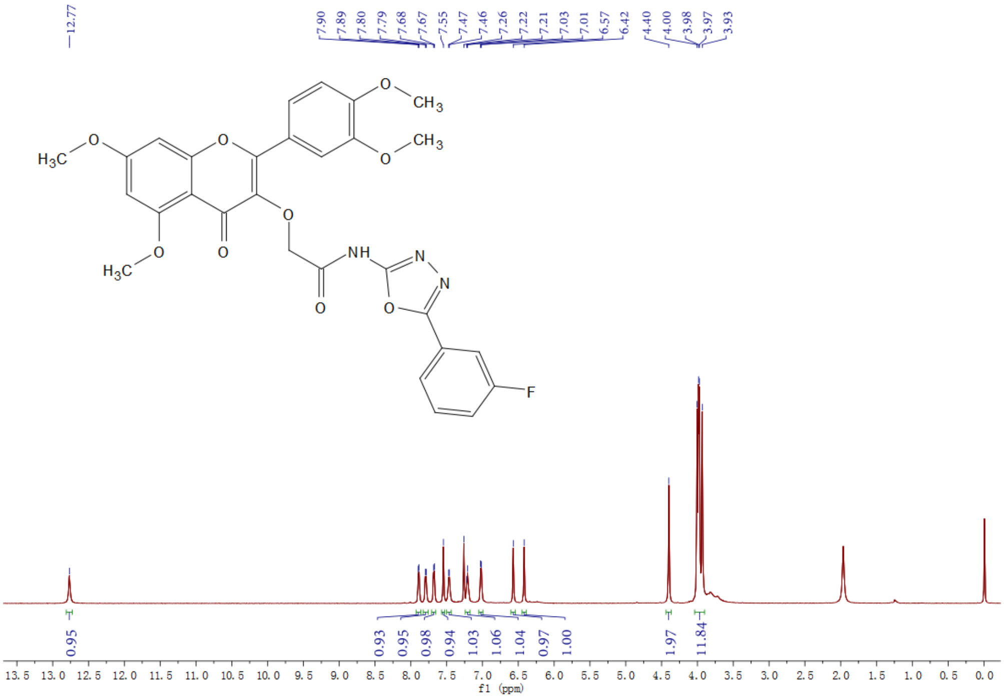

¹H NMR spectrum of compound B3

B4

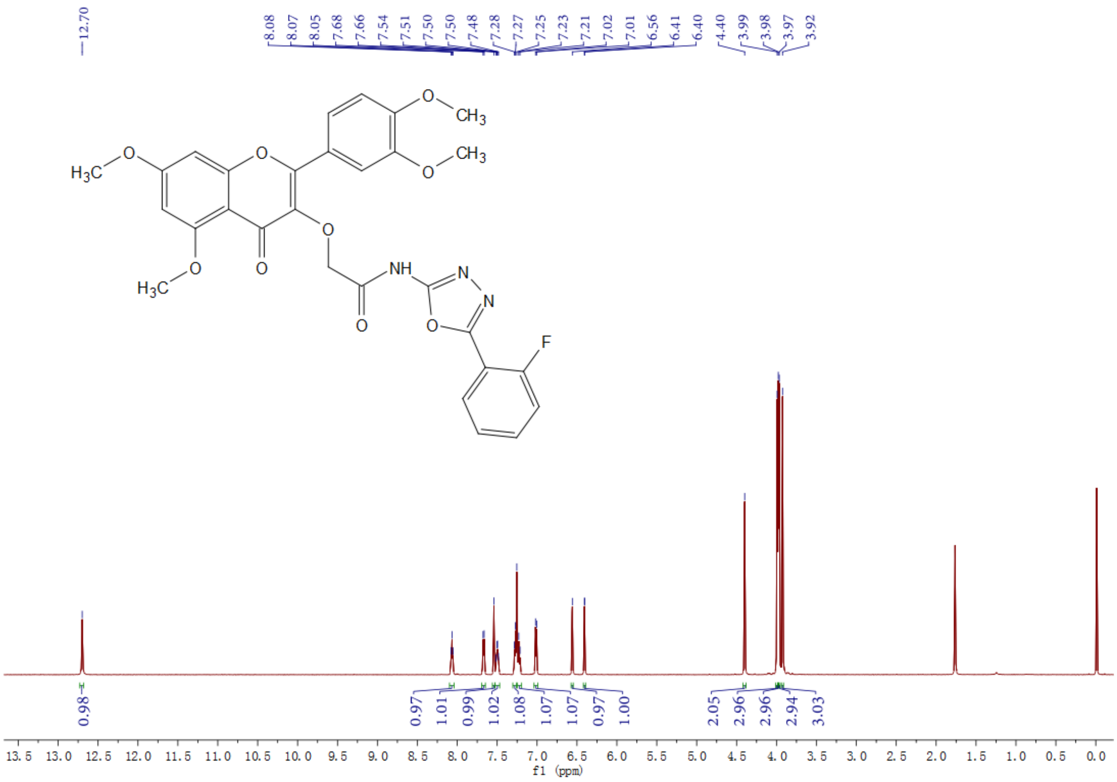

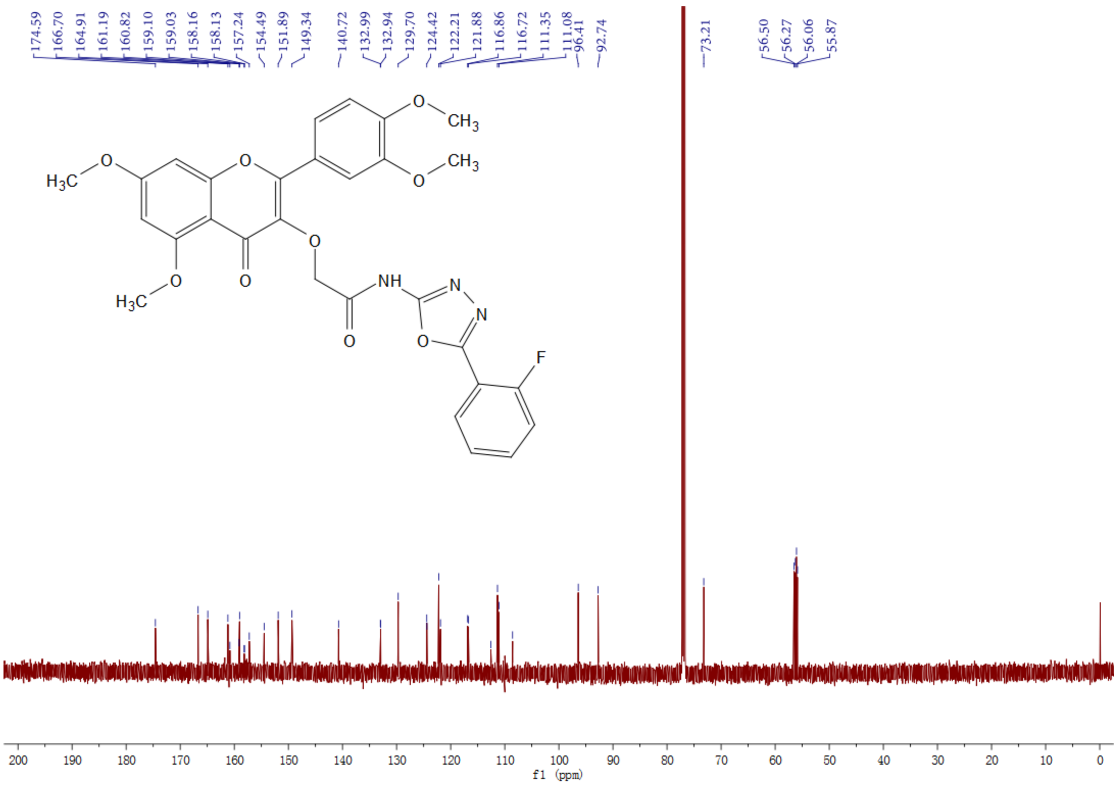

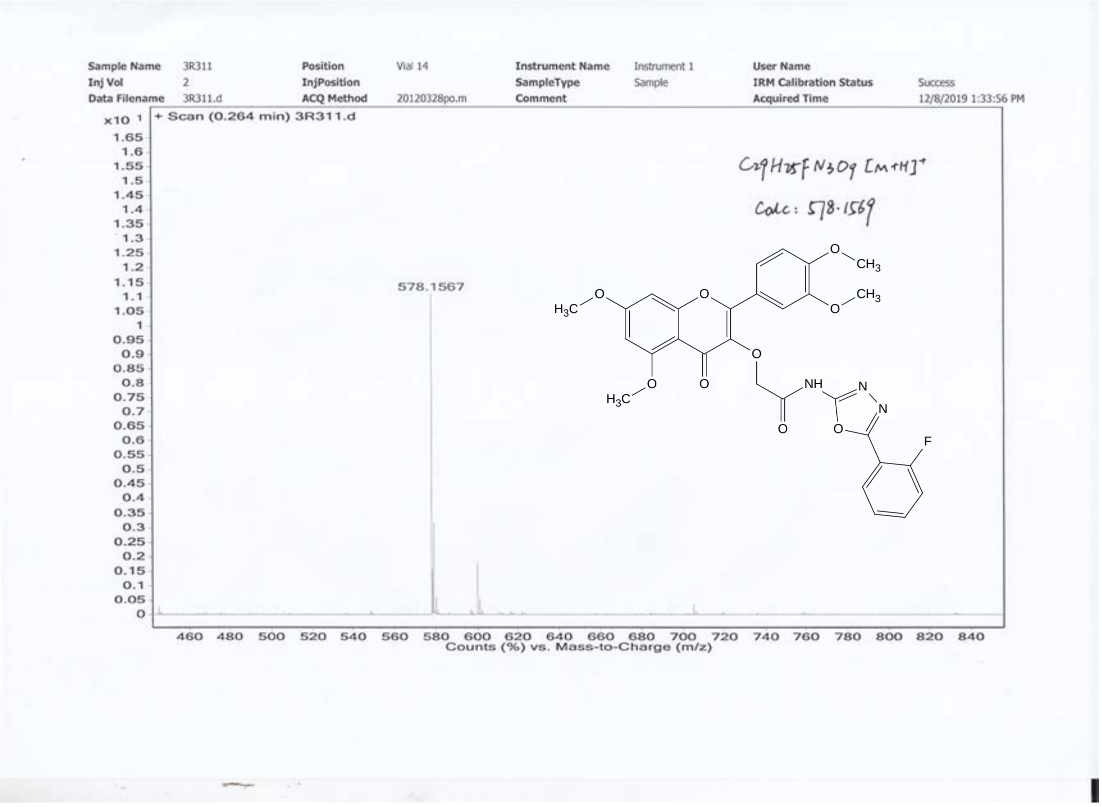

The spectrum of ^{1}H NMR, ^{13}C NMR and HRMS of compound B4

B5

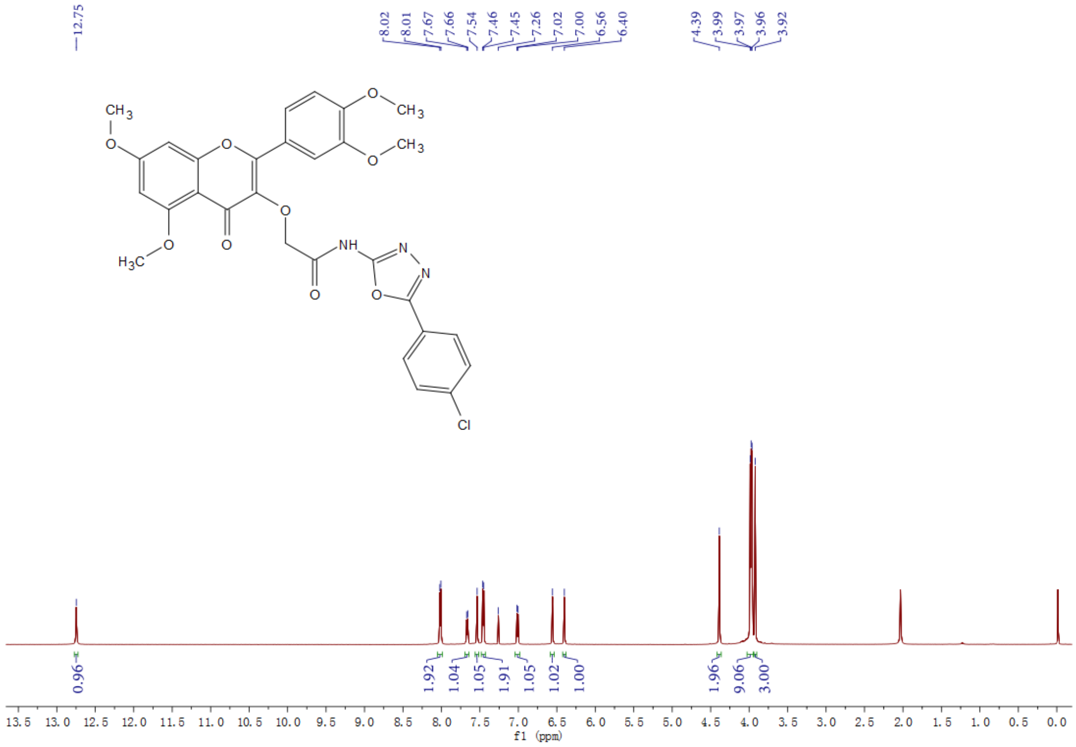

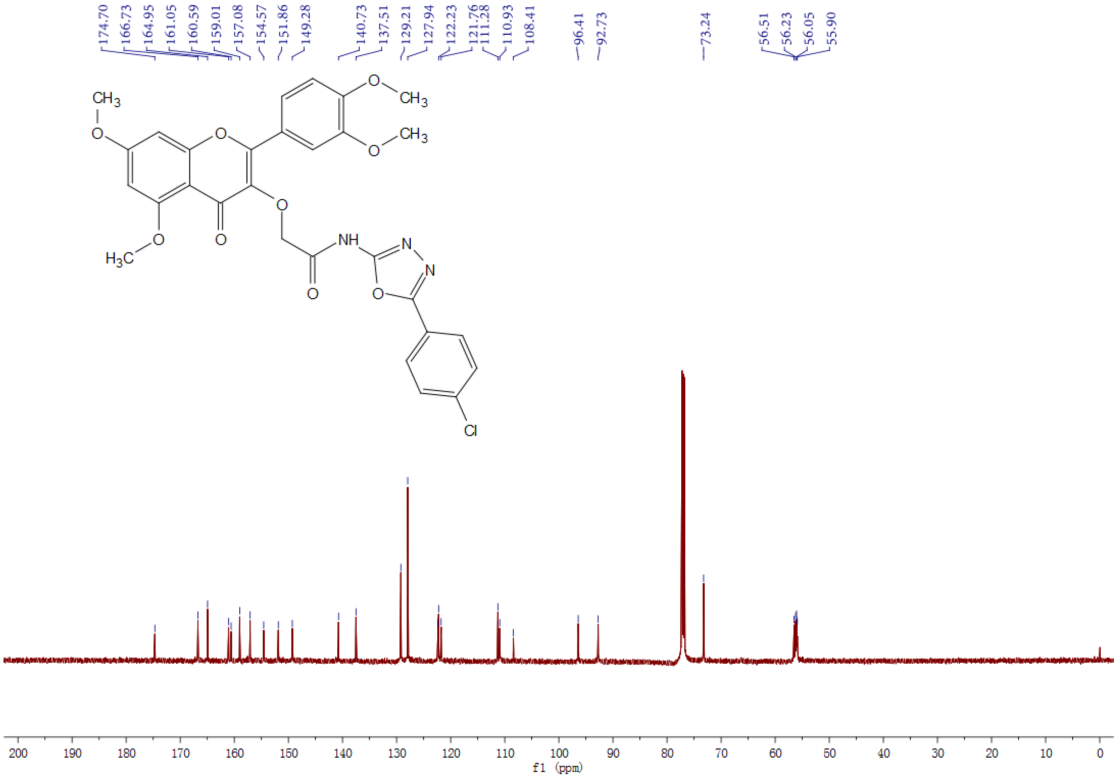

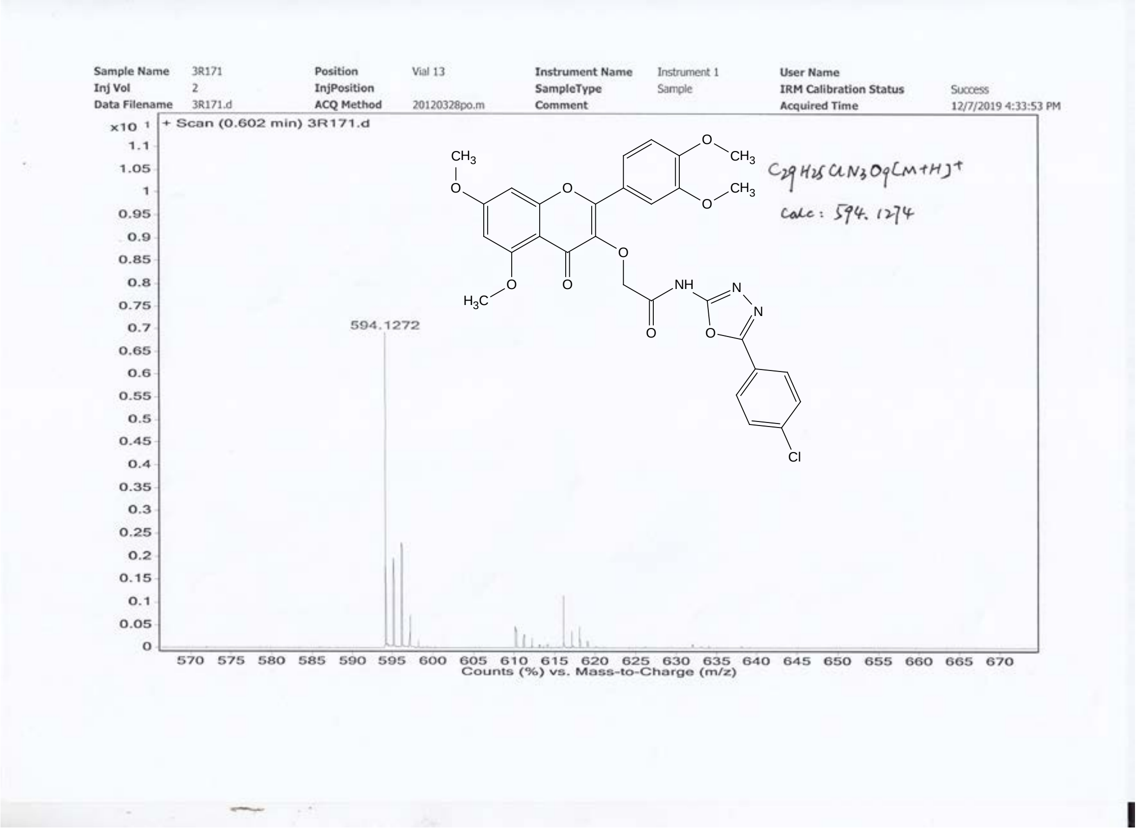

The spectrum of ^{1}H NMR, ^{13}C NMR and HRMS of compound B5

B6

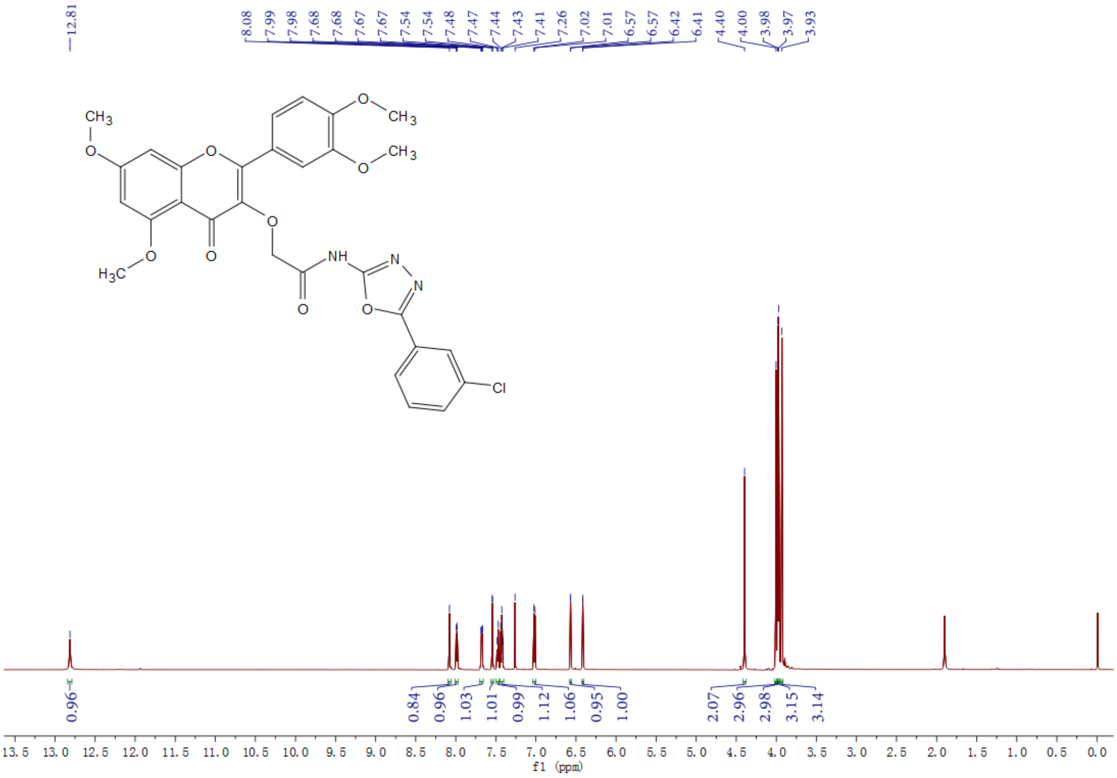
12.81
8.08
7.99
7.98
7.68
7.68
7.67
7.67
7.54
7.54
7.48
7.47
7.44
7.43
7.41
7.26
7.02
7.01
6.57
6.57
6.42
6.41
4.40
4.00
3.98
3.97
3.93
CH3
O
O
CH3
O
CH3
O
O
H3C
O
O
NH
N
N
O
O
Cl
0.96
0.84
0.96
1.03
1.01
0.99
1.12
1.06
0.95
1.00
2.07
2.96
2.98
3.15
3.14
13.5 13.0 12.5 12.0 11.5 11.0 10.5 10.0 9.5 9.0 8.5 8.0 7.5 7.0 6.5 6.0 5.5 5.0 4.5 4.0 3.5 3.0 2.5 2.0 1.5 1.0 0.5 0.0
f1 (ppm)

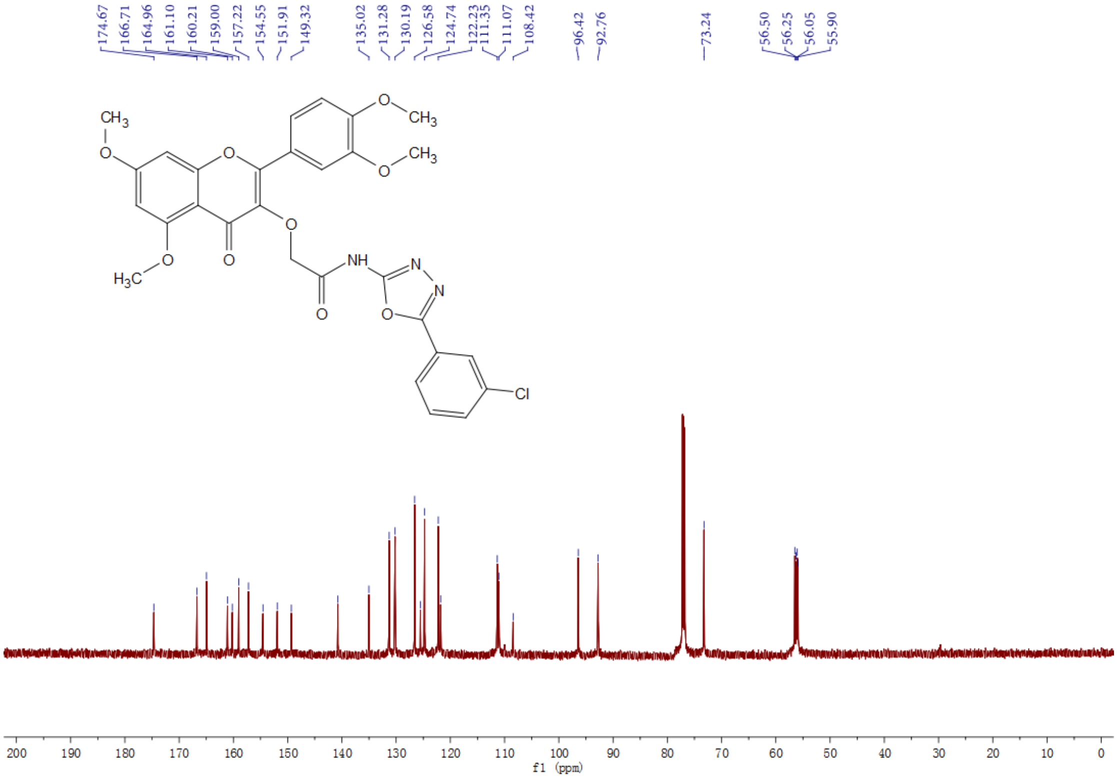
174.67
166.71
164.96
161.10
160.21
159.00
157.22
154.55
151.91
149.32
135.02
131.28
130.19
126.58
124.74
122.23
111.35
111.07
108.42
96.42
92.76
73.24
56.50
56.25
56.05
55.90
CH3
O
O
CH3
O
CH3
O
O
H3C
O
O
NH
N
N
O
O
Cl
200 190 180 170 160 150 140 130 120 110 100 90 80 70 60 50 40 30 20 10 0
f1 (ppm)

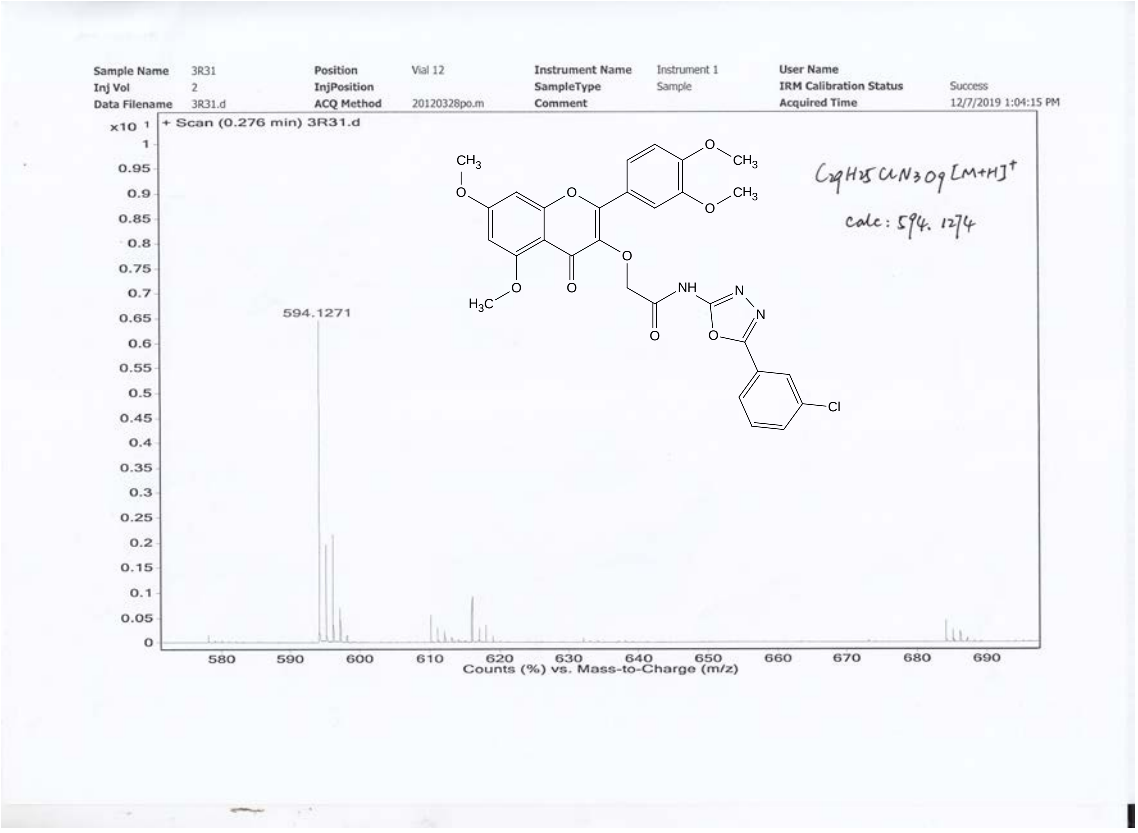

The spectrum of ^{1}H NMR, ^{13}C NMR and HRMS of compound B6

B7

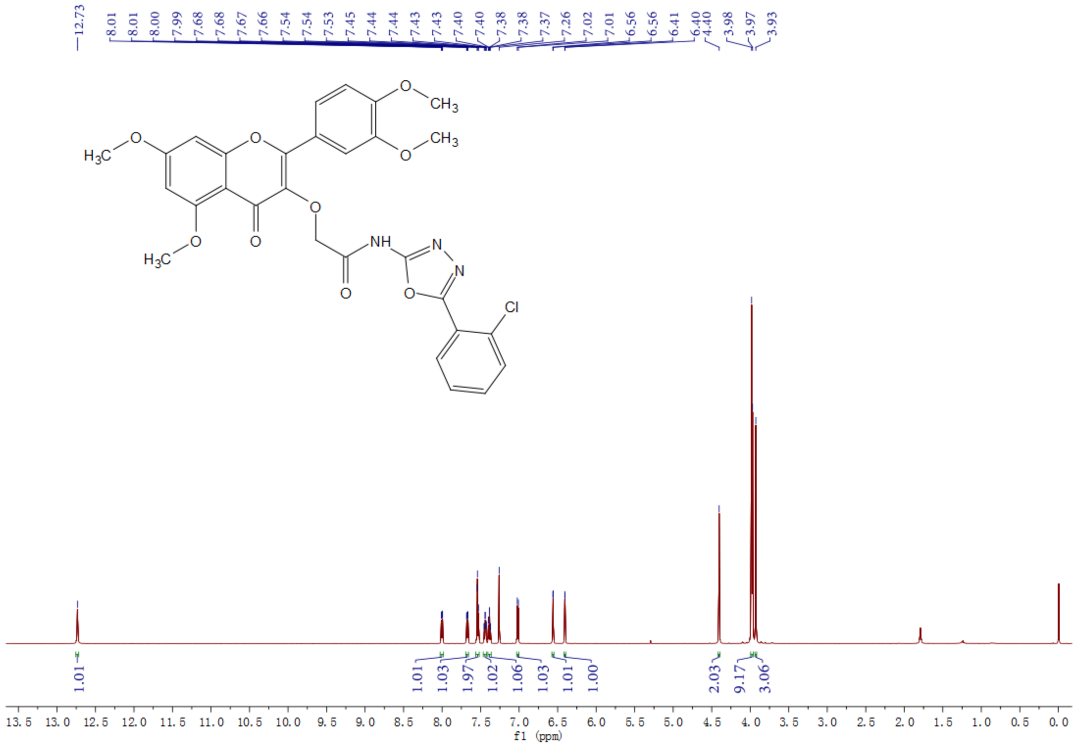

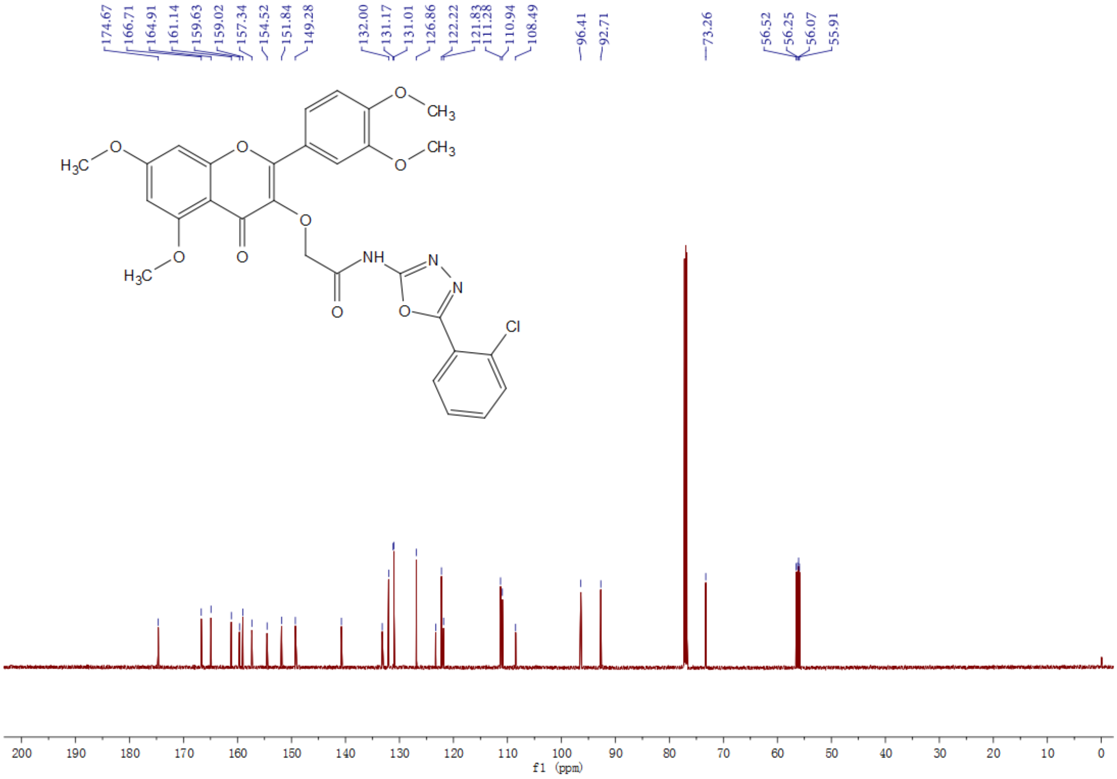

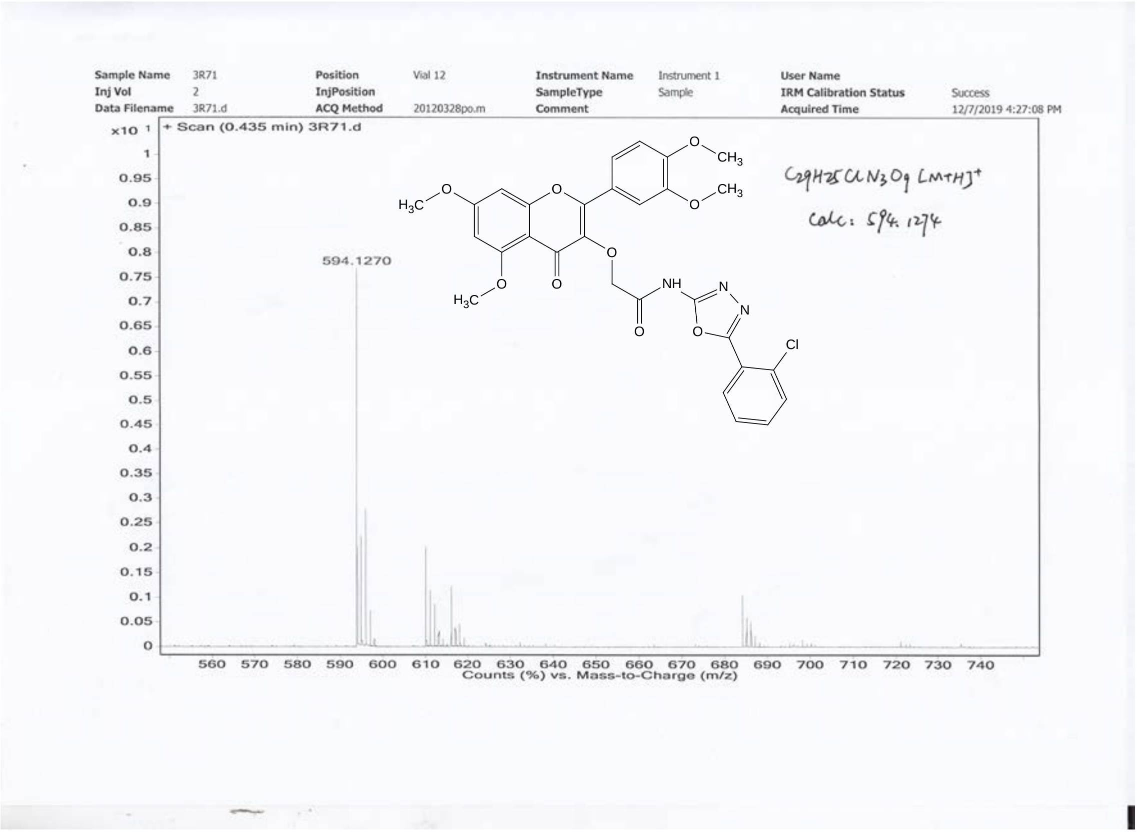

The spectrum of ^{1}H NMR, ^{13}C NMR and HRMS of compound B7

B8

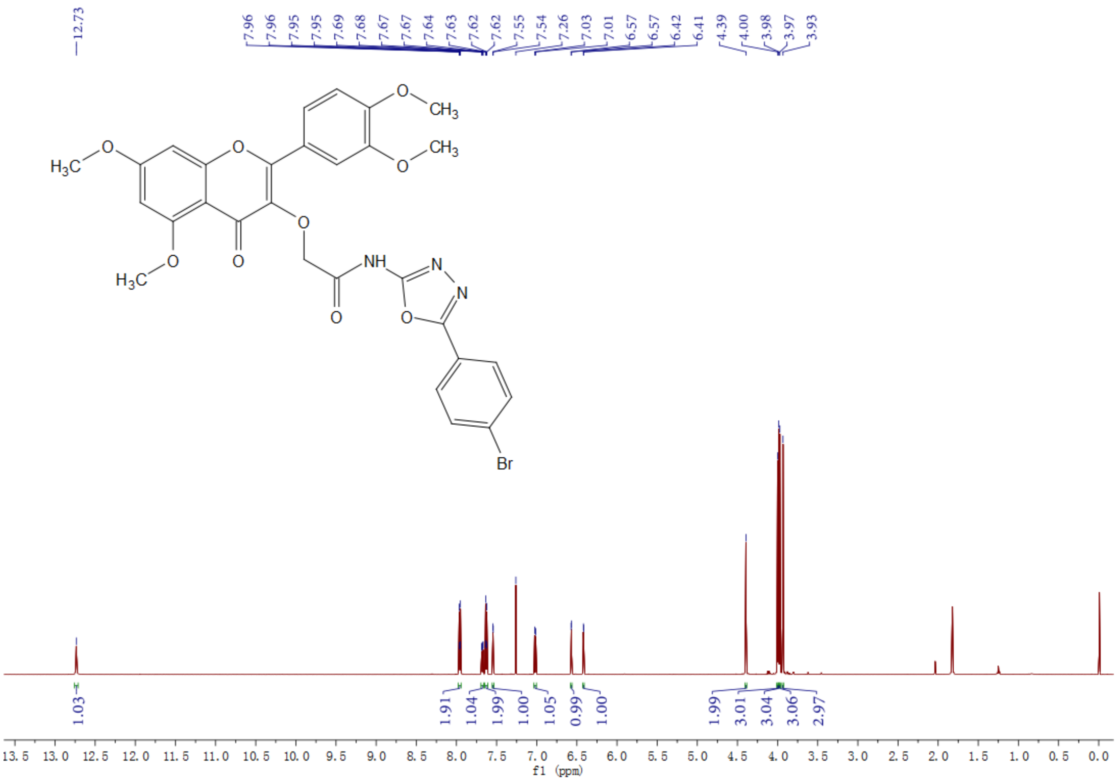
12.73
7.96
7.96
7.95
7.95
7.69
7.68
7.67
7.67
7.64
7.63
7.62
7.62
7.55
7.54
7.26
7.03
7.01
6.57
6.57
6.42
6.41
4.39
4.00
3.98
3.97
3.93
1.03
1.91
1.04
1.99
1.00
1.05
0.99
1.00
1.99
3.01
3.04
3.06
2.97
f1 (ppm)
Br
NH

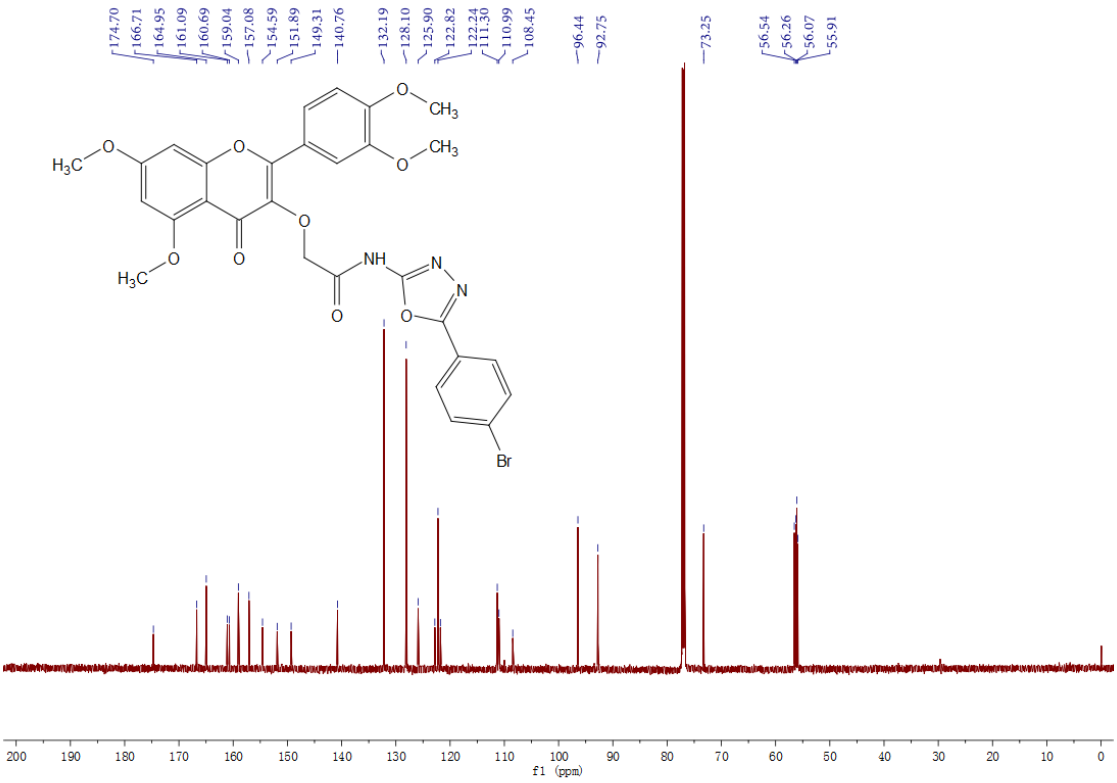
174.70
166.71
164.95
161.09
160.69
159.04
157.08
154.59
151.89
149.31
140.76
132.19
128.10
125.90
122.82
122.24
111.30
110.99
108.45
96.44
92.75
73.25
56.54
56.26
56.07
55.91
f1 (ppm)
Br
NH

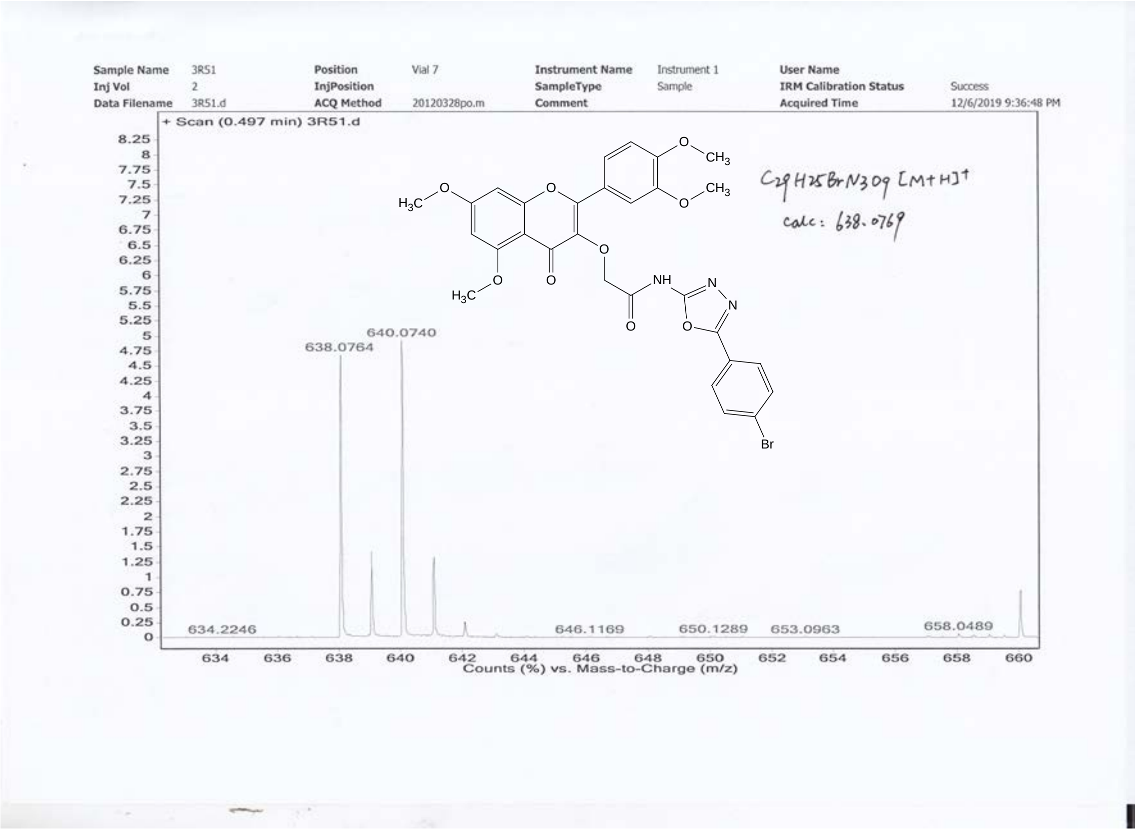

The spectrum of ^{1}H NMR, ^{13}C NMR and HRMS of compound B8

B9

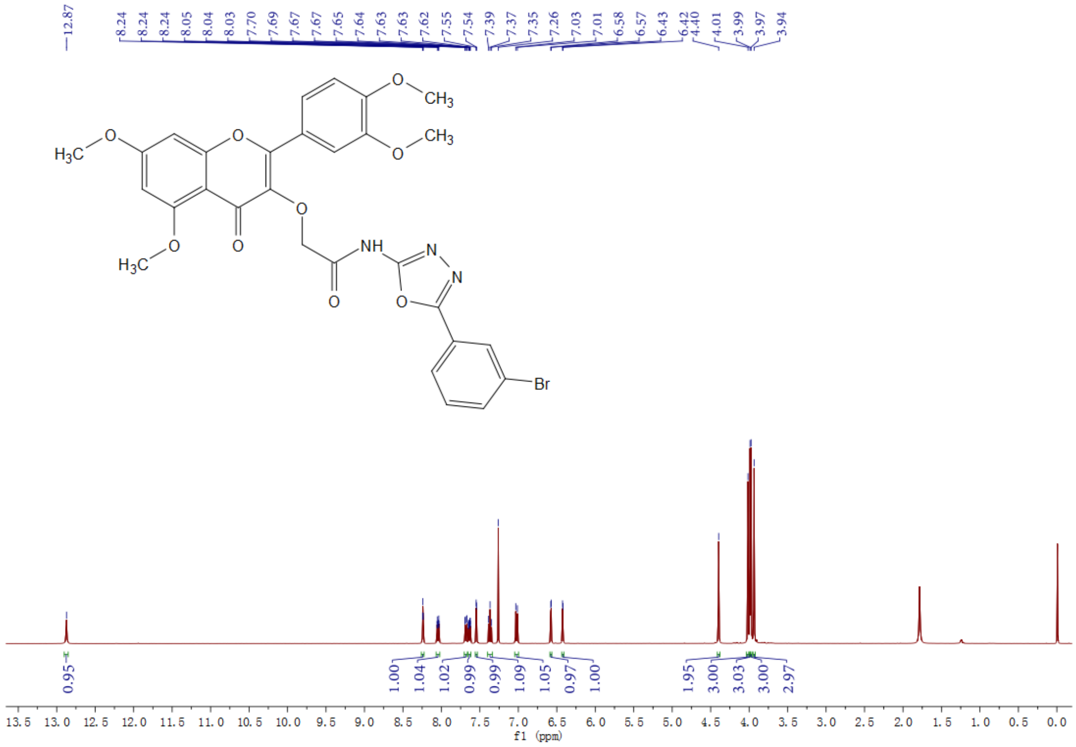

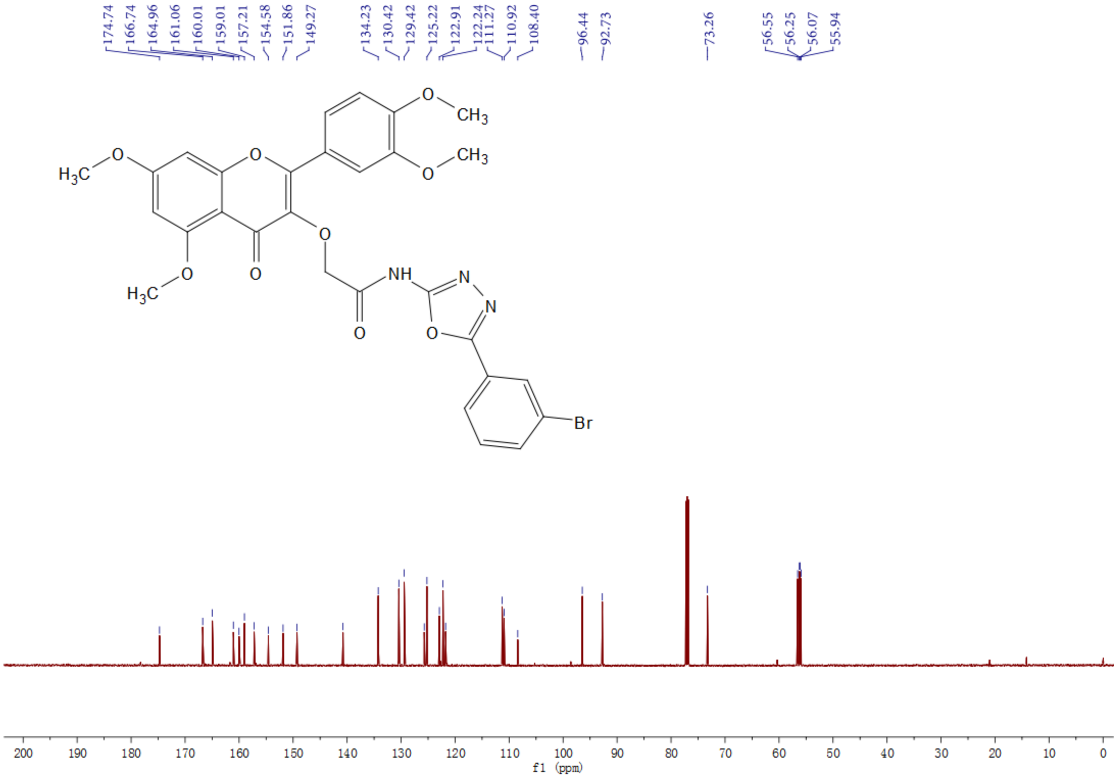

^{1}H NMR and ^{13}C NMR spectrum of compound B9

B10

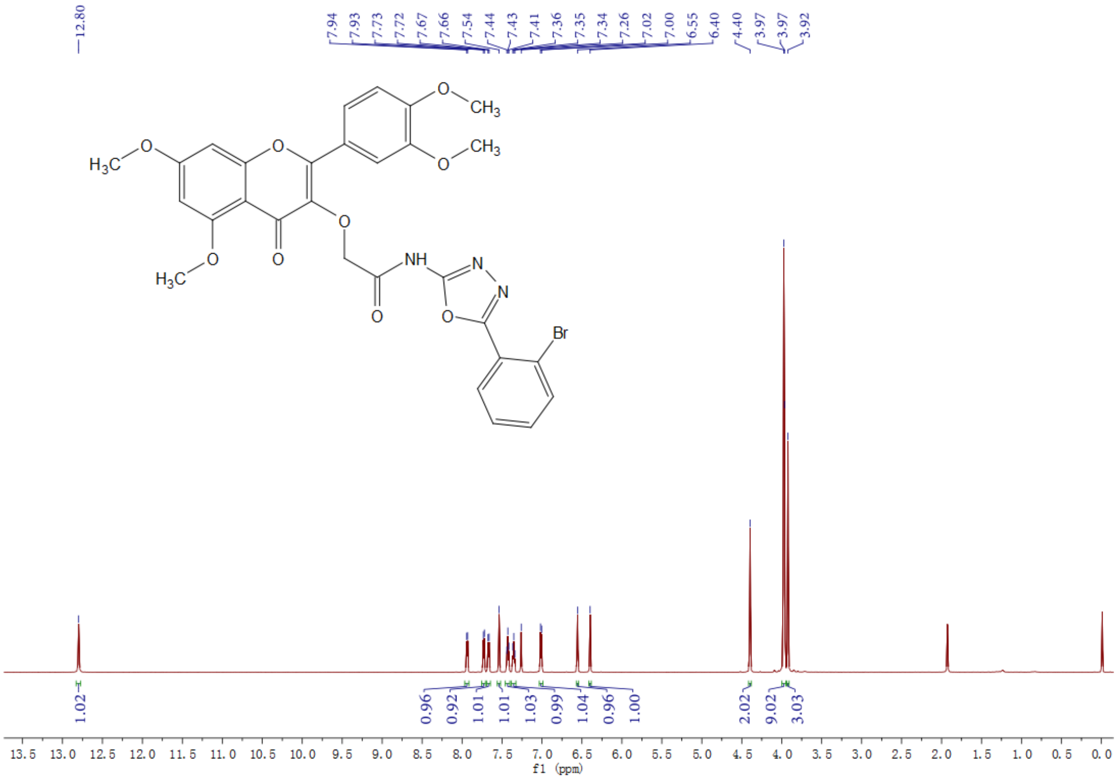

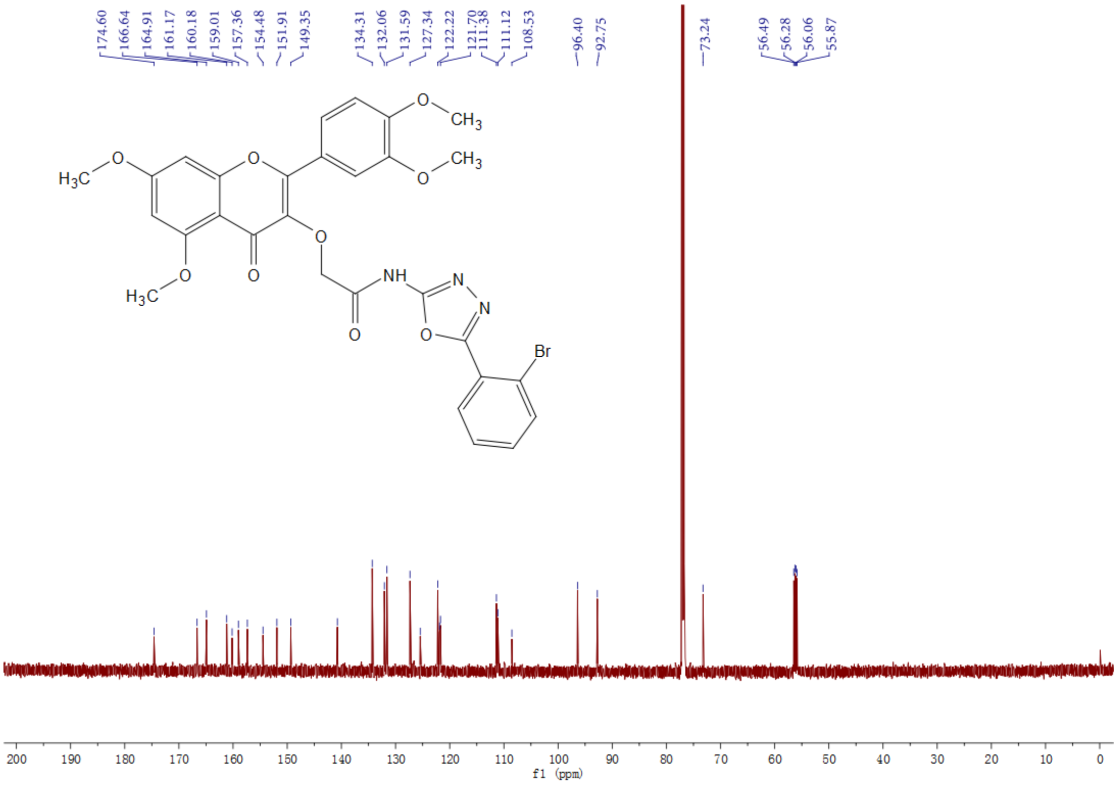

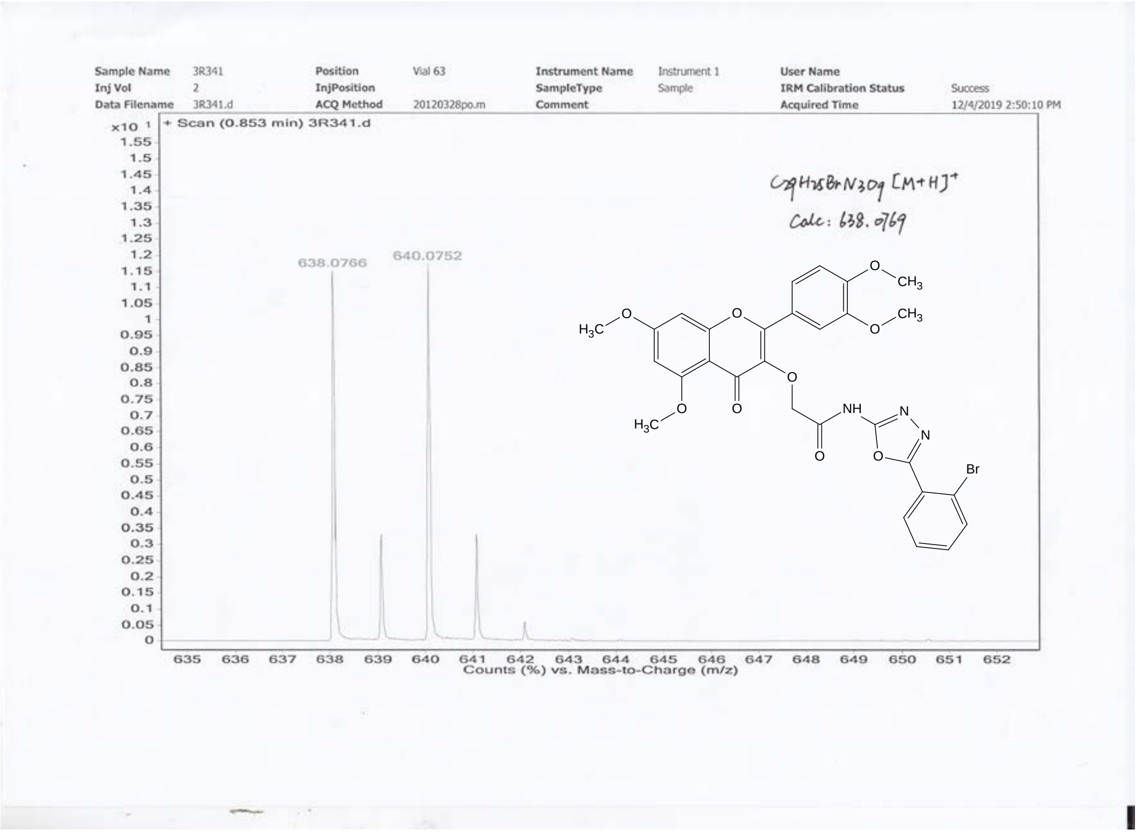

The spectrum of 1H NMR, 13C NMR and HRMS of compound B10

B11

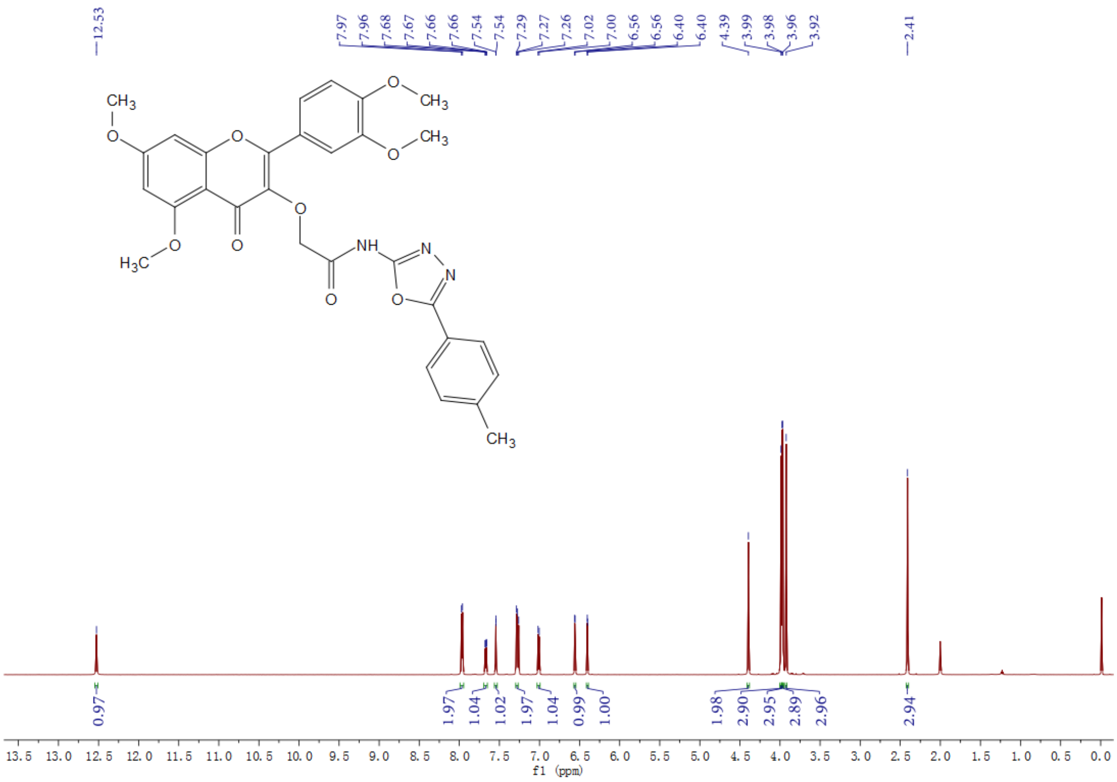

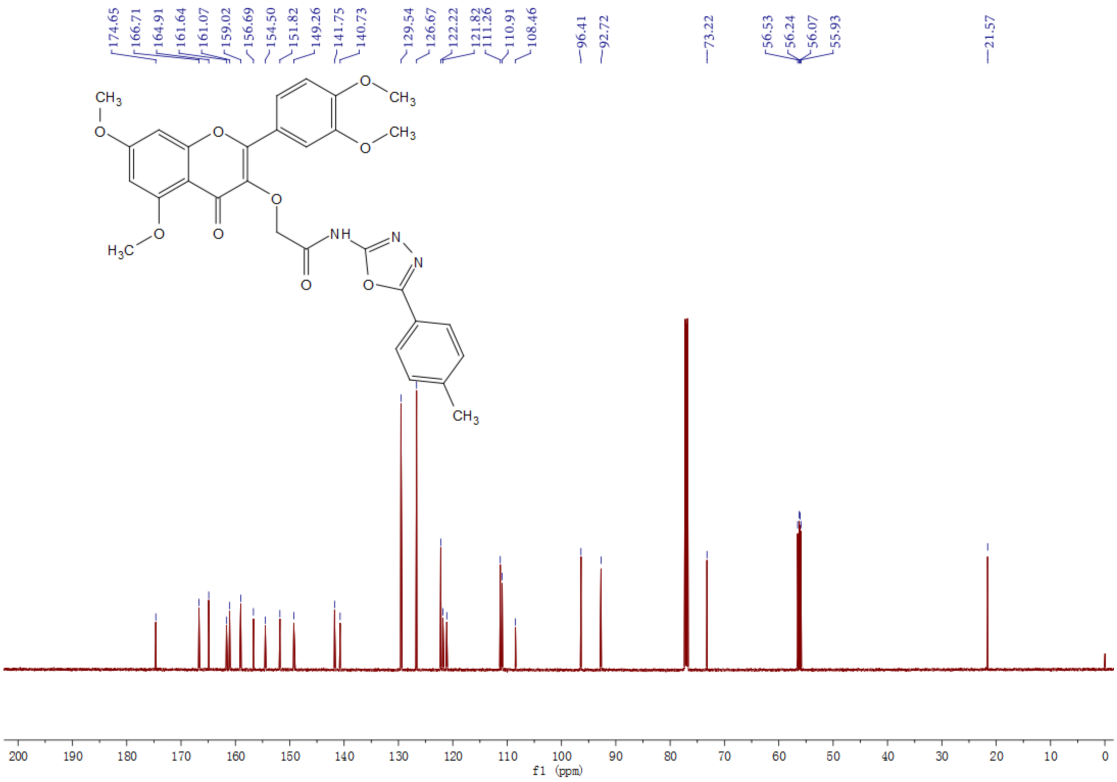

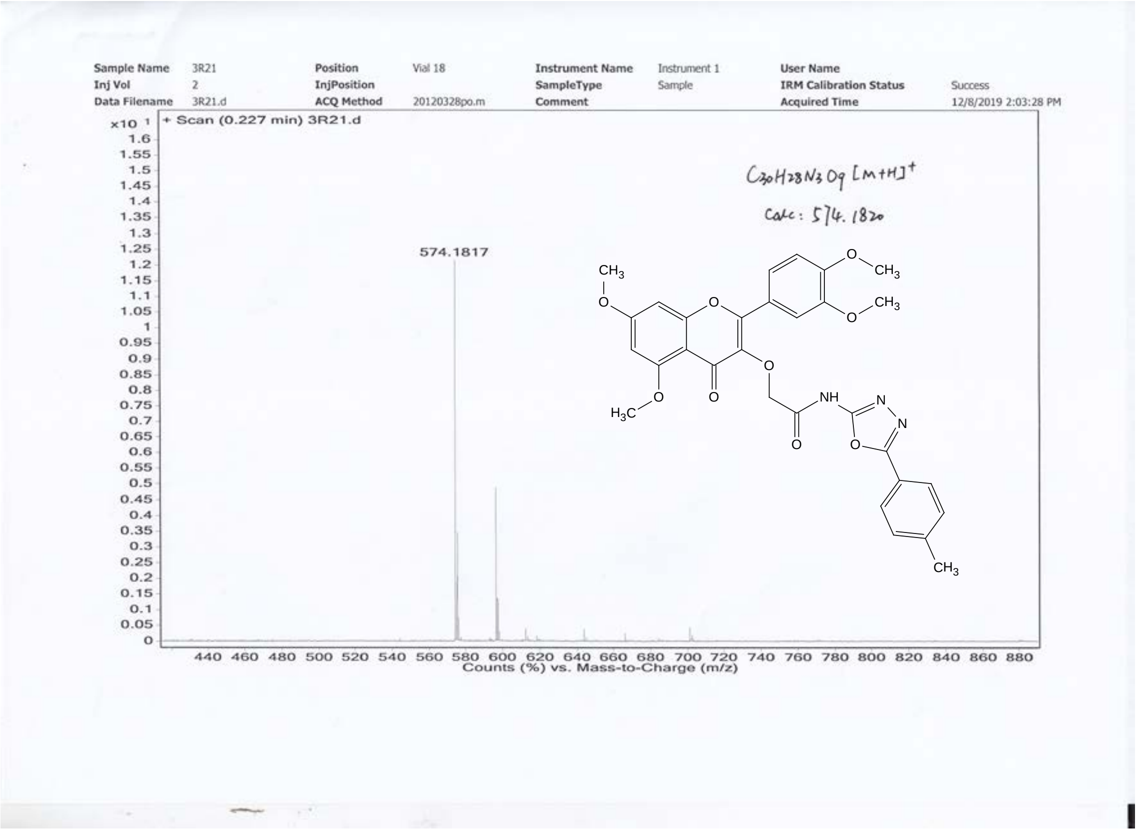

The spectrum of ^{1}H NMR, ^{13}C NMR and HRMS of compound B11

B12

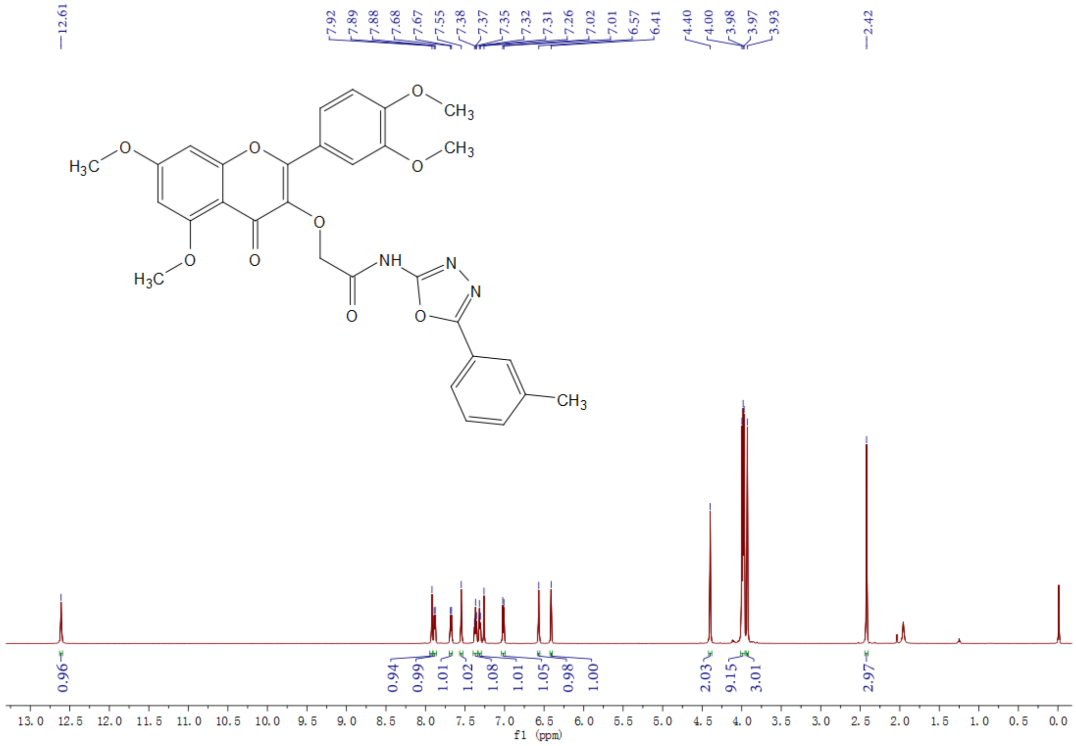

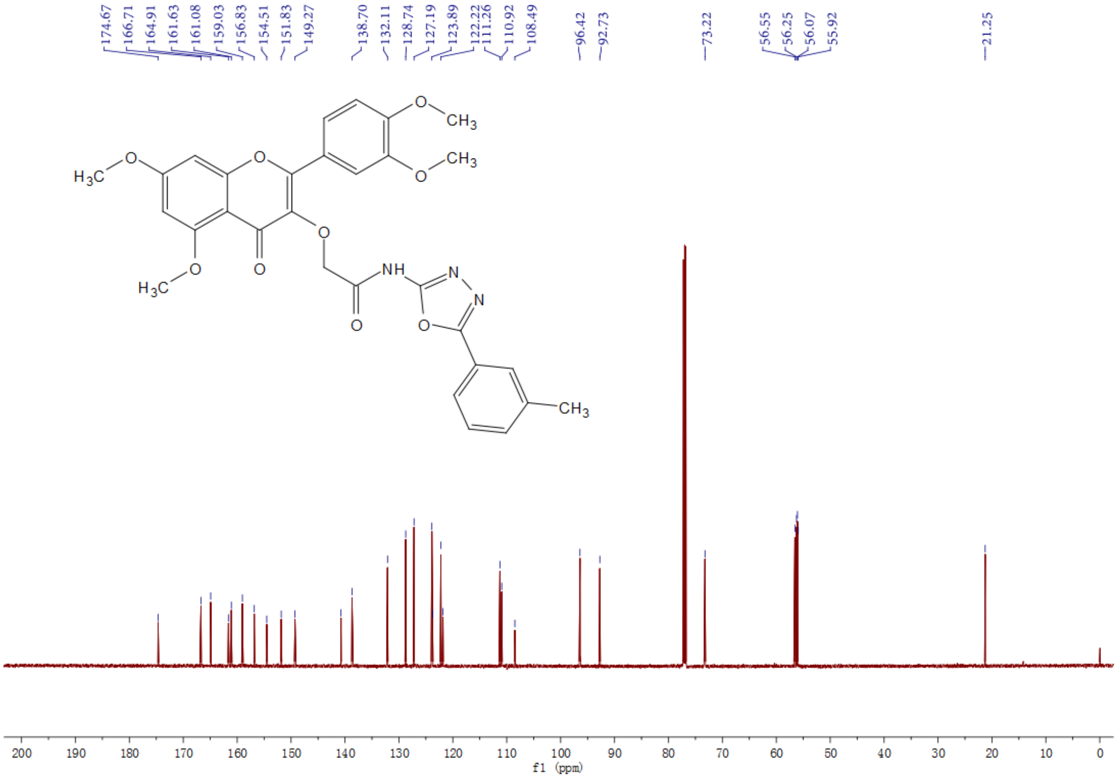

^{1}H NMR and ^{13}C NMR spectrum of compound B12

B13

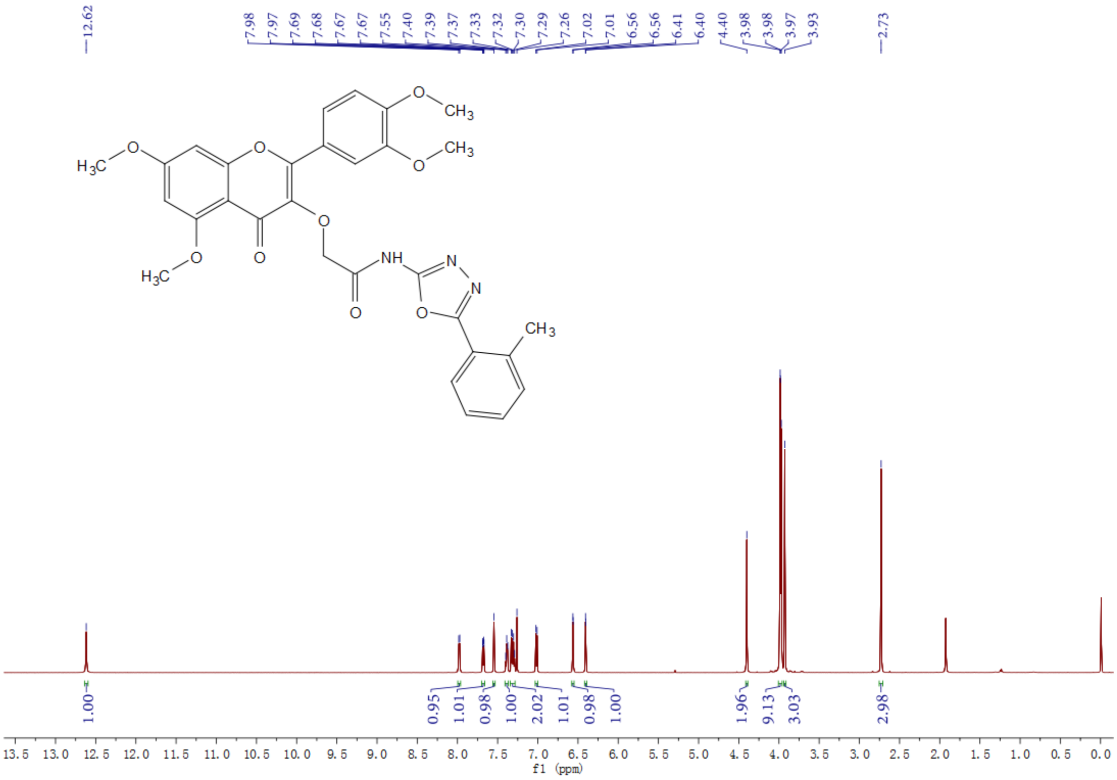

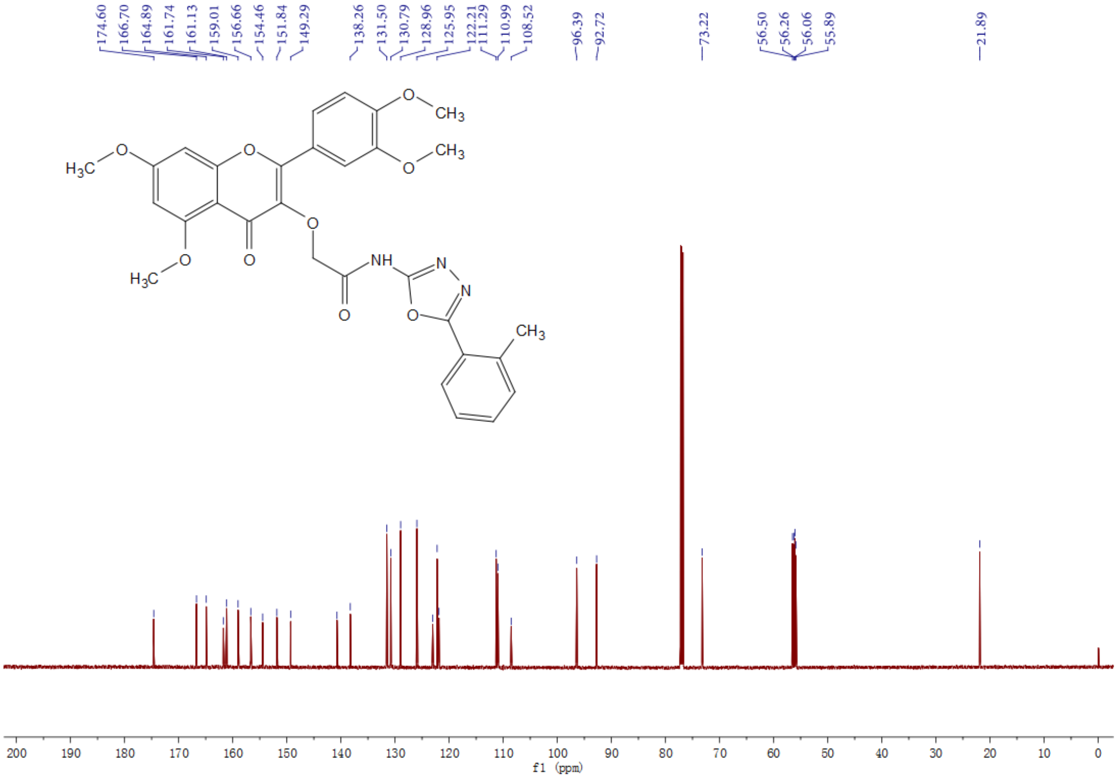

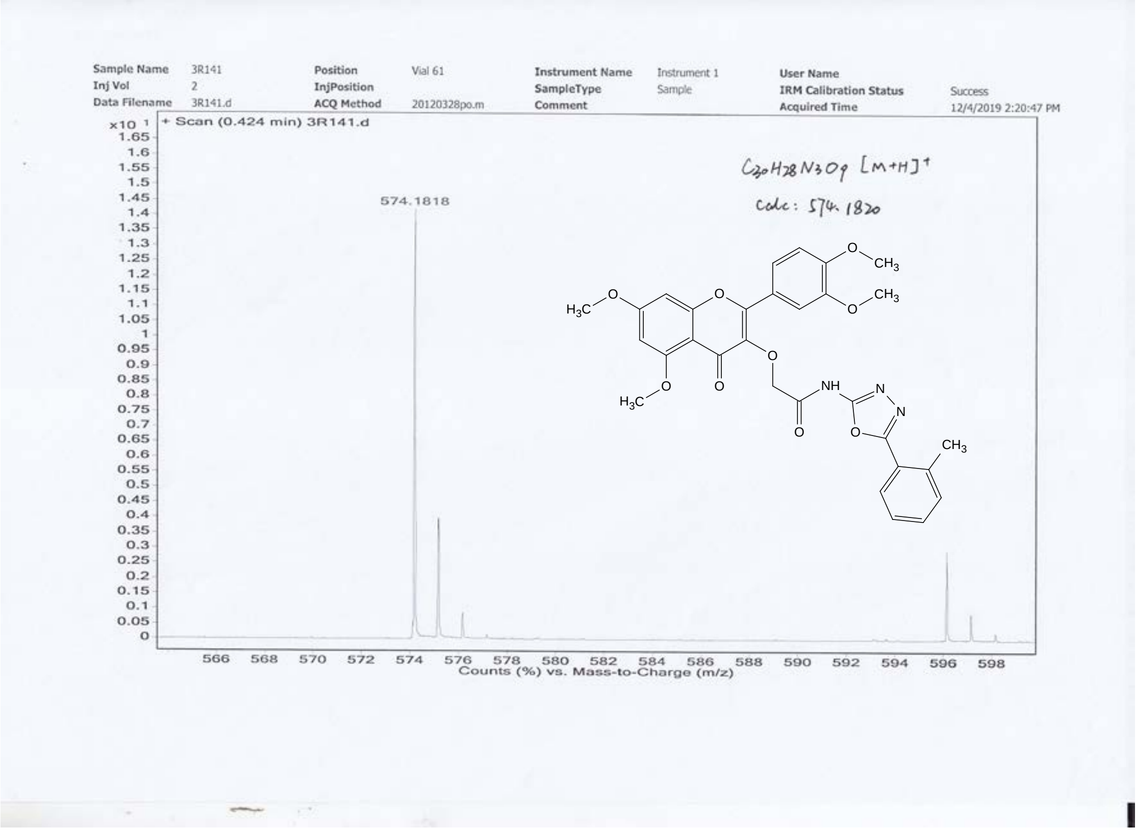

The spectrum of ^{1}H NMR, ^{13}C NMR and HRMS of compound B13

B14

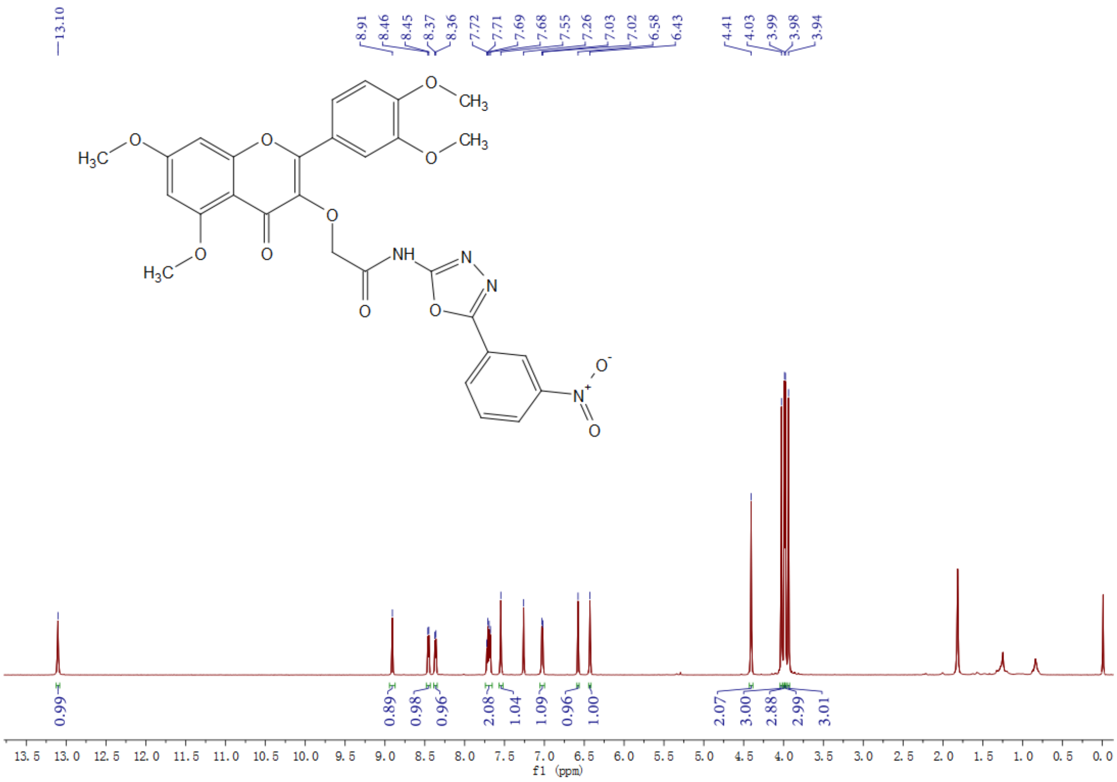

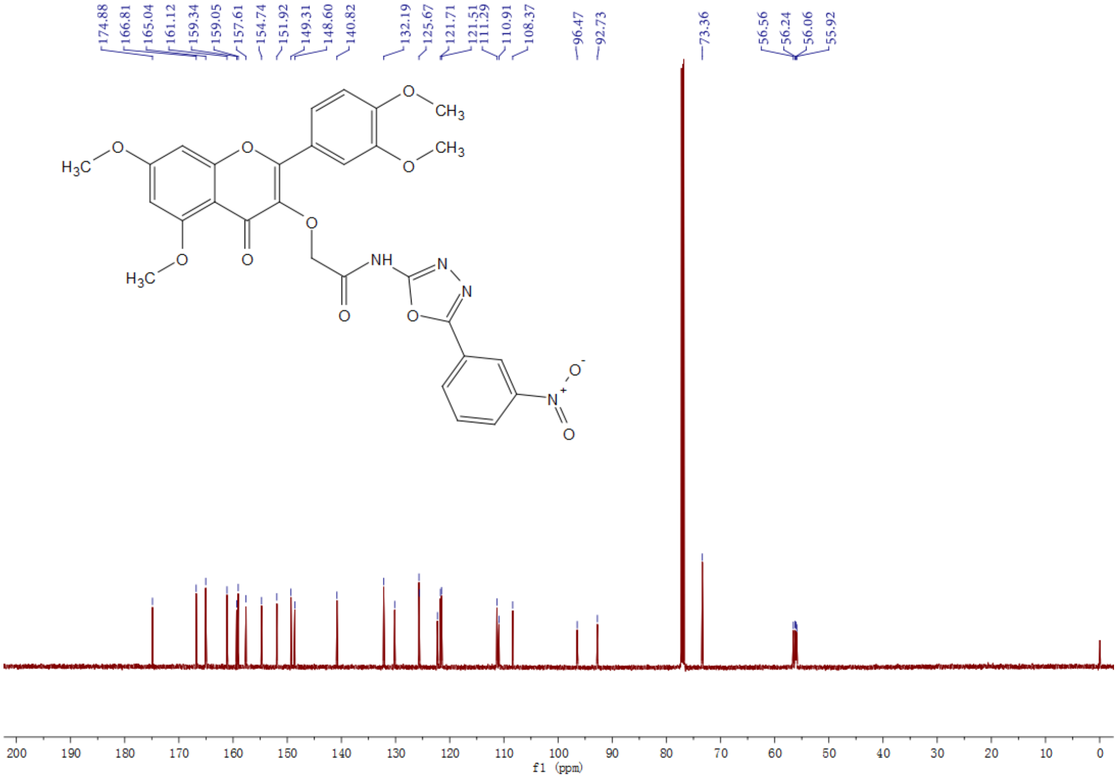

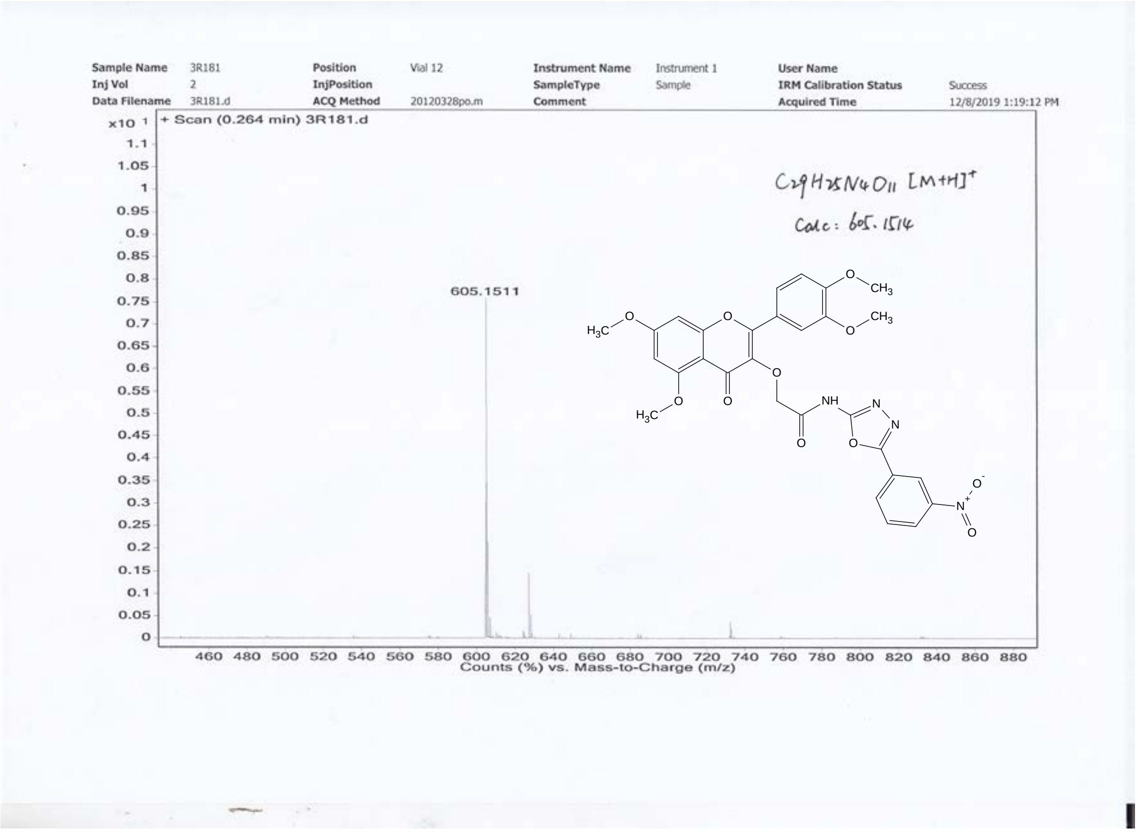

The spectrum of ^{1}H NMR, ^{13}C NMR and HRMS of compound B14

B15

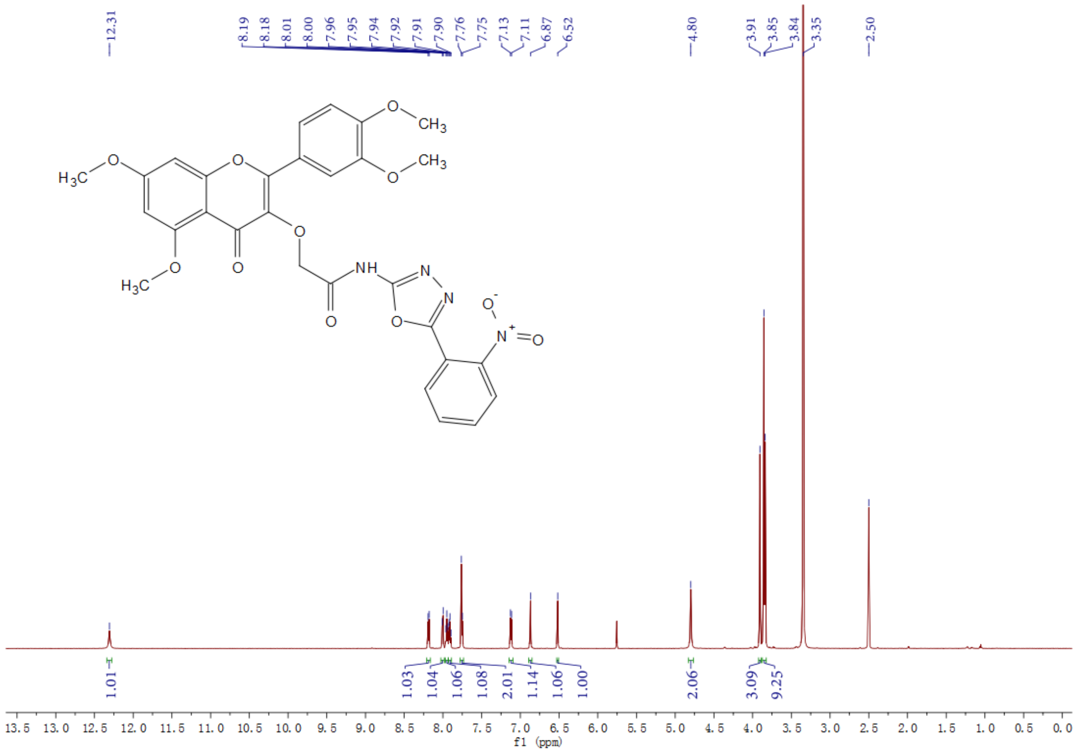

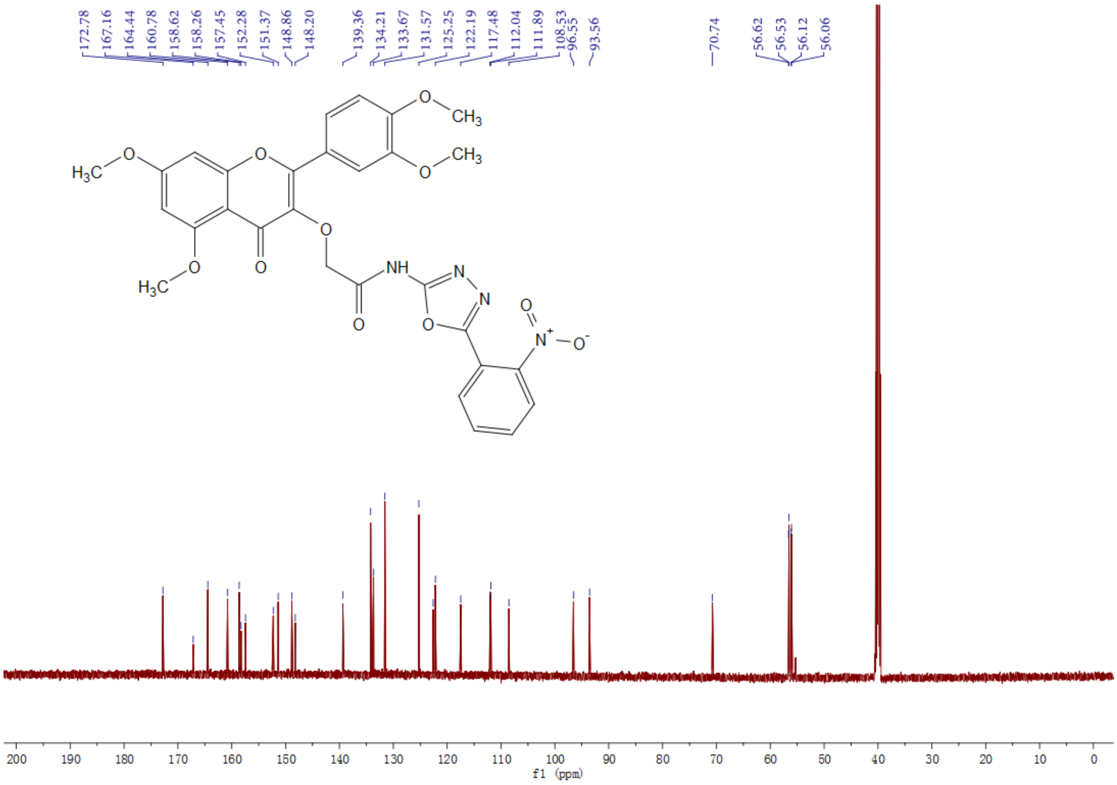

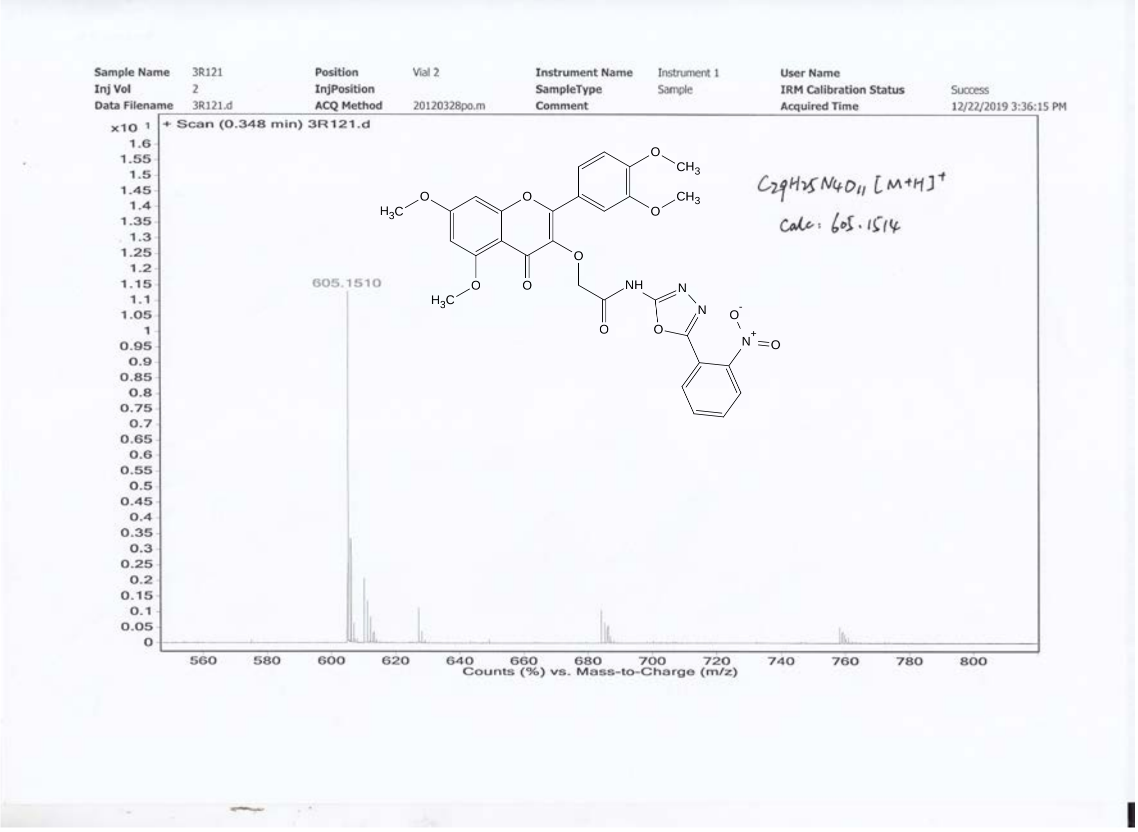

The spectrum of ^{1}H NMR, ^{13}C NMR and HRMS of compound B15

B16

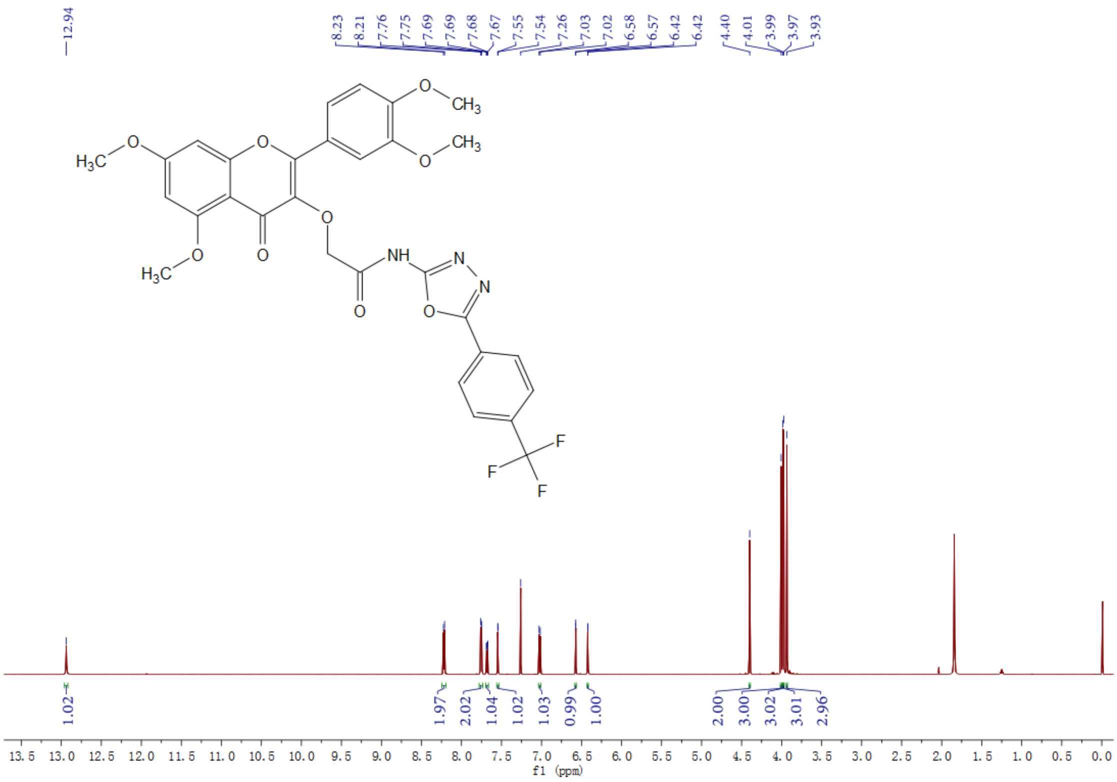

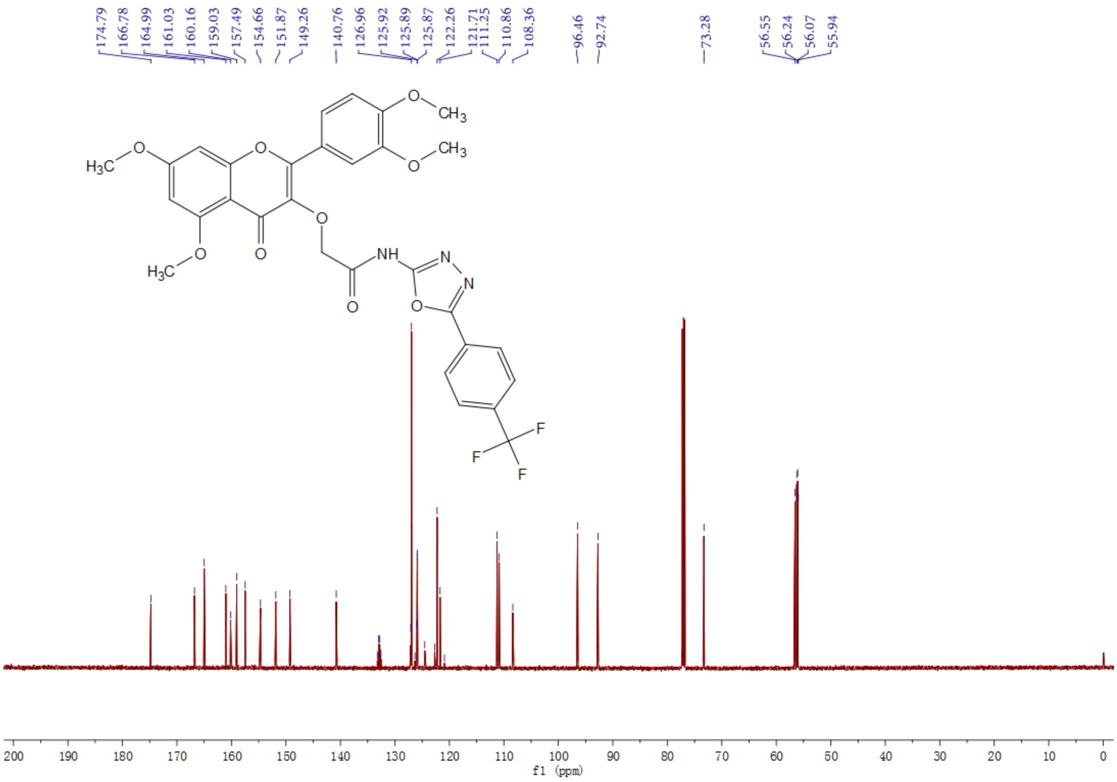

The spectrum of ^{1}H NMR and ^{13}C NMR compound B16

B17

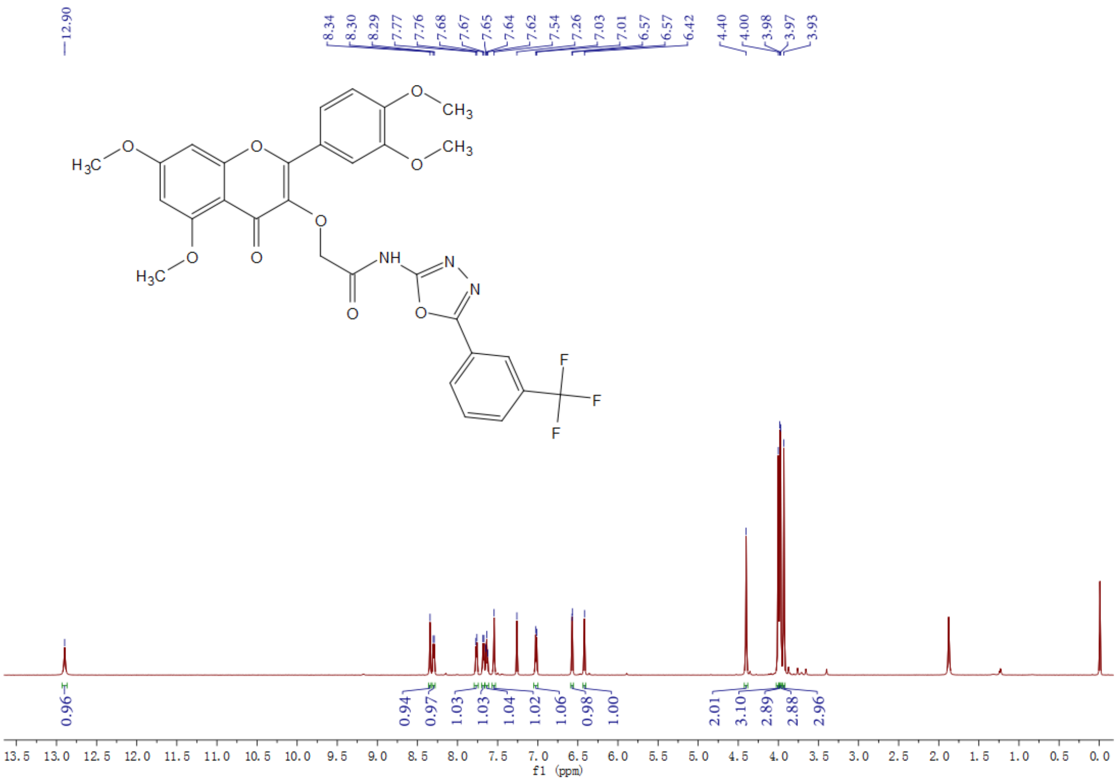

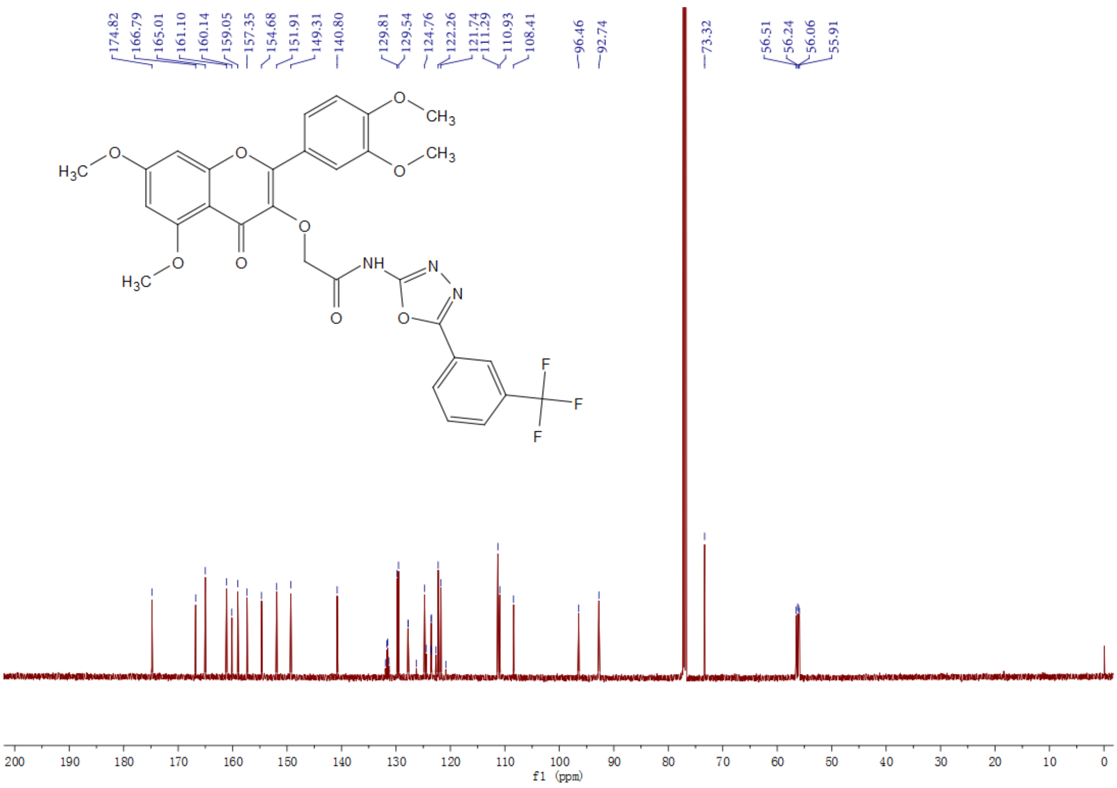

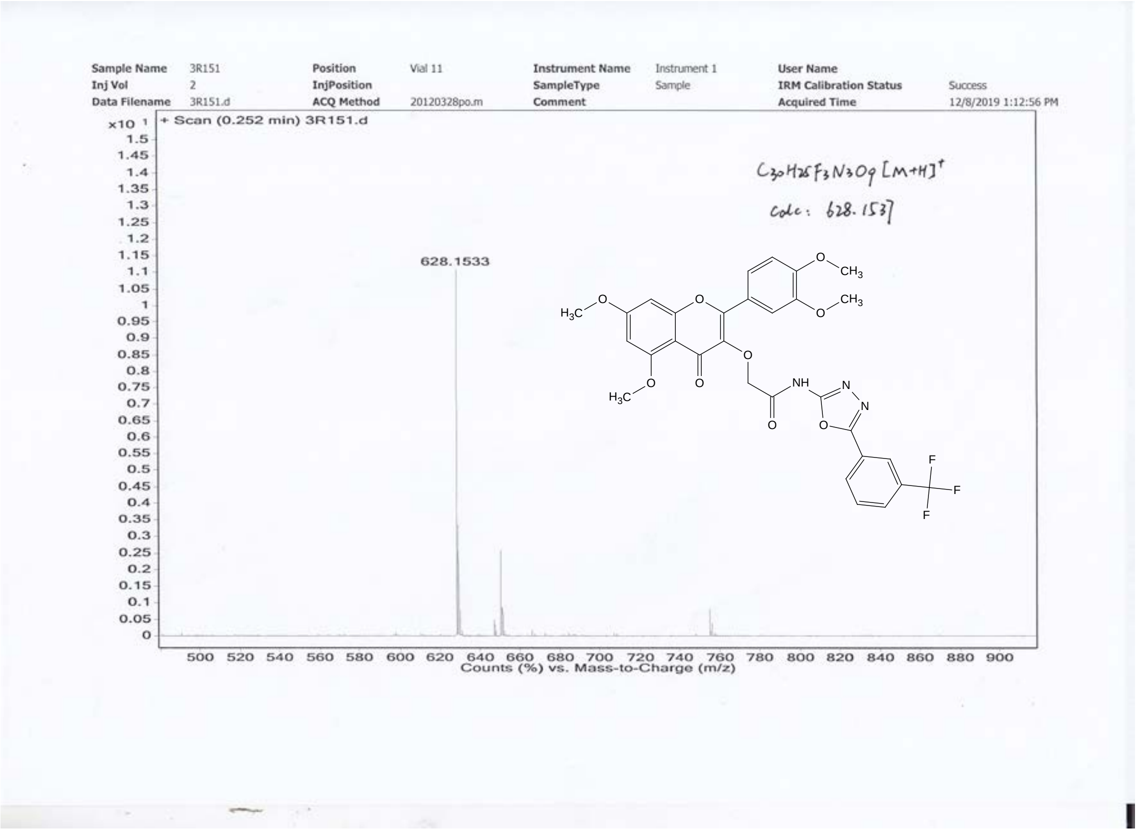

The spectrum of ^{1}H NMR, ^{13}C NMR and HRMS of compound B17

B18

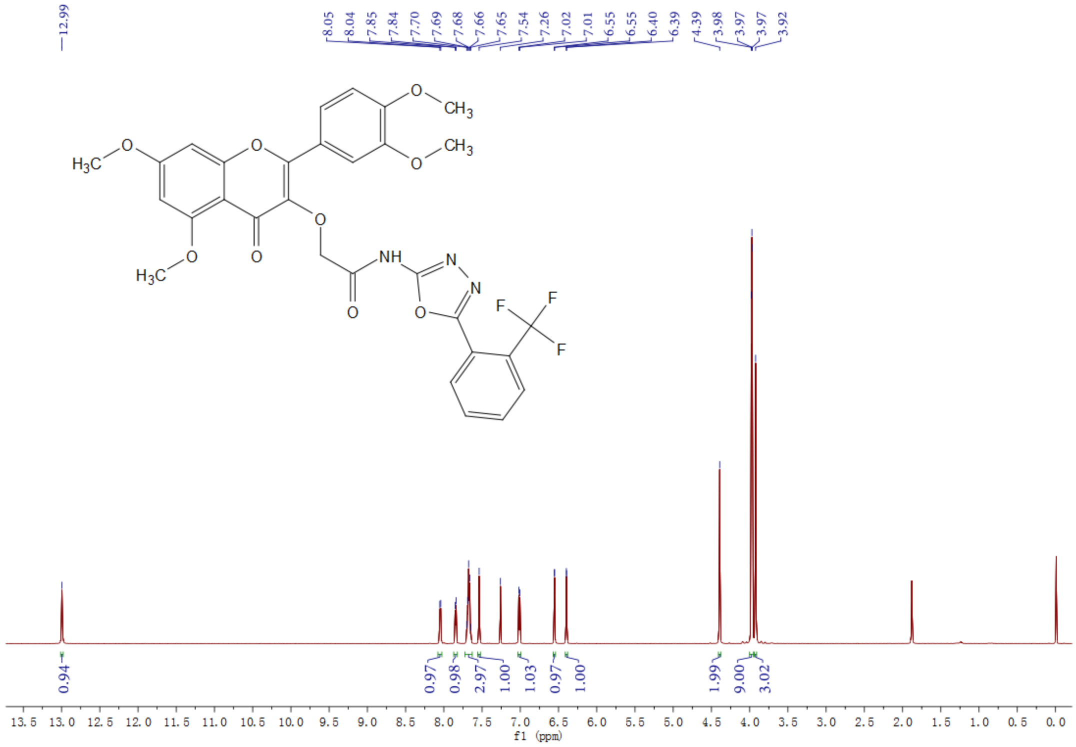

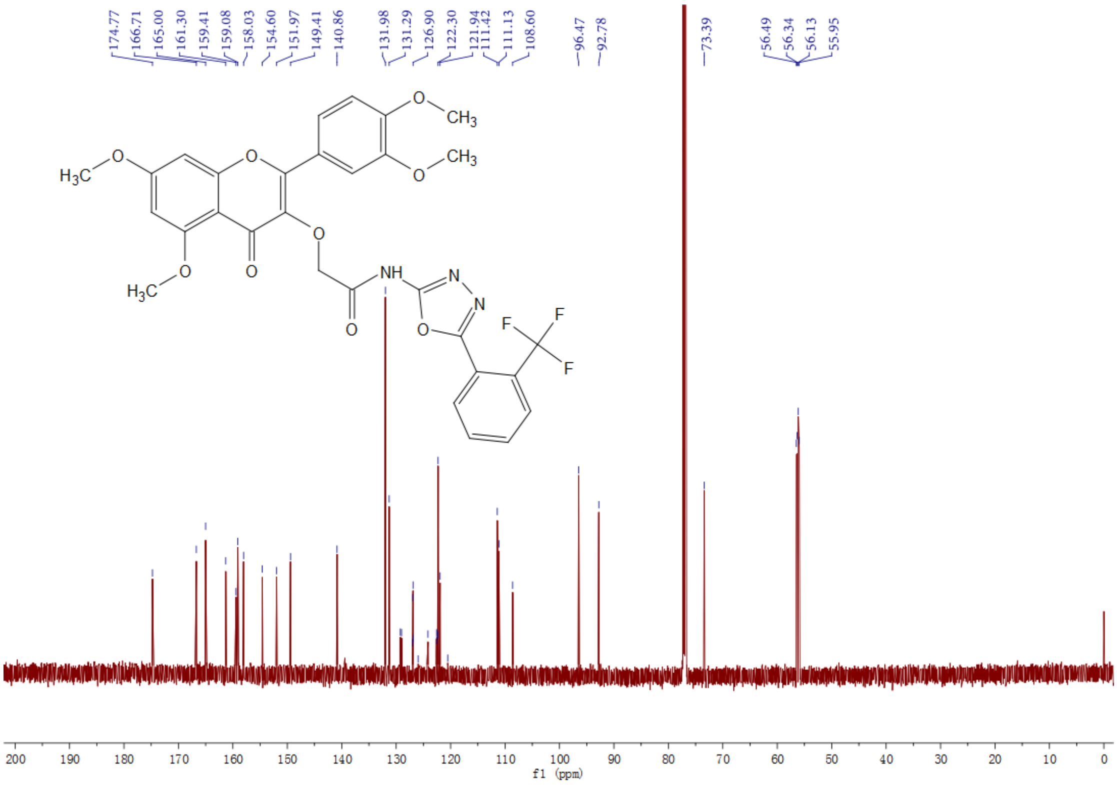

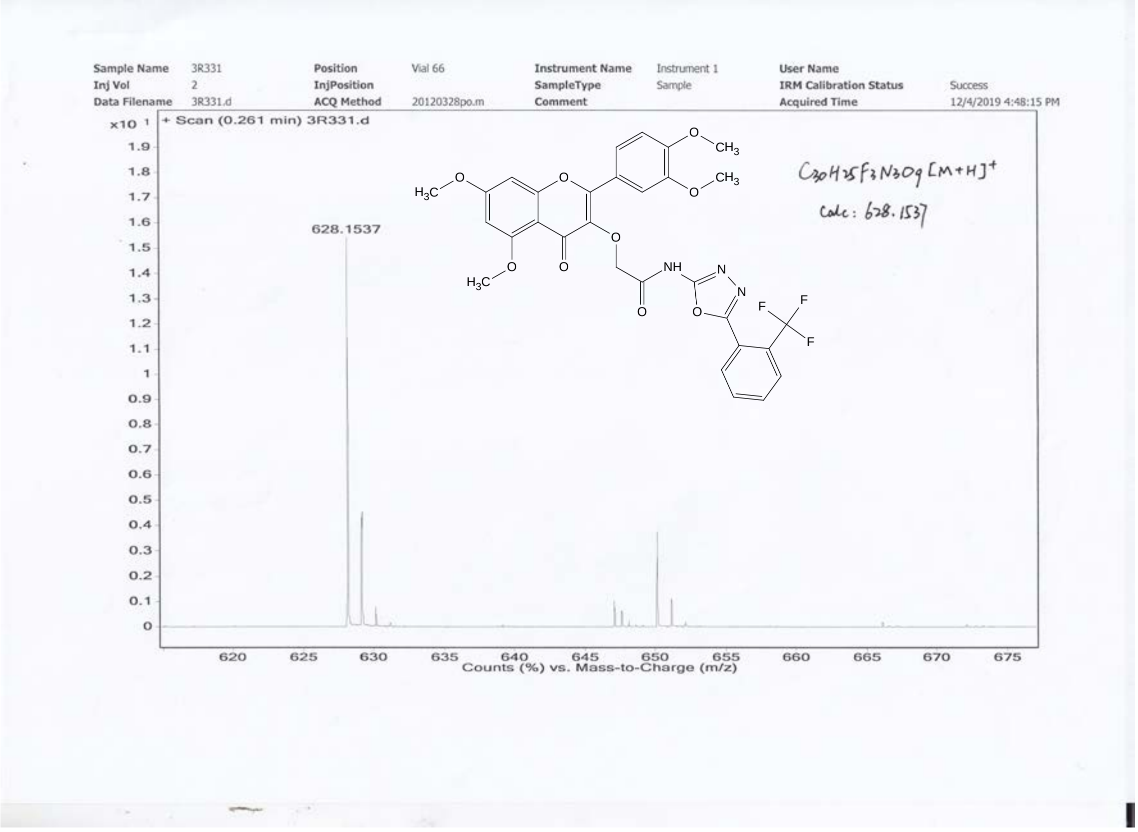

The spectrum of ^{1}H NMR, ^{13}C NMR and HRMS of compound B18

B19

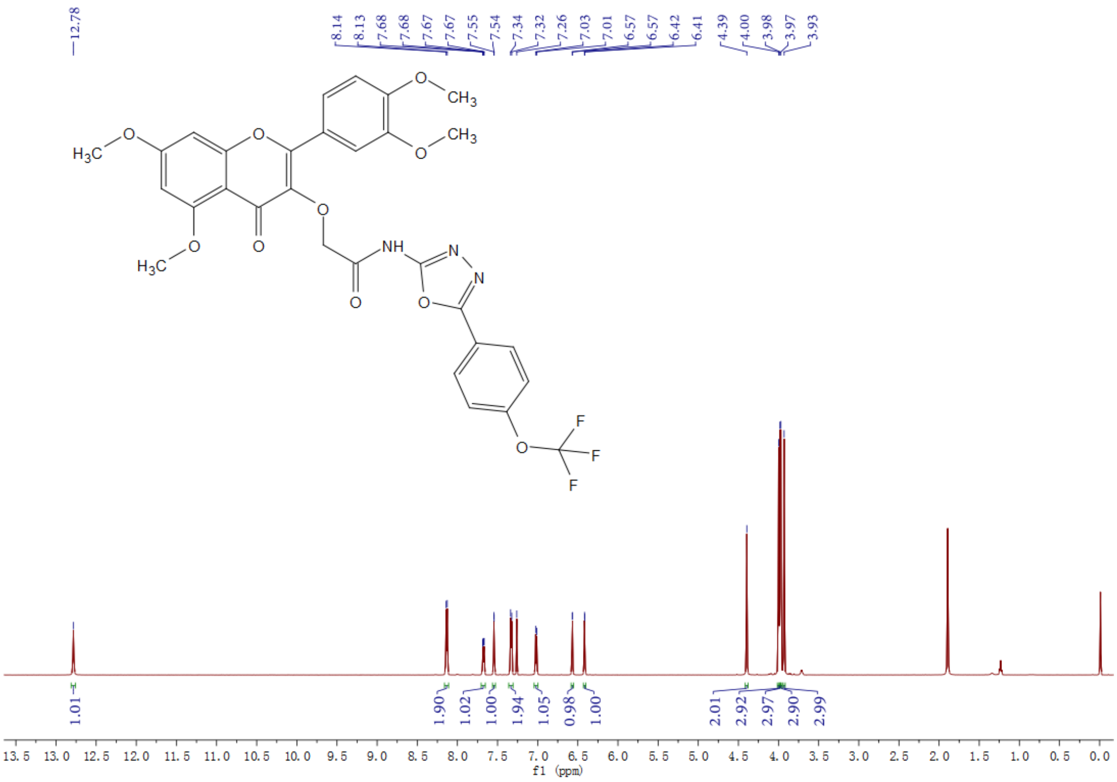

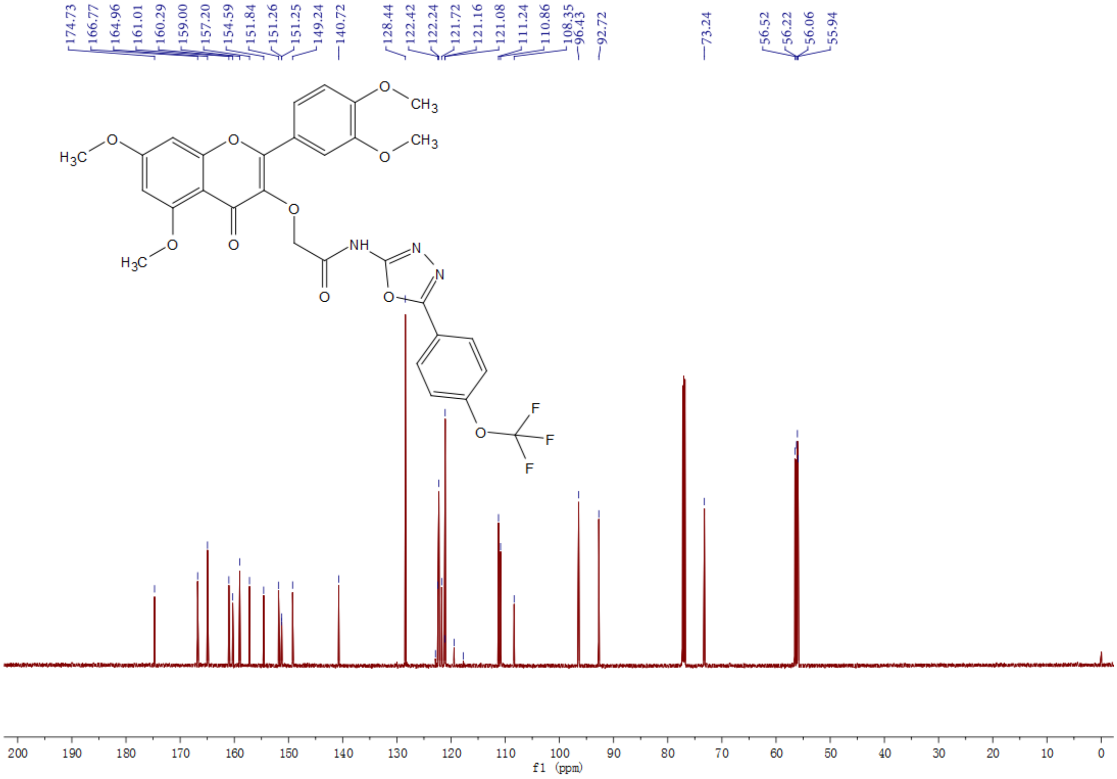

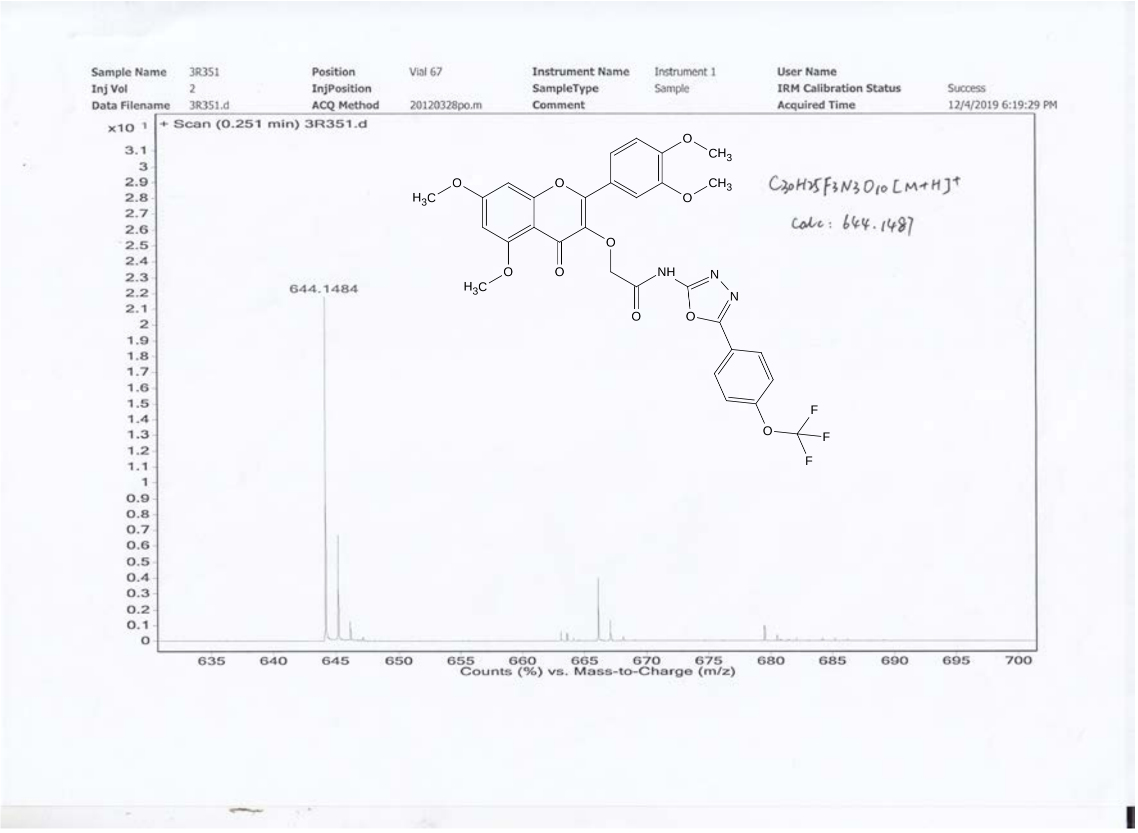

The spectrum of ^{1}H NMR, ^{13}C NMR and HRMS of compound B19

B20

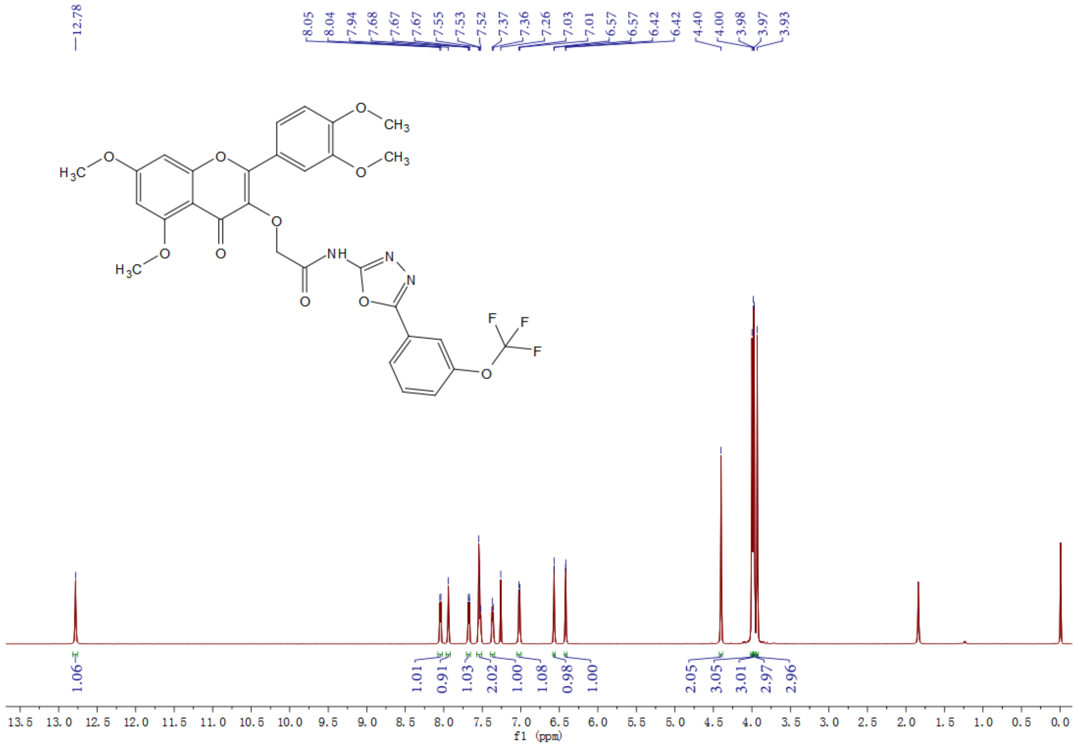

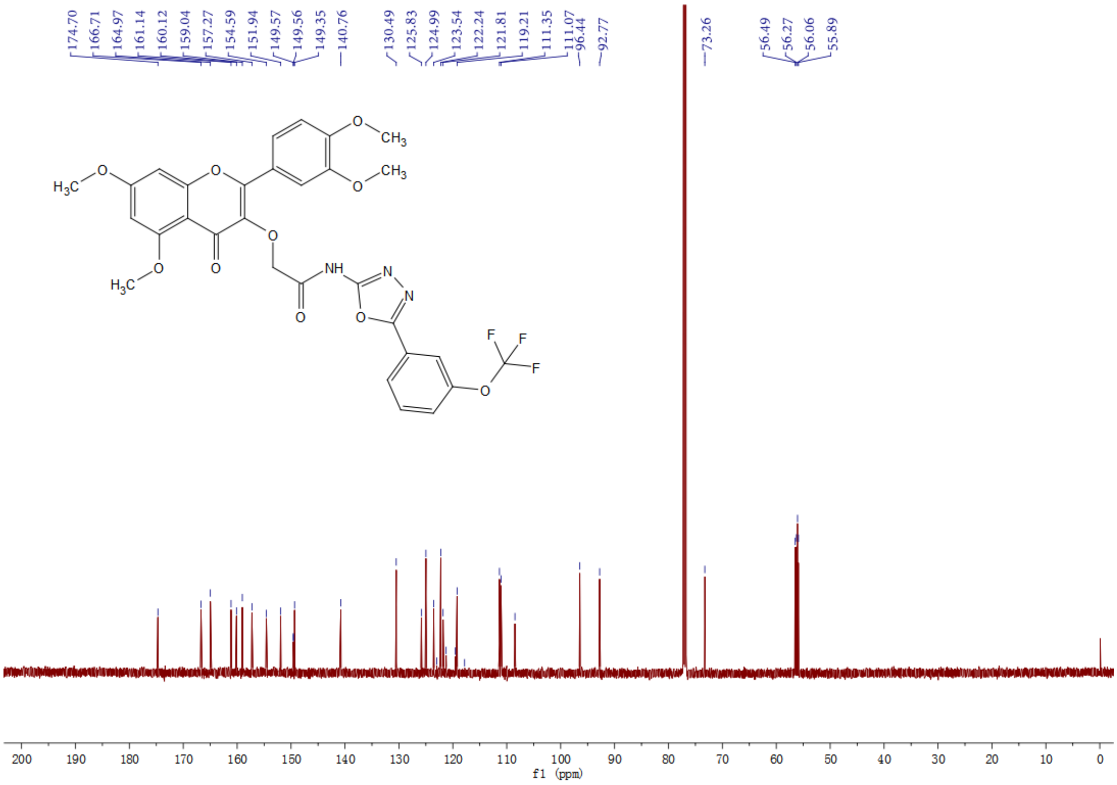

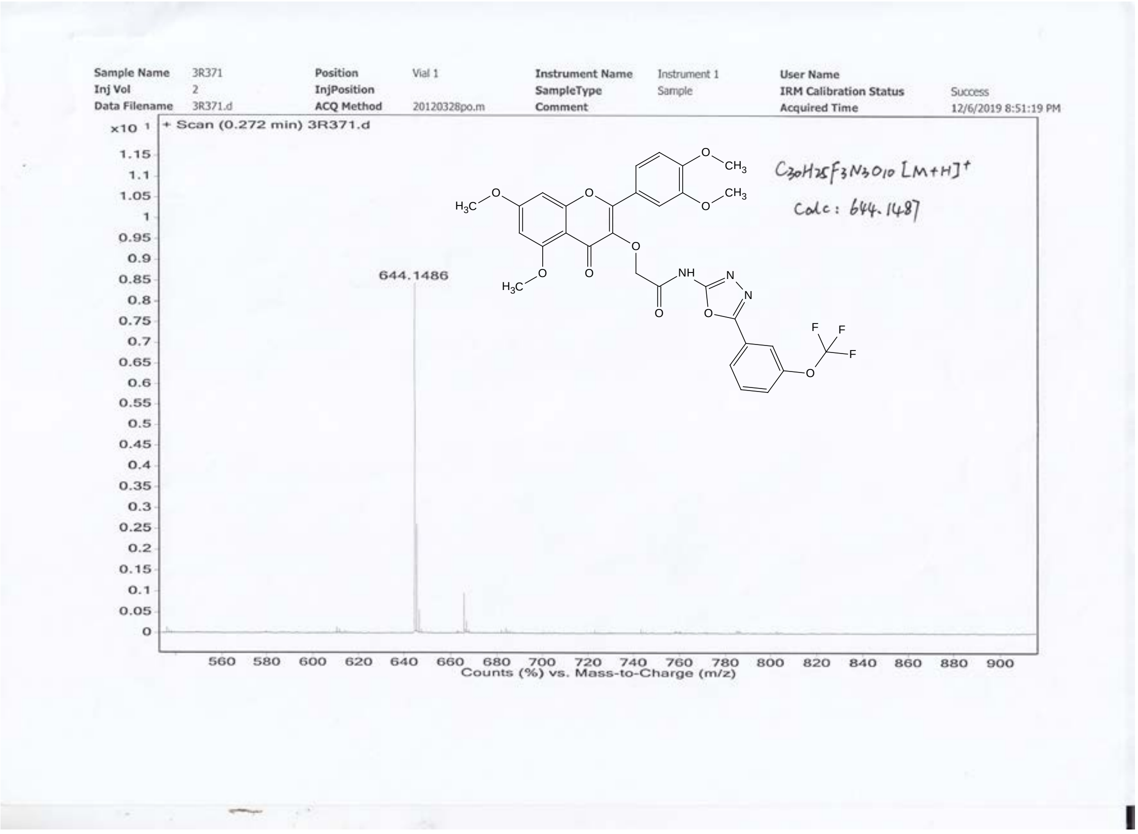

The spectrum of ^{1}H NMR, ^{13}C NMR and HRMS of compound B20

B21

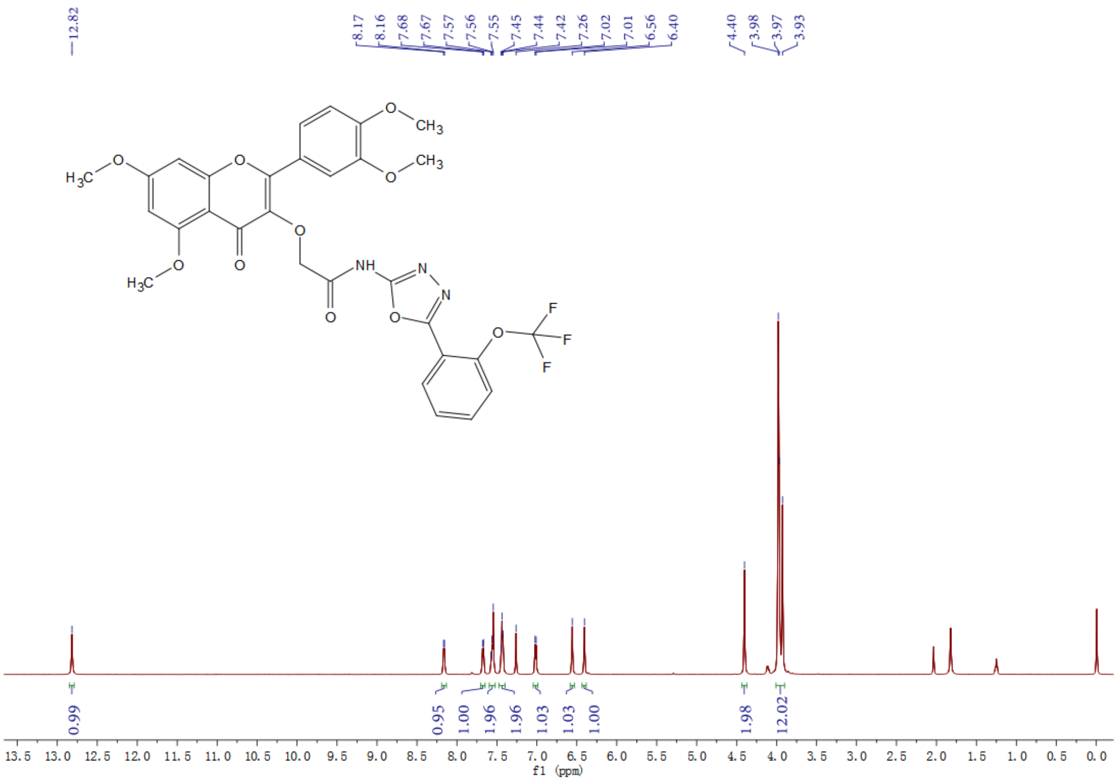
12.82
8.17
8.16
7.68
7.67
7.57
7.56
7.55
7.45
7.44
7.42
7.26
7.02
7.01
6.56
6.40
4.40
3.98
3.97
3.93
0.99
0.95
1.00
1.96
1.96
1.03
1.03
1.00
1.98
12.02
f1 (ppm)

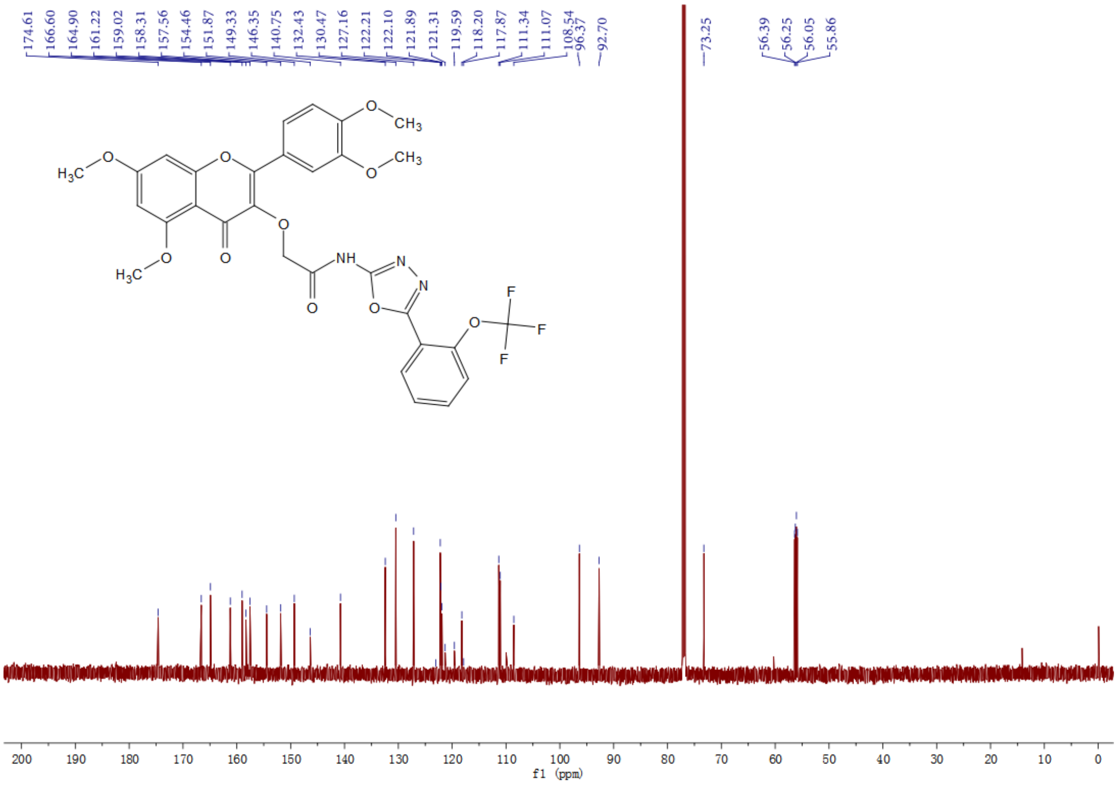
174.61
166.60
164.90
161.22
159.02
158.31
157.56
154.46
151.87
149.33
146.35
140.75
132.43
130.47
127.16
122.21
122.10
121.89
121.31
119.59
118.20
117.87
111.34
111.07
108.54
96.37
92.70
73.25
56.39
56.25
56.05
55.86
f1 (ppm)

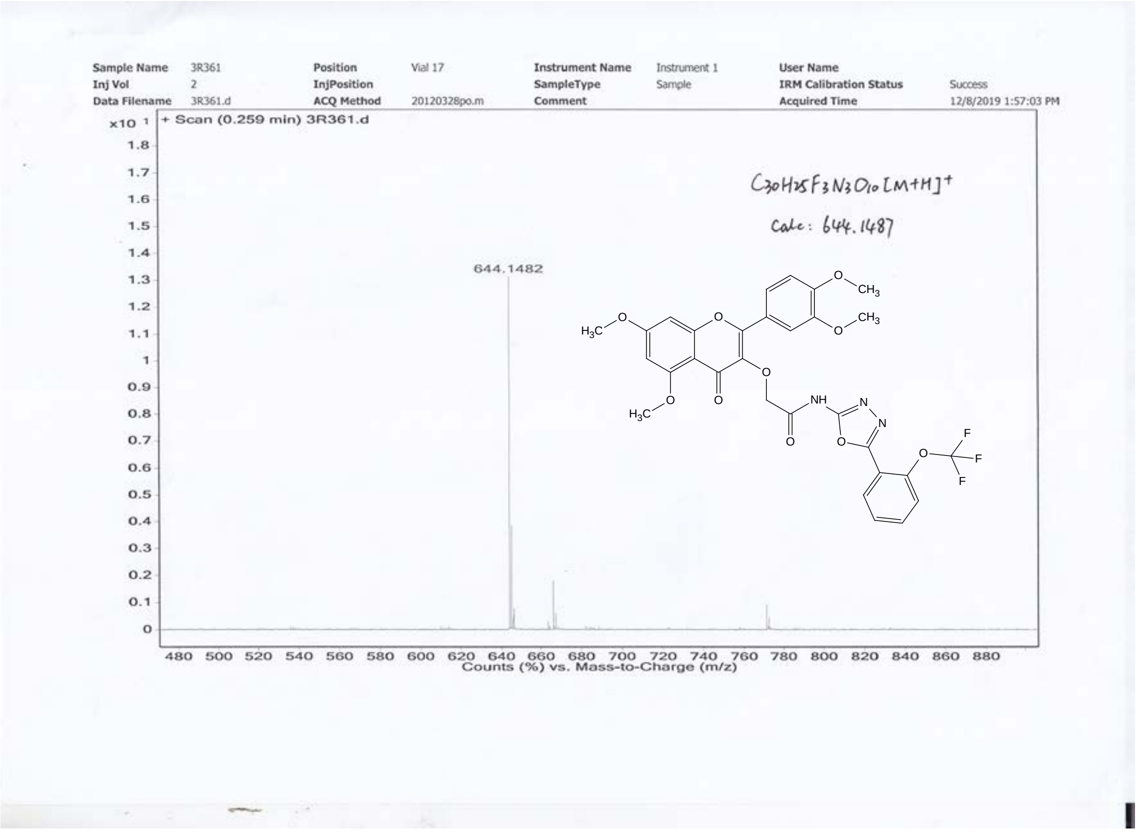

The spectrum of ^{1}H NMR, ^{13}C NMR and HRMS of compound B21

B22

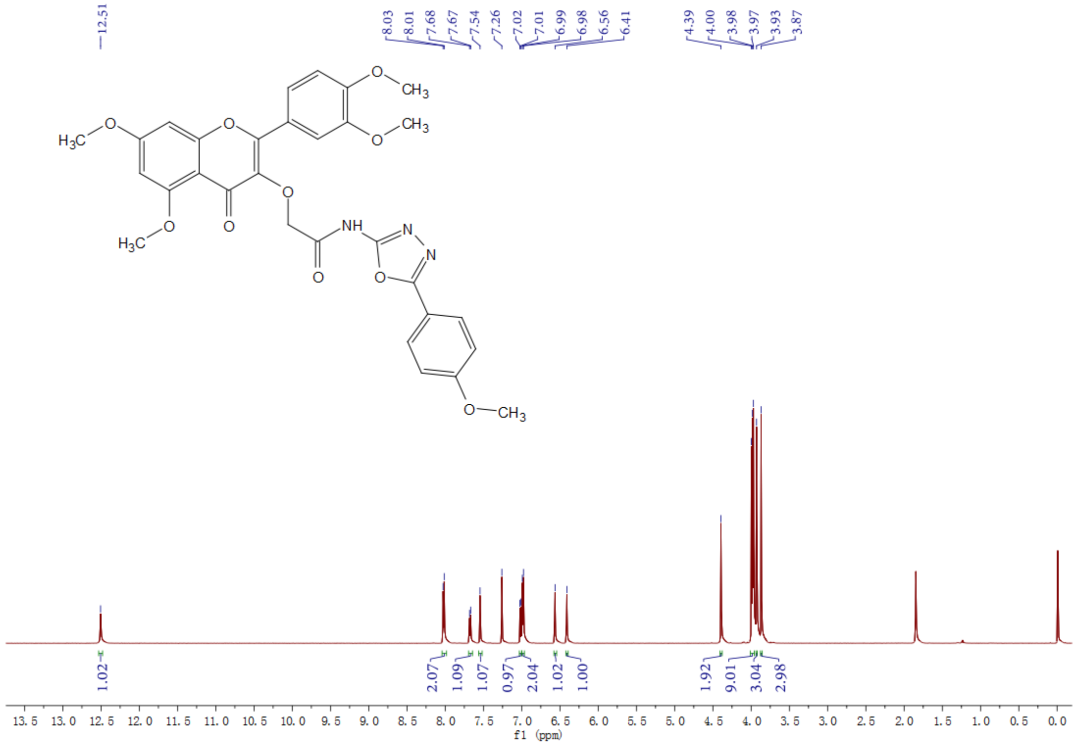

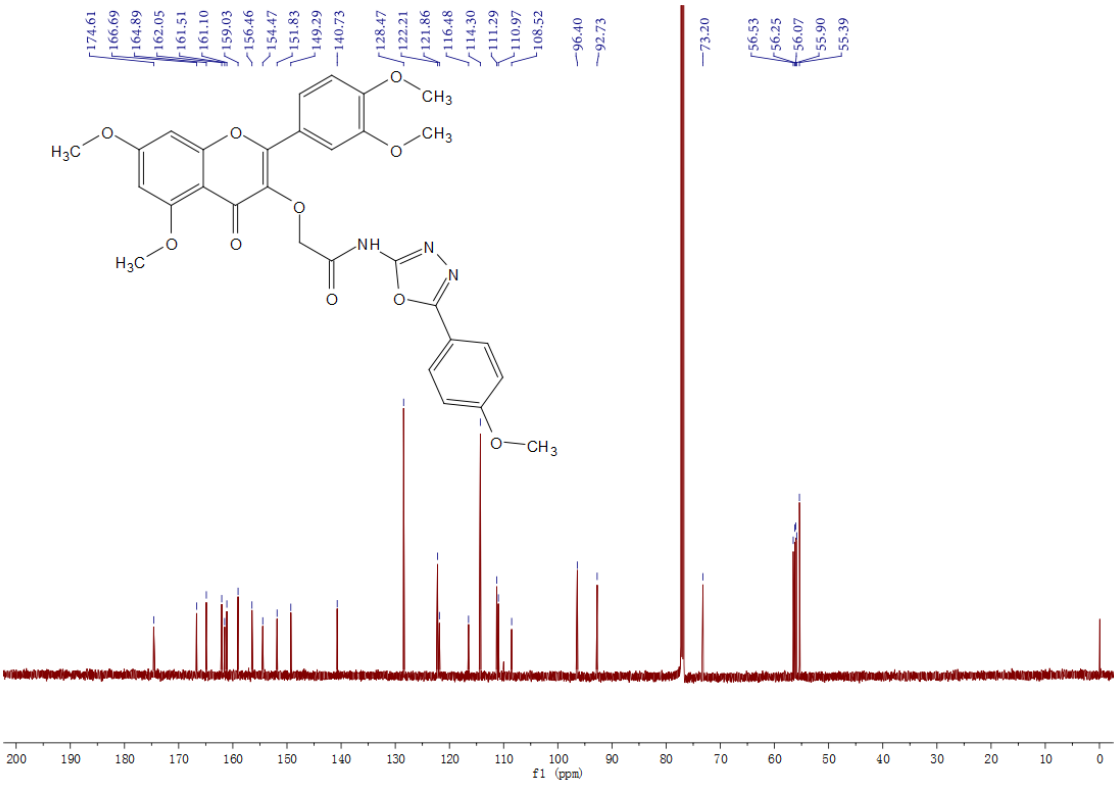

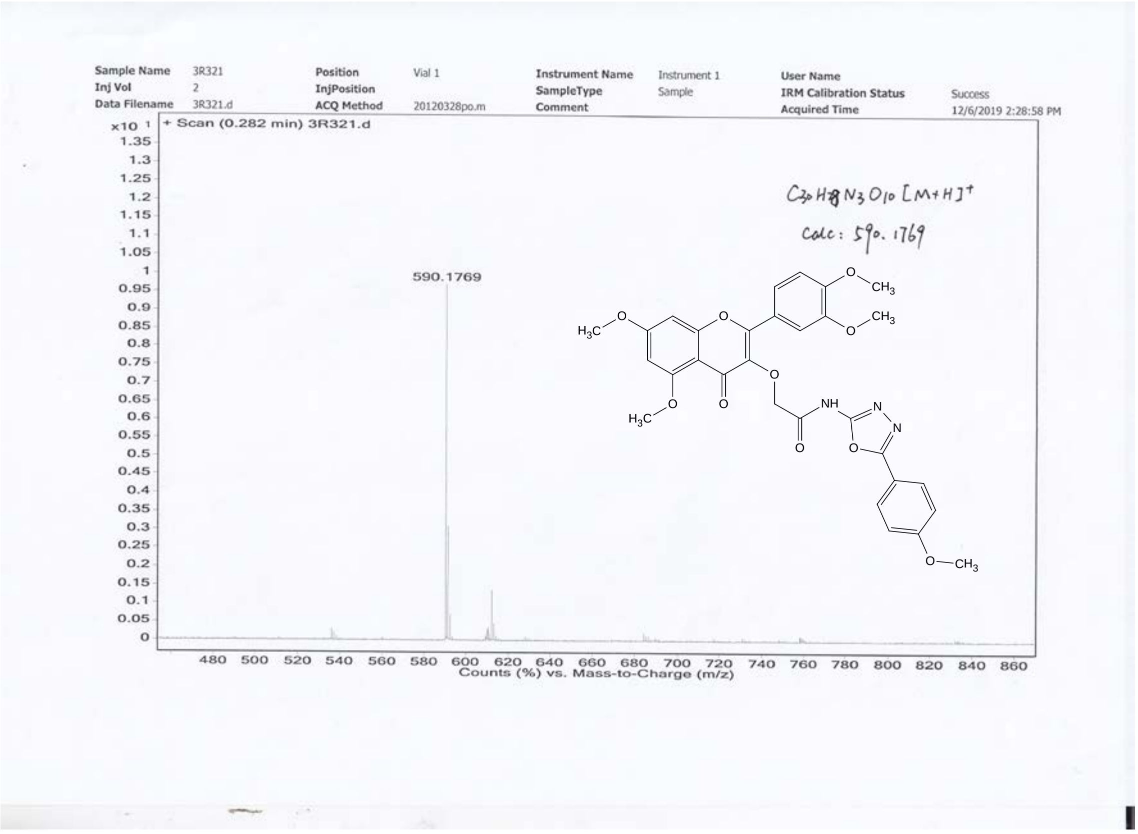

The spectrum of ^{1}H NMR, ^{13}C NMR and HRMS of compound B22

B23

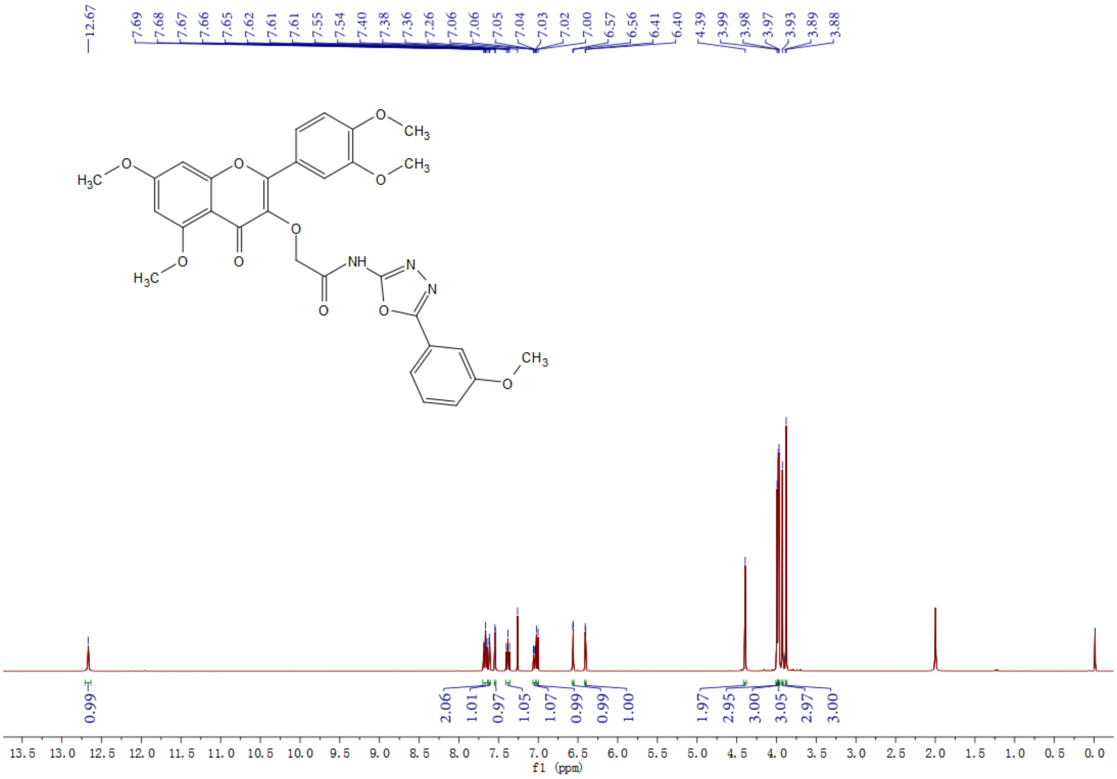

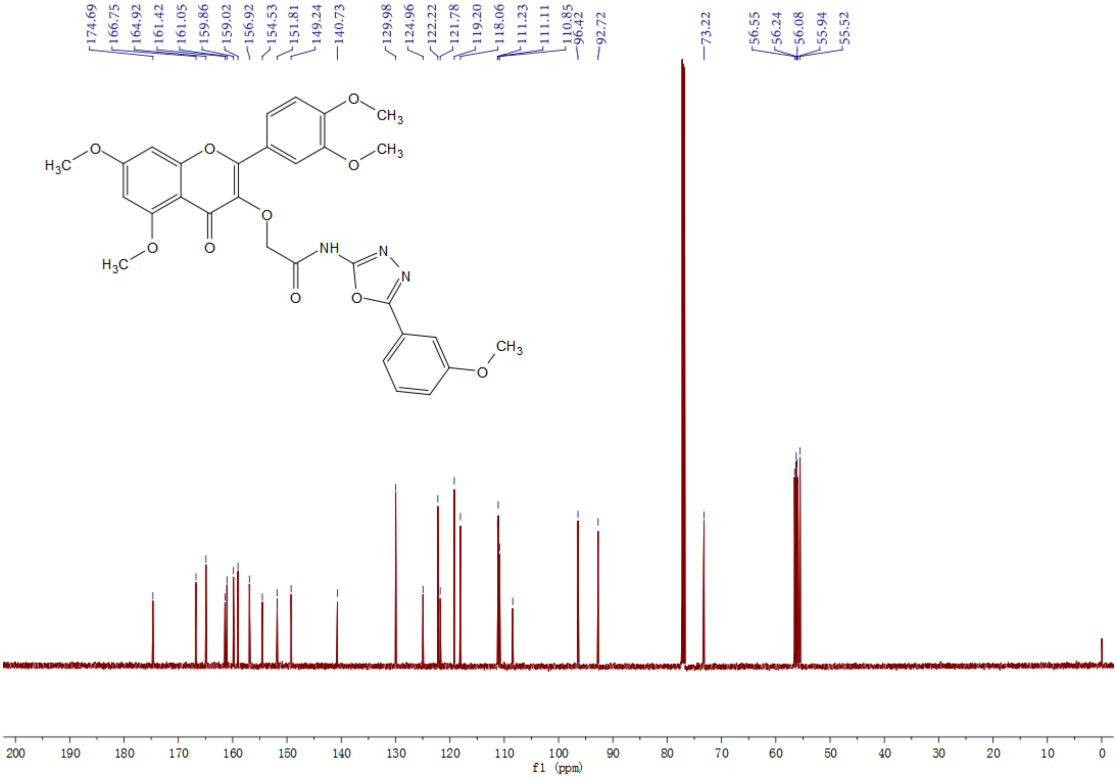

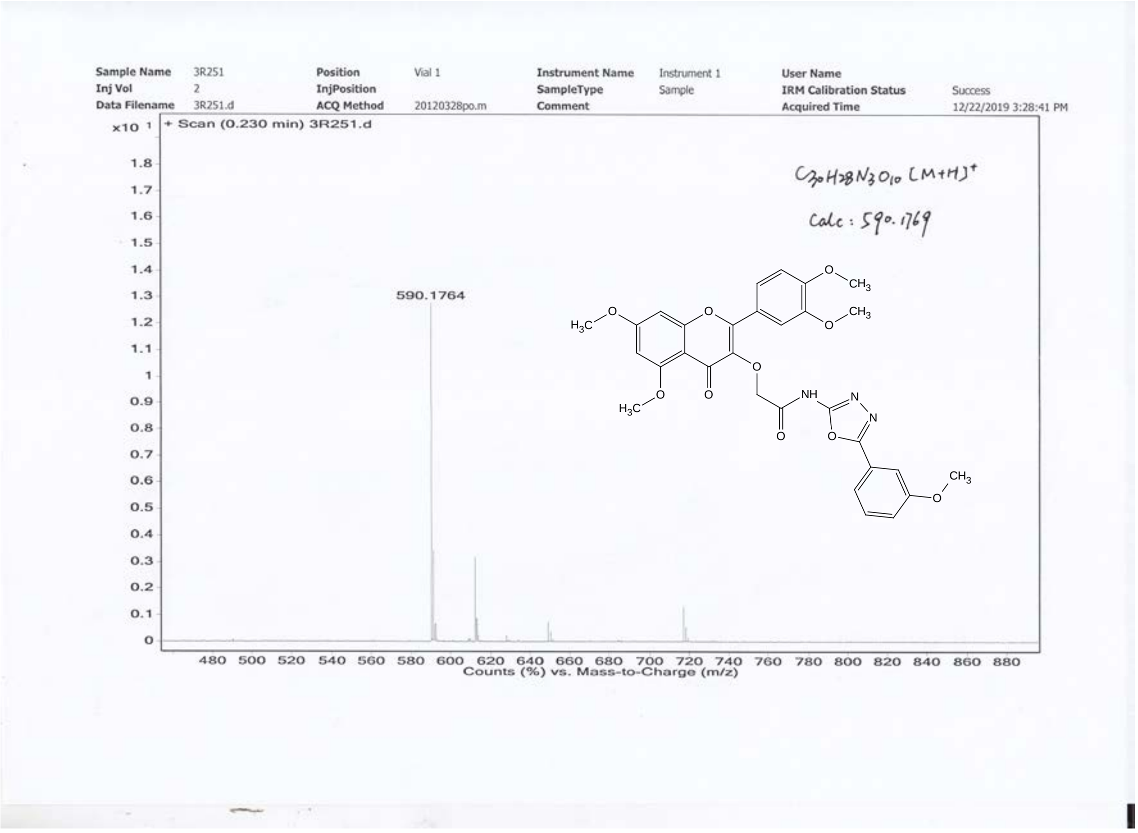

The spectrum of ^{1}H NMR, ^{13}C NMR and HRMS of compound B23

B24

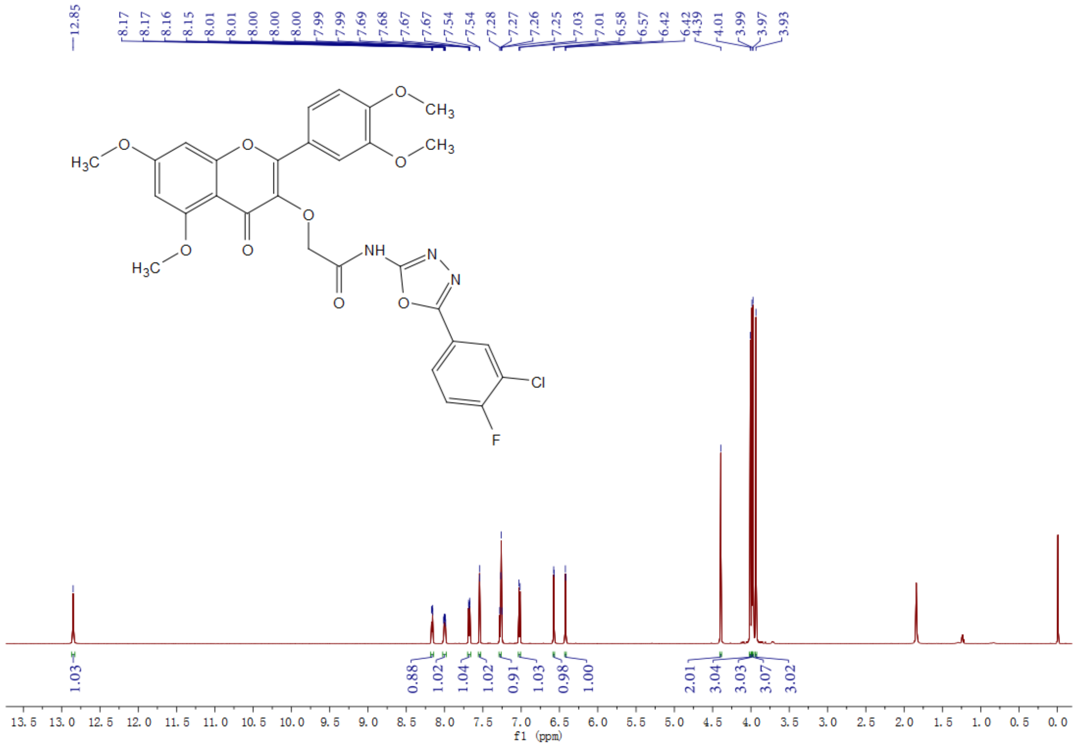

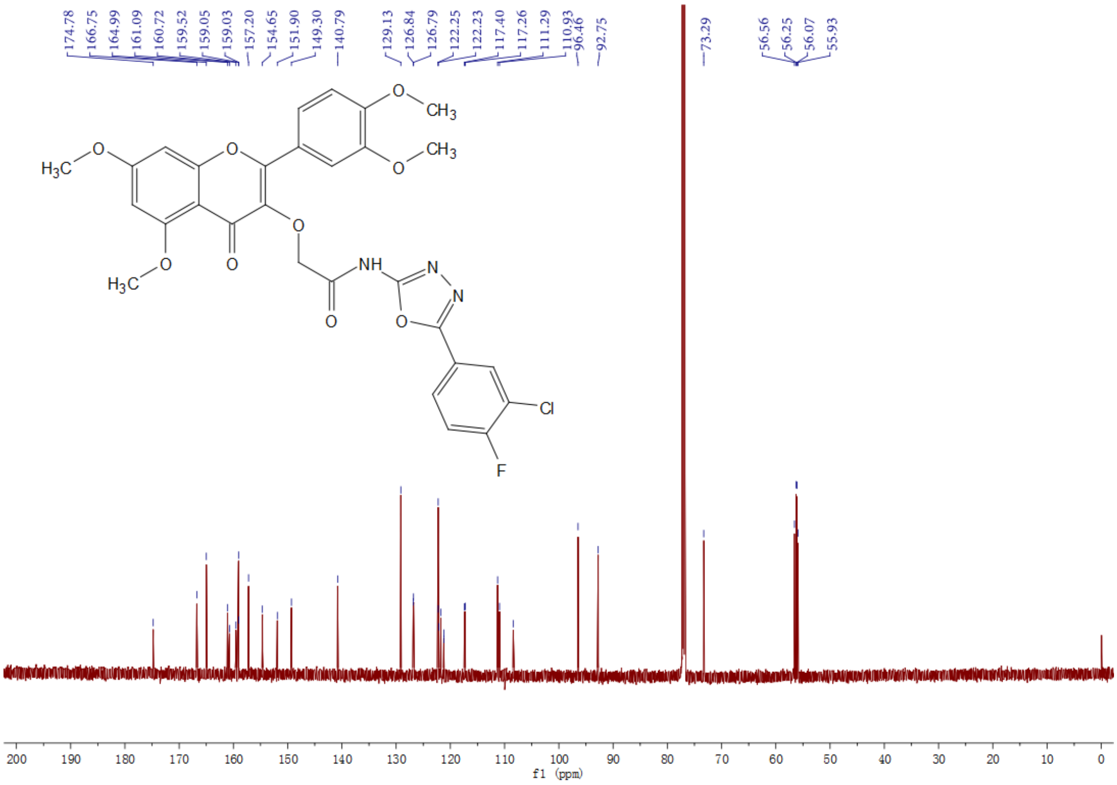

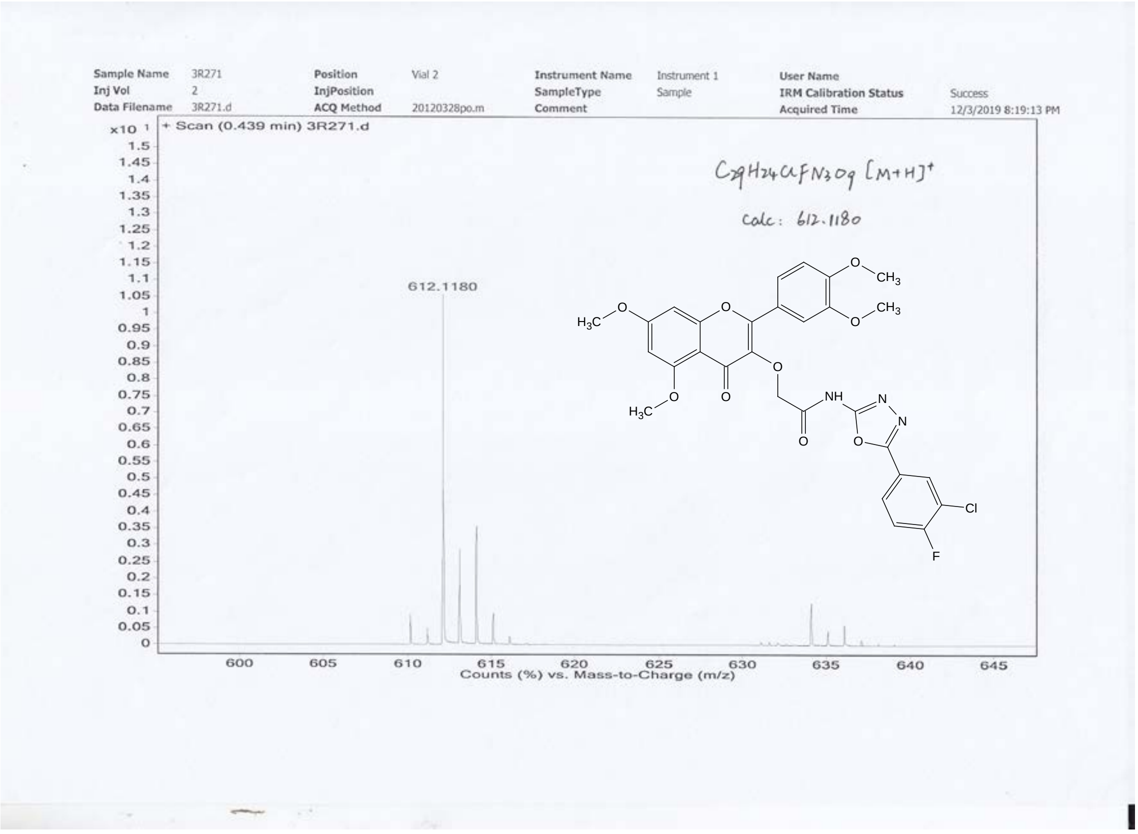

The spectrum of ^{1}H NMR, ^{13}C NMR and HRMS of compound B24

B25

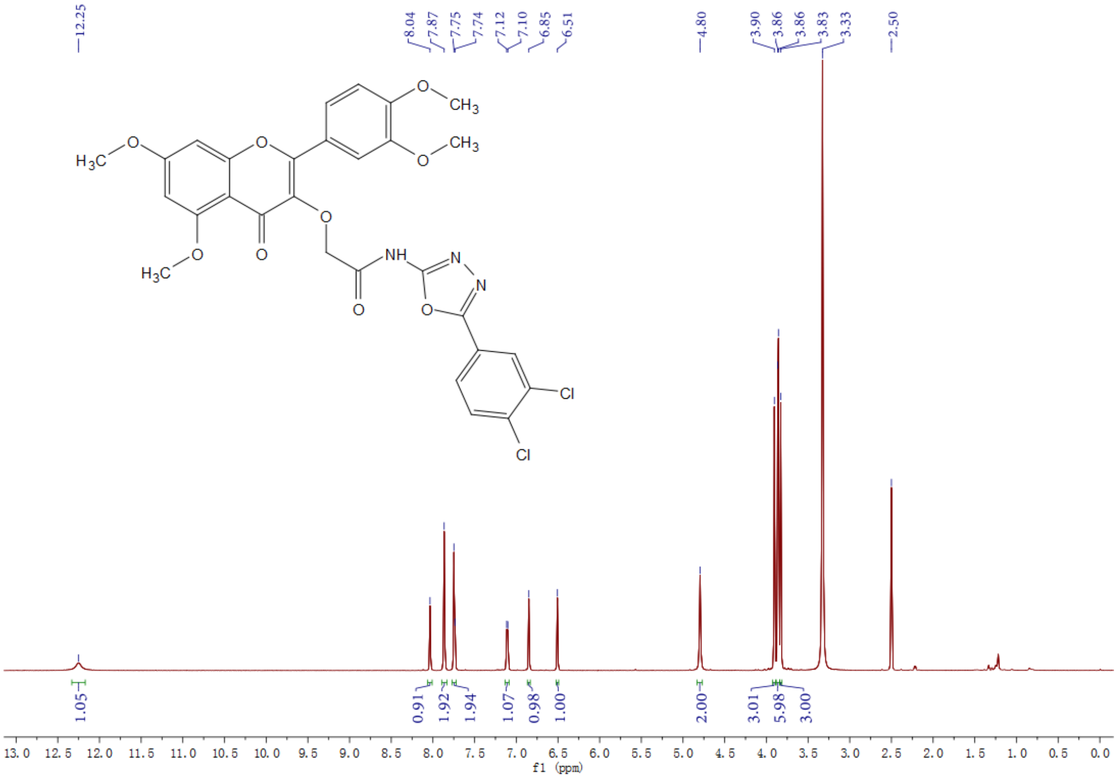
12.25
8.04 7.87 7.75 7.74 7.12 7.10 6.85 6.51
4.80
3.90 3.86 3.86 3.83 3.33
2.50
1.05 0.91 1.92 1.94 1.07 0.98 1.00 2.00 3.01 5.98 3.00
f1 (ppm)

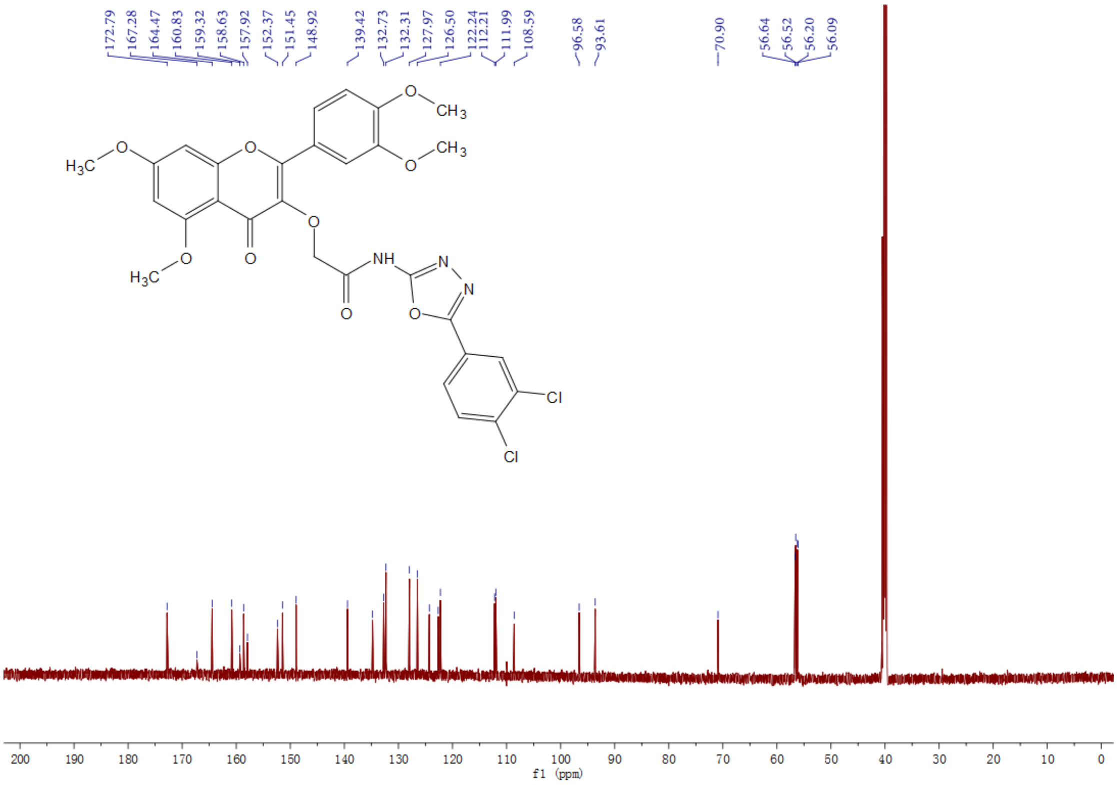
172.79 167.28 164.47 160.83 159.32 158.63 157.92 152.37 151.45 148.92
139.42 132.73 132.31 127.97 126.50 122.24 112.21 111.99 108.59
96.58 93.61
70.90
56.64 56.52 56.20 56.09
f1 (ppm)

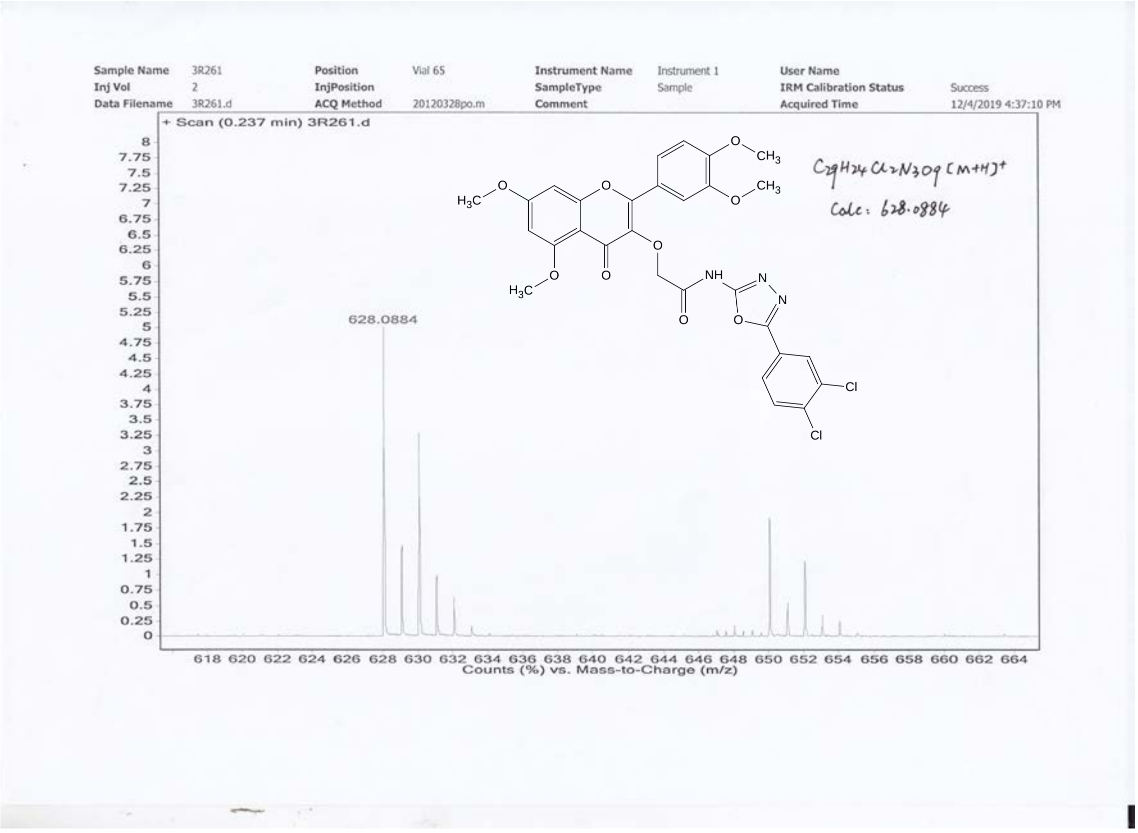

The spectrum of ^{1}H NMR, ^{13}C NMR and HRMS of compound B25

B26

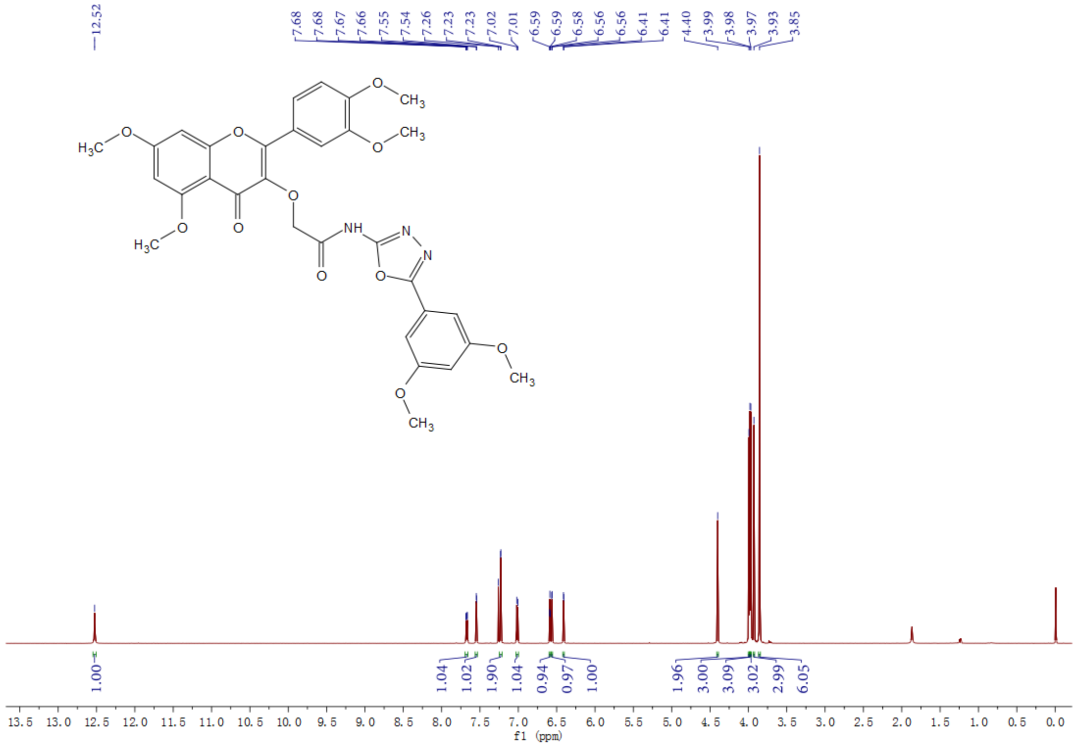

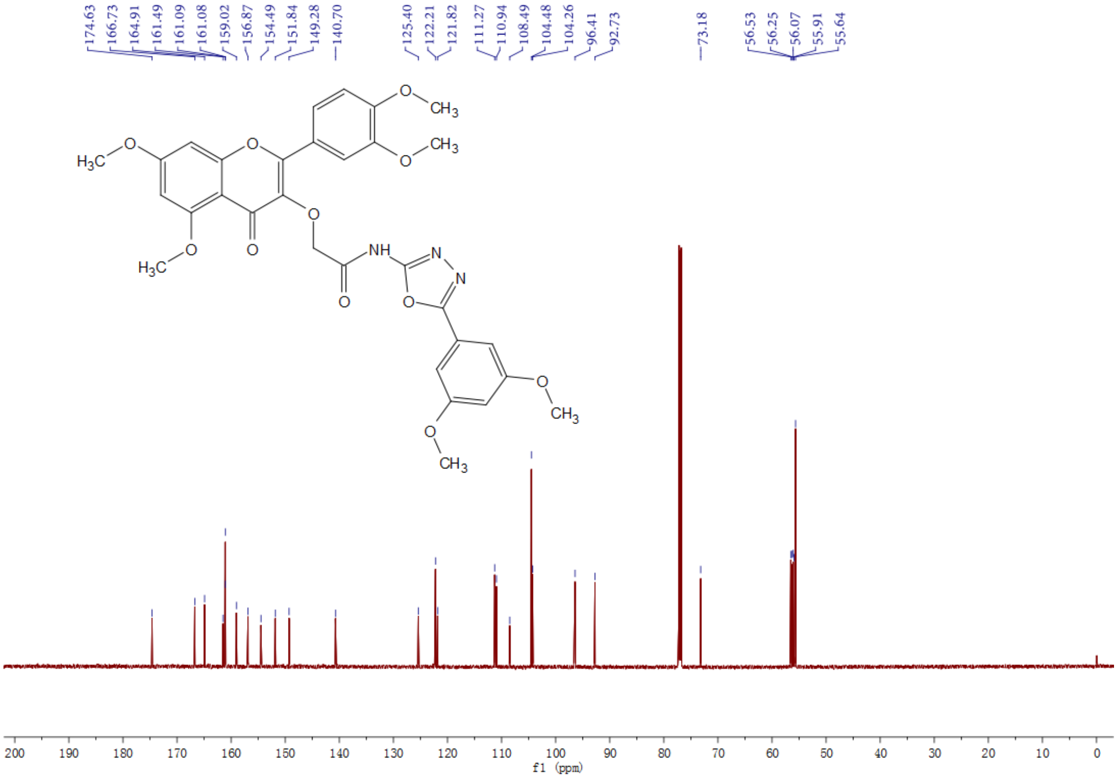

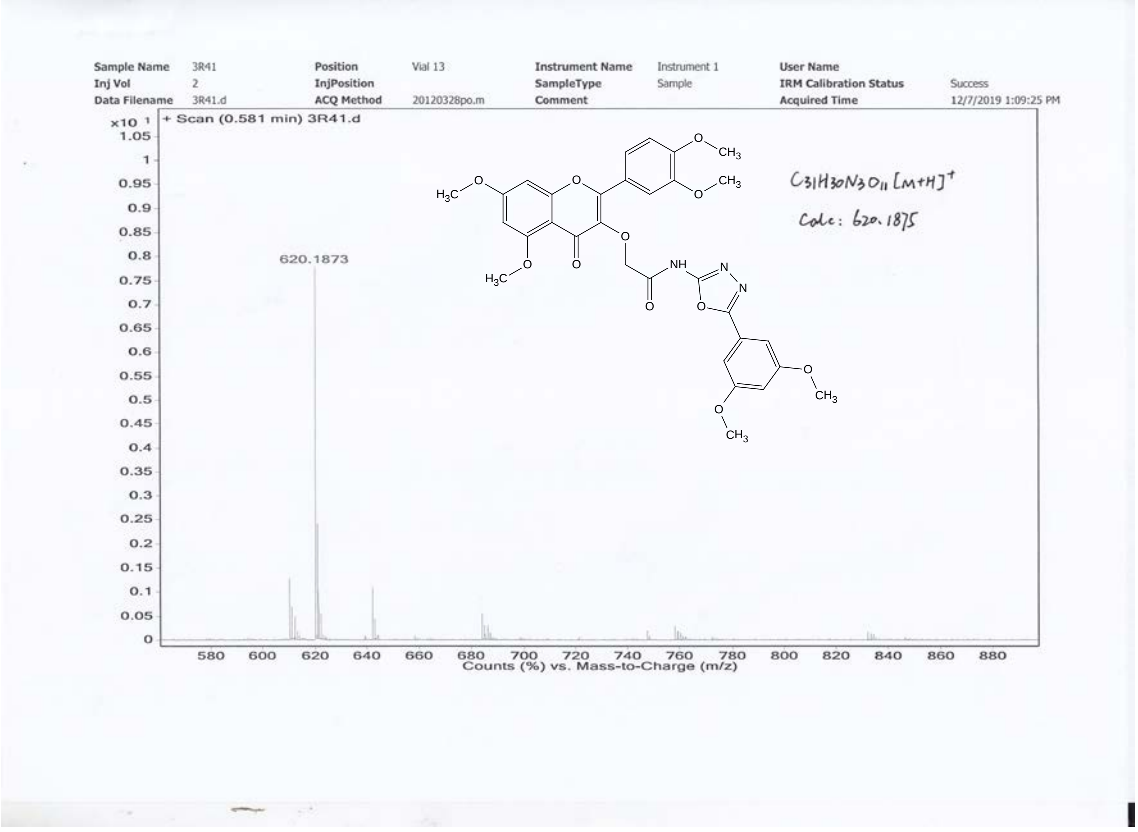

The spectrum of ^{1}H NMR, ^{13}C NMR and HRMS of compound B26

B27

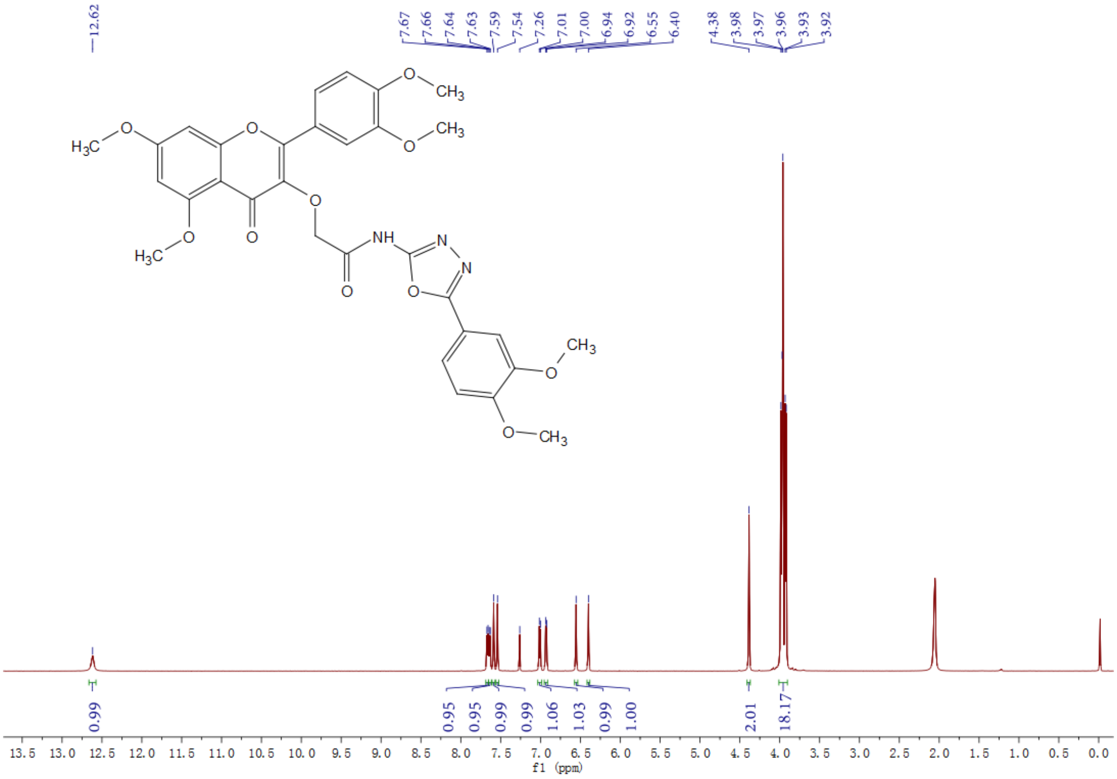

12.62
7.67
7.66
7.64
7.63
7.59
7.54
7.26
7.01
7.00
6.94
6.92
6.55
6.40
4.38
3.98
3.97
3.96
3.93
3.92
0.99
0.95
0.95
0.99
0.99
1.06
1.03
0.99
1.00
2.01
18.17
f1 (ppm)

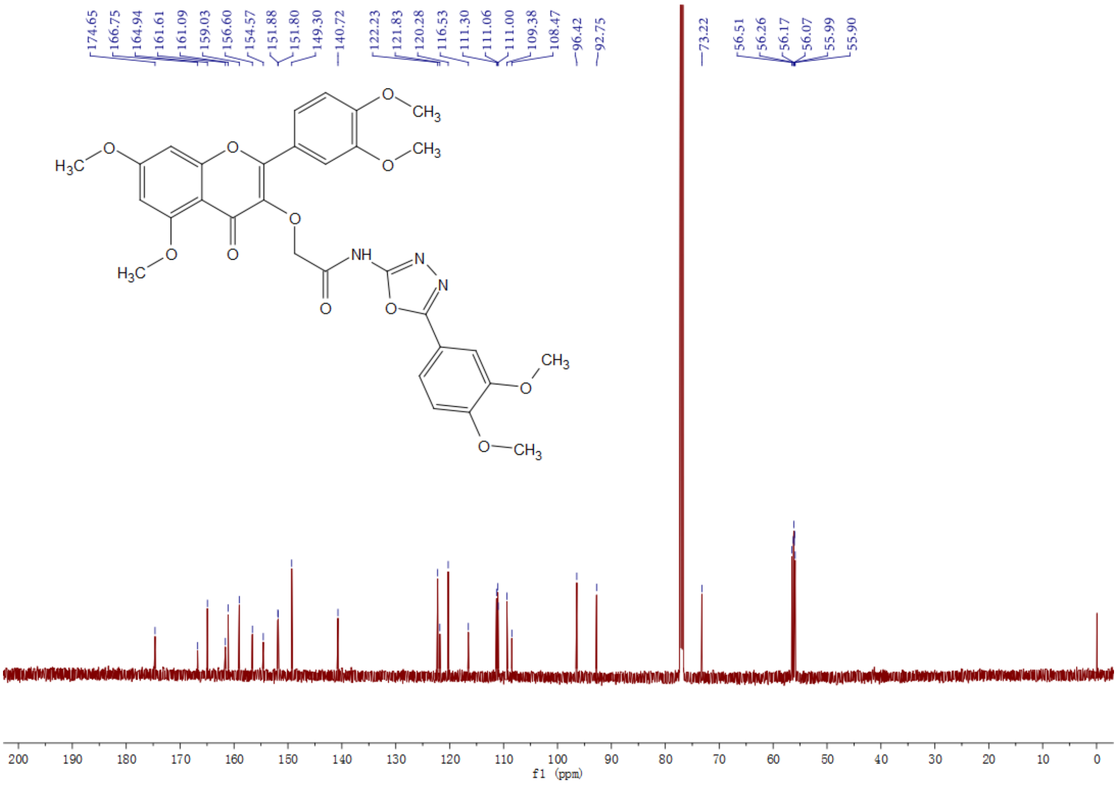

174.65
166.75
164.94
161.61
161.09
159.03
156.60
154.57
151.88
151.80
149.30
140.72
122.23
121.83
120.28
116.53
111.30
111.06
111.00
109.38
108.47
96.42
92.75
73.22
56.51
56.26
56.17
56.07
55.99
55.90
f1 (ppm)

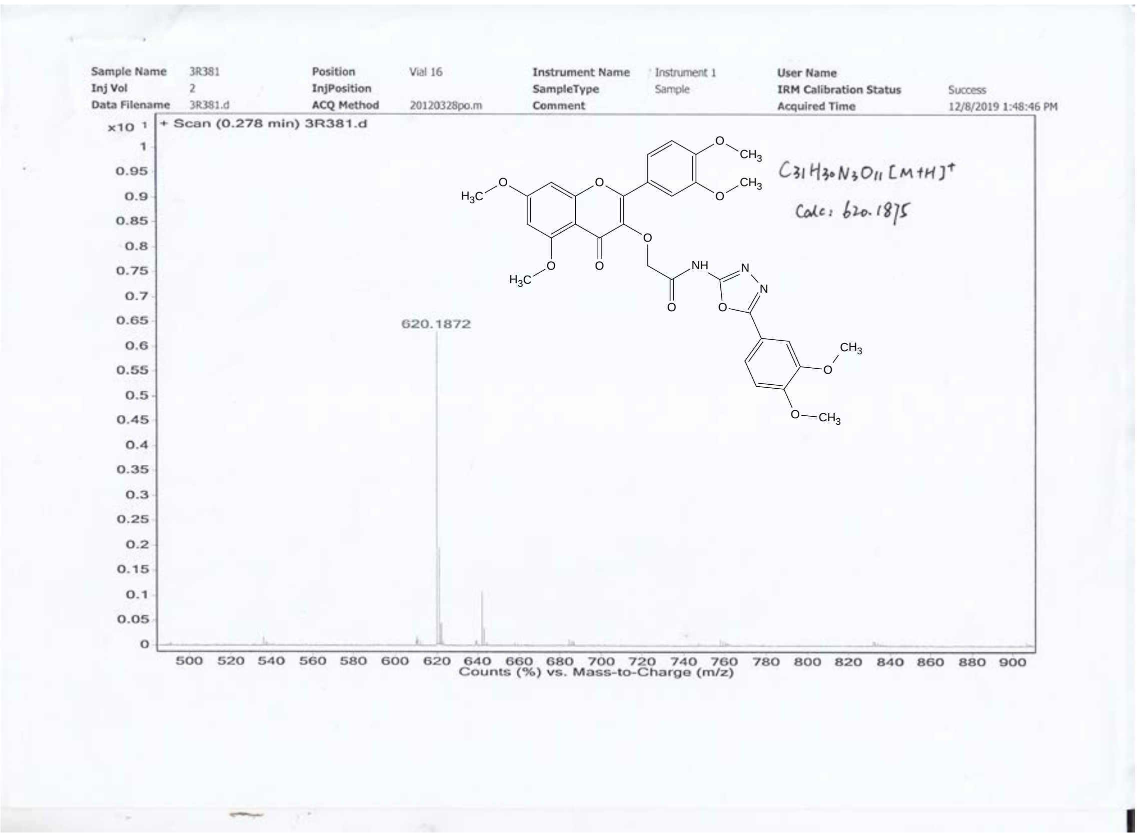

The spectrum of ^{1}H NMR, ^{13}C NMR and HRMS of compound B27

B28

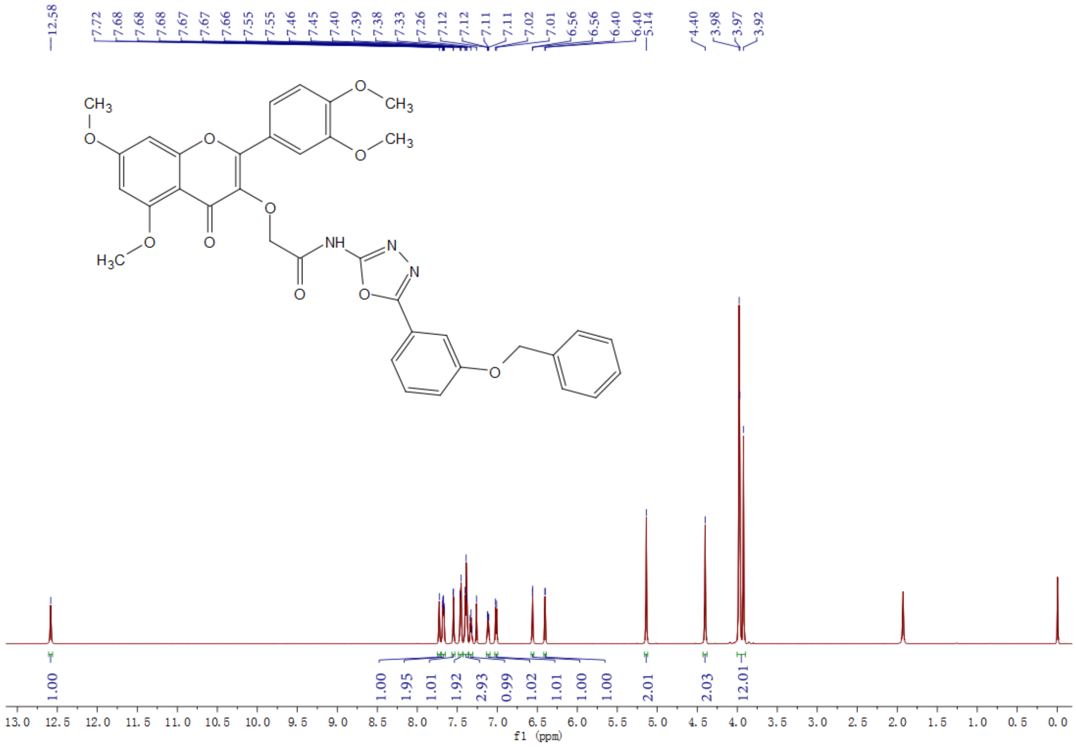

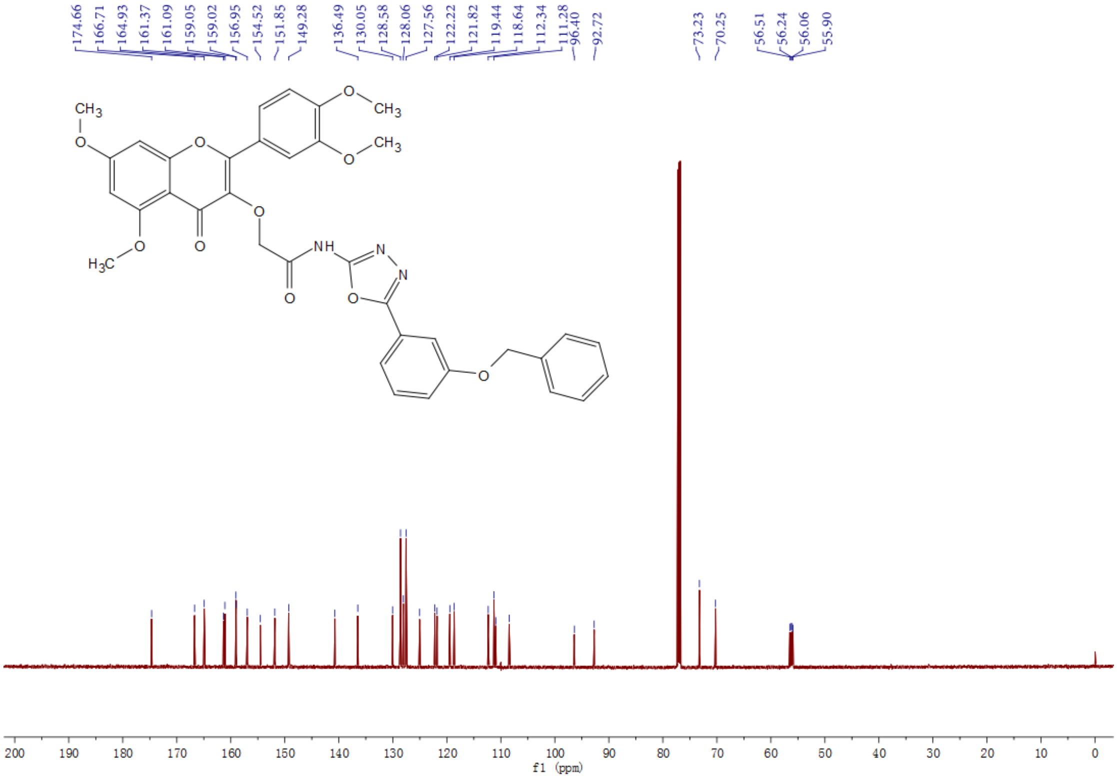

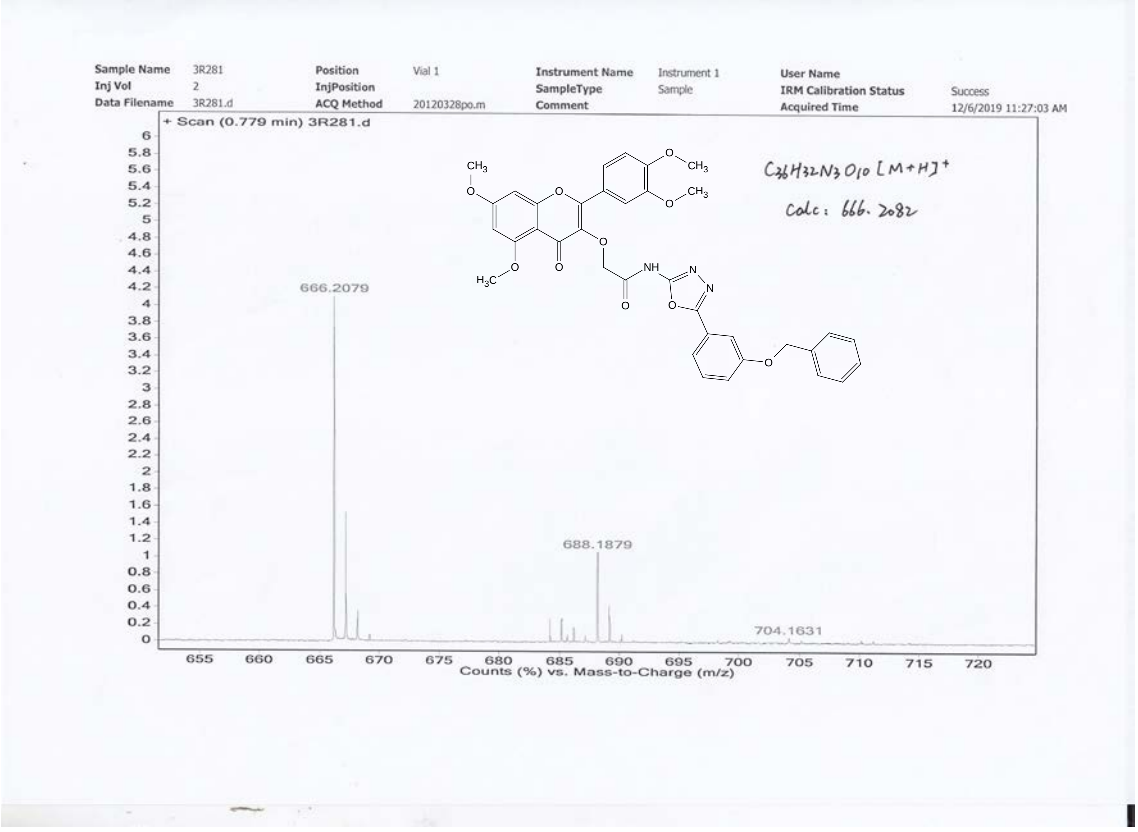

The spectrum of ^{1}H NMR, ^{13}C NMR and HRMS of compound B28

B29

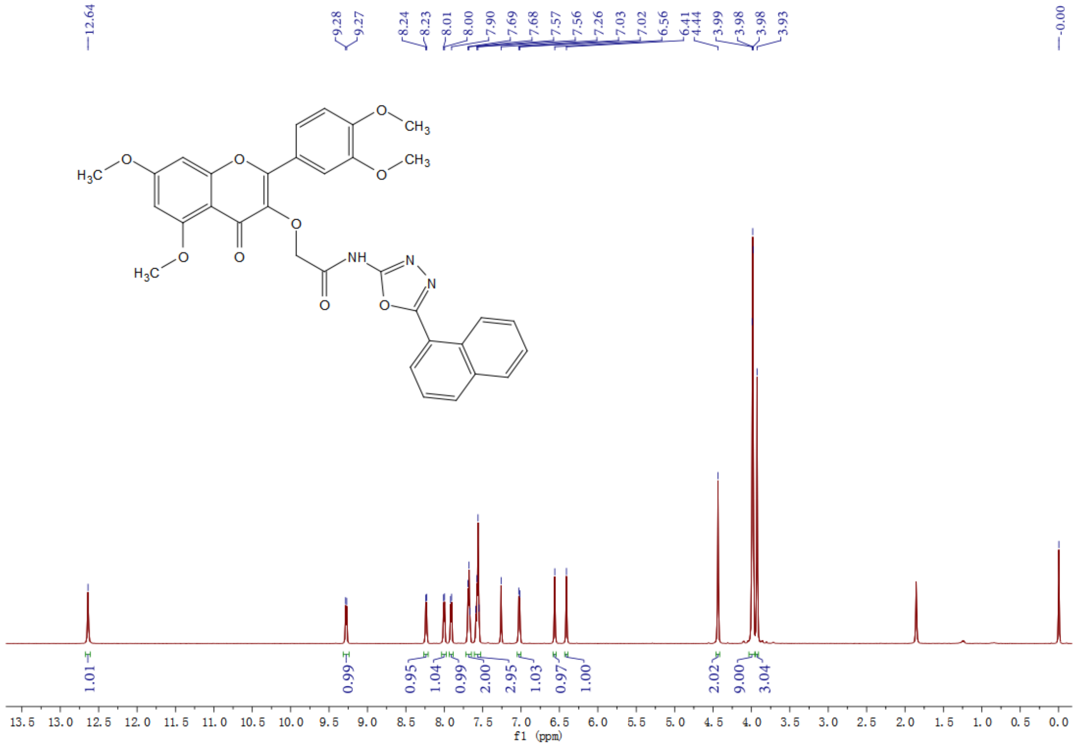

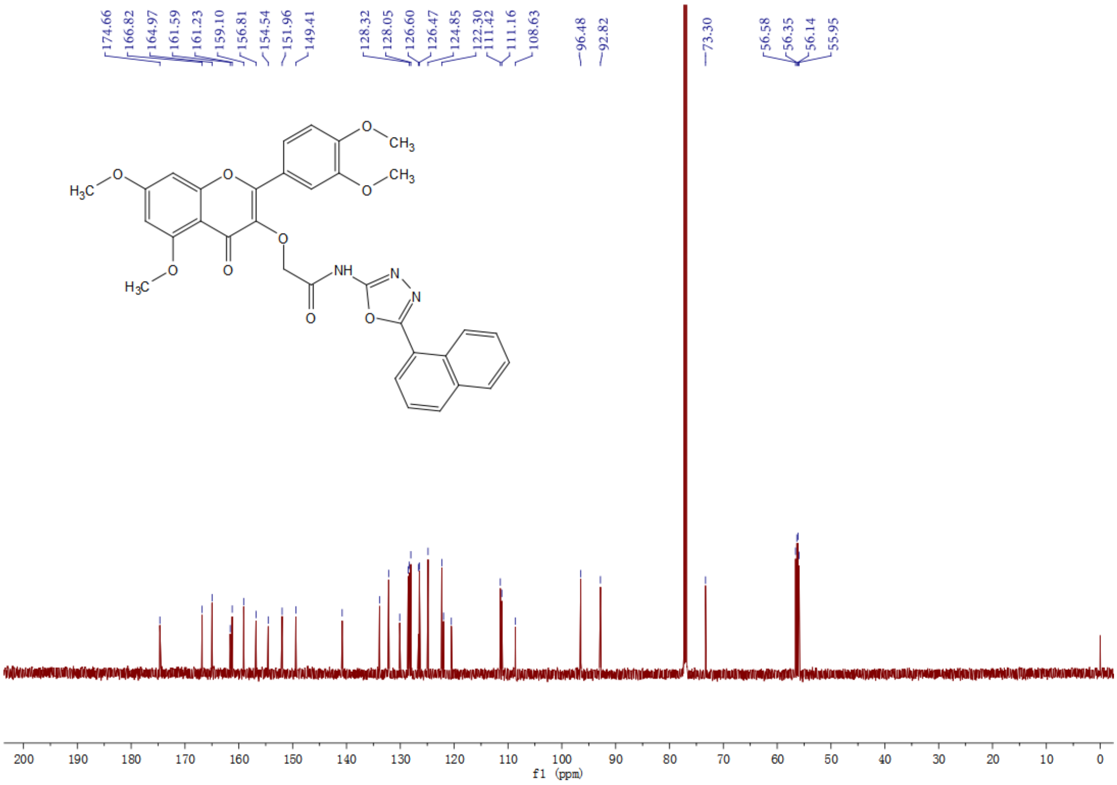

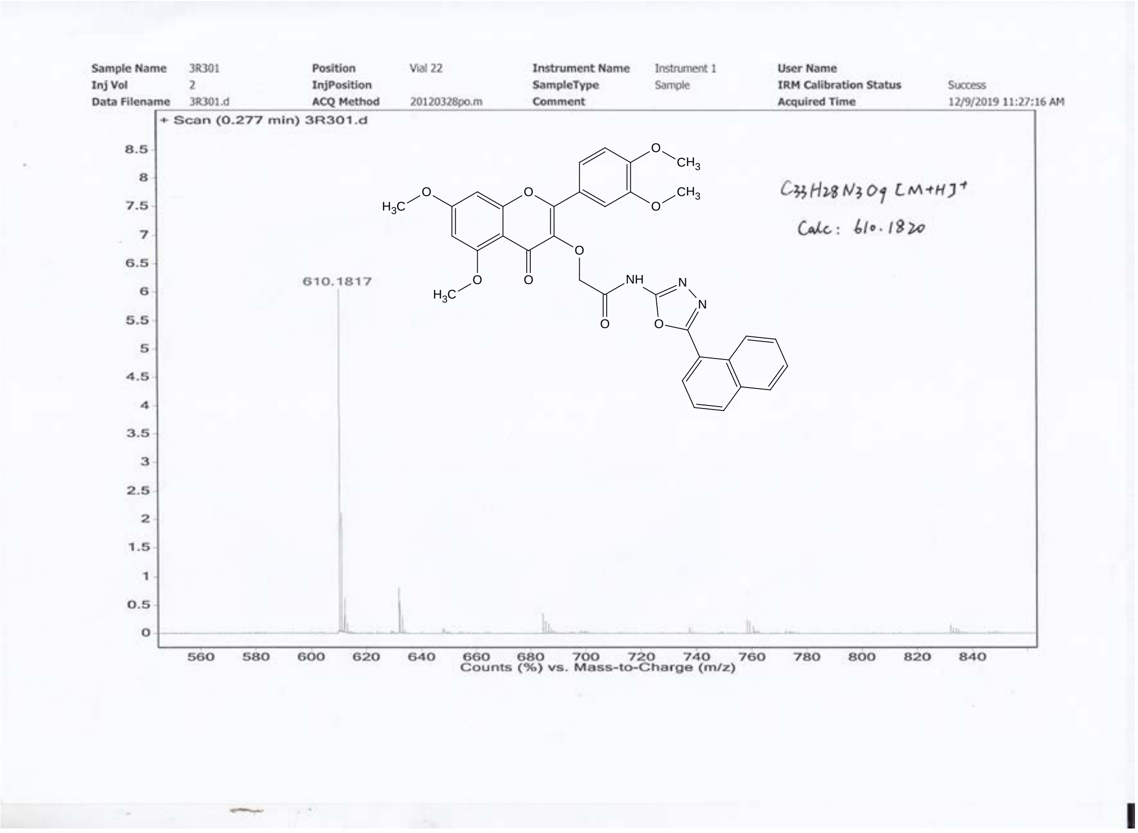

The spectrum of ¹H NMR, ¹³C NMR and HRMS of compound B29

B30

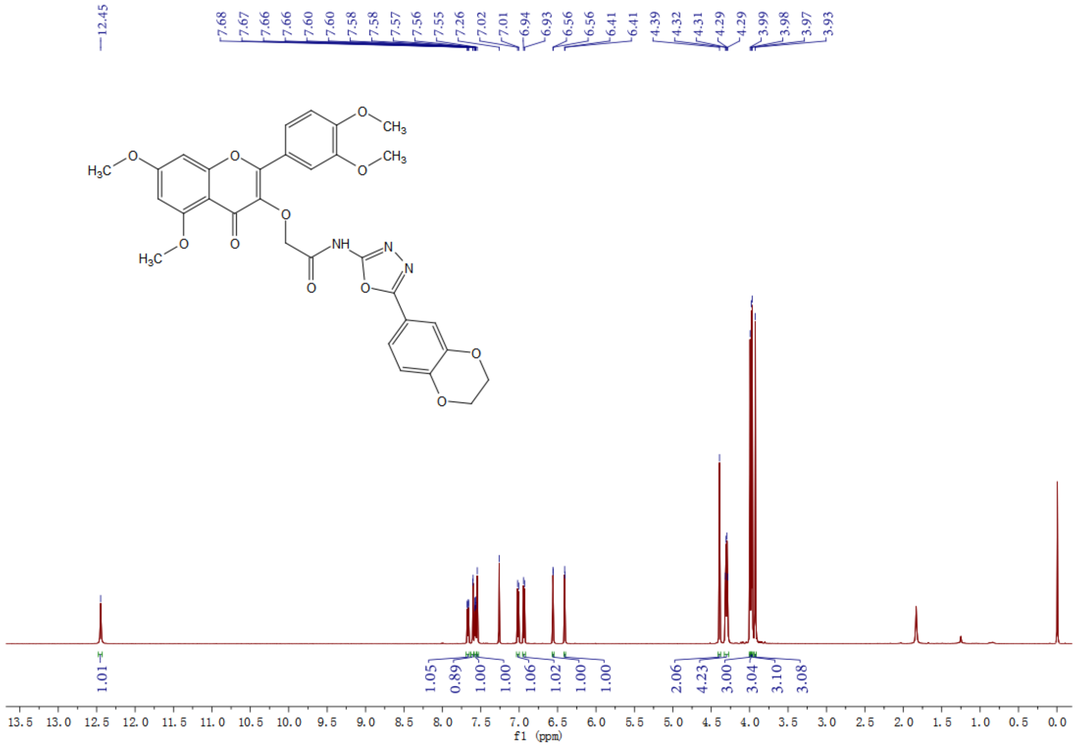

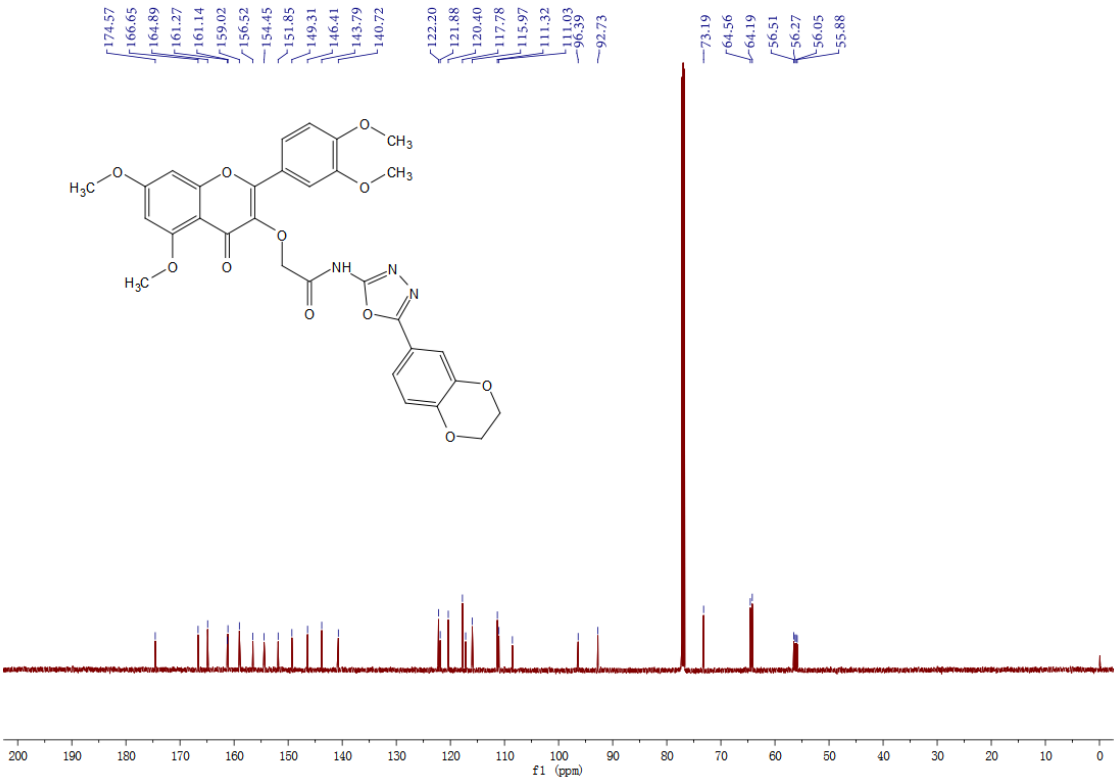

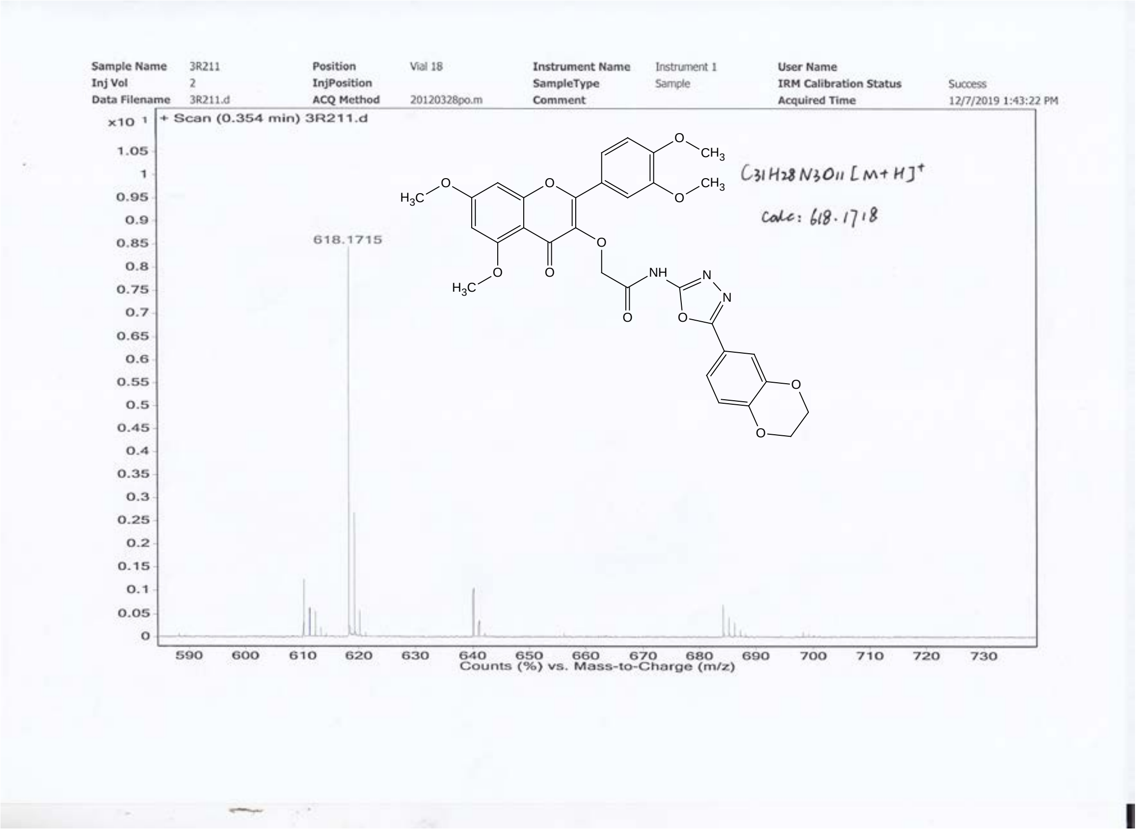

The spectrum of ¹H NMR, ¹³C NMR and HRMS of compound B30

B31

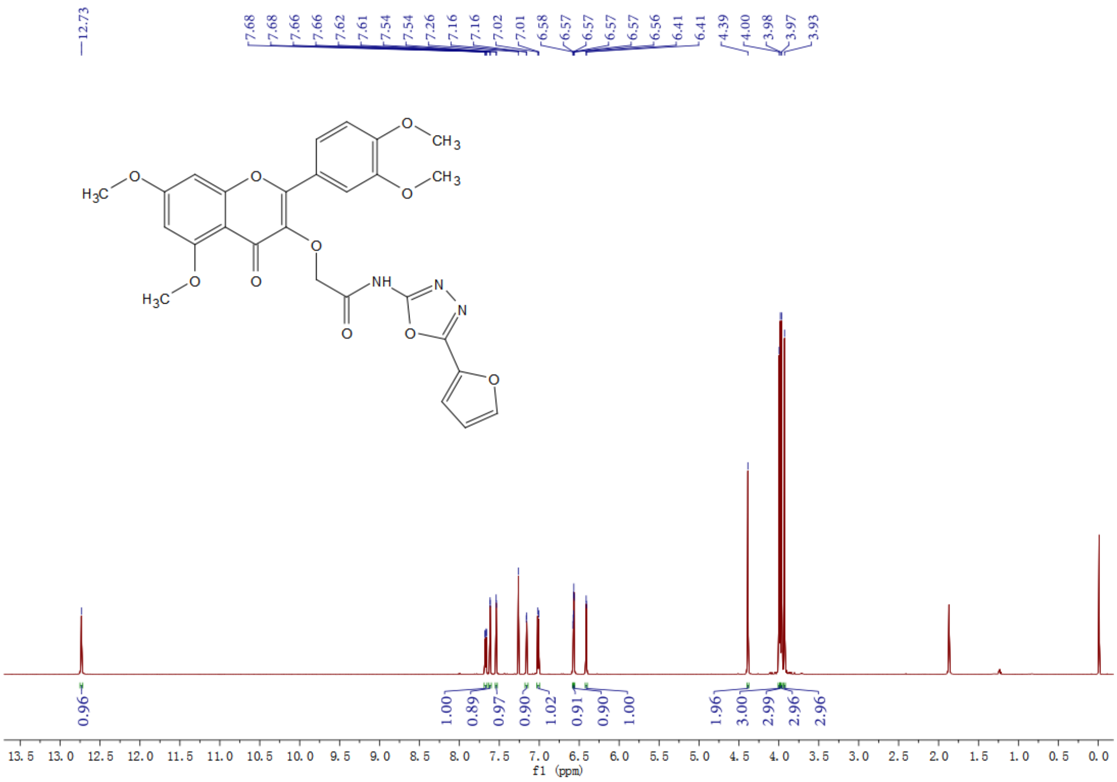
12.73
7.68
7.68
7.66
7.66
7.62
7.61
7.54
7.54
7.26
7.16
7.16
7.02
7.01
6.58
6.57
6.57
6.57
6.57
6.56
6.41
6.41
4.39
4.00
3.98
3.97
3.93
0.96
1.00
0.89
0.97
0.90
1.02
0.91
0.90
1.00
1.96
3.00
2.99
2.96
2.96
f1 (ppm)

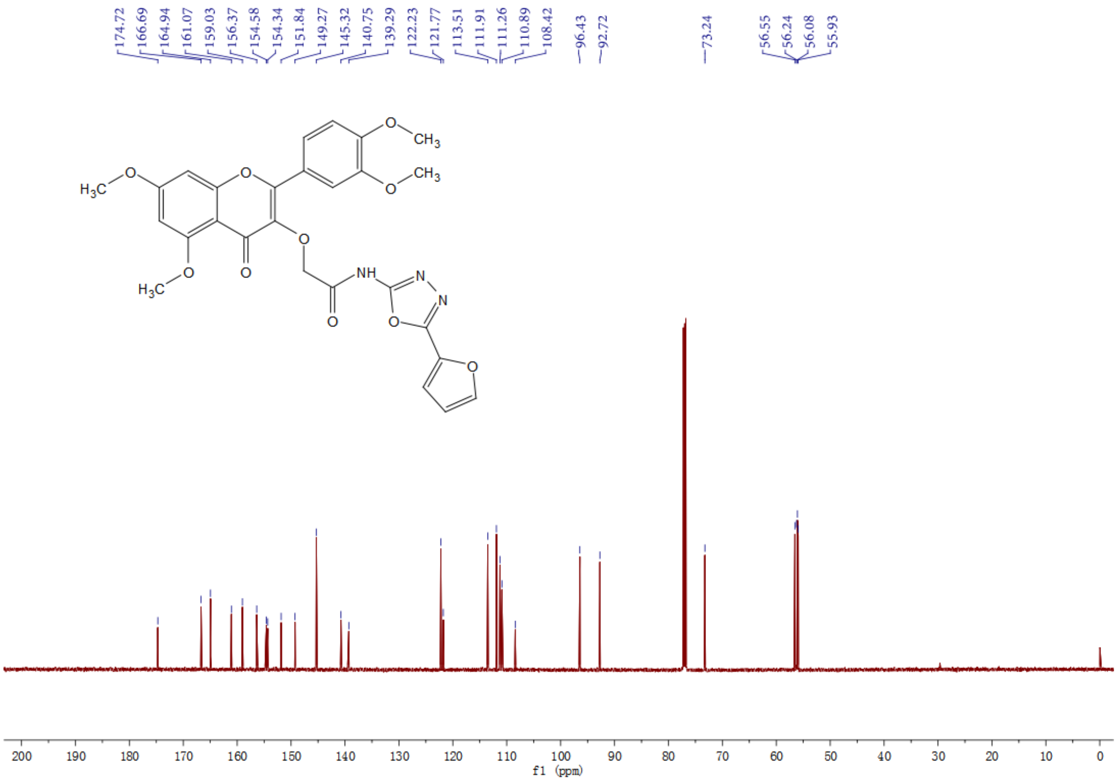
174.72
166.69
164.94
161.07
159.03
156.37
154.58
154.34
151.84
149.27
145.32
140.75
139.29
122.23
121.77
113.51
111.91
111.26
110.89
108.42
96.43
92.72
73.24
56.55
56.24
56.08
55.93
f1 (ppm)

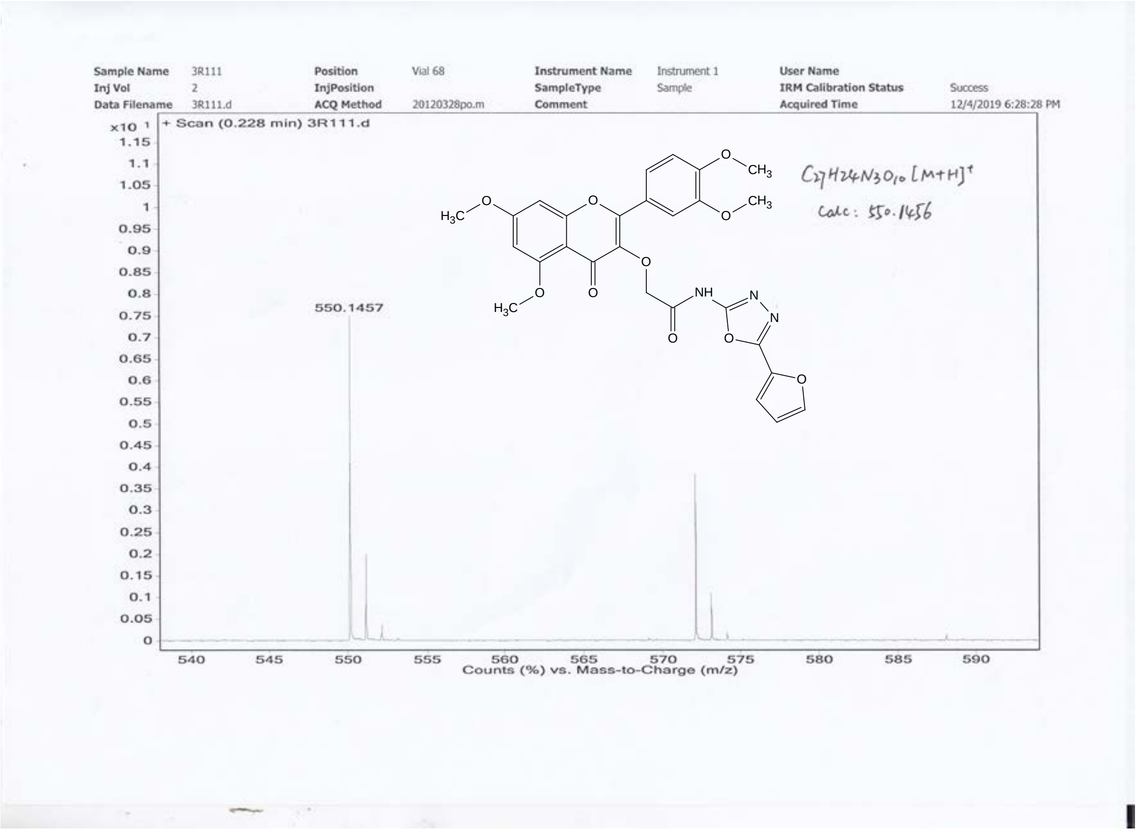

The spectrum of ^{1}H NMR, ^{13}C NMR and HRMS of compound B31

B32

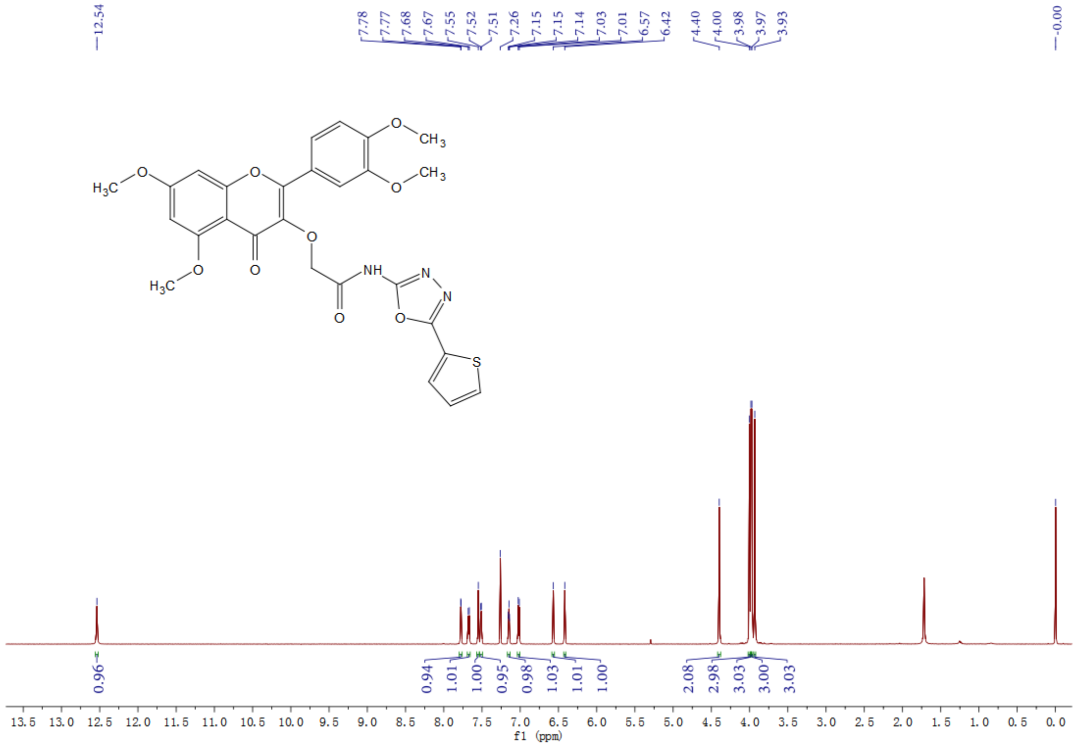

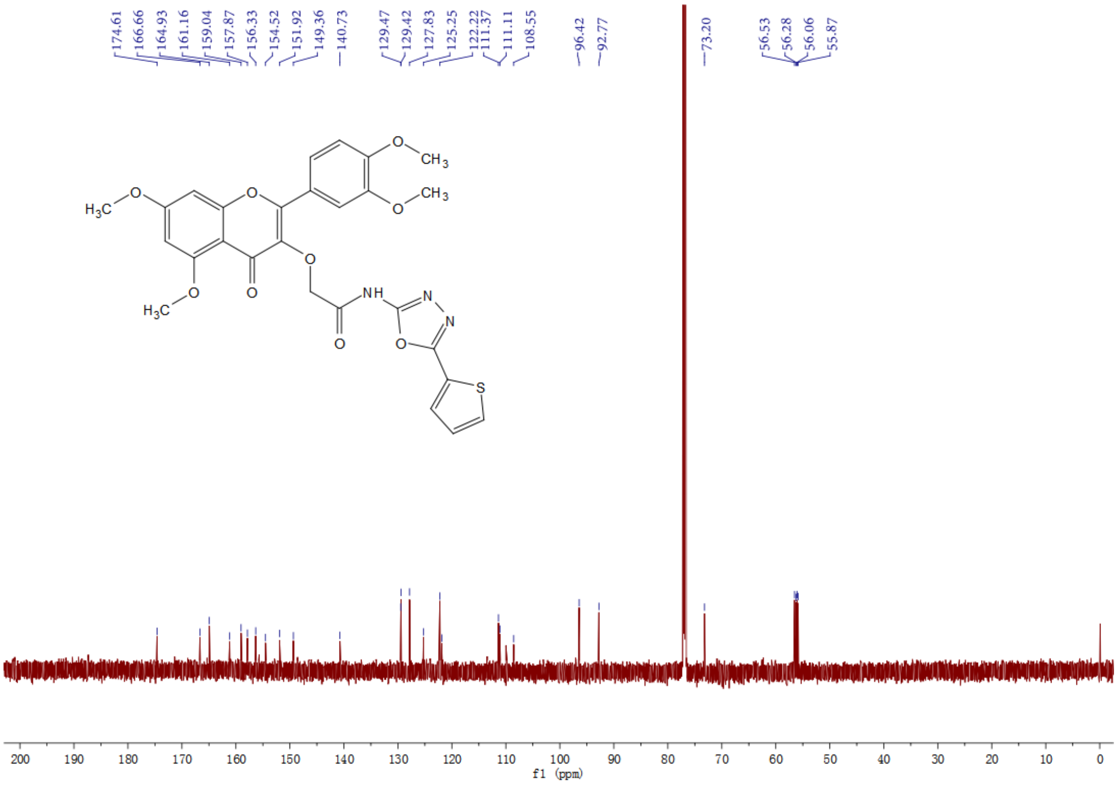

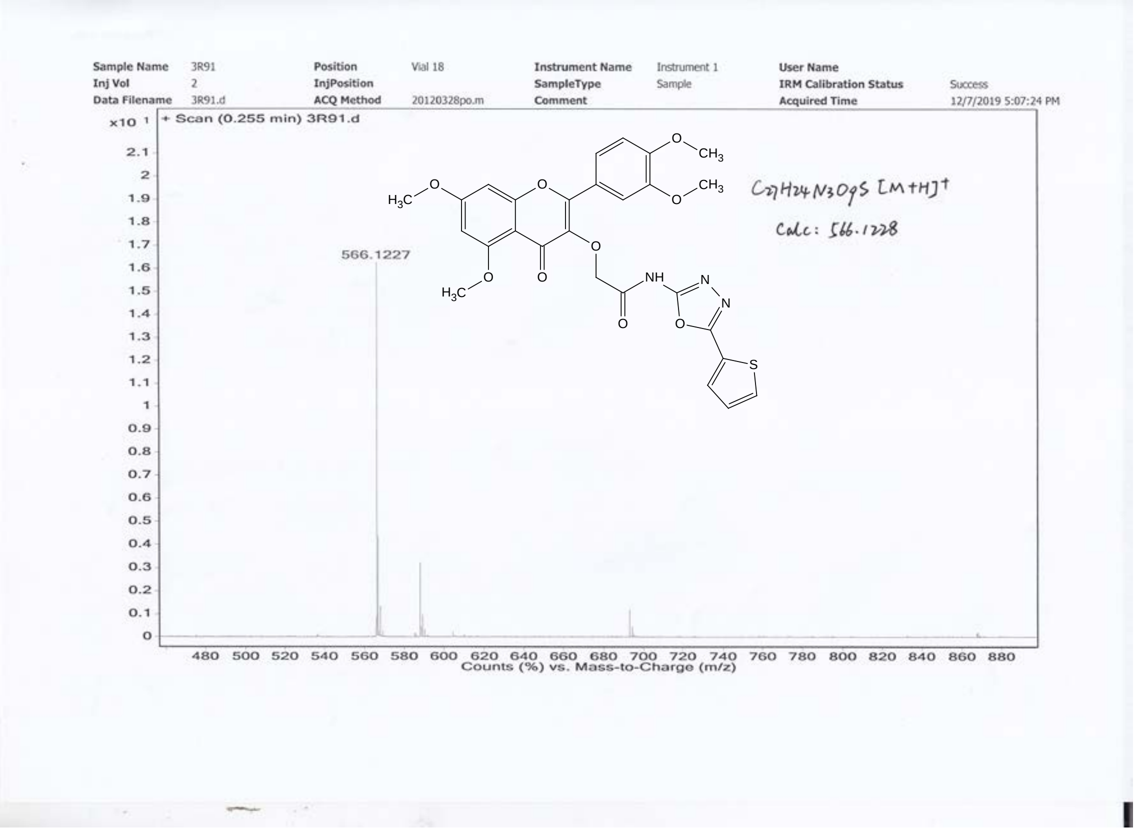

The spectrum of ^{1}H NMR, ^{13}C NMR and HRMS of compound B32

B33

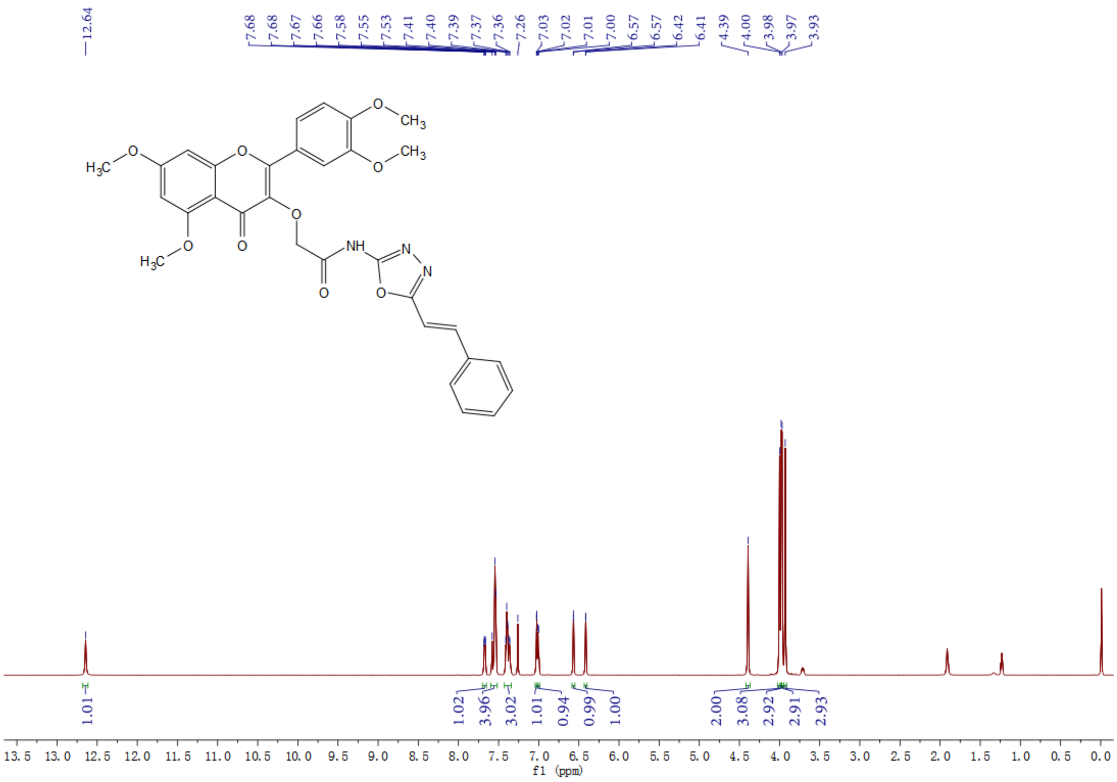

12.64
7.68 7.68 7.67 7.66 7.58 7.55 7.53 7.41 7.40 7.39 7.37 7.36 7.26 7.03 7.02 7.01 7.00 6.57 6.57 6.42 6.41 4.39 4.00 3.98 3.97 3.93
1.01
1.02 3.96 3.02 1.01 0.94 0.99 1.00 2.00 3.08 2.92 2.91 2.93
f1 (ppm)

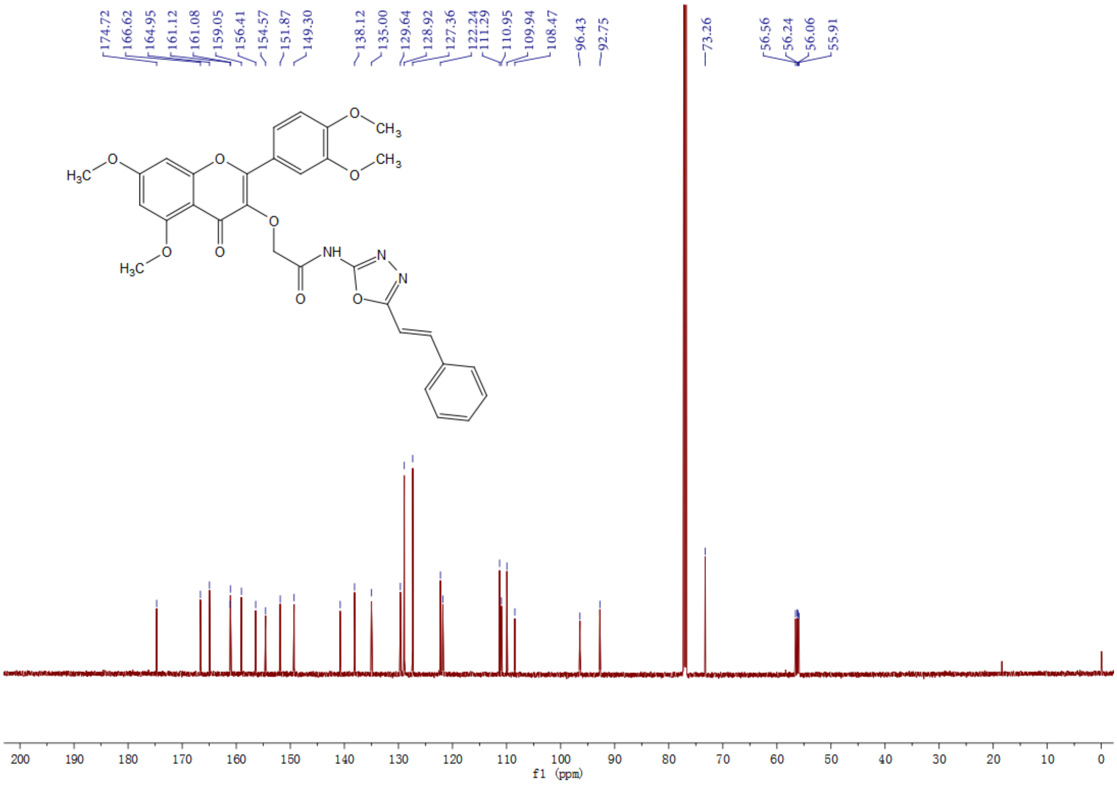

174.72 166.62 164.95 161.12 161.08 159.05 156.41 154.57 151.87 149.30 138.12 135.00 129.64 128.92 127.36 122.24 111.29 110.95 109.94 108.47 96.43 92.75 73.26 56.56 56.24 56.06 55.91
f1 (ppm)

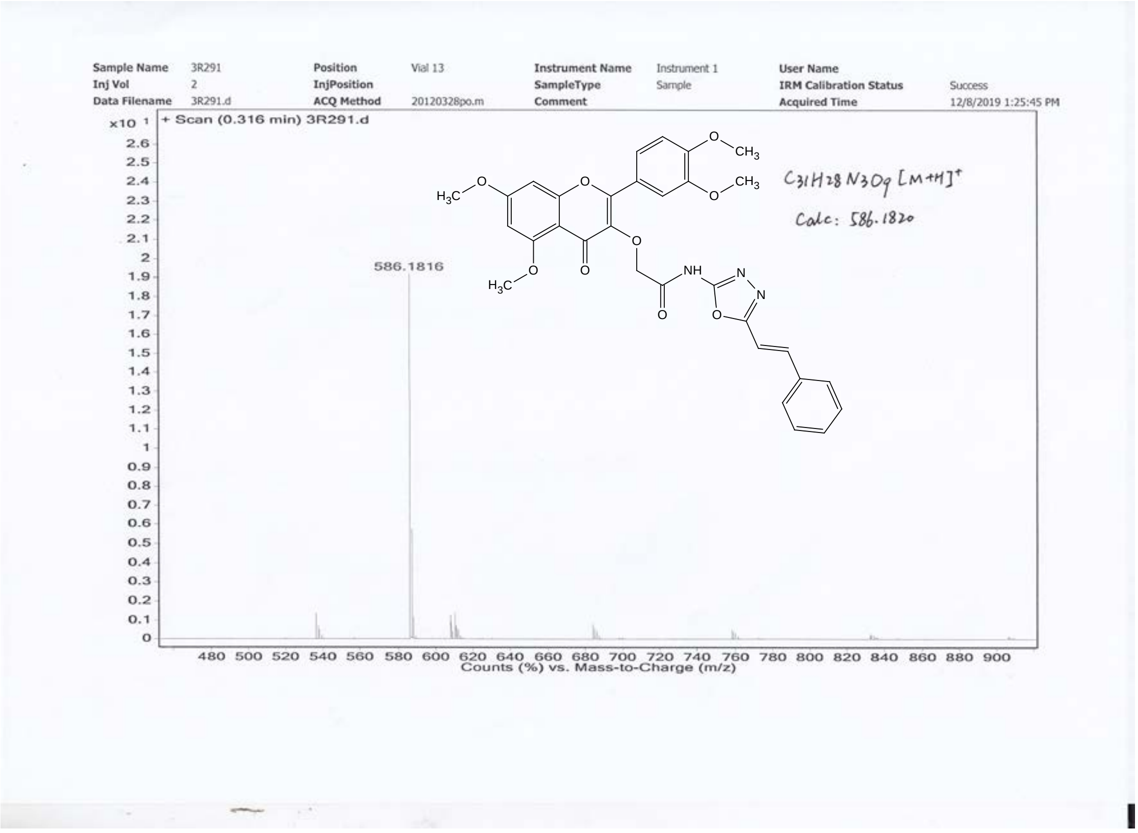

The spectrum of ^{1}H NMR, ^{13}C NMR and HRMS of compound B33

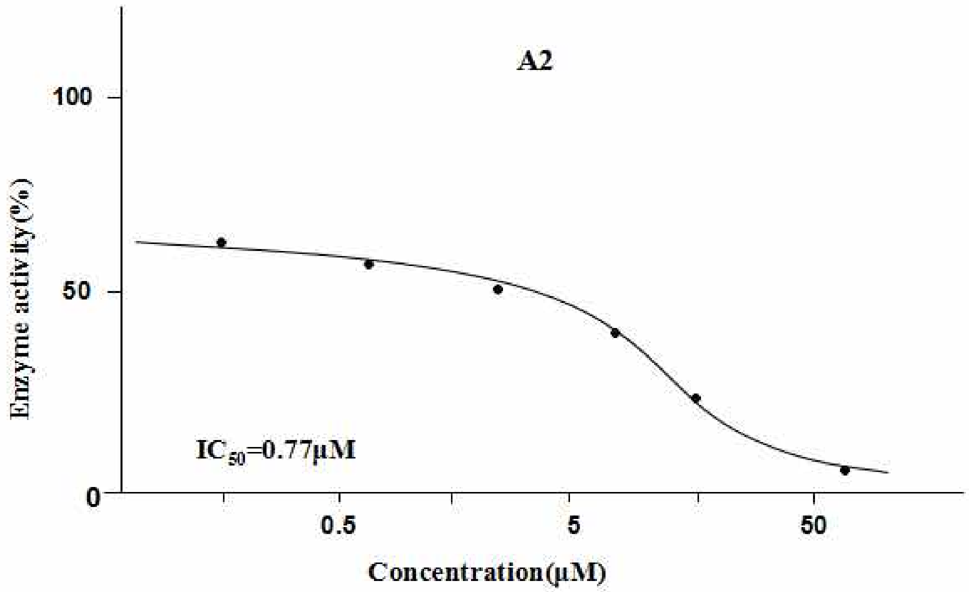
A2
100
50
0
Enzyme activity(%)
IC$_{50}$=0.77μM
0.5
5
50
Concentration(μM)

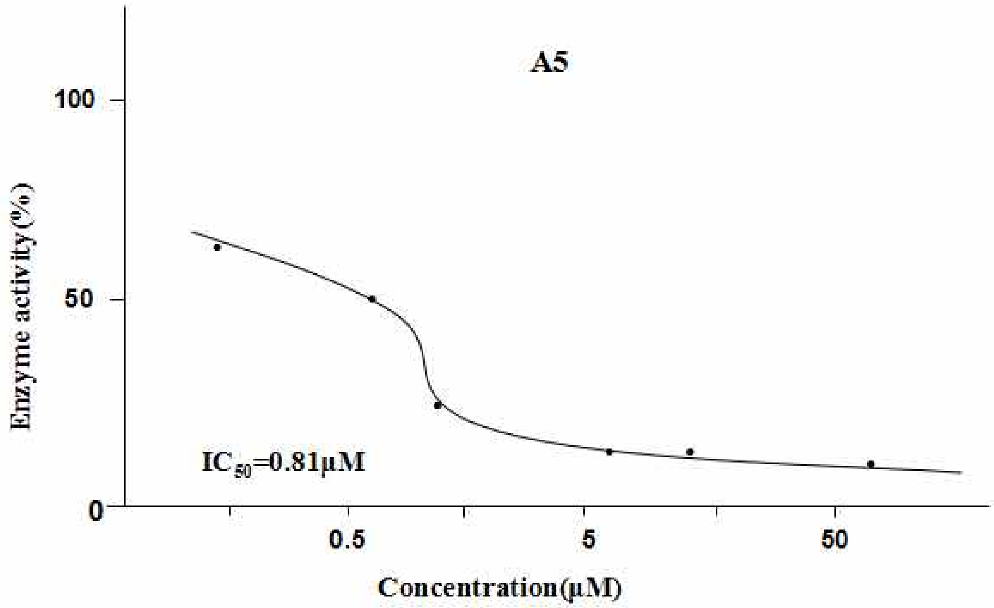
A5
100
50
0
Enzyme activity(%)
IC$_{50}$=0.81μM
0.5
5
50
Concentration(μM)

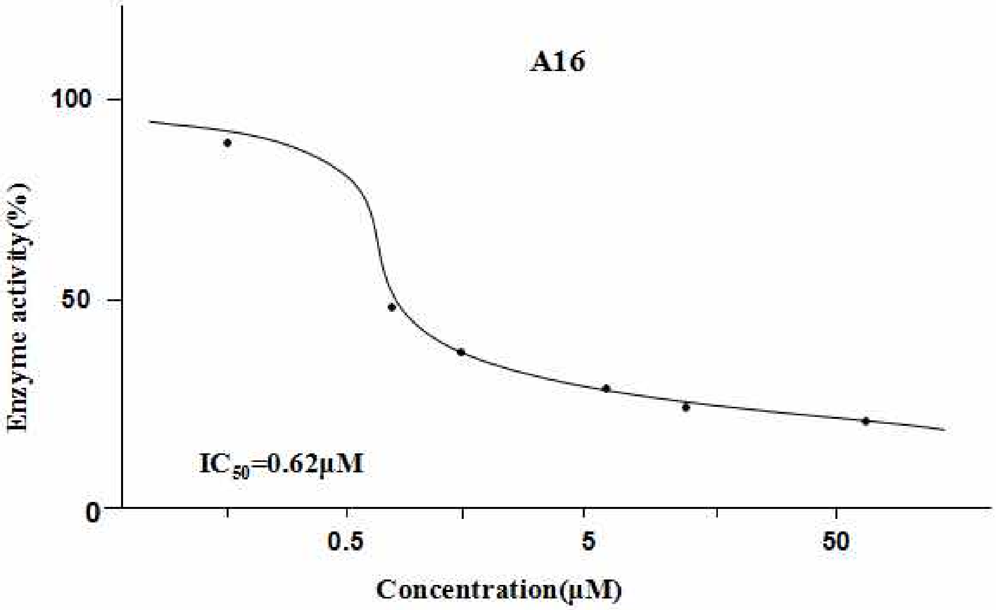
A16
100
50
0
Enzyme activity(%)
IC50=0.62μM
0.5
5
50
Concentration(μM)

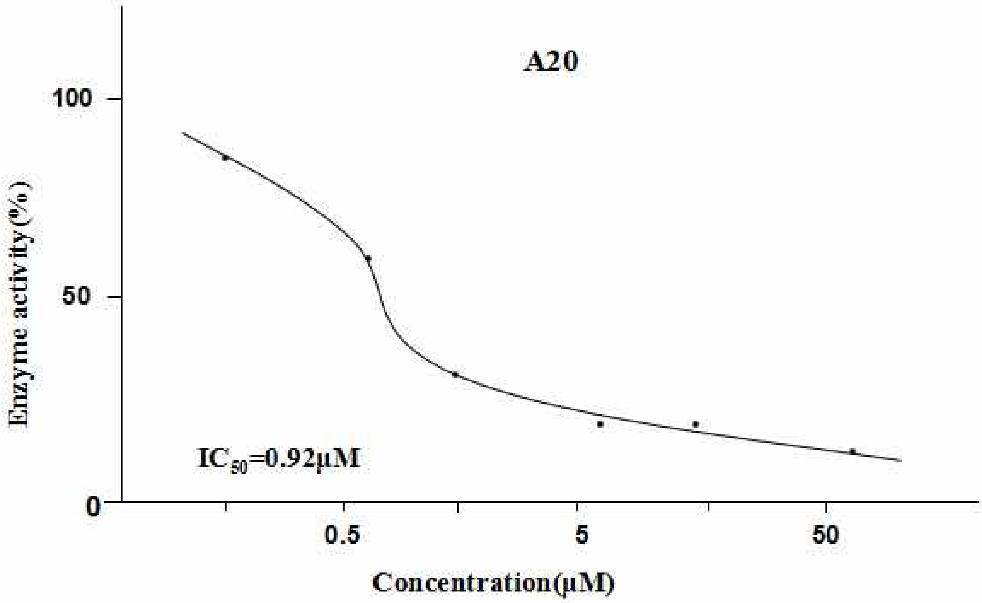
A20
100
50
0
Enzyme activity(%)
IC50=0.92μM
0.5
5
50
Concentration(μM)

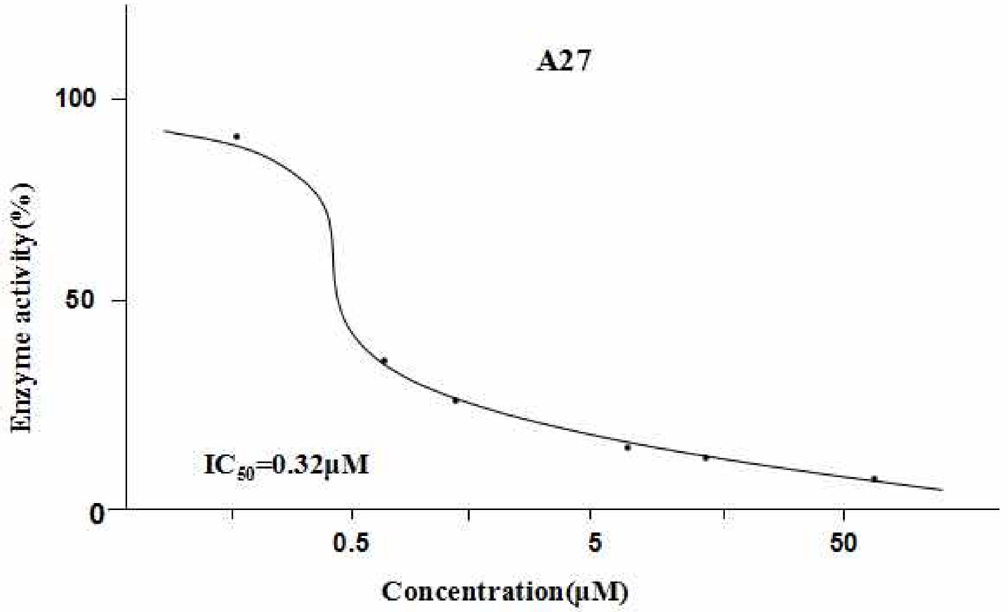
A27
100
50
0
Enzyme activity(%)
IC50=0.32μM
0.5
5
50
Concentration(μM)

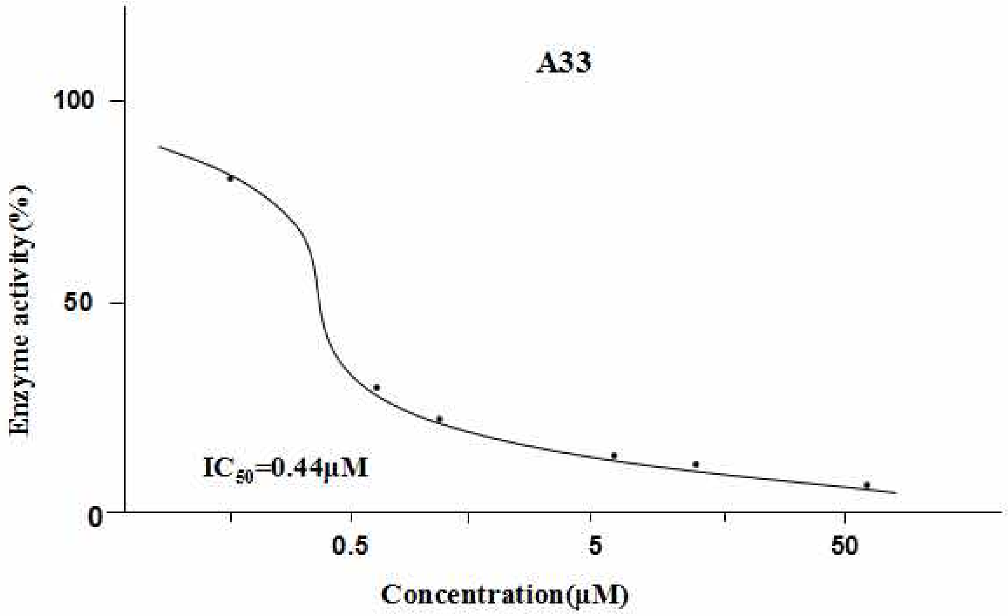
A33
100
50
0
Enzyme activity(%)
IC50=0.44μM
0.5
5
50
Concentration(μM)

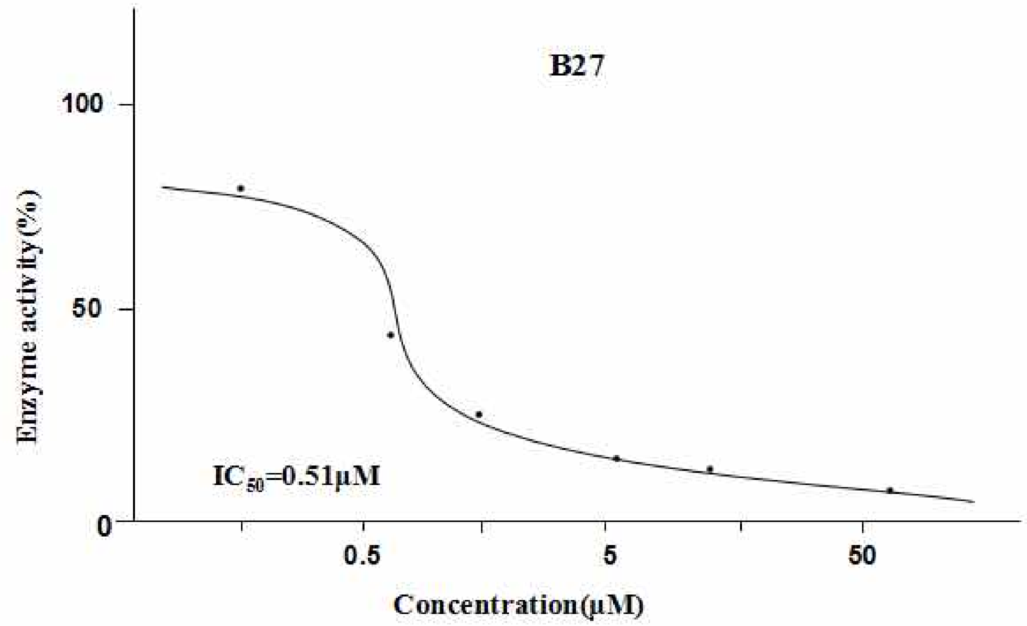
B27
100
50
0
Enzyme activity(%)
IC50=0.51μM
0.5
5
50
Concentration(μM)

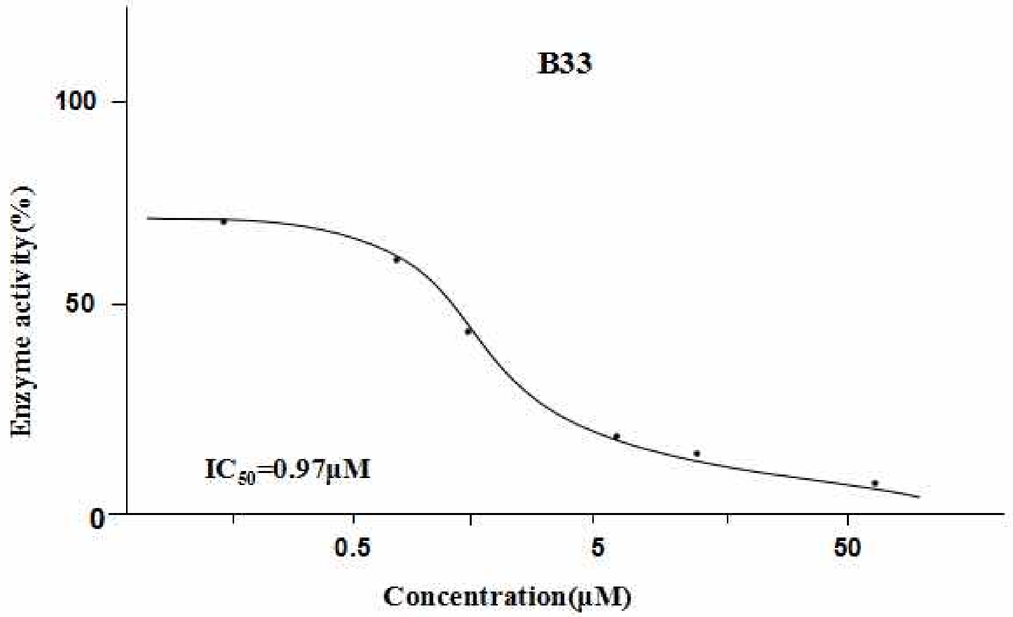
B33
100
50
0
Enzyme activity(%)
IC50=0.97μM
0.5
5
50
Concentration(μM)
